# Global, regional, and national trends in ischaemic stroke burden and risk factors among adults aged 20 + years (1990–2021): a systematic analysis of data from the Global Burden of Disease study 2021 with projections into 2050

**DOI:** 10.3389/fpubh.2025.1567275

**Published:** 2025-04-25

**Authors:** Sibo Liu, Yanzhao Li, Xiaoyan Lan, Long Wang, Hang Li, Dean Gu, Mengxing Wang, Jinjie Liu

**Affiliations:** ^1^Department of Intensive Care Unit, Central Hospital of Dalian University of Technology, Dalian, China; ^2^Department of Neurosurgery, Affiliated Zhongshan Hospital of Dalian University, Dalian, China; ^3^Department of Neurology, Central Hospital of Dalian University of Technology, Dalian, China; ^4^School of Computer and Communication Engineering, University of Science and Technology Beijing, Beijing, China; ^5^Department of Geriatrics, Affiliated Dalian Friendship Hospital of Dalian Medical University, Dalian, China; ^6^Department of Neurology, Qingdao Central Hospital, University of Health and Rehabilitation Sciences, Qingdao, China; ^7^China National Clinical Research Center for Neurological Diseases, Beijing, China; ^8^Department of General Medicine, Central Hospital of Dalian University of Technology, Dalian, China

**Keywords:** ischaemic stroke, prevalence, mortality, disability-adjusted life years, risk factors, average annual percentage change

## Abstract

**Background:**

The objective was to provide standardized, comprehensive, and updated estimates of the global, regional, and national burdens of and risk factors for ischaemic stroke (IS) in adults aged 20 years and older.

**Methods:**

This was a population-based study (Global Burden of Disease, Injuries and Risk Factors Study 2021). Adults aged 20 years and older from 204 countries and territories and 811 subnational locations from 1990 to 2021 were included. The primary outcomes were IS-related age-standardized prevalence, mortality, disability-adjusted life years (DALYs), average annual percentage change (AAPC), and risk factors associated with DALYs.

**Results:**

From 1990 to 2021, the global age-standardized prevalence of IS decreased from 1,309 (95% UI 1,151 to 1,481) to 1,266 (95% UI 1,120 to 1,423) cases per 100,000 population, with an average annual decrease of −0.12%. However, the prevalence increased notably in the middle sociodemographic index (SDI) regions and East Asia but remained stable in Southeast Asia. The total number of IS cases still increased significantly from 33.2 million to 68.4 million. During the same period, the overall age-standardized mortality for IS decreased from 116 to 70 cases per 100,000 population, with an average annual change of −1.60%. Similarly, the overall age-standardized DALYs for IS decreased by 35%, with an average annual change of −1.36%. The decrease in both the age-standardized mortality and DALY rates was correlated with the SDI, with the most rapid decrease occurring in high-SDI regions. Conversely, in subregions of southern sub-Saharan Africa, an increase was observed. Males consistently faced a greater burden of IS across all subgroups. High systolic blood pressure and high low-density lipoprotein cholesterol (LDL-C) levels consistently represented the most significant risk factors contributing to DALYs from 1990 to 2021.

**Conclusion:**

Globally, the total IS caseload has increased. Targeted strategies, such as metabolic risk control in high-SDI regions, low-cost interventions in middle-/low-SDI regions, and improved neuroimaging infrastructure in sub-Saharan Africa, are needed. Future research should focus on subtype-specific burdens, the legacy effects of COVID-19, and intervention cost effectiveness to guide policymakers in developing efficient strategies to combat the global burden of ischaemic stroke.

## 1 Introduction

The prevalence, mortality, disability-adjusted life years (DALYs), and DALYS attributable to ischaemic stroke (IS) are continuously changing on a global scale, and these changes have not been thoroughly evaluated among adults aged 20 years and older (1, 2). Standardized, comprehensive and up-to-date estimates of these metrics stratified by sex, age, sociodemographic index (SDI), region, country and territory are essential. Such an analysis would provide crucial evidence to inform healthcare policy, establish benchmarks for decision-making, and guide resource allocation at the global, regional and local levels (3).

A significant increase of 87.55% in the incidence of IS was reported globally from 1990 to 2019, although the age-standardized incidence rate (ASIR) has decreased, with an estimated annual percentage change (EAPC) of −0.43. Notably, high-middle- and middle-SDI regions present higher ASIRs, age-standardized death rates (ASDRs) and DALYs attributed to ISs than other SDI regions do (4, 5). However, three key gaps remain unaddressed: (1) Temporal Extensions: Existing projections end in 2030, leaving a 20-year gap (2030–2050) unexamined, despite the accelerating impact of ageing populations and shifting risk factors (e.g., rising LDL-C levels in East Asia); (2) Socioeconomic Heterogeneity: Prior analyses lack granular stratification by SDI quintiles and subnational regions, particularly in sub-Saharan Africa and East Asia, where the IS burden diverges sharply from global trends; 3. Post-Pandemic Dynamics: No study has incorporated data beyond 2019 to assess the potential influence of COVID-19-related factors, such as delayed healthcare access or disrupted hypertension management, on IS outcomes.

Our study addresses these gaps by leveraging the Global Burden of Disease, Injuries and Risk Factors Study (GBD) 2021. Our objective was to explore the trends in the prevalence, mortality and DALYs of IS among adults aged 20 years and older from 1990 to 2021. We used a comprehensive dataset spanning from 1990 to 2021 that encompasses 204 countries and territories, distributed across 21 GBD regions and categorized into five SDI groups. Additionally, we conducted a thorough assessment of all risk factors linked to IS, drawing from the 88 risk factors listed in the GBD hierarchy. To project the future trajectory of these trends, we employed a Bayesian age-period-cohort (BAPC) model, which allowed for a nuanced prediction of the changing patterns from 2021 to 2050. This modeling approach is crucial for anticipating the evolving landscape of IS and informing proactive healthcare strategies.

## 2 Methods

### 2.1 Study population and data collection

By incorporating data from 100,983 sources, the GBD 2021 database detailed 371 diseases and injuries, along with 88 risk factors and their health impacts. We estimated the global trends in the IS burden through age-standardized metrics encompassing prevalence, mortality, DALYs, and DALYs attributable to risk factors. This study included patients aged 20 years or older with IS, which is characterized by rapidly developing clinical signs of (usually focal) disturbances of cerebral function lasting over 24 h or leading to death due to the occlusion of blood flow to part of the brain due to a thrombus or embolism resulting in neurological dysfunction, as defined by the World Health Organization (WHO) (6). DALYs was defined as years of healthy life lost to premature death and disability. We extracted data on DALYs attributed to risk factors to assess the impact of particular risk factors on IS. From 1990 to 2021, we analysed the metrics stratified by age (every 5 years from 20 to 24 to 95 + years of age), sex, and region. Details on the GBD 2021 criteria, literature search and data extraction methods can be found in other papers (7, 8).

### 2.2 Fatal disease modeling

In the GBD 2021, vital registry data were utilized to model deaths from IS. The age-standardized mortality rate for IS was calculated per 100,000 populations via the Cause of Death Ensemble Model (CODEm). To make the random time series data more consistent with regional patterns, methods to reduce noise in cause-of-death data were updated (section 5.3 of the Appendix) (9). Deaths that were coded as having unclear intermediate causes in vital registration systems, or for which IS was not specified, were recoded via a multicause methodology (section 5 of the Appendix).

### 2.3 Nonfatal burdens modeling

To estimate the nonfatal burdens of IS, the prevalence, age-standardized prevalence, and age-standardized DALYs of IS were generated via the DisMod-MR 2.1 (Disease Model-Bayesian Meta-regression) modeling tool (10). This approach considers the factors of sex, region, time, and age group to provide a comprehensive disease parameter estimate. Leveraging geospatial modeling, DisMod-MR 2.1 is not only able to process existing epidemiological data but also estimates disease prevalence for data-scarce regions using data from well-studied areas as priors, which allowed us to generate comprehensive prevalence and morbidity estimates (section 4 of the Appendix) (7, 11).

### 2.4 Risk factor assessment

To assess the impact of 23 risk factors related to IS, we calculated the population attribution score (PAF) for DALYs in adults aged 20 years and older with data from the GBD 2021 (9). The analysis was performed within the comparative risk assessment framework (CRA), utilizing four key datasets: the burden of IS, the exposure level for each risk factor, the relative risk of IS associated with each risk factor, and the theoretical minimum risk exposure level (TMREL), which represents the level of exposure that reduces the risk of each person in a population to the lowest level (12). These data sources can be accessed through the interactive Global Health Data Exchange (GHDx) tool.^1^ Notably, owing to data limitations, the impact of the coronavirus disease 2019 (COVID-19) pandemic on risk factors or health outcomes was not formally included or quantified in this analysis.

The GBD categorizes risk factors into four levels of specificity, from the general level (level 1) to the specific level (level 4). The level 1 risk was calculated, which included environmental/occupational risks and behavioral and metabolic risks. As mentioned elsewhere, the PAFs for the risk factor groups considered interactions among the individual risk factors within each group (9). The percentages and numbers of DALYs were not mutually exclusive, and the total PAF attributed to risk factors could exceed 100%, given that many of these risk factors are partially or entirely mediated by another risk factor because many risk factors are interrelated or mediated by other risk factors. More information on risk factors, groups, and definitions can be found in the Appendix (section 6).

### 2.5 Health inequality and predictive analyses

The disparity in the burden of IS across nations relative to their SDI was assessed via the slope index of inequality and the concentration index, as outlined by the WHO in 2023 (13). The slope index of inequality was determined by regressing the prevalence of IS individuals aged 20 years and older against a sociodemographic development scale, which was derived from the median value of the SDI-ranked population’s cumulative classification spectrum. Similarly, the concentration index of health inequality was formulated by aligning a Lorenz curve with the cumulative distribution of disease prevalence across the SDI-ranked population strata, followed by computing the area under the curve, which represents the degree of inequality (section 7 of the Appendix).

To forecast the number and incidence of new IS cases from 2022 through 2050, Bayesian BAPC models were employed. In these models, we utilized the integrated nested Laplace approximation (INLA) method for comprehensive Bayesian analysis (14). The BAPC models could generate both age-specific and age-standardized predicted rates. The INLA algorithm was implemented using the R-INLA package (version 23.09.09) to efficiently approximate posterior distributions. Model convergence was assessed via trace plots and Geweke diagnostics. More information can be found in the Appendix (section 8). While sensitivity analyses under alternative scenarios (e.g., accelerated improvements in stroke care or lifestyle modifications) could enhance robustness, such analyses were limited by the lack of granular data on future policy interventions or technological advancements. Therefore, our projections reflect a baseline scenario, which is consistent with prior GBD studies (11). Future work should incorporate scenario-based modeling when region-specific intervention data become available.

### 2.6 Data source and presentation

For the GBD 2021 study, we sourced data from 21,353 vital registration sources, 2,570 cause-of-death sources, 394 nonfatal health outcome sources, 186 relative risk sources, and 11,538 exposure and TMREL sources. For more detailed information about the data sources used in our analysis, please refer to the GBD 2021 source tools.^2^

The estimates are presented in both absolute numbers and age-standardized rates per 100,000 population, accompanied by a 95% uncertainty interval (UI). The data were categorized by age, sex, GBD region (21 regions), GBD super region (7 super regions), and SDI level (5 levels). The SDI for each country was calculated via the most recent 2021 data (section 11 of the Appendix). The SDI is a comprehensive indicator that captures the social and demographic development of a region and is scored on a scale from 0.05 (lowest) to 1 (highest), with 1 indicating the highest education level, the highest per capita income, and the lowest fertility rate.

### 2.7 Statistical analysis

This study presented a thorough descriptive analysis to delineate the disease burden of IS in adults aged 20 years on a global scale. We conducted a comparative analysis of age-standardized prevalence, mortality, and DALYs across various dimensions, including age, sex, region, and country groups. The age-standardized rates for each region were calculated with data from the GBD study and aligned with the world standard population as referenced in the GBD 2021 report. The corresponding 95% confidence intervals (CIs) were derived to allow cross-regional comparisons. Furthermore, we estimated the average annual percentage change (AAPC) for each year, employing a join-point regression model to track the progression of the disease burden over time (15). All the results are expressed per 100,000 population, and the specific calculation formula is as follows.


$$\mathrm{Age}-\mathrm{standardized}\;\mathrm{rate}=\frac{\overset{A}{\underset{i=1}{\sum }}a_{i}w_{i}}{\overset{A}{\underset{i=1}{\sum }}w_{i}}$$

Where:

a_i_ is the age specific rate and w_i_ is the weight in the same age subgroup of the chosen reference standard population (in which i denotes the i^th^ age class) and A is the upper age limit.

The AAPC is a statistical measure that captures the overall trend of change in a variable across a defined time span (16). In this study, the AAPC was calculated with slope coefficients from a base join-point regression model, which spans from 1990 to 2021. These coefficients were then converted into an annual percentage change. Significance testing was conducted via a Monte Carlo permutation method, which can account for the variability estimated at each data point, or a Poisson model was employed to assess variation. The AAPC indicates whether a variable is increasing, decreasing, or remains stable on an annual basis. If the estimate of the annual percentage change and its 95% CI are consistently greater than zero, it signifies a significant upward trend in the variable. Conversely, if these values are consistently less than zero, a significant downward trend is indicated. The AAPC was calculated via the following formula:


$$\mathrm{AAPC}=\left\{\exp\left(\frac{\sum w_{i}b_{i}}{\sum w_{i}}\right)-1\right\}\times 100$$

Where:

b_i_ is the slope coefficient for the i^th^ segment with i indexing the segments in the desired range of years, and w_i_ is the length of each segment in the range of years.

All statistical analyses were conducted using R (version 4.4.1), Joinpoint Regression Program (version 5.2.0.0), GraphPad Prism (version 9.5.0), and Stata (version MP 17).

## 3 Results

### 3.1 Global trends

Globally, the number of adults aged 20 years or older living with IS increased from 33.2 million to 68.4 million (106%) from 1990 to 2021. During this period, the age-standardized prevalence of IS among this age group showed a slight decrease of 3%, from 1,309 to 1,266 cases per 100,000 population, with an average annual trend of −0.12% (Table 1). Furthermore, the proportion of IS patients in this age group steadily increased, from 95.7% in 1990 to 97.7% in 2021 (Supplementary Figure 1). Both the overall population prevalence of IS and the prevalence within this specific age group demonstrated a downward trend from 1990 to 2021 (Supplementary Figure 2).

**Table 1:** Age standardized prevalence and AAPC of ischaemic stroke in people aged ≥20 years at global and regional level, 1990–2021.

	Prevalence (95% UI)				
	No of people with IS in 1990 (000 s)	Age standardized rate in 1990 (per 100,000)	No of people with IS in 2021 (000 s)	Age standardized rate in 2021 (per 100,000)	AAPC (95% CI)
Global	33,170 (29,153 to 37,541)	1,309 (1,151 to 1,481)	68,357 (60,490 to 76,787)	1,266 (1,120 to 1,423)	−0.12 (−0.13 to −0.10)
Sex:					
Female	17,007 (14,936 to 19,255)	1,244 (1,092 to 1,408)	33,778 (29,793 to 38,100)	1,180 (1,040 to 1,331)	−0.18 (−0.19 to −0.17)
Male	16,163 (14,172 to 18,326)	1,403 (1,232 to 1,591)	34,578 (30,672 to 38,817)	1,372 (1,281 to 1,539)	−0.08 (−0.09 to −0.06)
Age group (years):					
20–24	794 (667 to 932)	161 (136 to 189)	871 (748 to 1,000)	146 (125 to 168)	−0.33 (−0.33 to −0.32)
25–29	936 (799 to 1,091)	211 (180 to 247)	1,140 (995 to 1,297)	194 (169 to 220)	−0.28 (−0.30 to −0.26)
30–34	1,063 (908 to 1,242)	276 (236 to 322)	1,554 (1,356 to 1780)	257 (224 to 294)	−0.23 (−0.25 to −0.21)
35–39	1,278 (1,101 to 1,458)	363 (313 to 414)	1954 (1710 to 2,189)	348 (305 to 390)	−0.14 (−0.16 to −0.12)
40–44	1,425 (1,225 to 1,635)	497 (428 to 571)	2,433 (2,131 to 2,757)	486 (426 to 551)	−0.08 (−0.09 to −0.06)
45–49	1741 (1,521 to 1969)	750 (655 to 848)	3,447 (3,052 to 3,869)	728 (645 to 817)	−0.10 (−0.12 to −0.07)
50–54	2,492 (2,165 to 2,828)	1,172 (1,019 to 1,330)	4,901 (4,293 to 5,528)	1,102 (965 to 1,243)	−0.20 (−0.27 to −0.14)
55–59	3,241 (2,844 to 3,629)	1750 (1,536 to 1960)	6,530 (5,751 to 7,261)	1,650 (1,453 to 1835)	−0.19 (−0.23 to −0.15)
60–64	4,153 (3,669 to 4,690)	2,586 (2,284 to 2,920)	7,623 (6,782 to 8,526)	2,382 (2,119 to 2,664)	−0.27 (−0.31 to −0.24)
65–69	4,486 (3,986 to 5,006)	3,629 (3,224 to 4,050)	9,473 (8,432 to 10,591)	3,434 (3,057 to 3,840)	−0.20 (−0.24 to −0.15)
70–74	4,105 (3,621 to 4,662)	4,849 (4,277 to 5,507)	9,534 (8,415 to 10,750)	4,632 (4,088 to 5,223)	−0.16 (−0.19 to −0.13)
75–79	3,712 (3,342 to 4,162)	6,031 (5,430 to 6,761)	7,856 (7,038 to 8,772)	5,957 (5,337 to 6,651)	−0.05 (−0.08 to −0.02)
80–84	2,372 (2,126 to 2,663)	6,705 (6,011 to 7,527)	6,071 (5,459 to 6,785)	6,932 (6,233 to 7,747)	0.09 (0.04 to 0.15)
85–89	1,011 (879 to 1,149)	6,690 (5,814 to 7,604)	3,265 (2,880 to 3,699)	7,140 (6,299 to 8,090)	0.19 (0.12 to 0.26)
90–94	289 (244 to 341)	6,750 (5,685 to 7,954)	1,288 (1,098 to 1,489)	7,200 (6,137 to 8,325)	0.18 (0.11 to 0.26)
≥95	70 (57 to 85)	6,890 (5,614 to 8,376)	416 (349 to 493)	7,624 (6,410 to 9,038)	0.30 (0.24 to 0.36)
SDI level:					
High	9,940 (8,884 to 11,065)	1,437 (1,281 to 1,602)	15,694 (14,225 to 17,216)	1,251 (1,131 to 1,376)	−0.45 (−0.47 to −0.43)
High-middle	8,900 (7,764 to 10,115)	1,396 (1,218 to 1,590)	16,931 (14,861 to 19,168)	1,384 (1,214 to 1,568)	−0.04 (−0.06 to −0.01)
Middle	8,028 (6,815 to 9,323)	1,176 (996 to 1,370)	21,791 (18,898 to 24,974)	1,298 (1,124 to 1,490)	0.30 (0.27 to 0.33)
Low-middle	4,319 (3,714 to 4,959)	1,048 (897 to 1,210)	9,812 (8,613 to 11,058)	1,031 (902 to 1,166)	−0.05 (−0.07 to −0.04)
Low	1945 (1728 to 2,178)	1,289 (1,140 to 1,449)	4,073 (3,687 to 4,477)	1,161 (1,046 to 1,282)	−0.33 (−0.35 to −0.31)

AAPC = average annual percentage change; CI = confidence interval; SDI = sociodemographic index; IS = ischaemic stroke; UI = uncertainty interval.

The burden of IS, which encompasses mortality and all-cause DALYs among adults aged 20 years or older, remained relatively stable, with minor fluctuations, although there was a noticeable decrease between 2020 and 2021 (Supplementary Figure 3). Notably, the age-standardized mortality rate for IS in this age group decreased by 40%, from 116 (104–125) cases per 100,000 population in 1990 to 70 (62–77) cases per 100,000 population in 2021, with an average annual trend of −1.6% (Supplementary Table 1). The number of IS-related DALYs in this age group increased by 54% over the same period. However, when standardized by age, the number of DALYs showed a 35% reduction, with an annual trend of −1.36% (Supplementary Table 2).

### 3.2 Global trends by sex

From 1990 to 2021, the global prevalence of IS among adults aged 20 years and older increased for both females (17.0 to 33.8 million) and males (16.2 to 34.6 million). However, when standardized by age, the prevalence showed a more pronounced decrease among females, decreasing from 1,244 to 1,180 cases per 100,000 population, compared with the decrease observed among males, which decreased from 1,403 to 1,372 cases per 100,000 population (AAPC of −0.18% for females vs. −0.08% for males) (Table 1).

During the same period, the age-standardized mortality rate for IS also decreased more obviously among females, from 111 to 61 cases per 100,000 population, than in males, from 121 to 81 cases per 100,000 population (AAPC of −1.28% for females vs. −1.88% for males) (Supplementary Table 1). Similarly, the age-standardized DALYs showed a more substantial decrease among females, decreasing by 40% (from 1,881 to 1,130 cases per 100,000 population, than in males (29% decrease; from 2,176 to 1,536 cases per 100,000 population) (Supplementary Table 2).

This disparity between the sexes was observed across all SDI levels and age subgroups, with males generally having a greater burden of IS than females did, especially in countries with high-middle SDIs (Supplementary Figures 4, 5).

### 3.3 Global trends by age subgroups

From 1990 to 2021, a global increase in the age-standardized prevalence of IS across all age subgroups was observed. Most of these subgroups demonstrated a decrease of more than 100%, with a dramatic increase observed in individuals aged 80 years and older (Table 1).

In 2021, the age-standardized mortality rate for IS increased with age, starting at 0.24 cases per 100,000 for individuals aged 20–24 years and ending at 2,530 cases per 100,000 for those aged over 95 years (Supplementary Table 1). From 1990 to 2021, the age-standardized mortality rate of IS decreased across all age groups and tended to be most significant among individuals aged 75–79 years, from 2,290 to 1,367 cases per 100,000 population, with an annual trend of −1.78% (95% CI −1.92 to −1.65).

The number of age-standardized DALYs for IS also increased with age. Across all age subgroups, the number of age-standardized DALYs decreased at a noticeable rate from 1990 to 2021, especially among adults aged over 75 years (Supplementary Table 2). In 2021, the highest number of age-standardized DALYs for IS was observed in individuals aged 95 years and older, reaching 22,001 cases per 100,000 population (Supplementary Table 2).

### 3.4 Global trends by sociodemographic index

Between 1990 and 2021, the age-standardized prevalence of IS among adults aged 20 years and older decreased in most SDI subgroups, except for the middle-SDI group, whose prevalence increased (AAPC 0.30% [95% CI 0.27 to 0.33]). In 2021, the highest age-standardized prevalence rates were observed in high–middle-SDI countries, at 1,384 cases per 100,000 population (Table 1; Supplementary Figures 7A, 8A).

The age-standardized mortality rates for IS decreased across all subgroups from 1990 to 2021, with the decrease being more pronounced in countries with high SDIs (APCC −3.26% [95% CI −3.49 to −3.03]), which was seven times faster than the rate in low-SDI countries (APCC -0.43% [95% CI −0.56 to −0.30]). In 2021, high-middle-SDI countries had the highest age-standardized mortality rate at 95 cases per 100,000 population, which was more than three times greater than that of high-SDI countries, which had a rate of 31 cases per 100,000 population (Supplementary Table 1; Supplementary Figures 4, 5).

The age-standardized DALYs for IS among adults aged 20 years and older clearly decreased across all the SDI subgroups, with the most significant decrease in high-SDI countries (APCC −2.70% [95% CI −2.87 to 2.53]). The impact of IS on the number of DALYs became more pronounced as the SDI exceeded the threshold of 0.75. In 2021, high-middle-SDI countries had the highest number of age-standardized DALYs at 1,700 years per 100,000 population, whereas high-SDI countries had the lowest number of DALYs at 620 years per 100,000 population (620 per 100,000) (Supplementary Table 2; Supplementary Figure 9).

### 3.5 Regional trends

From 1990 to 2021, the age-standardized prevalence of IS increased only in East Asia (AAPC 0.89% [95% CI 0.85 to 0.94]), remained steady in Southeast Asia (AAPC 0.00% [95% CI 0.00 to 0.01]), and decreased in most of the 21 regions (Supplementary Table 3). The most reduced burden was estimated in Tropical Latin America (AAPC −1.09% [95% CI −1.12 to −1.06]), Southern Latin America (AAPC −0.95% [95% CI −0.98 to −0.92]), and high-income Asia Pacific (AAPC −0.92% [95% CI −0.95 to −0.89]) (Supplementary Figure 10). In 2021, the highest rates were in southern sub-Saharan Africa (1,741 per 100,000), western sub-Saharan Africa (1,603 per 100,000), and East Asia (1,581 per 100,000) (Supplementary Table 3). When the analysis was stratified by sex, no significant differences were observed across different regions (Supplementary Table 4).

The age-standardized mortality rate for IS increased only in southern sub-Saharan Africa from 1990 to 2021, with an annual trend of 0.82% (95% CI 0.32 to 1.31). In contrast, the most significant decreases were observed in high-income Asia Pacific (AAPC −4.41% [95% CI −4.85 to −3.97]), Western Europe (AAPC −4.19% [95% CI −4.35 to −4.02]), and Australasia (AAPC −3.78% [95% CI −4.01 to −3.56]) (Supplementary Table 5).

Most regions experienced a decrease in the number of age-standardized DALYs in adults aged 20 years and older from 1990 to 2021, except for southern sub-Saharan Africa, where experienced an increase (AAPC 0.52% [95% CI 0.07 to 0.98]). The greatest reduction in age-standardized DALYs was estimated in Western Europe (AAPC −3.73% [95% CI −3.85 to −3.60]) (Supplementary Table 6; Supplementary Figure 10). In 2021, the highest numbers of DALYs were noted in Eastern Europe, Central Asia, North Africa and the Middle East. The lowest numbers of DALYs were observed in Australasia, Western Europe, and Andean Latin America. When the analysis was stratified by sex, no significant differences were found across different regions (Supplementary Figure 11).

### 3.6 National Trends

**Figure 1:**
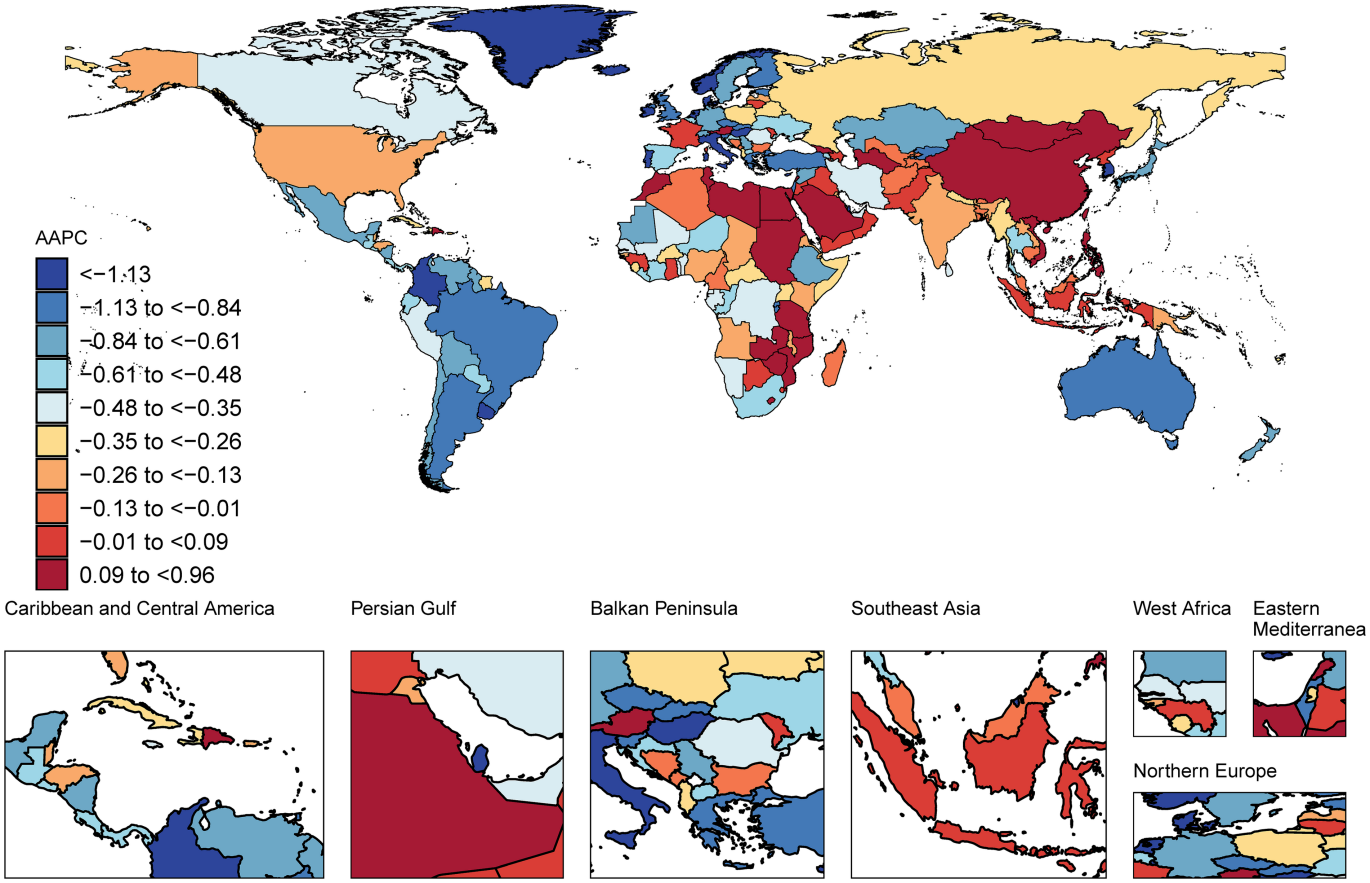
Map showing average annual percentage change in global prevalence of ischaemic stroke among people aged ≥20 years, 1990–2021.

From 1990 to 2021, the greatest decrease in the age-standardized prevalence of IS was observed in Portugal, with an average annual trend of −2.82% [95% CI −2.91 to −2.73], followed by Singapore (AAPC −2.32% [95% CI −2.38 to −2.26]) and the Republic of Korea (AAPC −2.21% [95% CI −2.29 to −2.13]). Conversely, the most significant increase in the age-standardized prevalence was observed in China, with an average annual trend of 0.96% [95% CI 0.91 to 1.01], followed by Turkmenistan (AAPC 0.67% [95% CI 0.62 to 0.73]), Egypt (AAPC 0.66% [95% CI 0.64 to 0.68]), and Lesotho (AAPC 0.62% [95% CI 0.60 to 0.64]). In 2021, the highest age-standardized prevalence was observed in Ghana, at 2,475 cases per 100,000 population, whereas the lowest was observed in Cyprus, at 475 cases per 100,000 population (Figure 1; Supplementary Table 7).

**Figure 2:**
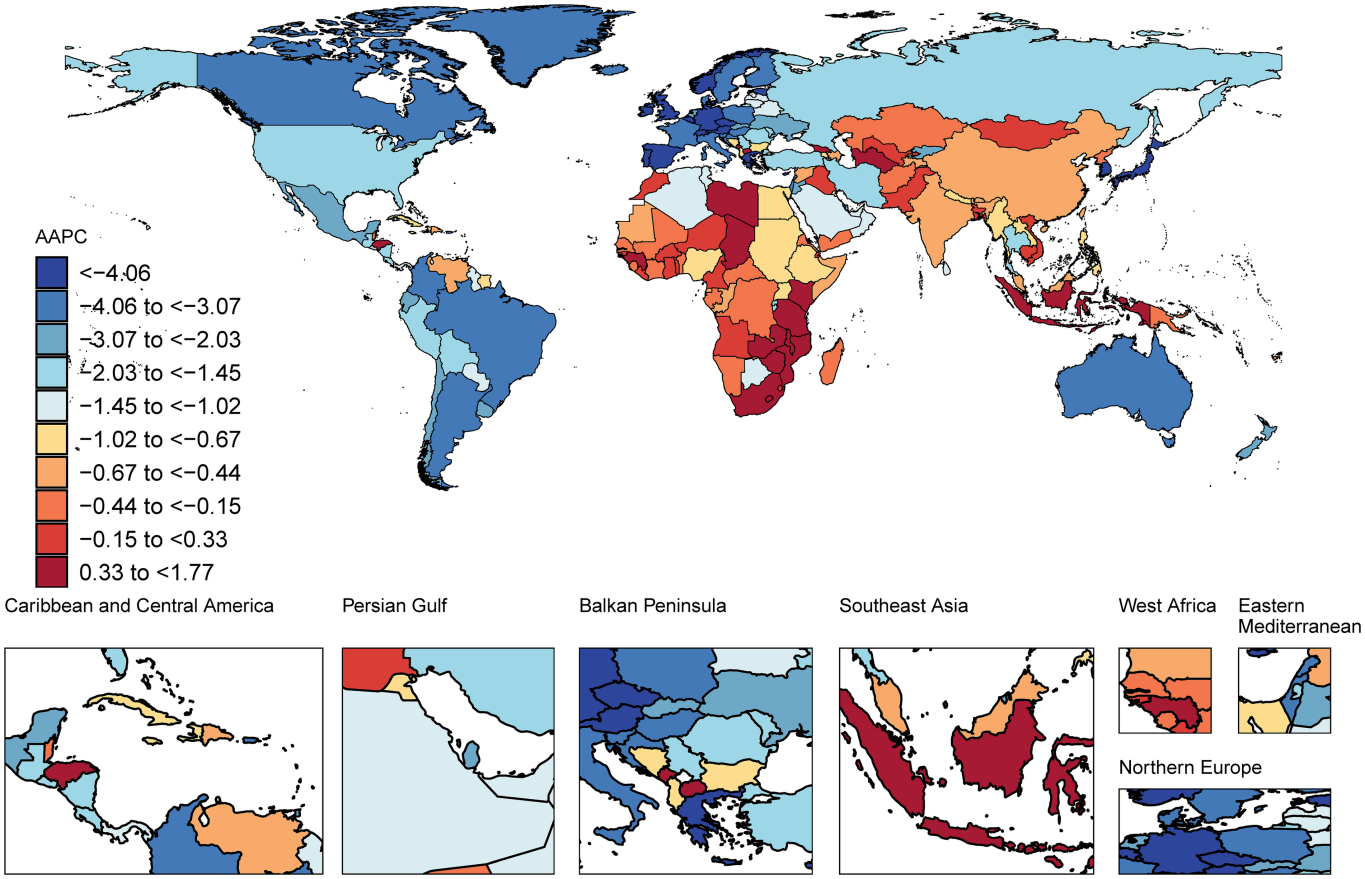
Map showing average annual percentage change in global mortality among people with ischaemic stroke aged ≥20 years, 1990–2021.

The age-standardized mortality rate for IS decreased in most countries among this age group, with the greatest increase in Singapore, with an AAPC of −6.58 [95% CI −7.62 −5.51], followed by Portugal, with a value of −5.64 [95% CI −6.17 to −5.10], and Luxembourg, with a value of −5.59 [95% CI −6.27 to −4.90]. In 2021, North Macedonia presented an age-standardized mortality rate of 344 cases per 100,000 population, whereas Singapore presented the lowest rate of 11 cases per 100,000 population (Figure 2; Supplementary Table 7).

**Figure 3:**
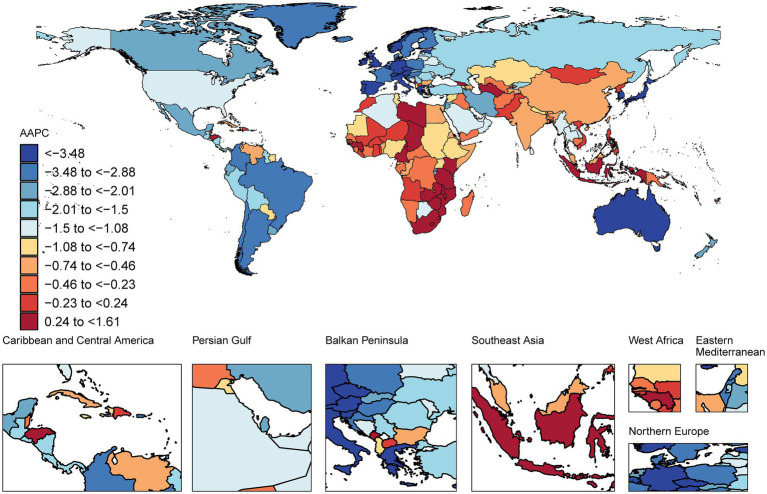
Map showing average annual percentage change in global DALYs among people with ischaemic stroke aged ≥20 years, 1990–2021.

From 1990 to 2021, the most significant decrease in the number of age-standardized DALYs for IS was observed in Portugal (AAPC −5.59% [95% CI −6.05 to −5.12]), followed by Singapore (AAPC −5.44% [95% CI −6.51 to −4.36]) and Luxembourg (AAPC −5.34% [95% CI −5.93 to −4.75]). The most significant increases in the number of age-standardized DALYs were observed in Lesotho (AAPC 1.61% [95% CI 1.22 to 1.99]), Montenegro (AAPC 1.27% [95% CI 0.85 to 1.69]), and Zimbabwe (AAPC 1.16% [95% CI 0.51 to 1.81]). In 2021, North Macedonia presented the highest number of age-standardized DALYs for adults, at 4,812 years per 100,000 population, whereas Puerto Rico presented the lowest, at 284 years per 100,000 population (Figure 3; Supplementary Table 7).

### 3.7 DALYs attributable to risk factors

In 2021, 89.2% [82.8 to 93.6] of the age-standardized DALYs among adults aged 20 years and older were attributed to 23 of the 88 risk factors in the GBD study. From 1990 to 2021, the total number of IS-related DALYs due to these risk factors increased from 40.9 million [37.3–44.7] to 62.3 million [55.7–68.7]. In addition to the decrease in the high-SDI subgroup, with the number of DALYs decreasing from 9.1 million [8.2–9.8] in 1990 to 7.6 million [6.6–8.6] in 2021, there was an increase in the low- to high-middle-SDI subgroups. The cumulative DALYs for these four subgroups were estimated to increase from 31.8 million in 1990 to 54.6 million in 2021 (Table 2; Supplementary Table 8).

**Table 2:** Risk factors for ischaemic stroke related DALYs among people aged ≥20 years in 2021.

	Globally		High SDI		High-middle SDI		Middle SDI		Low-middle SDI		Low SDI	
	Absolute NO (00000 s)	Percent	Absolute NO (00000 s)	Percent	Absolute NO (00000 s)	Percent	Absolute NO (00000 s)	Percent	Absolute NO (00000 s)	Percent	Absolute NO (00000 s)	Percent
**Environmental risks**	247.6 (191.2 to 302.9)	35.2% (27.4 to 42.5)	17.5 (13.0 to 22.2)	19.5% (14.6 to 24.2)	62.2 (47.4 to 78.3)	29.5% (23.0 to 36.3)	89.4 (66.4 to 112.7)	37.4% (28.7 to 46.2)	56.9 (44.3 to 70.2)	46.3% (36.7 to 55.2)	21.4 (17.0 to 27.3)	52.8% (43.7 to 61.0)
Ambient particulate matter pollution	120.0 (83.4 to 151.4)	17.2% (12.3 to 21.4)	9.6 (7.1 to 12.4)	10.7% (8.2 to 13.8)	37.5 (26.9 to 48.1)	17.8% (13.4 to 22.2)	50.3 (31.7 to 63.5)	21.1% (13.8 to 26.4)	19.0 (12.0 to 26.5)	15.6% (9.8 to 21.1)	3.6 (2.3 to 5.3)	9.3% (5.9 to 13.0)
Household air pollution from solid fuels	62.9 (34.7 to 116.6)	9.0% (5.0 to 16.8)	0.05 (0.00 to 0.5)	0.06% (0.00 to 0.6)	3.2 (0.2 to 17.1)	1.5% (0.09 to 7.9)	17.1 (3.9 to 43.7)	7.2% (1.6 to 18.7)	27.6 (17.6 to 39.7)	22.7% (14.9 to 31.6)	14.9 (11.6 to 19.2)	38.3% (30.7 to 45.4)
Low temperature	34.5 (29.7 to 40.2)	4.9% (4.3 to 5.7)	5.4 (4.5 to 6.3)	6.0% (5.2 to 6.9)	14.9 (12.7 to 17.4)	7.1% (6.1 to 8.1)	10.7 (9.0 to 13.0)	4.5% (4.0 to 5.2)	2.7 (1.7 to 3.9)	2.2% (1.4 to 3.1)	0.8 (0.6 to 1.1)	2.1% (1.5 to 2.8)
High temperature	6.6 (0.7 to 15.4)	0.9% (0.1 to 2.2)	0.4 (−0.2 to 1.1)	0.4% (−0.2 to 1.2)	0.5 (−0.8 to 2.5)	0.3% (−0.4 to 1.2)	2.3 (0.4 to 5.3)	1.0% (0.1 to 2.2)	2.8 (0.7 to 5.5)	2.3% (0.6 to 4.5)	0.7 (0.3 to 1.3)	1.7% (0.7 to 3.2)
Lead exposure	51.8 (−7.1 to 113.4)	7.4% (−1.0 to 16.5)	3.5 (−0.5 to 7.8)	3.9% (−0.5 to 8.9)	12.5 (−1.7 to 28.1)	5.9% (−0.8 to 13.2)	19.3 (−2.7 to 42.7)	8.1% (−1.1 to 18.2)	12.3 (−1.7 to 26.6)	10.1% (−1.4 to 22.4)	4.2 (−0.5 to 9.3)	10.7% (−1.5 to 23.5)
**Dietary risks**	132.2 (39.7 to 223.1)	18.8% (5.8 to 31.8)	13.5 (2.8 to 24.3)	15.1% (3.1 to 27.5)	40.2 (11.7 to 68.3)	19.1% (5.5 to 32.7)	47.6 (15.6 to 79.8)	19.9% (6.3 to 33.0)	22.5 (6.5 to 38.0)	18.2% (5.2 to 30.3)	8.2 (2.6 to 14.0)	20.2% (6.3 to 32.3)
Diet high in sodium	72.5 (17.9 to 160.1)	10.4% (2.6 to 22.5)	6.9 (1.1 to 17.4)	7.7% (1.2 to 19.2)	25.7 (7.3 to 53.3)	12.2% (3.6 to 24.6)	29.9 (8.3 to 62.4)	12.6% (3.6 to 25.4)	7.9 (0.8 to 21.5)	6.6% (0.7 to 17.5)	1.9 (0.07 to 6.0)	5.0% (0.2 to 15.0)
Diet high in red meat	1.2 (−1.3 to 11.6)	0.2% (−0.2 to 1.7)	0.7 (−0.4 to 2.3)	0.7% (−0.4 to 2.5)	1.0 (−0.4 to 4.9)	0.5% (−0.2 to 2.4)	0.4 (−0.8 to 4.1)	0.2% (−0.3 to 1.7)	−0.6 (−1.7 to 0.3)	−0.5% (−1.4 to 0.3)	−0.3 (−0.8 to 0.1)	−0.7% (−1.9 to 0.4)
Diet low in fruits	20.6 (10.9 to 32.7)	3.0% (1.6 to 4.6)	1.3 (0.4 to 2.2)	1.4% (0.5 to 2.4)	3.4 (1.6 to 5.4)	1.6% (0.8 to 2.6)	6.5 (3.5 to 10.1)	2.7% (1.5 to 4.2)	7.1 (4.0 to 11.4)	5.9% (3.3 to 9.0)	2.4 (1.3 to 3.8)	6.1% (3.5 to 8.9)
Diet high in processed meat	4.4 (1.0 to 7.8)	0.6% (0.2 to 1.1)	1.6 (0.4 to 2.8)	1.8% (0.4 to 3.0)	1.7 (0.4 to 3.1)	0.8% (0.2 to 1.4)	0.6 (0.1 to 1.0)	0.2% (0.06 to 0.4)	0.4 (0.09 to 0.7)	0.3% (0.07 to 0.5)	0.1 (0.03 to 0.2)	0.3% (0.07 to 0.5)
Diet low in vegetables	11.9 (6.4 to 18.3)	1.7% (0.9 to 2.6)	0.7 (0.1 to 1.3)	0.8% (0.1 to 1.5)	1.0 (0.4 to 1.7)	0.5% (0.2 to 0.8)	3.2 (1.7 to 4.8)	1.3% (0.7 to 2.0)	4.1 (2.3 to 6.4)	3.4% (1.9 to 5.0)	2.8 (1.7 to 4.3)	7.3% (4.4 to 10.1)
Diet low in whole grains	31.3 (−31.7 to 87.8)	4.5% (−4.6 to 12.3)	3.1 (−3.0 to 9.3)	3.4% (−3.4 to 10.3)	10.0 (−10.0 to 27.8)	4.7% (−4.8 to 13.6)	10.4 (−10.6 to 27.8)	4.4% (−4.5 to 11.8)	5.5 (−5.7 to 15.2)	4.5% (−4.7 to 11.9)	2.3 (−2.3 to 6.2)	5.8% (−6.3 to 14.7)
High alcohol use	38.7 (−6.0 to 96.2)	5.6% (−0.9 to 13.6)	7.7 (−1.1 to 18.4)	8.6% (−1.2 to 21.1)	14.3 (−2.1 to 35.8)	6.8% (−1.0 to 16.8)	12.2 (−2.1 to 30.4)	5.1% (−0.9 to 12.5)	3.2 (−0.5 to 8.5)	2.6% (−0.4 to 6.7)	1.3 (−0.2 to 3.4)	3.3% (−0.6 to 8.4)
Diet high in sugar-sweetened beverages	1.4 (0.7 to 2.3)	0.2% (0.1 to 0.3)	0.4 (0.2 to 0.7)	0.5% (0.2 to 0.8)	0.5 (0.2 to 0.8)	0.2% (0.1 to 0.4)	0.4 (0.2 to 0.6)	0.1% (0.08 to 0.2)	0.1 (0.06 to 0.2)	0.1% (0.06 to 0.2)	0.02 (0.01 to 0.03)	0.05% (0.03 to 0.08)
Diet low in fiber	9.5 (−0.4 to 19.2)	1.4% (−0.1 to 2.8)	0.9 (−0.1 to 2.0)	1.0% (−0.1 to 2.3)	1.7 (−0.1 to 3.7)	0.8% (−0.03 to 1.8)	3.7 (−0.2 to 7.6)	1.5% (−0.1 to 3.1)	2.6 (−0.1 to 5.3)	2.1% (−0.1 to 4.2)	0.6 (−0.03 to 1.3)	1.5% (−0.1 to 3.1)
Diet low in polyunsaturated fatty acids	0.2 (0.1 to 0.3)	0.03% (0.01 to 0.05)	0.01 (0.00 to 0.03)	0.02% (0.00 to 0.03)	0.05 (0.01 to 0.09)	0.02% (0.01 to 0.04)	0.06 (0.02 to 0.1)	0.03% (0.01 to 0.05)	0.04 (0.01 to 0.07)	0.03% (0.01 to 0.05)	0.01 (0.00 to 0.03)	0.03% (0.01 to 0.06)
**Physical activity**												
Low physical activity	33.6 (9.1 to 63.0)	4.8% (1.3 to 9.0)	3.7 (−0.3 to 8.2)	4.1% (−0.3 to 8.8)	9.4 (1.6 to 18.9)	4.5% (0.8 to 9.0)	12.4 (4.1 to 22.8)	5.2% (1.6 to 9.5)	6.5 (2.4 to 11.3)	5.3% (2.0 to 8.9)	1.6 (0.6 to 2.8)	4.1% (1.6 to 6.8)
**Tobacco smoking**	109.2 (87.6 to 134.0)	15.5% (12.7 to 18.4)	10.7 (8.5 to 13.3)	11.9% (9.6 to 14.2)	36.3 (29.1 to 45.1)	17.2% (14.1 to 20.4)	41.6 (32.6 to 51.6)	17.4% (14.1 to 20.6)	16.9 (13.1 to 21.5)	13.8% (11.2 to 16.6)	3.6 (2.7 to 4.7)	8.8% (6.9 to 10.8)
Smoking	85.1 (70.4 to 102.8)	12.2% (10.3 to 14.3)	9.0 (7.3 to 11.1)	10.0% (8.3 to 11.9)	28.9 (23.7 to 35.2)	13.8% (11.6 to 16.1)	32.2 (25.6 to 39.1)	13.5% (11.2 to 16.0)	12.5 (10.0 to 15.7)	10.3% (8.5 to 12.2)	2.5 (2.0 to 3.3)	6.5% (5.4 to 7.8)
Secondhand smoke	27.4 (18.2 to 36.8)	3.9% (2.7 to 5.2)	1.9 (1.3 to 2.7)	2.2% (1.5 to 2.9)	8.6 (5.7 to 11.6)	4.1% (2.8 to 5.5)	10.8 (7.1 to 14.5)	4.5% (3.1 to 6.0)	5.0 (3.3 to 6.8)	4.1% (2.9 to 5.5)	1.1 (0.7 to 1.6)	2.9% (2.0 to 3.9)
**Physiological factors**	550.0 (468.0 to 627.8)	78.2% (67.3 to 86.4)	69.0 (57.1 to 80.3)	76.9% (66.0 to 85.5)	167.2 (141.6 to 190.4)	79.4% (68.3 to 87.8)	187.3 (156.5 to 216.5)	78.4% (67.5 to 86.8)	96.3 (81.1 to 112.6)	78.3% (68.0 to 86.3)	29.7 (24.1 to 37.5)	73.1% (62.2 to 81.3)
High body-mass index	44.4 (6.5 to 86.5)	6.4% (1.0 to 12.0)	6.6 (1.0 to 12.9)	7.4% (1.1 to 14.0)	15.4 (2.2 to 30.3)	7.3% (1.1 to 14.1)	13.5 (2.0 to 26.2)	5.7% (0.8 to 10.8)	7.1 (1.1 to 13.6)	5.9% (0.9 to 11.0)	1.8 (0.2 to 3.3)	4.5% (0.6 to 8.4)
High fasting plasma glucose	123.7 (95.9 to 153.8)	17.7% (14.0 to 21.8)	18.4 (14.2 to 22.6)	20.5% (16.5 to 24.9)	36.3 (28.3 to 45.4)	17.3% (13.7 to 21.3)	40.7 (31.4 to 51.2)	17.1% (13.5 to 21.2)	22.3 (16.6 to 28.1)	18.3% (14.5 to 22.6)	5.9 (4.4 to 7.8)	15.2% (12.1 to 18.9)
High systolic blood pressure	410.6 (311.5 to 502.0)	58.8% (44.6 to 70.0)	48.5 (35.3 to 60.3)	54.2% (40.2 to 65.4)	126.5 (94.7 to 155.6)	60.2% (45.5 to 71.5)	140.3 (104.5 to 175.6)	59.0% (44.3 to 70.7)	72.8 (55.6 to 89.7)	60.0% (45.2 to 71.1)	22.1 (16.3 to 28.5)	56.8% (42.5 to 67.8)
High LDL cholesterol	209.8 (74.1 to 346.6)	30.0% (10.4 to 48.6)	26.6 (9.0 to 43.8)	29.8% (9.8 to 49.3)	66.1 (23.2 to 108.4)	31.5% (10.8 to 50.7)	71.5 (25.4 to 117.8)	30.1% (10.5 to 48.4)	34.6 (12.4 to 57.3)	28.5% (10.1 to 46.2)	10.7 (3.8 to 18.0)	27.6% (9.9 to 45.0)
Kidney dysfunction	69.6 (49.4 to 91.6)	10.0% (7.1 to 12.9)	8.4 (5.4 to 11.6)	9.4% (6.2 to 12.6)	18.9 (13.1 to 25.3)	9.0% (6.3 to 11.9)	24.2 (17.0 to 31.7)	10.2% (7.3 to 13.1)	13.9 (9.9 to 18.2)	11.4% (8.4 to 14.6)	4.2 (2.9 to 5.8)	10.7% (7.9 to 13.6)
**Combined risk factors**												
All factors	622.5 (557.3 to 686.7)	89.2% (82.8 to 93.6)	76.4 (66.1 to 86.4)	85.4% (77.3 to 91.4)	186.8 (166.1 to 208.5)	88.9% (82.0 to 93.6)	213.1 (189.0 to 238.3)	89.6% (83.7 to 94.0)	110.4 (97.8 to 126.8)	90.9% (85.5 to 94.5)	35.3 (30.0 to 43.6)	90.6% (85.0 to 94.1)

Data in parentheses are 95% uncertainty intervals. Count data in 100,000 are presented to one decimal places and percentage data are presented to one decimal place. Percentages and number of DALYs are not mutually exclusive: the sum of percentages and number of DALYs in the columns exceeds the totals for all risk factors combined because of overlap between various risk factors. The crude sum of population attributable fraction (PAF) of the risk factors might exceed 100% because the effects of many of these risk factors are mediated partly or wholly through another risk factor or risk factors.

Among the 21 GBD regions, the most significant increase in the number of DALYs attributable to risk factors for IS was observed in East Asia, with a 133% increase from 9.2 million in 1990 to 22.5 million in 2021. Conversely, the most significant decrease from 5.2 million in 1990 to 2.8 million in 2021 was observed in Western Europe (Supplementary Table 9; Supplementary Figure 12). Among the 204 countries and territories, China presented the most substantial increase in risk factor-attributable DALYs (135%), from 8.9 million in 1990 to 21.0 million in 2021. Moreover, the Russian Federation demonstrated the most prominent decrease of 21%, from 4.6 million in 1990 to 3.6 million in 2021. The most significant increase in the age-standardized PAF for IS was observed in Palau, at 7.2%, with an increase from 82.8% in 1990 to 88.8% in 2021. The most significant decrease in the age-standardized PAF for IS was observed in Italy, at 9.8%, with a decrease from 90.9% in 1990 to 82.0% in 2021 (Supplementary Table 10).

Globally, from 1990 to 2021, the risk factor associated with the greatest increase in the age-standardized PAF for IS was a high fasting plasma glucose level, which increased by 29.2%, from 13.7 to 17.7%. Other risk factors, including exposure to ambient particulate matter pollution and a high body mass index (BMI), increased by 30.3% (from 13.2 to 17.2%) and 36.2% (from 4.7 to 6.4%), respectively. Conversely, the PAF for exposure to household air pollution from solid fuels decreased significantly by 50%, from 18.0 to 9.0%. The PAF for smoking-related IS decreased from 14.0 to 12.2% (Table 2; Supplementary Table 8).

**Figure 4:**
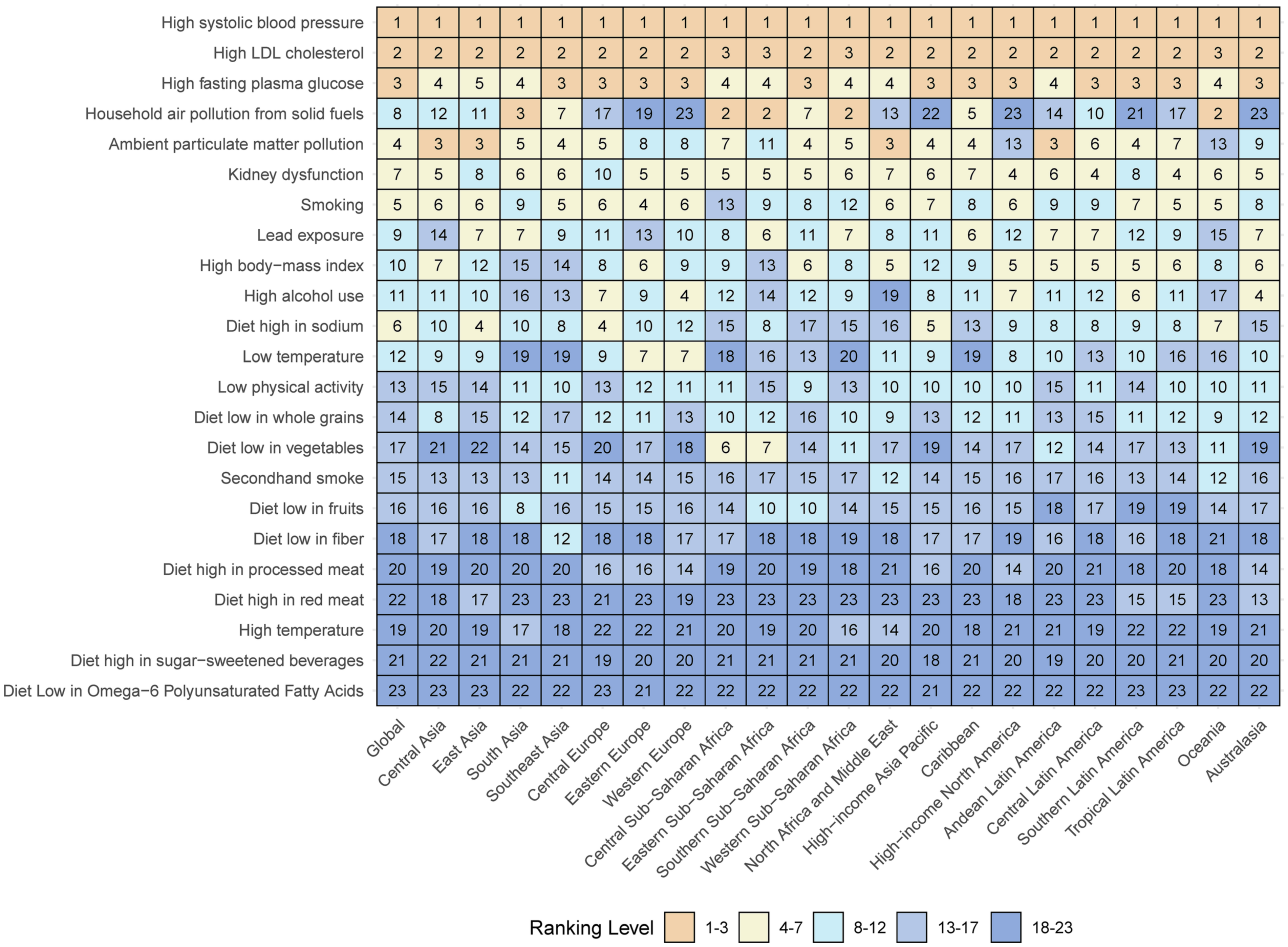
Age-standardized ischaemic stroke related DALYs attributable to risk factors by 21 GBD regions, for both sexes, 2021. Numbers show the ranking level (1 = highest, 23 = lowest) by the number of DALYs attributable to the corresponding risk factors. Orange shows 1st–3rd ranking; yellow, 4–7 ranking; cyan, 8–12 ranking; light blue, 13–17 ranking; and dark blue, 18–23 ranking. DALY = disability-adjusted life-year. GBD = Global Burden of Diseases, Injuries, and Risk Factors Study.

In 2021, the number of DALYs attributable to risk factors differed across region, country and SDI subgroups (Supplementary Tables 9–11). The proportion of DALYs attributable to risk factors varied more significantly among different regions, with Western Sub-Saharan Africa having the highest proportion of DALYs attributable to risk factors, reaching 91.2%, whereas Australasia had the lowest proportion, at 83.1% (Supplementary Tables 6, 9). In 2021, risk factors associated with age-standardized DALYs for IS differed among the 21 GBD regions (Figure 4).

In 2021, the leading specific risk factors contributing to DALYs were high systolic blood pressure (4.1 million attributable DALYs; 58.8% of all IS DALYs), high LDL cholesterol (21.0 million; 30.0%), high fast plasma glucose (12.4 million; 17.7%), ambient particle matter pollution (12.0 million; 17.2%) and smoking (8.5 million; 12.2%; Table 2; Supplementary Figure 12). When sex differences were considered, high systolic blood pressure and high LDL cholesterol remained the primary contributors to both sexes. However, the impact of smoking was more pronounced in males (20.3% vs. 3.1%) (Supplementary Table 12; Supplementary Figure 13). From 1990 to 2021, the two primary risk factors that contributed most to the IS burden continued to be high systolic blood pressure and high LDL cholesterol (Supplementary Figure 14).

### 3.8 Health inequality analyses

**Figure 5:**
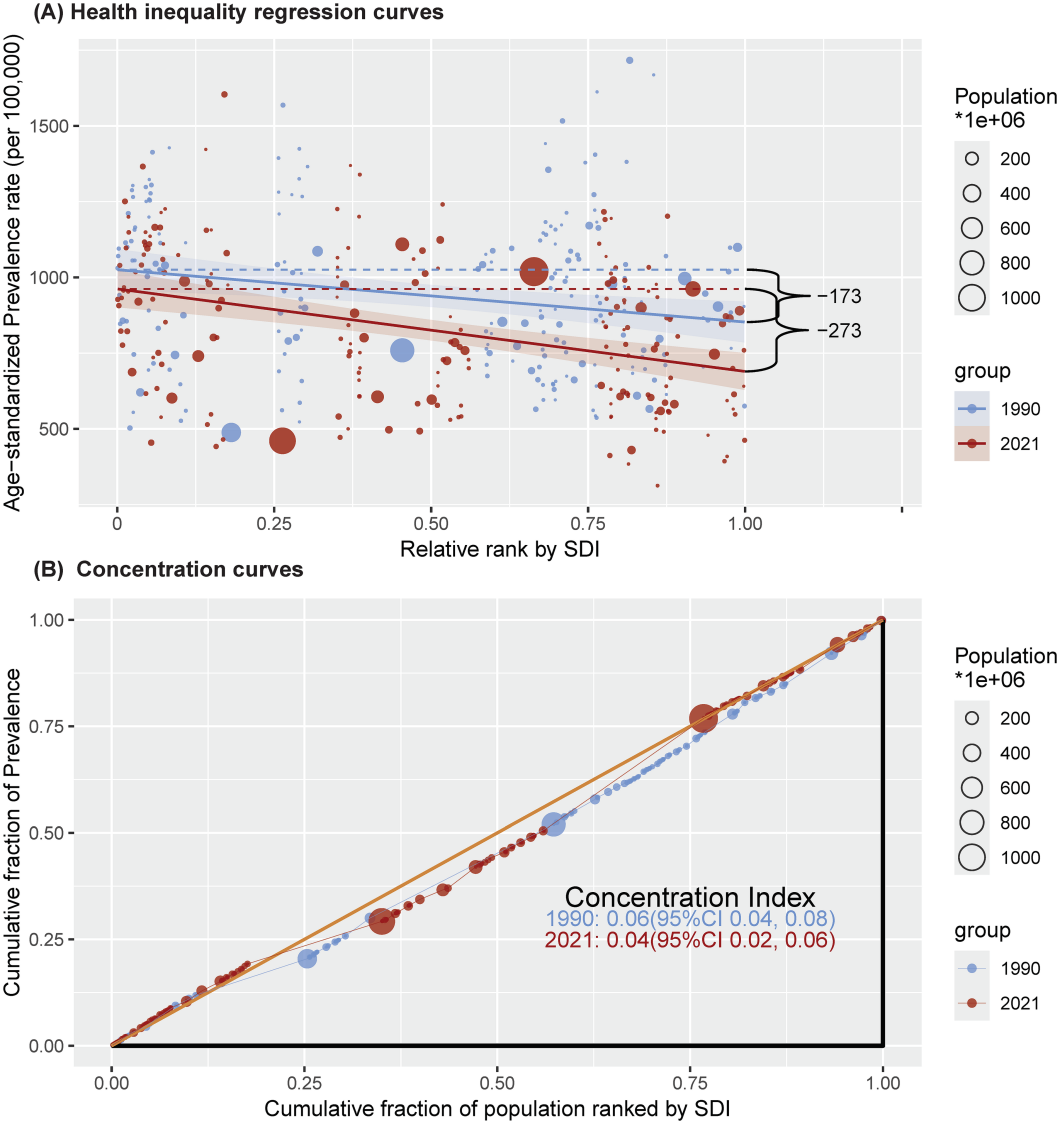
Health inequality regression curves **(A)** and concentration curves **(B)** for the prevalence of ischaemic stroke among people aged ≥20 years from 1990 to 2021 across the world. SDI = sociodemographic index.

The slope index of inequality showed a pronounced gap in the age-standardized prevalence, mortality, and DALY rates for IS between countries with the highest and lowest SDI values, decreasing from −172.7, −3.9 and −234.8 in 1990 to −272.6, −40.8 and −757.0 in 2021, respectively, indicating that countries with lower SDIs experienced disproportionately higher burdens. In contrast, the relative gradient inequality, as measured by the concentration index, was 0.06, 0.04 and −0.02 in 1990 and 0.04, −0.06 and −0.06 in 2021, respectively, indicating that the burden was fairly distributed between the poor and rich populations (Figure 5; Supplementary Figures 15, 16; Supplementary Table 13).

### 3.9 Global trends of IS predicted by the BAPC model

**Figure 6:**
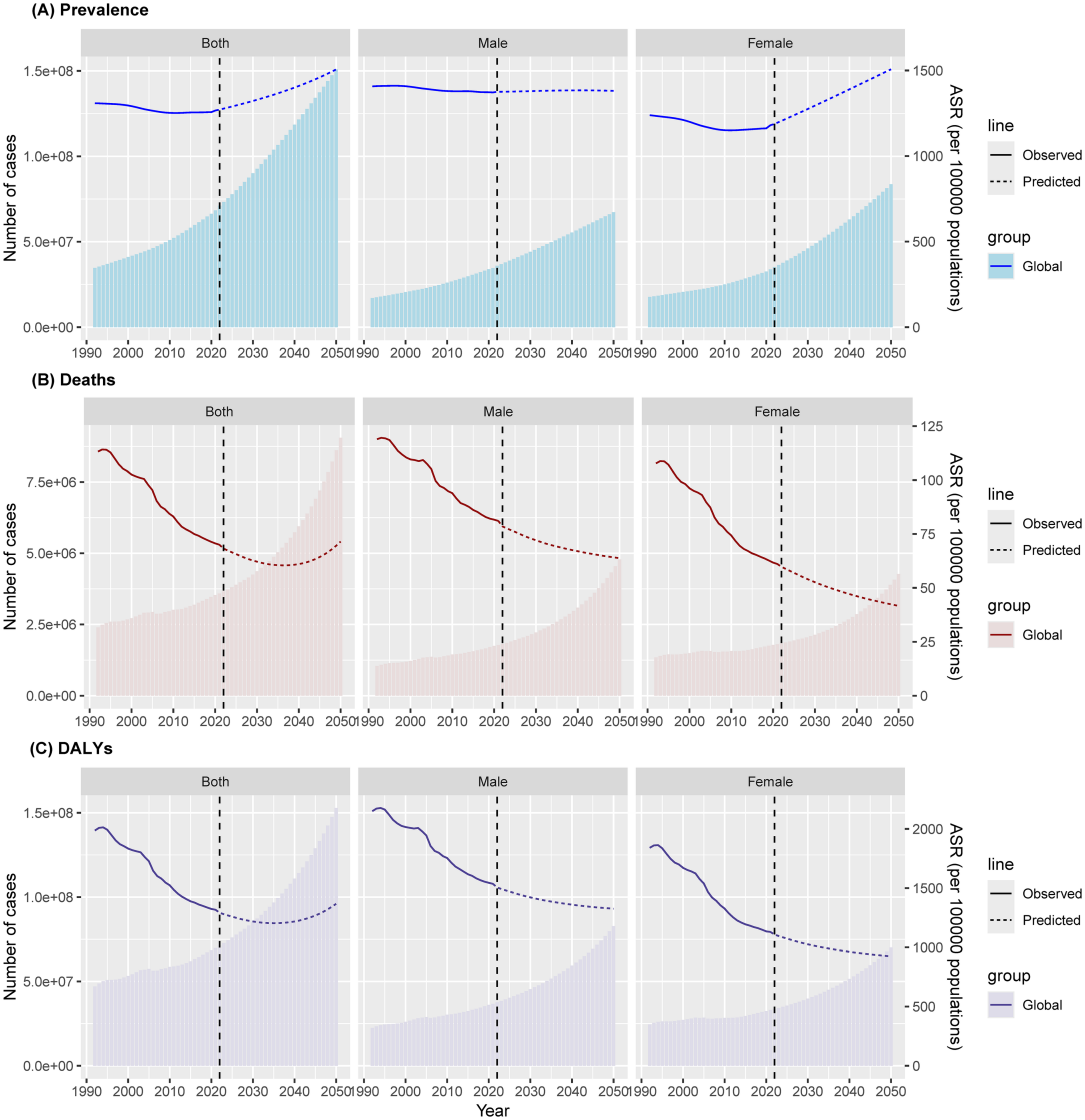
Age-standardized ischaemic stroke among people aged ≥20 years prevalence **(A)**, mortality **(B)** and DALYs **(C)** over time at global, with projections up to 2050. DALY = disability-adjusted life-year; ASR = age-standardized rate.

The BAPC model projected that the age-standardized prevalence of IS among this population will increase significantly worldwide, from approximately 1266.1 cases per 100,000 population in 2021 to approximately 1506.2 cases per 100,000 population in 2050. This represents an increase of 19.0% over three decades. Globally, the age-standardized mortality rate is projected to increase to approximately 71.5 cases per 100,000 population, resulting in an estimated 9.1 million deaths by 2050. Similarly, the age-standardized DALY rate will increase to 1,366.7 cases per 100,000 population, with 152.9 million cases of DALYs projected in 2050 (Figure 6; Supplementary Tables 14–16). Notably, sex differences persist, with men having a greater burden of disease than women in all three indicators. However, the difference in incidence between males and females appears to be decreasing, a trend that has become apparent over time.

## 4 Discussion

This study presented standardized, comprehensive and updated estimates of the burden of IS and the burden attributable to risk factors among adults aged 20 years and older in 204 countries and territories from 1990 to 2021. Globally, between 1990 and 2021, the number of individuals living with IS in this age group increased from 33.2 to 68.4 million. Over the same period, the global age-standardized prevalence of IS decreased from 1,309 cases per 100,000 population to 1,266 cases per 100,000 population. However, there was a dramatic increase in the subgroup older than 80 years in the middle-SDI subgroup and in East Asia, whereas Southeast Asia experienced stability. The global age-standardized mortality rate for IS decreased from 116 cases per 100,000 population to 70 cases per 100,000 population, with a consistent downward trend across all age, sex and SDI subgroups, except for an increase in southern sub-Saharan Africa. Compared with low-SDI countries, high-SDI countries presented a sevenfold faster decrease in the age-standardized mortality rate. Additionally, there was a 35% reduction in the number of global age-standardized DALYs, whereas an increase was observed in southern sub-Saharan Africa. Furthermore, males consistently faced a greater burden of IS across all subgroups. At the global level, individuals with high systolic blood pressure and high LDL-C levels consistently remained at the highest risk for DALYs from 1990 to 2021. Projections for 2050 indicate a significant increase in the age-standardized prevalence of IS among adults worldwide, with a predicted narrowing of the sex difference.

Despite a global decrease in the age-standardized estimated burden of IS, reflecting ongoing efforts in primary prevention worldwide, the overall number of IS cases continues to increase (17). This insufficient decrease underscores the need for nuanced policy and preventive measures across different subgroups. Notably, an increase in the age-standardized prevalence was observed in middle-SDI countries, particularly in East Asia, whereas this rate remained stable in Southeast Asia. However, it is important to highlight that within Southeast Asia, Vietnam exhibits a distinct trend. According to a recent study by Thien Tan Tri Tai Truyen et al., stroke was responsible for 166,954 deaths in Vietnam in 2021, with an age-standardized incidence rate of 203.36 per 100,000 people, exceeding the Southeast Asian average of 187.98 and the global average of 141.55. This indicates a rising burden of stroke in Vietnam, suggesting that while the overall trend in Southeast Asia may be stable, specific countries within the region may still face increasing challenges (18). In contrast, high-SDI regions, especially Tropical Latin America, southern Latin America and high-income Asia Pacific, exhibited the most significant decreases. Additionally, the number of age-standardized DALYs increased in southern sub-Saharan Africa, in contrast with the substantial reductions in western Europe, high-income Asia Pacific, and Australasia. Factors such as hypertension and diabetes were most prevalent in black individuals and Asian individuals (19). The observed disparities in the IS burden across SDI groups may be driven by systemic differences in healthcare infrastructure and access. In high-SDI regions, advanced stroke care networks (e.g., thrombectomy-capable centres) and robust primary prevention programs (e.g., population-wide hypertension screening) are likely contributors to declining mortality rates (10). Conversely, in low-SDI regions such as southern sub-Saharan Africa, limited access to acute interventions (e.g., thrombolysis availability <10%) and fragmented poststroke rehabilitation services exacerbate disability rates (20). Notably, middle-SDI countries such as China exhibit mixed trends: similar to high-SDI nations, urban hubs show decreased mortality, whereas rural areas lag due to uneven resource distributions (21). In addition, most prevention guidelines and treatment recommendations originate from high-income countries, considering comorbidities, resource implications, and practical implementation challenges (22), which are associated with differences in socioeconomic status, health awareness, health resource allocation, policies and disease prevention measures across different SDIs (23, 24). This disparity highlights the need for more proactive measures in low-, low-middle- and middle-SDI regions, where efforts to address IS risk and primary prevention strategies are especially critical.

Males consistently face a greater burden of IS across all subgroups, although the sex gap is projected to narrow between 2021 and 2050. This result was also supported by an increased absolute number of DALYs due to stroke in males (10). The relationship between sex and IS risk is poorly understood. Our analysis further revealed that high blood pressure and high LDL-C levels were the two top risk factors for both males and females, whereas a greater percentage of males had smoke exposure. If smoke exposure is reduced to its TMREL, the burden of IS could be reduced by 20.3% in males and only by 3.1% in females. Previous studies have indicated that behavioral risk factors are more prominent in males, whereas metabolic risk factors are more prevalent in females (18). For example, smoking, a significant behavioral risk factor, is more common among males, contributing to a greater stroke burden in this group. Conversely, metabolic risk factors such as high cholesterol and diabetes may have a more substantial impact on females. This disparity in risk factor prevalence between sexes underscores the need for targeted interventions. By addressing the specific risk factors prevalent in each sex, more effective prevention strategies can be developed to reduce the stroke burden in both males and females.

Globally, from 1990 to 2021, high systolic blood pressure and high LDL-C levels remained the top two risk factors for IS burden in most subgroups, accounting for 58.8 and 30.0% of the global number of DALYs, respectively, emphasizing the ongoing challenge of controlling blood pressure and LDL-C. A recent observational study involving nearly one million participants revealed that even stage 1 hypertension increased the risk of IS in adults under 65 years of age, underscoring the importance of maintaining blood pressure below 140/90 mmHg (25). However, most studies have focused on intensive blood pressure control or increased individual-based blood pressure control during the acute phase of IS or secondary prevention (26–28). More intensive or personalized blood pressure targets for primary prevention, especially in hypertension or high-risk patients, could inform new community prevention guidelines. High LDL-C levels are the most common cause of mortality in high-middle-SDI countries (29). A high HDL-C/LDL-C ratio of 0.4–0.6 was associated with a lower risk of IS, suggesting the need for proactive management of serum LDL-C levels in high-risk adults. Further research on LDL-C control prior to the first IS event is needed to support health policies. The other top risk factors among this age group included high fasting plasma glucose levels, exposure to household air pollution from solid fuels, exposure to ambient particulate matter pollution, kidney dysfunction, smoking habits, lead exposure, high BMI and high alcohol consumption. Reducing the risk of IS involves comprehensive and systematic control of all risk factors (21). A comprehensive approach to managing these risk factors is essential for reducing the risk of IS, with a focus on primary and secondary prevention strategies, including lifestyle modifications, risk factor treatments, antiplatelet therapy, anticoagulation for atrial fibrillation, and air pollution control (17).

Finally, there was a notable decrease in the age-standardized mortality and DALY s from 2019 to 2021, potentially related to the COVID-19 pandemic. The pandemic impacted life expectancy and shifted trends for many leading causes of death (9, 11). The establishment of green channels for stroke care, which facilitate swift medical intervention, might be another important measure contributing to the reduction in IS-related mortality and DALYs (30).

These findings suggest that addressing the dual challenges of population ageing and an increasing number of IS cases requires tailored policies across different global subgroups. More active exploration and rigorous control of major risk factors might provide promise for significantly reducing the burden of IS globally.

There are several limitations in this study. First, our study has general limitations common to all GBD studies: the GBD database is a compilation of data from multiple sources, which could vary in completeness and accuracy across different regions. This might affect the reliability of our findings. However, the GBD standardized its method to minimize biases and included a wide range of data sources to ensure comprehensive coverage. Second, our calculations did not account for the impact of the COVID-19 pandemic on the IS burden because of data limitations. Future updates will incorporate pandemic data to assess the effects of the COVID-19 pandemic on the trend of IS burden. Third, GBD data are usually published in specific years and may not reflect the latest health trends. We used the latest available GBD 2021 data but acknowledge potential lags in capturing recent developments. Fourth, while our study provides comprehensive estimates of overall IS burden, the GBD 2021 dataset does not stratify IS into aetiological subtypes (e.g., embolic vs. thrombotic). This granularity is critical, as embolic strokes (often linked to atrial fibrillation) require different prevention strategies (e.g., anticoagulation) than thrombotic events do, as these events are driven by atherosclerosis. Future iterations of the GBD study should prioritize subtype-specific data collection to enable targeted policy design.

In conclusion, our study underscores the necessity of SDI-specific strategies to combat the increasing global burden of ischaemic stroke. High-SDI regions should focus on developing metabolic risk control interventions, such as implementing AI-driven primary care platforms for LDL-C monitoring, and establish geriatric stroke units to address ageing-related IS incidence. Middle−/low-SDI regions would benefit from scaling up low-cost interventions such as community hypertension screening kits and strengthening referral networks for acute stroke care. In sub-Saharan Africa, investment in neuroimaging infrastructure is crucial to improve the accuracy of IS diagnosis and reduce its misclassification as haemorrhagic stroke.

Future research should focus on subtype-specific burden through prospective cohorts that integrate imaging and biomarker data to better understand embolic vs. thrombotic IS trends. Additionally, longitudinal studies are needed to assess the legacy effects of COVID-19, particularly how pandemic-induced disruptions in hypertension management impact post-2021 IS outcomes. Finally, modeling studies should evaluate the cost-effectiveness of various interventions for IS prevention across different SDI strata, such as comparing polypills to telehealth programs. These efforts will provide essential evidence to guide policymakers in developing targeted and efficient strategies to reduce the global burden of ischaemic stroke.

## Data Availability

The original contributions presented in the study are included in the article/Supplementary material, further inquiries can be directed to the corresponding author.

## Footnotes

^1^ https://vizhub.healthdata.org/gbd-results/

^2^ https://ghdx.healthdata.org/gbd-2021/sources
