# Supplementary material for: Global, regional, and national trends in ischaemic stroke burden and risk factors among adults aged 20 + years (1990–2021): a systematic analysis of data from the Global Burden of Disease study 2021 with projections into 2050
Source: Front Public Health. 2025 Apr 25;13:1567275. doi: 10.3389/fpubh.2025.1567275 (PMC12061693; doi:10.3389/fpubh.2025.1567275)
Supplement: Supplementary file 1 [file Data_Sheet_1.pdf]

Supplementary Material

This appendix formed part of the original submission.

Supplement to: Global, regional, and national trends in ischaemic stroke burden and risk factors among adults aged 20+ years (1990-2021): A systematic analysis of data from the Global Burden of Disease Study 2021 with projections into 2050

Content

Supplementary Methods 5
1. Overview 5
2. Data sources 5
3. Calculation methods for DALYs 6
3.1 YLLs 6
3.2 YLDs 7
4. Estimation of ischemic stroke non-fatal burdens in GBD 2021 10
4.1 Case definition 10
4.2 Data seeking and flowchart 11
5. Estimation of ischemic stroke fatal burdens in GBD 2021 11
6. Estimation of ischemic stroke burdens attributable to risk factor in GBD 2021 11
6.1 Definitions of risk factors in GBD 2021 12
7. Estimation of ischemic stroke cross-country health inequality in GBD 2021 16
7.1 Slope index of inequality 17
7.2 Concentration index 17
8. Predictive analysis of ischemic stroke 18
9. Spatiotemporal Gaussian process regression (ST-GPR) modelling 19
10. Data accuracy 20
10.1 Data extractions in each country 20
11. Socio-demographic Index (SDI) 21
11.1 SDI definition 21
11.2 SDI calculation 21
11.3 SDI grouping and quantification 22
12. Reference 59
Additional Results in Tables and Figures 61
eTable 1. Age standardised mortality of ischemic stroke in people aged ⩾20 years and their AAPC at global and regional level, 1990-2021 61
eTable 2. Age standardised DALYs of ischemic stroke in people aged ⩾20 years and their AAPC at global and regional level, 1990-2021 63
eTable 3. Age standardized prevalence of ischemic stroke in people aged ≥20 years and their AAPCs at regional levels, 1990-2021 65
eTable 4. Age standardized prevalence, mortality and DALYs of ischemic stroke in people aged ⩾20 years at regional levels by sex, 2021 67
eTable 5. Age standardized mortality of ischemic stroke in people aged ⩾20 years and their AAPCs at regional levels, 1990-2021 69
eTable 6. Age standardized DALYs of ischemic stroke in people aged ⩾20 years and their AAPCs at regional levels, 1990-2021 71
eTable 7. Age standardized prevalence, mortality and DALYs of ischemic stroke in people aged ⩾20 years in 2021 and their AAPCs between 1990-2021 in 204 countries and territories 73
eTable 8. Risk factors for ischemic stroke related DALYs among people aged ⩾20 years in

1990..81
eTable 9. Ischemic stroke related DALYs (absolute numbers [in 100000 with 95% UI] and percentage [with 95% UI]) associated with risk factors and their clusters in 1990 and 2021, by SDI, aged ⩾20 years, both sexes...84
eTable 10. Ischemic stroke related DALYs (absolute numbers [in millions with 95% UI] and percentage [with 95% UI]) associated with risk factors and their clusters in 1990 and 2021, by countries and territories, aged ⩾20 years, both sexes 87
eTable 11a. Percent of DALYs (with 95% uncertainty intervals) due to IS for environmental risks by country and territory for both sexes combined in 2021, aged ⩾20 years 95
eTable 11b. Percent of DALYs (with 95% uncertainty intervals) due to IS for dietary risks by country and territory for both sexes combined in 2021, aged ⩾20 years............................104
eTable 11c. Percent of DALYs (with 95% uncertainty intervals) due to IS for metabolic risks by country and territory for both sexes combined in 2021, aged ⩾20 years.......................116
eTable 11d. Percent of DALYs (with 95% uncertainty intervals) due to IS for tobacco and behavioral risks by country and territory for both sexes combined in 2021, aged ⩾20 years 125
eTable 12. Risk factors for ischemic stroke related DALYs among people aged ⩾20 years at global in 2021, by sex 134
eTable 13. Sociodemographic-index-related inequalities in prevalence, deaths, disease-adjusted life years (DALYs) for ischemic stroke, aged ⩾20 years, both sexes...................136
eTable 14. Case number and ASR prediction of prevalence for ischemic stroke aged ⩾20 years from 2022 to 2050 by BAPC models in global, by both sexes..................................143
eTable 15. Case number and ASR prediction of deaths for ischemic stroke aged ⩾20 years from 2022 to 2050 by BAPC models in global, by both sexes 148
eTable 16. Case number and ASR prediction of DALYs for ischemic stroke aged ⩾20 years from 2022 to 2050 by BAPC models in global, by both sexes 153
eFigure 1. The changes in the proportion of prevalence cases among ischemic stroke patients (IS) aged over 20 years to the overall IS patients from 1990 to 2021.................................158
eFigure 2. Temporal trend of age-standardized prevalence (A) mortality (B) and disability-adjusted life years (C) for IS patients aged over 20 years and overall IS patients from 1990 to 2021..159
eFigure 3. The age-standardized mortality (A) and disability-adjusted life years (B) proportion of IS to all-cause in patients aged over 20 years from 1990 to 2021..................160
eFigure 4. Temporal trend of age-standardized mortality and disability-adjusted life years of IS in patients aged over 20 years from 1990 to 2021 at global and socio-demographic index levels by sex...161
eFigure 5. Average annual percent changes of age-standardized mortality and disability-adjusted life years of IS in patients aged over 20 years from 1990 to 2021 at socio-demographic index levels by sex. 162
eFigure 6. Average annual percent changes of age-standardized prevalence, mortality and disability-adjusted life years of IS in patients aged over 20 years from 1990 to 2021 by sex and age. 163
eFigure 7. Temporal trend of age-standardized prevalence, mortality and disability-adjusted life years of IS aged over 20 years and overall IS patients from 1990 to 2021 at global and

socio-demographic index levels. 164
eFigure 8. Average annual percent changes of age-standardized prevalence (A), mortality (B) and disability-adjusted life years (C) of IS aged over 20 years and overall IS patients from 1990 to 2021 at global and socio-demographic index levels. 165
eFigure 9. Prevalence (A), mortality (B) and disability-adjusted life years (C) rate of IS patients aged over 20 years from 204 countries according to the socio-demographic index in 2021....167
eFigure 10. Average annual percent changes of age-standardized prevalence, mortality and disability-adjusted life years of IS patients aged over 20 years from 1990 to 2021 at regions levels. 168
eFigure 11. Average annual percent changes of age-standardized mortality and disability-adjusted life years of IS patients aged over 20 years from 1990 to 2021 at regions levels by sex. 169
eFigure 12. Proportion of DALYs attributable to risk factors of IS patients aged over 20 years for both sexes, 2021 170
eFigure 13. Global ischemic stroke related DALYs attributable to risk factors in 1990 and 2021 (rank based on total number of IS patients aged over 20 years DALYs), by sex 171
eFigure 14. Global ischemic stroke related DALYs attributable to risk factors in 1990 and 2021 (rank based on total number of IS patients aged over 20 years DALYs) 172
eFigure 15. Health inequality regression curves and concentration curves for ischemic stroke aged over 20 years in terms of deaths 174
eFigure 16. Health inequality regression curves and concentration curves for ischemic stroke aged over 20 years in terms of disease-adjusted life years (DALYs)....175
STROBE Statement....176

Supplementary Methods

1. Overview

For more than three decades, the Global Burden of Diseases, Injuries, and Risk Factors Study (GBD) has been systematically and comprehensively recording and analysing causes of human death stratified by age, sex, and time across the world[1]. This information has been used to guide policy solutions, reduce modifiable risk factors, monitor and evaluate national and sub-national health interventions, and ultimately improve health recommendations at both regional and local levels. Published in The Lancet in May 2020, GBD 2021 provides, for the first time, an independent estimation of population for each of 204 countries and territories and for the globe using a standardized, replicable approach, as well as a comprehensive update on fertility and migration[2]. GBD 2021 incorporates major data additions and improvements and methodological refinements. Mortality and life expectancy estimates have expanded to a total of 881 locations at the most detailed level, and new causes have been added to the fatal and nonfatal cause lists, for a total of 371 diseases and injuries (https://www.healthdata.org/research-analysis/about-gbd/protocol). GBD 2021 produced estimates for each epidemiological quantity of interest— incidence, prevalence, mortality, years lived with disability (YLDs), years of life lost (YLLs), and disability-adjusted life-years (DALYs)— for 288 causes of death by age-sex-location-year for 25 age groups from birth to 95 years and older; for males, females, and both sexes combined; in 204 countries and territories grouped into 21 regions and seven super-regions; and for every year from 1990 to 2021. GBD 2021 also includes subnational analyses for 21 countries and territories. An international network of collaborators provides, reviews, and analyses the available data to generate these metrics; GBD 2021 drew on the expertise of more than 11 000 collaborators from more than 160 countries and territories. GBD classifies diseases and injuries into a hierarchy with four levels that include both fatal and non-fatal causes. Level 1 causes include three broad aggregate categories (communicable, maternal, neonatal, and nutritional [CMNN] diseases; non-communicable diseases [NCDs]; and injuries) and Level 2 disaggregates those categories into 22 clusters of causes, which are further disaggregated into Level 3 and Level 4 causes. At the most detailed level, 288 fatal causes are estimated[2].

2. Data sources

GBD 2021 synthesises a large and growing number of data input sources including surveys, censuses, vital statistics, and other health-related data sources. The data from these sources are used to estimate morbidity; illness, and injury; and attributable risk for 204 countries and territories from 1990 to 2021; mortality deaths are estimated from 1980 to 2021.

The GBD estimation process is based on identifying multiple relevant data sources for each disease or injury, including censuses, household surveys, civil registration and vital statistics, disease registries, health service use, air pollution monitors, satellite imaging, disease notifications, and other sources. Each of these types of data is identified from a systematic review of published studies, searches of government and international organization websites, published reports, primary data sources such as the Demographic and Health Surveys, and contributions of datasets by GBD collaborators. All data

used in this study were extracted from the Global Health Data Exchange (https://vizhub.healthdata.org/gbd-results/) including (1) global age- and sex- specific prevalence, mortality, DALYs numbers and crude rates (per 100,000 persons) from 1990 to 2021; (2) Regional age- and sex- specific incidence, mortality, DALYs numbers and crude rates from 1990 to 2021 by socio-demographic index (SDI) categories; (3) National age- and sex- specific incidence, mortality, DALYs numbers and crude rates from 1990 to 2021; (4) GBD world standard population in 2021; (5) Age- and sex- specific ischemic stroke DALYs numbers and crude rates attributable to risk factors (level 4).

3. Calculation methods for DALYs

To estimate DALYs, GBD 2021 started by estimating cause-specific mortality and non- fatal health loss. For each year for which YLDs have been estimated, GBD 2021 computed DALYs by adding YLLs and YLDs for each age-sex-location (Figure 1). Uncertainty in YLLs was assumed to be independent of uncertainty in YLDs. A total of 500 draws for DALYs were computed by initially summing the first instance of the 500 draws for YLLs and YLDs, followed by repeating this process for each subsequent draw. 95% UIs were computed by using the 25th and 975th ordered draw of the DALY uncertainty distribution. DALYs were determined as the aggregate of YLLs and YLDs for every cause, stratified by location, age group, sex, and year. For more information, please refer to the previously reported literature[3].

Figure 1. DALY burden estimation for GBD 2021

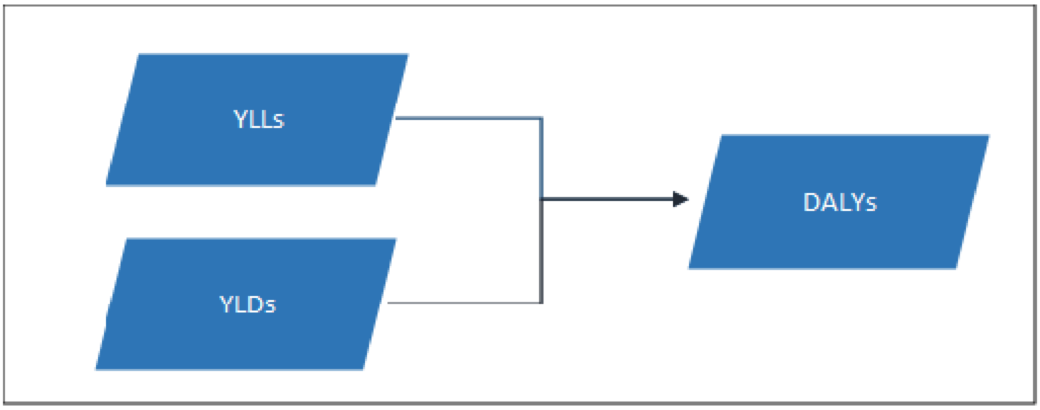

3.1 YLLs

Years of life lost (YLLs) owing to premature mortality were computed for 1082 locations and 39 years. The initial step involved employing the lowest observed age-specific mortality rates by location and sex across all estimation years. These rates were extracted from locations with total populations exceeding 5 million in 2016 to construct a theoretical minimum risk reference life table.

The YLL is a metric that is computed by multiplying the number of estimated deaths by the predicted life expectancy by age, sex, location, and year. The metric therefore highlights premature deaths by

applying a larger weight to deaths that occur in younger age groups. Uncertainty was propagated from the CoDCorrected deaths across all demographic groups. The predicted life expectancy is calculated with two main components, a global, sex-agnostic, all time theoretical "best" life expectancy (assuming that males and females in all countries in the world could theoretically have this life expectancy) and the average age of death from with-shock life tables for each location, sex, age group, and year. Further details on GBD life expectancy values can be found in the previous papers[2,4]. The core equation can be written as follows:

$$YLL = \sum_{c=1,a=o,s=1}^{\infty} d_{cas}\, e_a$$

3.2 YLDs

The critical stage in the estimation of YLDs is a micro-simulation, which adjusts for comorbidity. GBD 2021 refer to this micro-simulation process as "COMO" (for comorbidity correction). For GBD 2021, the co-occurrence of different diseases was estimated by simulating 20,000 individuals in each location-age-sex-year combination, with each individual exposed to the independent probability of having any of the sequelae included in GBD, based on prevalence. Age was the main predictor of comorbidity such that age- specific micro-simulations accommodated most of the required comorbidity correction[5].

The two components necessary for the computation of YLDs and are the two inputs into COMO: 1) prevalence of each disease sequela and 2) DWs. The prevalence values of causes are primarily produced by using DisMod-MR 2.1 and, for causes with multiple sequelae, subsequently apportioned into sequela-specific prevalence based on available estimates of the severity distribution.

The micro-simulation, as performed for each age-sex-location-year, can best be represented as a four-step process. First, simulated individuals (simulants) are exposed to independent probabilities of having each sequela, where the probability is equal to the prevalence estimate. For each simulant, the probability of having a disease sequela is equal to the estimated prevalence. Each simulant is determined to have or not have the disease sequelae based on a draw from a binomial distribution. From this simulation, simulants end up with any number of sequelae, from 0 up to the theoretical maximum given their demographics. Second, the DW for each simulant is estimated on the basis of the disease sequelae that they have acquired. The formula for the cumulative DW for a simulant is one minus the multiplicative sum of one minus each DW present

$$Simulant\ DW_l = 1 - \prod_{k=i}^{j} (1 - \mathrm{DWk})$$

Where:

DWk is the DW for the kth disease sequela that the simulant l has acquired.

Once the simulant DW is computed, the DW attributable to each sequela for the simulant is calculated by using the following formula:

$$ADW_{lk} = \frac{DW_k}{\sum_{k=i}^{k=j} DW_k} * Simulant\ DW_l$$

Where:

$ADWlk$ is the attributable DW for disease sequela k in simulant l

DWk is the DW for disease sequela k

Simulant DW_l is the DW for simulant l from the combination of all sequelae that they have acquired.

This formula apportions the overall simulant DW to each condition in proportion to the DW of each condition in isolation.

Finally, YLDs per capita in an age-sex-country-year are computed by taking the sum of the attributable DWs for a disease sequela across simulants.

$$YLD\ Ratek = \frac{\sum_{l=1}^{n} ADW_{lk}}{n}$$

The actual number of YLDs from disease sequela k in an age-sex-location-year is then computed as the YLD rate k times the appropriate age-sex-location-year population.

GBD 2021 determined the disability weights for each sequela from the GBD disability weight survey. The table below illustrates the severity levels, lay descriptions, and associated disability weights applicable for outcomes related to acute stroke (Table 1).

Table 1. Severity distribution, details on the severity levels for acute stroke in GBD 2021 and the associated disability weight (DW) with that severity

Severity level	Lay description	Modified Rankin score	Cognitive status	DW (95% Cl)
Stroke, mild	Has some difficulty in moving around and some weakness in one hand, but is able to walk without help.	1	N/A	0.019 (0.010-0.032)
Stroke, moderate, with no heart failure	Has some difficulty in moving around, and in using the hands for lifting and holding things, dressing, and grooming.	2,3	MoCA >26 or MMSE >24	0.070 (0.046-0.099)
Stroke, moderate, with controlled, medically managed heart failure	Has some difficulty in moving around, and in using the hands for lifting and holding things, dressing, and grooming. Has been diagnosed with clinical heart failure, a chronic disease that requires medication every day and causes some worry but minimal	2,3	MoCA >26 or MMSE >24	0.116 (0.076-0.164)

	interference with daily activities.			
Stroke, moderate, with mild heart failure	Has some difficulty in moving around, and in using the hands for lifting and holding things, dressing, and grooming. Is short of breath and easily tires with moderate physical activity, such as walking uphill or more than a quarter-mile on level ground. The person feels comfortable at rest or during activities requiring less effort.	2,3	MoCA >26 or MMSE >24	0.109 (0.074-0.154)
Stroke, moderate, with moderate heart failure	Has some difficulty in moving around, and in using the hands for lifting and holding things, dressing, and grooming. Is short of breath and easily tires with minimal physical activity, such as walking only a short distance. The person feels comfortable at rest but avoids moderate activity.	2,3	MoCA >26 or MMSE >24	0.137 (0.091-0.191)
Stroke, moderate, with severe heart failure	Has some difficulty in moving around, and in using the hands for lifting and holding things, dressing, and grooming. Is short of breath and feels tired when at rest. The person avoids any physical activity, for fear of worsening the breathing problems.	2,3	MoCA >26 or MMSE >24	0.236 (0.165-0.319)
Stroke, moderate plus cognition problems, with no heart failure	Has some difficulty in moving around, in using the hands for lifting and holding things, dressing and grooming, and in speaking. The person is often forgetful and confused.	2,3	MoCA <26 or MMSE <24	0.316 (0.206-0.437)
Stroke, moderate plus cognition problems, with controlled, medically managed heart failure	Has some difficulty in moving around, in using the hands for lifting and holding things, dressing and grooming, and in speaking. The person is often forgetful and confused. Has been diagnosed with clinical heart failure, a chronic disease that requires medication every day and causes some worry but minimal interference with daily activities.	2,3	MoCA <26 or MMSE <24	0.349 (0.241-0.470)
Stroke, moderate plus cognition problems, with mild heart failure	Has some difficulty in moving around, in using the hands for lifting and holding things, dressing and grooming, and in speaking. The person is often forgetful and confused. Is short of breath and easily tires with moderate physical activity, such as walking uphill or more than a quarter-mile on level ground. The person feels comfortable at rest or during activities requiring less effort.	2,3	MoCA <26 or MMSE <24	0.344 (0.237-0.464)
Stroke, moderate plus cognition problems, with moderate heart failure	Has some difficulty in moving around, in using the hands for lifting and holding things, dressing and grooming, and in speaking. The person is often forgetful and confused. Is short of breath and easily tires with minimal physical activity, such as walking only a short distance. The person feels comfortable at rest but avoids moderate activity.	2,3	MoCA <26 or MMSE <24	0.365 (0.253-0.487)
Stroke, moderate plus cognition problems, with severe heart failure	Has some difficulty in moving around, in using the hands for lifting and holding things, dressing and grooming, and in speaking. The person is often forgetful and confused. Is short of breath and feels tired when at rest. The person avoids any physical activity, for fear of worsening the breathing problems.	2,3	MoCA <26 or MMSE <24	0.437 (0.308-0.575)
Stroke, severe, with no heart failure	Is confined to bed or a wheelchair, has difficulty speaking, and depends on others for feeding, toileting, and dressing.	4,5	MoCA >26 or MMSE >24	0.552 (0.377-0.707)
Stroke, severe, with controlled, medically managed heart failure	Is confined to bed or a wheelchair, has difficulty speaking, and depends on others for feeding, toileting, and dressing. Has been diagnosed with clinical heart failure, a chronic disease that requires medication every day and causes some worry but minimal interference with daily activities.	4,5	MoCA >26 or MMSE >24	0.573 (0.408-0.720)
Stroke, severe, with mild heart failure	Is confined to bed or a wheelchair, has difficulty speaking, and depends on others for feeding, toileting, and dressing. Is short of breath and easily tires with moderate physical activity, such as walking uphill or more than a quarter-mile on level ground. The person feels comfortable at rest or during activities requiring less effort.	4,5	MoCA >26 or MMSE >24	0.570 (0.404-0.720)
Stroke, severe, with moderate heart failure	Is confined to bed or a wheelchair, has difficulty speaking, and depends on others for feeding, toileting, and dressing. Is short of breath and easily tires with minimal physical activity, such as walking only a short distance. The person feels comfortable at rest but avoids moderate activity.	4,5	MoCA >26 or MMSE >24	0.584 (0.417-0.732)
Stroke, severe, with severe heart failure	Is confined to bed or a wheelchair, has difficulty speaking, and depends on others for feeding, toileting, and dressing. Is short of	4,5	MoCA >26 or MMSE >24	0.630 (0.458-

	breath and feels tired when at rest. The person avoids any physical activity, for fear of worsening the breathing problems.			0.777)
Stroke, severe plus cognition problems, no heart failure	Is confined to bed or a wheelchair, depends on others for feeding, toileting, and dressing, and has difficulty speaking, thinking clearly, and remembering things.	4,5	MoCA <26 or MMSE <24	0.588 (0.411-0.744)
Stroke, severe plus cognition problems, controlled, medically managed heart failure	Is confined to bed or a wheelchair, depends on others for feeding, toileting, and dressing, and has difficulty speaking, thinking clearly, and remembering things. Has been diagnosed with clinical heart failure, a chronic disease that requires medication every day and causes some worry but minimal interference with daily activities.	4,5	MoCA <26 or MMSE <24	0.608 (0.438-0.759)
Stroke, severe plus cognition problems, mild heart failure	Is confined to bed or a wheelchair, depends on others for feeding, toileting, and dressing, and has difficulty speaking, thinking clearly, and remembering things. Is short of breath and easily tires with moderate physical activity, such as walking uphill or more than a quarter-mile on level ground. The person feels comfortable at rest or during activities requiring less effort.	4,5	MoCA <26 or MMSE <24	0.604 (0.436-0.758)
Stroke, severe plus cognition problems, moderate heart failure	Is confined to bed or a wheelchair, depends on others for feeding, toileting, and dressing, and has difficulty speaking, thinking clearly, and remembering things. Is short of breath and easily tires with minimal physical activity, such as walking only a short distance. The person feels comfortable at rest but avoids moderate activity.	4,5	MoCA <26 or MMSE <24	0.617 (0.448-0.768)
Stroke, severe plus cognition problems, severe heart failure	Is confined to bed or a wheelchair, depends on others for feeding, toileting, and dressing, and has difficulty speaking, thinking clearly, and remembering things. Is short of breath and feels tired when at rest. The person avoids any physical activity, for fear of worsening the breathing problems.	4,5	MoCA <26 or MMSE <24	0.659 (0.488-0.808)

4. Estimation of ischemic stroke non-fatal burdens in GBD 2021

4.1 Case definition

Stroke was defined according to WHO criteria as rapidly developing clinical signs of focal (or less commonly global) disturbance of cerebral function lasting more than 24 hours or leading to death with no apparent cause other than that of vascular origin[6]. Cases of transient ischemic attack (TIA) were not included.

Acute stroke: Stroke cases are considered acute from the day of incidence of a first-ever stroke through day 28 following the event.

Chronic stroke: Stroke cases are considered chronic beginning 28 days following the occurrence of an event. Chronic stroke includes the late sequelae of an acute stroke and all recurrent stroke events. GBD 2015 adopted this broader definition of chronic stroke than what was used in prior iterations to model acute strokes using only first-ever incident events.

Ischemic stroke: Ischemic strokes are characterised by occlusion of blood flow to part of the brain due to hypoperfusion, most commonly due to a thrombus or embolism. It is defined as an episode of neurological dysfunction caused by focal cerebral, spinal, or retinal infarction.

The reference definitions for ischemic stroke was first-ever, subtype-specific stroke, which included subjects who did not survive to hospital admission. For this subtype, inclusion occurred, after adjustment, of sources employing the following alternate definitions: 1) sources which included first and recurrent strokes; 2) sources which reported only estimates for all subtypes combined; and 3) sources which included only stroke cases which survived to hospital admission.

Table 2: ICD codes used for inclusion of hospital and claims data

Stroke subtype	ICD-9	ICD-10
Ischemic stroke	433-435.9, 437.0-437.1, 437.5-437.8	G45-G46.8, I63-I63.9, I65-I66.9, I67.2-I67.3, I67.5-I67.6, I69.3

4.2 Data seeking and flowchart

Details on the GBD 2021 criteria, literature search and data extraction methods can be found in the other papers[2, 3].

5. Estimation of ischemic stroke fatal burdens in GBD 2021

Details on the estimation of ischemic stroke fatal burdens in GBD 2021 can be found in the other papers[2, 3].

6. Estimation of ischemic stroke burdens attributable to risk factor in GBD 2021

Four key components are included in the estimation of the burden attributable to a given risk factor: the metric of burden being assessed (the number of deaths, YLLs, YLDs, or DALYs [the sum of YLLs and YLDs]); the exposure levels for a risk factor; the RR of a given outcome due to exposure; and the counterfactual level of risk factor exposure. Uncertainty intervals for attributable burden were calculated using 500 draws for GBD 2021[8,9]. Estimates of attributable burden as DALYs for risk–outcome pairs were generated by using the following model:

$$AB_{jasgt} = \sum_{o=1}^{w} DALY_{joasgt}\, PAF_{joasgt}$$

Where:

$ABjasgt$ is the attributable burden for risk factor j for age group a, sex s, location g, and year t;

$DALYjoasgt$ is total DALYs for cause o (of w relevant outcomes for risk factor j) for age group a, sex s, location g, and year t;

$PAFjoasgt$ is the PAF for cause o due to risk factor j for age group a, sex s, location g, and year t.

The proportions of deaths, YLLs, or YLDs attributable to a given risk factor or risk factor cluster were analogously computed by sequentially substituting each metric in place of DALYs in the equation provided.

6.1 Definitions of risk factors in GBD 2021

In GBD 2021, the risk factors associated with ischemic stroke are defined as follows[9].

✓ High fasting plasma glucose:

High fasting plasma glucose (FPG) is measured as the mean FPG in a population, where FPG is a continuous exposure in units of mmol/L. Since FPG is along a continuum, GBD 2021 define high FPG as any level above the theoretical minimum-risk exposure level (TMREL), which is 4.9–5.3 mmol/L.

✓ Non-optimal temperature

The non-optimal temperature risk factor is characterized by same-day exposure to ambient temperatures that deviate from the optimal level for minimal mortality risk, either being warmer or colder. The theoretical minimum risk exposure level (TMREL) for non-optimal temperature refers to the temperature at which the overall mortality related to this risk is at its lowest within a specific location and year. Acknowledging the diversity in exposure–response curves across different mean annual temperature zones and the variability in cause of death composition over space and time, the estimation of TMRELs is conducted on a year-by-year and location-by-location basis, rather than applying a single global TMREL. Exposure to high temperatures (heat) is categorized as conditions where temperatures exceed the TMREL, while exposure to low temperatures (cold) is identified as temperatures that fall below the TMREL.

✓ Ambient particulate matter pollution

Exposure to ambient particulate matter pollution is defined as the population-weighted annual average mass concentration of particles with an aerodynamic diameter less than 2.5 micrometers ($PM_{2.5}$) in a cubic meter of air. This measurement is reported in $\mu g/m^3$.

✓ High alcohol use

High alcohol use is defined as alcohol consumption in excess of the theoretical minimum risk exposure level (TMREL), the level of alcohol consumption at which all-cause risk is minimised. Prior to GBD

2021, this risk factor was simply "Alcohol use" and quantified the burden of alcohol consumption over the entire exposure range.

GBD 2021 defined exposure as the grams per day of pure alcohol consumed among current drinkers, and constructed this exposure using the indicators outlined below:

1). Current drinkers, defined as the proportion of individuals who have consumed at least one alcoholic beverage (or some approximation) in a 12-month period.

2). Alcohol consumption (in grams per day), defined as grams of alcohol consumed by current drinkers, per day, over a 12-month period.

3). Alcohol litres per capita (LPC) stock, defined in LPC of pure alcohol, over a 12-month period.

✓ High body-mass index

High body-mass index (BMI) for adults (ages 20+) is defined as BMI greater than 20 to 23 kg/m^2. High BMI for children and adolescents (ages 2–19) is defined as being overweight or obese based on International Obesity Task Force standards.

✓ High LDL cholesterol

In earlier iterations of the GBD study, including GBD 2010, 2015, and 2016, the burden attributable to total cholesterol (TC) was estimated. Starting with GBD 2017, the modelling of blood concentration of low-density lipoprotein cholesterol (LDL) was initiated, presented in units of mmol/L. Data on blood levels for LDL, TC, triglyceride (TGL), and high-density lipoprotein cholesterol (HDL) were utilized from literature sources as well as from household survey microdata and reports.

✓ High systolic blood pressure

Brachial systolic blood pressure (SBP) in mmHg.

✓ Household air pollution

Exposure to household air pollution from solid fuels (HAP) is estimated from both the proportion of individuals using solid cooking fuels and the level of exposure to particulate matter less than 2.5 micrometres in diameter ($PM_{2.5}$) air pollution for these individuals. Solid fuels in our analysis include wood, coal/charcoal, dung, and agricultural residues.

✓ Kidney dysfunction

The kidney dysfunction (KD) risk factor exposure is divided into four categories of renal function defined by urinary albumin to creatinine ratio (ACR) and estimated glomerular filtration rate (eGFR). The definitions of KD exposures can be found in Table 6.

Table 3: Case Definitions of KD Exposure

Exposure	Parameter	Status	Definition
Stages 1&2 chronic kidney disease (CKD)	Prevalence	Reference	ACR of >=30 mg/g and estimated glomerular filtration rate (eGFR) > 60 mL/min/1.73m^2 as estimated using the CKD-EPI equation for individuals age >18 and the Schwartz equation for those <18.
Stage 3 chronic kidney disease	Prevalence	Reference	Estimated glomerular filtration rate (eGFR) 30-60 mL/min/1.73m^2 as estimated using the CKD-EPI equation for individuals age >18 and the Schwartz equation for those <18 not on renal replacement therapy.
Stage 3 chronic kidney disease	Prevalence	Alternative	Estimated glomerular filtration rate (eGFR) 30-60 mL/min/1.73m^2 as estimated using the MDRD equation (or modifications thereof) for individuals age >18 not on renal replacement therapy.
Stage 3 chronic kidney disease	Prevalence	Alternative	Estimated glomerular filtration rate (eGFR) 30-60 mL/min/1.73m2 as estimated using the Cockcroft-Gault equation (standardised for body surface area) for individuals age >18 not on renal replacement therapy.
Stage 4 chronic kidney disease	Prevalence	Reference	Estimated glomerular filtration rate (eGFR) 15-30 mL/min/1.73m^2 as estimated using the CKD-EPI equation for individuals age >18 and the Schwartz equation for those <18 not on renal replacement therapy.
Stage 4 chronic kidney disease	Prevalence	Alternative	Estimated glomerular filtration rate (eGFR) 15-30 mL/min/1.73m2 as estimated using the MDRD equation (or modifications thereof) for individuals age >18 not on renal replacement therapy.
Stage 4 chronic kidney disease	Prevalence	Alternative	Estimated glomerular filtration rate (eGFR) 15-30 mL/min/1.73m2 as estimated using the Cockcroft-Gault equation (standardised for body surface area) for individuals age >18 not on renal replacement therapy.
Stage 5 chronic kidney disease	Prevalence	Reference	Estimated glomerular filtration rate (eGFR) <15 mL/min/1.73m2 as estimated using the CKD-EPI equation for individuals age >18 and the Schwartz equation for those <18 not on renal replacement therapy.
Stage 5 chronic kidney disease	Prevalence	Alternative	Estimated glomerular filtration rate (eGFR) <15 mL/min/1.73m2 as estimated using the MDRD equation (or modifications thereof) for individuals age >18 not on renal replacement therapy.
Stage 5 chronic kidney disease	Prevalence	Alternative	Estimated glomerular filtration rate (eGFR) <15 mL/min/1.73m2 as estimated using the Cockcroft-Gault equation (standardised for body surface area) for individuals age >18 not on renal replacement therapy.

The modelling of renal function prevalence estimates is described in detail in the CKD section of the appendix to the GBD 2021 disease and injury paper.

✓ Lead exposure

Exposure to lead is defined in two different ways according to the currently known pathways of attributable health loss. Acute lead exposure, measured as micrograms of lead per decilitre of blood (µg/dL), is associated with IQ loss in children. Chronic lead exposure, measured as micrograms of lead per gram of bone (µg/g), is associated with cardiovascular diseases, mediated by the impact of lead on increased systolic blood pressure.

✓ Low physical activity

Low physical activity is defined as objectively measured, total physical activity less than 3600 to 4400 MET-minutes per week. Assessment of physical activity in adults over the age of 25 involves evaluating activities performed for a minimum of ten minutes at a stretch, across various life domains, including leisure, work, household chores, and transportation. The total metabolic equivalent (MET)-minutes per week are calculated based on the frequency, duration, and intensity of these activities.

MET is the ratio of the working metabolic rate to the resting metabolic rate. One MET is equivalent to 1 kcal/kg/hour and is equal to the energy cost of sitting quietly. A MET is also defined as the oxygen uptake in ml/kg/min with one MET equal to the oxygen cost of sitting quietly, around 3.5 ml/kg/min.

✓ Secondhand smoke

GBD 2021 define secondhand smoke exposure as current exposure to secondhand tobacco smoke at home or at work. Household composition serves as a proxy for estimating household secondhand smoke exposure, with the assumption that all individuals residing with a daily smoker are subjected to tobacco smoke exposure. Surveys are utilized to gauge the proportion of the population exposed to secondhand smoke in the workplace environment. For the purposes of these estimates, only non-smokers are considered to be potentially exposed to secondhand smoke. Non-smokers are defined as all persons who are not daily smokers. Ex-smokers and occasional smokers are considered non-smokers in this analysis. Exposure is evaluated for both children and adults.

✓ Smoking

Consistent with prior GBD cycles, the prevalence of current smoking and the prevalence of former smoking was estimated using data sourced from cross-sectional, nationally representative household surveys. Current smokers were identified as individuals partaking in the use of any smoked tobacco product on either a daily or occasional basis. Former smokers were categorized as those who had ceased the use of all smoked tobacco products for a minimum duration of six months, or in accordance with the definition employed by the respective survey, where possible.

✓ Dietary risk factors

The definitions of Dietary risk factor exposure can be found in Table 7.

Table 4: Dietary risk factor exposure definitions and optimal level of exposure as defined by GBD 2021

Dietary Risk Factor Definition of exposure	Dietary Risk Factor Definition of exposure	Optimal level or range of intake*
Diet low in fruit	Average daily consumption (in grams per day) of fruit including fresh, frozen, cooked, canned, or dried fruit, excluding fruit juices and salted or pickled fruits	340–350 g/day
Diet low in vegetables	Average daily consumption (in grams per day) of vegetables, including fresh, frozen, cooked, canned, or dried vegetables and excluding legumes and salted or pickled vegetables, juices, nuts and seeds, and starchy vegetables such as potatoes or corn	306-372 g/day
Diet low in whole grains	Average daily consumption (in grams per day) of whole grains (bran, germ, and endosperm in their natural proportion) from breakfast cereals, bread, rice, pasta, biscuits, muffins, tortillas, pancakes, and other sources	160-210 g/day
Diet low in nuts and seeds	Average daily consumption (in grams per day) of nuts and seeds, including tree nuts and seeds and peanuts	19-24 g/day
Diet low in fibre	Average daily consumption (in grams per day) of fibre from all sources including fruits, vegetables, grains, legumes, and pulses	22-25 g/day

Diet low in seafood omega-3 fatty acids	Average daily consumption (in milligrams per day) of eicosapentaenoic acid (EPA) and docosahexaenoic acid (DHA)	470-660 mg/day
Diet low in omega- 6 polyunsaturated fatty acids	Average daily consumption (in % daily energy) from omega-6 polyunsaturated fatty acids (PUFA) (specifically linoleic acid, Y- linolenic acid, eicosadienoic acid, dihomo-Y- linolenic acid, arachidonic acid)	9-10% of total daily energy
Diet low in calcium	Average daily consumption (in grams per day) of calcium from all sources, including milk, yoghurt, and cheese	0.72-0.86 g/day (males)<br>1.1-1.2 g/day (females)
Diet low in milk	Average daily consumption (in grams per day) of dairy milk including non-fat, low-fat, and full-fat milk, but excluding plant-based milks, fermented milk products such as buttermilk, and other dairy products such as cheese	280-340 g/day (males)<br>500-610 g/day (females)
Diet low in legumes	Average daily consumption (in grams per day) of legumes and pulses, including fresh, frozen, cooked, canned, or dried legumes	100-110 g/day
Diet high in red meat	Average daily consumption (in grams per day) of unprocessed red meat including pork and bovine meats such as beef, pork, lamb, and goat, but excluding all processed meats, poultry, fish, and eggs	0-200 g/day
Diet high in processed meat	Average daily consumption (in grams per day) of meat preserved by smoking, curing, salting, or addition of chemical preservatives	0 g/day
Diet high in sugar-sweetened beverages (SSBs)	Average daily consumption (in grams per day) of beverages with >50 kcal per 226.8 gram serving, including carbonated beverages, sodas, energy drinks, and fruit drinks, but excluding 100% fruit and vegetable juices	0 g/day
Diet high in trans fatty acids	Average daily consumption (in percent daily energy) of trans fat from all sources, mainly partially hydrogenated vegetable oils and ruminant products	0-1.1% of total daily energy
Diet high in sodium	Average 24-hour urinary sodium excretion (in grams per day)	1-5 g/day

* “Optimal level” is equivalent to “TMREL” throughout this document.

7. Estimation of ischemic stroke cross-country health inequality in GBD 2021

Health inequities are the unjust differences in health between persons of different social groups, and can be linked to forms of disadvantage such as poverty, discrimination and lack of access to services or goods. While health inequity is a normative concept, and thus cannot be precisely measured or monitored, health inequality –observable differences between subgroups within a population – can be measured and monitored, and serves as an indirect means of evaluating health inequity. For detailed information on this topic, please consult the section on health inequality on the official website of the World Health Organization[10].

The two most common complex measures to summarize health inequality in a series of subgroups with a natural ordering are the slope index of inequality (to assess absolute inequality) and the concentration index (to assess relative inequality). A common strength of both of these measures is that their calculation involves weighting by the size of the population, enabling them to yield a single number that describes inequality among all subgroups, taking into account the population size. The detailed calculation principles for these two indicators can be found in the Healthy Inequality Monitoring handbook on the official website of the World Health Organization[11].

7.1 Slope index of inequality

The slope index of inequality is used to show the gradient of health across multiple subgroups with natural ordering (most commonly education or wealth). The slope index of inequality represents the absolute difference in predicted values of a health indicator between those with the highest level of education or wealth and those with the lowest level of education or wealth, while taking into consideration the entire distribution of education or wealth using an appropriate regression model. To calculate the slope index of inequality, a weighted sample of the whole population is ranked from the most disadvantaged subgroup (at rank zero or 0) to the most advantaged (at rank 1) according to, for example, education or wealth. This ranking is weighted, accounting for the proportional distribution of the population within each subgroup. The population of each wealth or education category is then considered in terms of its range in the cumulative population distribution, and the midpoint of this range. Then, the health indicator of interest is regressed against this midpoint value for wealth or education subgroups using an appropriate model, and the predicted values of the health indicator are calculated for the two extremes (rank 1 and rank 0). The difference between the predicted values at rank 1 and rank 0 (covering the entire distribution) generates the slope index of inequality value. Thus, the slope index of inequality represents the difference between the lowest and the highest, while considering all other subgroups in the regression (that is, the effect of change in the whole distribution of population by education or wealth). When the slope of the regression line is flat, the slope index of inequality is 0. When ranking from the most disadvantaged to the most advantaged, positive values indicate that the health indicator of interest is more prevalent in the most advantaged subgroup, whereas negative values mean that the indicator is more prevalent in the most disadvantaged subgroup.

The slope index of inequality value has straightforward meaning and has the same unit of measure as the health indicator, making it very useful. Non-technical audiences can understand the slope index as an estimate of the difference in a given health indicator between the worst-off and best-off individual in a population, though they may initially understand little about how the number is calculated or why it provides an advantage over simple difference.

7.2 Concentration index

The concentration index is a relative measure of inequality that shows the health gradient across multiple subgroups with natural ordering (most commonly education or wealth). It indicates the extent to which a health indicator is concentrated among the disadvantaged or the advantaged. Given that a population is ranked by increasing socioeconomic status, the concentration index has a negative value when the health indicator – whether a favourable indicator such as measles immunization coverage or an adverse indicator such as under-five mortality – is concentrated among the disadvantaged (for example, the poor or less educated); and it has a positive value when the health indicator is concentrated among the advantaged (for example, the rich or more educated). When there is no inequality, the concentration index is 0. If a single individual (the smallest possible population subgroup) accounted for 100% of a health indicator in a population (the highest relative inequality that is theoretically possible), this would cause the concentration index to approach its maximum absolute value of either –1 or +1. While ±1 is the theoretical maximum of a concentration index, in practice absolute values for the concentration index

will rarely exceed 0.5, and a value of 0.2 to 0.3 is considered to represent a reasonably high level of relative inequality.

The visual way to illustrate the concentration index is to use a related concept called the concentration curve. Like the slope index of inequality, it starts with ranking a weighted sample of the whole population from the most-disadvantaged subgroup (at rank 0) to the most-advantaged (at rank 1) according to, for example, education or wealth (x axis). The y axis indicates the cumulative fraction of the health indicator corresponding to each subgroup. The concentration curve is drawn by connecting the dots. The concentration curve lies below the 45° diagonal line from the bottom left corner to the top right – the so-called line of equality – if the health indicator is concentrated among the advantaged; the concentration curve lies above the line of equality if the health indicator is concentrated among the disadvantaged. When there is no inequality, the concentration curve lies on the line of equality. The concentration index is calculated as twice the area between the hypothetical line of equality and the concentration curve.

The slope index is calculated using the Linear Model (LM) function of R or Robust Linear Model (RLM) function (in the event of suspicion of significant heteroscedasticity in the data), following the reorganization of the data in accordance with the guidelines set forth in the Healthy Inequality Monitoring handbook. The concentration index is calculated using the egen_inequal package of Stata[12,13].

8. Predictive analysis of ischemic stroke

In order to forecast the number and incidence of new cases from 2022 through 2050, we used Bayesian age-period-cohort models (BAPC). The BAPC model is founded upon the Age-Period-Cohort (APC) model. The theoretical foundation of this model is the assertion that the incidence or mortality rate is correlated with age structure and population size. The foundation for this analysis is the generalized linear model (GLM).

The application of BAPC models leverages the integrated nested Laplace approximations (INLA) technique to enable a thorough Bayesian analytical process. These models are capable of producing both age-specific and age-standardized predictive rates. In scenarios where the focus is on forecasting, these models inherently incorporate Poisson noise to enhance the predictive distribution's accuracy[14-17].

The BAPC framework decomposes temporal trends into age, period, and cohort effects, enabling nuanced predictions of future disease burden. The model structure is defined as follows:

$$\log(\lambda_{a,p})=\alpha_a+\beta_p+\gamma_c+\epsilon_{a,p}$$

Where:

$\lambda_{a,p}$ represents the expected IS incidence rate for age group a in period p, α_a denotes age-specific intercepts, β_p captures period effects (temporal trends), γ_c accounts for cohort effects (birth year c=p−a), and $\epsilon_{a,p}$ represents residual noise modeled as a Poisson-distributed random variable.

Age-specific incidence rates from 1990 to 2021 were stratified into 5-year age intervals (20–24 to 95+

years) and aligned with the GBD 2021 reference population. Missing data in sparse regions were imputed using geospatial smoothing via DisMod-MR 2.1.

We assigned weakly informative priors to model parameters:

- Age effects (α_a): Gaussian random walk of order 2 to capture smooth age patterns.
- Period (β_p) and cohort (γ_c) effects: First-order random walks with precision parameters τ_β and τ_γ, respectively, estimated via penalized complexity (PC) priors to avoid overfitting.

The INLA algorithm was implemented using the R-INLA package (version 23.09.09) to efficiently approximate posterior distributions. Model convergence was assessed via trace plots and Geweke diagnostics.

Predictive accuracy was evaluated using 10-fold cross-validation, comparing observed vs. predicted rates for 2010–2021. Uncertainty intervals (95% UI) were derived from 1,000 posterior samples, incorporating both parameter uncertainty and Poisson noise.

9. Spatiotemporal Gaussian process regression (ST-GPR) modelling

The input data were modelled by using ST-GPR to allow for smoothing over age, time, and location in locations that were missing complete datasets.

The flowchart showing the analytic steps can be found elsewhere[18]. The approach is a stochastic modelling technique that is designed to detect signals amidst noisy data. It also serves as a powerful tool for interpolating non-linear trends [19,20]. Unlike classical linear models that assume that the trend underlying data follows a definitive functional form, GPR assumes that the specific trend of interest follows a Gaussian process, which is defined by a mean function $m(\cdot)$ and a covariance function $Cov(\cdot)$. For example, let pc,a,s,t be the prevalence, in normal, log, or logit space, observed in country c, for age group a, and sex s at time t:

$$(p_{c,a,s,t}) = g_{c,a,s}(t) + \epsilon_{c,a,s,t}$$

Where:

$$\epsilon_{c,a,s,t} \sim Normal(0, \sigma_p^2) \ ;$$

$$g_{c,a,s}(t) \sim GP\ (m_{c,a,s}(t), Cov\ (g_{c,a,s}(t)))\ .$$

The derivation of the mean and covariance functions, $mc,a,s(t)$ and $Cov\ (gc,a,s(t))$, along with a more detailed description of the error variance ($\sigma 2$), is described in detail in earlier papers[3].

10. Data accuracy

Ischemic stroke is a common and frequent disease worldwide, but only some countries can provide sufficient data. Tables 8 display source count information for non-fatal ischemic stroke.

This is particularly the case in low-resource regions and countries, resulting in a data gap for first-hand epidemiological studies. GBD 2021 study used the following steps to make the estimates as accurate as possible: (1) compilation of data sources through data identification and extraction; (2) data adjustment; (3) estimation of prevalence and incidence by cause and sequelae by using DisMod-MR 2.1 or alternative modelling strategies for selected cause groups; (4) estimation by impairment; (5) severity distributions; (6) incorporation of disability weights (DWs); (7) adjustment for comorbidity; and (8) the estimation of YLDs by sequelae and causes.

Table 5: Source counts for ischemic stroke models

Measure	Total sources	Countries with data
Prevalence	123	27
Incidence	348	63
Excess mortality rate	150	48
With-condition mortality rate	16	10

10.1 Data extractions in each country

Data pertaining to ischemic stroke from registries and hospital records are assembled globally, encompassing contributions from medical facilities, governmental entities, research surveys, and a spectrum of databases. The Global Burden of Disease (GBD) study compiles this information through a systematic process:

Initially, the GBD Collaborators embark on a comprehensive data collection effort, sourcing health-related data from worldwide hospitals, governmental agencies, research surveys, systematic literature reviews, and various databases. This initial phase is followed by a meticulous data cleaning and organization procedure. Subsequently, the research teams employ the Bayesian meta-regression model, DisMod-MR 2.1, to produce estimates for regions and time periods lacking direct data.

In the final stage, to ensure a thorough compilation of data sources not previously captured in the Global Health Data Exchange (GHDx) or PubMed, the team consults with additional experts in the field. The GBD Network encompasses a diverse array of over 12,000 Collaborators hailing from more than 160 countries, representing a broad spectrum of sectors including research, science, healthcare, policy-making, and international organizations.

The GBD employs the DisMod-MR2.1 model to generate ischemic stroke statistics. It is noteworthy that

the original datasets fed into the model are not restricted by age. The GBD refines these estimates through a series of meticulous steps: data cleansing, standardization, adjustment, model development, and calibration validation.

11. Socio-demographic Index (SDI)

11.1 SDI definition

The Socio-demographic Index (SDI) serves as a comprehensive measure reflecting the underlying societal and economic factors that shape health outcomes across geographic locations. Essentially, it represents the geometric average of three distinct indices ranging from 0 to 1: the total fertility rate (TFR) for individuals under the age of 25 (denoted as TFU25), the average educational attainment for the population aged 15 and above (referred to as EDU15+), and the per capita income that has been subject to a lag distribution (known as LDI). Post-calculation of the SDI for the Global Burden of Disease (GBD) 2021, the derived values were adjusted by scaling them up by a factor of 100 to fit within a range of 0 to 100. This scaling facilitates a more intuitive interpretation and comparison across different regions.

11.2 SDI calculation

For each covariate input, an index score of 0 represents the minimum level of each covariate input past which selected health outcomes can get no worse, and an index score of 1 represents the maximum level of each covariate input past which selected health outcomes cease to improve. As a composite, a location with an SDI of 0 would have a theoretical minimum level of sociodemographic development relevant to these health outcomes, and a location with an SDI of 1 (before multiplying by 100 for reporting) would have a theoretical maximum level of sociodemographic development relevant to these health outcomes.

The index scores that form the basis of the SDI were calculated in the following manner:

$$ICly = \max(\frac{C_{ly} - C_{low}}{C_{high} - C_{low}}, 0.05)$$

Where:

> *ICly* is the index for covariate *C*, location *l*, and year *y* and is equal to the difference between the value of that covariate in that location-year and the lower bound of the covariate divided by the difference between the upper and lower bounds for that covariate.

The composite SDI is the geometric mean of these three indices for a given location-year. For GBD 2021, final SDI values were multiplied by 100 for reporting, in order to improve understanding of and

broader engagement with the values[3].

11.3 SDI grouping and quantification

GBD 2021 SDI grouping and quantification are shown in the following tables (Table 9 and Table 10).

Table 6. GBD 2021 SDI groupings by location

Location Name	Level
Addis Ababa	Low SDI
Afar	Low SDI
Afghanistan	Low SDI
Amhara	Low SDI
Balochistan	Low SDI
Bauchi	Low SDI
Benin	Low SDI
Benishangul-Gumuz	Low SDI
Bihar, Rural	Low SDI
Borno	Low SDI
Burkina Faso	Low SDI
Burundi	Low SDI
Central African Republic	Low SDI
Chad	Low SDI
Côte d'Ivoire	Low SDI
Democratic Republic of the Congo	Low SDI
Dire Dawa	Low SDI
Eritrea	Low SDI
Gambella	Low SDI
Gambia	Low SDI
Gilgit-Baltistan	Low SDI
Gombe	Low SDI
Guinea	Low SDI
Guinea-Bissau	Low SDI
Haiti	Low SDI
Harari	Low SDI
Jharkhand, Rural	Low SDI
Jigawa	Low SDI
Kaduna	Low SDI
Kano	Low SDI
Katsina	Low SDI
Kebbi	Low SDI
Khyber Pakhtunkhwa	Low SDI
Liberia	Low SDI
Madagascar	Low SDI
Madhya Pradesh, Rural	Low SDI
Malawi	Low SDI

Mali	Low SDI
Mozambique	Low SDI
Nepal	Low SDI
Niger	Low SDI
Niger	Low SDI
Oromia	Low SDI
Papua New Guinea	Low SDI
Rwanda	Low SDI
Senegal	Low SDI
Sierra Leone	Low SDI
Sokoto	Low SDI
Solomon Islands	Low SDI
Somali	Low SDI
Somalia	Low SDI
South Sudan	Low SDI
Southern Nations, Nationalities, and Peoples	Low SDI
Taraba	Low SDI
Tigray	Low SDI
Timor-Leste	Low SDI
Togo	Low SDI
Uganda	Low SDI
United Republic of Tanzania	Low SDI
Yemen	Low SDI
Yobe	Low SDI
Zamfara	Low SDI
Abia	Low-middle SDI
Abra	Low-middle SDI
Aceh	Low-middle SDI
Acre	Low-middle SDI
Adamawa	Low-middle SDI
Aguascalientes	Low-middle SDI
Agusan Del Norte	Low-middle SDI
Agusan Del Sur	Low-middle SDI
Aklan	Low-middle SDI
Akwa Ibom	Low-middle SDI
Alagoas	Low-middle SDI
Albania	Low-middle SDI
Albay	Low-middle SDI
Alborz	Low-middle SDI
Amazonas	Low-middle SDI
Andhra Pradesh, Rural	Low-middle SDI
Angola	Low-middle SDI
Arunachal Pradesh, Rural	Low-middle SDI
Assam, Rural	Low-middle SDI
Azad Jammu & Kashmir	Low-middle SDI
Bahia	Low-middle SDI
Bangladesh	Low-middle SDI

Baringo	Low-middle SDI
Bayelsa	Low-middle SDI
Belize	Low-middle SDI
Bengkulu	Low-middle SDI
Benue	Low-middle SDI
Bhutan	Low-middle SDI
Bihar, Urban	Low-middle SDI
Bolivia (Plurinational State of)	Low-middle SDI
Bomet	Low-middle SDI
Bungoma	Low-middle SDI
Busia	Low-middle SDI
Cabo Verde	Low-middle SDI
Cambodia	Low-middle SDI
Cameroon	Low-middle SDI
Ceará	Low-middle SDI
Central Java	Low-middle SDI
Central Sulawesi	Low-middle SDI
Chhattisgarh, Rural	Low-middle SDI
Comoros	Low-middle SDI
Congo	Low-middle SDI
Cross River	Low-middle SDI
Delta	Low-middle SDI
Democratic People's Republic of Korea	Low-middle SDI
Djibouti	Low-middle SDI
East Nusa Tenggara	Low-middle SDI
Ebonyi	Low-middle SDI
Egypt	Low-middle SDI
Ekiti	Low-middle SDI
El Salvador	Low-middle SDI
Elgeyo-Marakwet	Low-middle SDI
Embu	Low-middle SDI
Enugu	Low-middle SDI
Eswatini	Low-middle SDI
Garissa	Low-middle SDI
Ghana	Low-middle SDI
Gorontalo	Low-middle SDI
Guatemala	Low-middle SDI
Gujarat, Rural	Low-middle SDI
Haryana, Rural	Low-middle SDI
Homa Bay	Low-middle SDI
Honduras	Low-middle SDI
Isiolo	Low-middle SDI
Jammu & Kashmir and Ladakh, Rural	Low-middle SDI
Kajiado	Low-middle SDI
Kakamega	Low-middle SDI
Karnataka, Rural	Low-middle SDI
Kericho	Low-middle SDI

Kiambu	Low-middle SDI
Kilifi	Low-middle SDI
Kiribati	Low-middle SDI
Kirinyaga	Low-middle SDI
Kisii	Low-middle SDI
Kisumu	Low-middle SDI
Kitui	Low-middle SDI
Kogi	Low-middle SDI
Kwale	Low-middle SDI
Kwara	Low-middle SDI
Kyrgyzstan	Low-middle SDI
Laikipia	Low-middle SDI
Lampung	Low-middle SDI
Lamu	Low-middle SDI
Lao People's Democratic Republic	Low-middle SDI
Lesotho	Low-middle SDI
Machakos	Low-middle SDI
Maharashtra, Rural	Low-middle SDI
Makueni	Low-middle SDI
Maluku	Low-middle SDI
Mandera	Low-middle SDI
Manipur, Rural	Low-middle SDI
Maranhão	Low-middle SDI
Marsabit	Low-middle SDI
Marshall Islands	Low-middle SDI
Mauritania	Low-middle SDI
Meghalaya, Rural	Low-middle SDI
Meru	Low-middle SDI
Micronesia (Federated States of)	Low-middle SDI
Migori	Low-middle SDI
Mizoram, Rural	Low-middle SDI
Mombasa	Low-middle SDI
Mongolia	Low-middle SDI
Morocco	Low-middle SDI
Murang'a	Low-middle SDI
Myanmar	Low-middle SDI
Nagaland, Rural	Low-middle SDI
Nairobi	Low-middle SDI
Nakuru	Low-middle SDI
Namibia	Low-middle SDI
Nandi	Low-middle SDI
Narok	Low-middle SDI
Nasarawa	Low-middle SDI
Nicaragua	Low-middle SDI
North Maluku	Low-middle SDI
Nyamira	Low-middle SDI
Nyandarua	Low-middle SDI

Nyeri	Low-middle SDI
Odisha, Rural	Low-middle SDI
Ogun	Low-middle SDI
Ondo	Low-middle SDI
Other Union Territories, Rural	Low-middle SDI
Oyo	Low-middle SDI
Pará	Low-middle SDI
Paraíba	Low-middle SDI
Pernambuco	Low-middle SDI
Piaui	Low-middle SDI
Plateau	Low-middle SDI
Punjab	Low-middle SDI
Punjab, Rural	Low-middle SDI
Rajasthan, Rural	Low-middle SDI
Rio Grande do Norte	Low-middle SDI
Rondônia	Low-middle SDI
Roraima	Low-middle SDI
Algeria	Middle SDI
Amapá	Middle SDI
Anambra	Middle SDI
Andhra Pradesh, Urban	Middle SDI
Anhui	Middle SDI
Antique	Middle SDI
Apayao	Middle SDI
Ardebil	Middle SDI
Armenia	Middle SDI
Arunachal Pradesh, Urban	Middle SDI
Assam, Urban	Middle SDI
Aurora	Middle SDI
Azerbaijan	Middle SDI
Baja California	Middle SDI
Baja California Sur	Middle SDI
Bali	Middle SDI
Bangka-Belitung Islands	Middle SDI
Banten	Middle SDI
Basilan	Middle SDI
Bataan	Middle SDI
Batanes	Middle SDI
Batangas	Middle SDI
Benguet	Middle SDI
Biliran	Middle SDI
Bohol	Middle SDI
Botswana	Middle SDI
Bukidnon	Middle SDI
Bulacan	Middle SDI
Bushehr	Middle SDI
Cagayan	Middle SDI

Camarines Norte	Middle SDI
Camarines Sur	Middle SDI
Camiguin	Middle SDI
Campeche	Middle SDI
Capiz	Middle SDI
Catanduanes	Middle SDI
Cavite	Middle SDI
Cebu	Middle SDI
Central Kalimantan	Middle SDI
Chahar Mahaal and Bakhtiari	Middle SDI
Chhattisgarh, Urban	Middle SDI
Chiapas	Middle SDI
Chihuahua	Middle SDI
Coahuila	Middle SDI
Colima	Middle SDI
Colombia	Middle SDI
Costa Rica	Middle SDI
Cotabato (North Cotabato)	Middle SDI
Cuba	Middle SDI
Davao de Oro	Middle SDI
Davao Del Norte	Middle SDI
Davao Del Sur	Middle SDI
Davao Occidental	Middle SDI
Davao Oriental	Middle SDI
Delhi, Rural	Middle SDI
Dinagat Islands	Middle SDI
Dominican Republic	Middle SDI
Durango	Middle SDI
East Azarbayejan	Middle SDI
East Java	Middle SDI
Eastern Cape	Middle SDI
Eastern Samar	Middle SDI
Ecuador	Middle SDI
Edo	Middle SDI
Equatorial Guinea	Middle SDI
Espírito Santo	Middle SDI
Fars	Middle SDI
FCT (Abuja)	Middle SDI
Fiji	Middle SDI
Free State	Middle SDI
Gabon	Middle SDI
Gansu	Middle SDI
Gauteng	Middle SDI
Gilan	Middle SDI
Goa, Rural	Middle SDI
Goiás	Middle SDI
Golestan	Middle SDI

Grenada	Middle SDI
Guanajuato	Middle SDI
Guangxi	Middle SDI
Guerrero	Middle SDI
Guimaras	Middle SDI
Guizhou	Middle SDI
Gujarat, Urban	Middle SDI
Guyana	Middle SDI
Hainan	Middle SDI
Hamadan	Middle SDI
Haryana, Urban	Middle SDI
Hebei	Middle SDI
Henan	Middle SDI
Hidalgo	Middle SDI
Himachal Pradesh, Rural	Middle SDI
Hormozgan	Middle SDI
Hubei	Middle SDI
Hunan	Middle SDI
Ifugao	Middle SDI
Ilam	Middle SDI
Ilocos Norte	Middle SDI
Ilocos Sur	Middle SDI
Iloilo	Middle SDI
Imo	Middle SDI
Iraq	Middle SDI
Isabela	Middle SDI
Isfahan	Middle SDI
Islamabad Capital Territory	Middle SDI
Jalisco	Middle SDI
Jamaica	Middle SDI
Jambi	Middle SDI
Jammu & Kashmir and Ladakh, Urban	Middle SDI
Jharkhand, Urban	Middle SDI
Jiangxi	Middle SDI
Kalinga	Middle SDI
Karnataka, Urban	Middle SDI
Kerala, Rural	Middle SDI
Kerala, Urban	Middle SDI
Kerman	Middle SDI
Kermanshah	Middle SDI
Khorasan-e-Razavi	Middle SDI
Khuzestan	Middle SDI
Kohgiluyeh and Boyer-Ahmad	Middle SDI
Kurdistan	Middle SDI
KwaZulu-Natal	Middle SDI
La Union	Middle SDI
Lagos	Middle SDI

Laguna	Middle SDI
Lanao Del Norte	Middle SDI
Lanao Del Sur	Middle SDI
Leyte	Middle SDI
Limpopo	Middle SDI
Lorestan	Middle SDI
Madhya Pradesh, Urban	Middle SDI
Maguindanao	Middle SDI
Maharashtra, Urban	Middle SDI
Maldives	Middle SDI
Manipur, Urban	Middle SDI
Marinduque	Middle SDI
Markazi	Middle SDI
Masbate	Middle SDI
Mato Grosso	Middle SDI
Mato Grosso do Sul	Middle SDI
Mazandaran	Middle SDI
Meghalaya, Urban	Middle SDI
México	Middle SDI
Mexico City	Middle SDI
Michoacán de Ocampo	Middle SDI
Minas Gerais	Middle SDI
Misamis Occidental	Middle SDI
Misamis Oriental	Middle SDI
Mizoram, Urban	Middle SDI
Morelos	Middle SDI
Mountain Province	Middle SDI
Mpumalanga	Middle SDI
Nagaland, Urban	Middle SDI
National Capital Region	Middle SDI
Nauru	Middle SDI
Nayarit	Middle SDI
Negros Occidental	Middle SDI
Negros Oriental	Middle SDI
Ningxia	Middle SDI
North Khorasan	Middle SDI
North Sulawesi	Middle SDI
North Sumatra	Middle SDI
Northern Cape	Middle SDI
Northern Samar	Middle SDI
North-West	Middle SDI
Nueva Ecija	Middle SDI
Nueva Vizcaya	Middle SDI
Nuevo León	Middle SDI
Oaxaca	Middle SDI
Occidental Mindoro	Middle SDI
Odisha, Urban	Middle SDI

Oriental Mindoro	Middle SDI
Osun	Middle SDI
Other Union Territories, Urban	Middle SDI
Palawan	Middle SDI
Palestine	Middle SDI
Pampanga	Middle SDI
Panama	Middle SDI
Pangasinan	Middle SDI
Papua	Middle SDI
Paraguay	Middle SDI
Paraná	Middle SDI
Peru	Middle SDI
Puebla	Middle SDI
Punjab, Urban	Middle SDI
Qazvin	Middle SDI
Qinghai	Middle SDI
Qom	Middle SDI
Querétaro	Middle SDI
Quezon	Middle SDI
Quintana Roo	Middle SDI
Quirino	Middle SDI
Rajasthan, Urban	Middle SDI
Rio de Janeiro	Middle SDI
Rio Grande do Sul	Middle SDI
Rivers	Middle SDI
Rizal	Middle SDI
Romblon	Middle SDI
Saint Lucia	Middle SDI
Saint Vincent and the Grenadines	Middle SDI
Samar (Western Samar)	Middle SDI
Samburu	Middle SDI
Samoa	Middle SDI
San Luis Potosí	Middle SDI
Santa Catarina	Middle SDI
São Paulo	Middle SDI
Sao Tome and Principe	Middle SDI
Sarangani	Middle SDI
Semnan	Middle SDI
Sergipe	Middle SDI
Shanxi	Middle SDI
Siaya	Middle SDI
Sichuan	Middle SDI
Sikkim, Rural	Middle SDI
Sikkim, Urban	Middle SDI
Sinaloa	Middle SDI
Sindh	Middle SDI
Siquijor	Middle SDI

Sistan and Baluchistan	Middle SDI
Sonora	Middle SDI
Sorsogon	Middle SDI
South Cotabato	Middle SDI
South Kalimantan	Middle SDI
South Khorasan	Middle SDI
South Sulawesi	Middle SDI
South Sumatra	Middle SDI
Southeast Sulawesi	Middle SDI
Southern Leyte	Middle SDI
Sri Lanka	Middle SDI
Sudan	Middle SDI
Sultan Kudarat	Middle SDI
Sulu	Middle SDI
Surigao Del Norte	Middle SDI
Surigao Del Sur	Middle SDI
Suriname	Middle SDI
Syrian Arab Republic	Middle SDI
Tabasco	Middle SDI
Taita Taveta	Middle SDI
Tajikistan	Middle SDI
Tamaulipas	Middle SDI
Tamil Nadu, Rural	Middle SDI
Tamil Nadu, Urban	Middle SDI
Tana River	Middle SDI
Tarlac	Middle SDI
Tawi-Tawi	Middle SDI
Tehran	Middle SDI
Telangana, Rural	Middle SDI
Telangana, Urban	Middle SDI
Thailand	Middle SDI
Tharaka Nithi	Middle SDI
Tibet	Middle SDI
Tlaxcala	Middle SDI
Tocantins	Middle SDI
Tokelau	Middle SDI
Tonga	Middle SDI
Trans Nzoia	Middle SDI
Tripura, Rural	Middle SDI
Tripura, Urban	Middle SDI
Tunisia	Middle SDI
Turkana	Middle SDI
Turkmenistan	Middle SDI
Tuvalu	Middle SDI
Uasin Gishu	Middle SDI
Uttar Pradesh, Rural	Middle SDI
Uttar Pradesh, Urban	Middle SDI

Uttarakhand, Rural	Middle SDI
Uzbekistan	Middle SDI
Vanuatu	Middle SDI
Venezuela (Bolivarian Republic of)	Middle SDI
Veracruz de Ignacio de la Llave	Middle SDI
Viet Nam	Middle SDI
Vihiga	Middle SDI
Wajir	Middle SDI
West Azarbayejan	Middle SDI
West Bengal, Rural	Middle SDI
West Bengal, Urban	Middle SDI
West Java	Middle SDI
West Kalimantan	Middle SDI
West Nusa Tenggara	Middle SDI
West Papua	Middle SDI
West Pokot	Middle SDI
West Sulawesi	Middle SDI
West Sumatra	Middle SDI
Western Cape	Middle SDI
Xinjiang	Middle SDI
Yazd	Middle SDI
Yogyakarta	Middle SDI
Yucatán	Middle SDI
Yunnan	Middle SDI
Zacatecas	Middle SDI
Zambales	Middle SDI
Zambia	Middle SDI
Zamboanga Del Norte	Middle SDI
Zamboanga Del Sur	Middle SDI
Zamboanga Sibugay	Middle SDI
Zanjan	Middle SDI
Zimbabwe	Middle SDI
Abruzzo	High-middle SDI
Altai kray	High-middle SDI
American Samoa	High-middle SDI
Amur oblast	High-middle SDI
Antigua and Barbuda	High-middle SDI
Argentina	High-middle SDI
Arkhangelsk oblast without Nenets autonomous district	High-middle SDI
Astrakhan oblast	High-middle SDI
Bahamas	High-middle SDI
Bahrain	High-middle SDI
Barbados	High-middle SDI
Basilicata	High-middle SDI
Belarus	High-middle SDI
Belgorod oblast	High-middle SDI
Bosnia and Herzegovina	High-middle SDI

Brunei Darussalam	High-middle SDI
Bryansk oblast	High-middle SDI
Bulgaria	High-middle SDI
Calabria	High-middle SDI
Campania	High-middle SDI
Chechen Republic	High-middle SDI
Chelyabinsk oblast	High-middle SDI
Chile	High-middle SDI
Chongqing	High-middle SDI
Chukotka Autonomous Area	High-middle SDI
Chuvash Republic	High-middle SDI
Cook Islands	High-middle SDI
Croatia	High-middle SDI
Delhi, Urban	High-middle SDI
Distrito Federal	High-middle SDI
Dominica	High-middle SDI
East Kalimantan	High-middle SDI
Emilia-Romagna	High-middle SDI
Friuli-Venezia Giulia	High-middle SDI
Fujian	High-middle SDI
Georgia	High-middle SDI
Goa, Urban	High-middle SDI
Greece	High-middle SDI
Guam	High-middle SDI
Guangdong	High-middle SDI
Heilongjiang	High-middle SDI
Himachal Pradesh, Urban	High-middle SDI
Hungary	High-middle SDI
Inner Mongolia	High-middle SDI
Irkutsk oblast	High-middle SDI
Israel	High-middle SDI
Ivanovo oblast	High-middle SDI
Jakarta	High-middle SDI
Jewish autonomous oblast	High-middle SDI
Jiangsu	High-middle SDI
Jilin	High-middle SDI
Jordan	High-middle SDI
Kabardino-Balkar Republic	High-middle SDI
Kaliningrad oblast	High-middle SDI
Kaluga oblast	High-middle SDI
Kamchatka kray	High-middle SDI
Karachay-Cherkess Republic	High-middle SDI
Kazakhstan	High-middle SDI
Kemerovo oblast	High-middle SDI
Khabarovsk kray	High-middle SDI
Khanty-Mansi autonomous area	High-middle SDI
Kirov oblast	High-middle SDI

Komi Republic	High-middle SDI
Kostroma oblast	High-middle SDI
Krasnodar kray	High-middle SDI
Krasnoyarsk kray	High-middle SDI
Kurgan oblast	High-middle SDI
Kursk oblast	High-middle SDI
Lazio	High-middle SDI
Lebanon	High-middle SDI
Leningrad oblast	High-middle SDI
Liaoning	High-middle SDI
Libya	High-middle SDI
Liguria	High-middle SDI
Lipetzk oblast	High-middle SDI
Lombardia	High-middle SDI
Magadan oblast	High-middle SDI
Malaysia	High-middle SDI
Malta	High-middle SDI
Marche	High-middle SDI
Mauritius	High-middle SDI
Molise	High-middle SDI
Montenegro	High-middle SDI
Moscow City	High-middle SDI
Moscow oblast	High-middle SDI
Murmansk oblast	High-middle SDI
Nenets autonomous district	High-middle SDI
Niue	High-middle SDI
Nizhny Novgorod oblast	High-middle SDI
North Kalimantan	High-middle SDI
North Macedonia	High-middle SDI
Northern Mariana Islands	High-middle SDI
Novgorod oblast	High-middle SDI
Novosibirsk oblast	High-middle SDI
Oman	High-middle SDI
Omsk oblast	High-middle SDI
Orenburg oblast	High-middle SDI
Oryol oblast	High-middle SDI
Palau	High-middle SDI
Penza oblast	High-middle SDI
Perm kray	High-middle SDI
Piemonte	High-middle SDI
Portugal	High-middle SDI
Primorsky kray	High-middle SDI
Provincia autonoma di Bolzano	High-middle SDI
Provincia autonoma di Trento	High-middle SDI
Pskov oblast	High-middle SDI
Puglia	High-middle SDI
Republic of Adygeya	High-middle SDI

Republic of Altai	High-middle SDI
Republic of Bashkortostan	High-middle SDI
Republic of Buryatia	High-middle SDI
Republic of Crimea	High-middle SDI
Republic of Dagestan	High-middle SDI
Republic of Ingushetia	High-middle SDI
Republic of Kalmykia	High-middle SDI
Republic of Karelia	High-middle SDI
Republic of Khakassia	High-middle SDI
Republic of Mari El	High-middle SDI
Republic of Moldova	High-middle SDI
Republic of Mordovia	High-middle SDI
Republic of North Ossetia-Alania	High-middle SDI
Republic of Sakha (Yakutia)	High-middle SDI
Republic of Tatarstan	High-middle SDI
Republic of Tuva	High-middle SDI
Riau	High-middle SDI
Riau Islands	High-middle SDI
Romania	High-middle SDI
Rostov oblast	High-middle SDI
Ryazan oblast	High-middle SDI
Saint Kitts and Nevis	High-middle SDI
Saint Petersburg	High-middle SDI
Sakhalin oblast	High-middle SDI
Samara oblast	High-middle SDI
Saratov oblast	High-middle SDI
Sardegna	High-middle SDI
Serbia	High-middle SDI
Sevastopol	High-middle SDI
Seychelles	High-middle SDI
Shaanxi	High-middle SDI
Shandong	High-middle SDI
Sicilia	High-middle SDI
Slovakia	High-middle SDI
Smolensk oblast	High-middle SDI
Spain	High-middle SDI
Stavropol kray	High-middle SDI
Sverdlovsk oblast	High-middle SDI
Tambov oblast	High-middle SDI
Tomsk oblast	High-middle SDI
Toscana	High-middle SDI
Trinidad and Tobago	High-middle SDI
Tula oblast	High-middle SDI
Türkiye	High-middle SDI
Tver oblast	High-middle SDI
Tyumen oblast without autonomous areas	High-middle SDI
Udmurt Republic	High-middle SDI

Ukraine (without Crimea & Sevastopol)	High-middle SDI
Ulyanovsk oblast	High-middle SDI
Umbria	High-middle SDI
Uruguay	High-middle SDI
Uttarakhand, Urban	High-middle SDI
Valle d'Aosta	High-middle SDI
Veneto	High-middle SDI
Vladimir oblast	High-middle SDI
Volgograd oblast	High-middle SDI
Vologda oblast	High-middle SDI
Voronezh oblast	High-middle SDI
Yamalo-Nenets autonomous area	High-middle SDI
Yaroslavl oblast	High-middle SDI
Zabaikalsk kray	High-middle SDI
Zhejiang	High-middle SDI
Agder	High SDI
Aichi	High SDI
Akita	High SDI
Alabama	High SDI
Alaska	High SDI
Andorra	High SDI
Aomori	High SDI
Arizona	High SDI
Arkansas	High SDI
Australia	High SDI
Austria	High SDI
Barking and Dagenham	High SDI
Barnet	High SDI
Barnsley	High SDI
Bath and North East Somerset	High SDI
Bedford	High SDI
Beijing	High SDI
Belgium	High SDI
Bermuda	High SDI
Bexley	High SDI
Birmingham	High SDI
Blackburn with Darwen	High SDI
Blackpool	High SDI
Bolton	High SDI
Bournemouth	High SDI
Bracknell Forest	High SDI
Bradford	High SDI
Brent	High SDI
Brighton and Hove	High SDI
Bristol, City of	High SDI
Bromley	High SDI
Buckinghamshire	High SDI

Bury	High SDI
Calderdale	High SDI
California	High SDI
Camden	High SDI
Canada	High SDI
Central Bedfordshire	High SDI
Cheshire East	High SDI
Cheshire West and Chester	High SDI
Chiba	High SDI
Colorado	High SDI
Connecticut	High SDI
Cornwall	High SDI
County Durham	High SDI
Coventry	High SDI
Croydon	High SDI
Cumbria	High SDI
Cyprus	High SDI
Czechia	High SDI
Darlington	High SDI
Delaware	High SDI
Denmark	High SDI
Derby	High SDI
Derbyshire	High SDI
Devon	High SDI
District of Columbia	High SDI
Dolnośląskie	High SDI
Doncaster	High SDI
Dorset	High SDI
Dudley	High SDI
Ealing	High SDI
East Riding of Yorkshire	High SDI
East Sussex	High SDI
Ehime	High SDI
Enfield	High SDI
Essex	High SDI
Estonia	High SDI
Finland	High SDI
Florida	High SDI
France	High SDI
Fukui	High SDI
Fukuoka	High SDI
Fukushima	High SDI
Gateshead	High SDI
Georgia	High SDI
Germany	High SDI
Gifu	High SDI
Gloucestershire	High SDI

Greenland	High SDI
Greenwich	High SDI
Gunma	High SDI
Hackney	High SDI
Halton	High SDI
Hammersmith and Fulham	High SDI
Hampshire	High SDI
Haringey	High SDI
Harrow	High SDI
Hartlepool	High SDI
Havering	High SDI
Hawaii	High SDI
Herefordshire, County of	High SDI
Hertfordshire	High SDI
Hillingdon	High SDI
Hiroshima	High SDI
Hokkaidō	High SDI
Hong Kong Special Administrative Region of China	High SDI
Hounslow	High SDI
Hyōgo	High SDI
Ibaraki	High SDI
Iceland	High SDI
Idaho	High SDI
Illinois	High SDI
Indiana	High SDI
Innlandet	High SDI
Iowa	High SDI
Ireland	High SDI
Ishikawa	High SDI
Isle of Wight	High SDI
Islington	High SDI
Iwate	High SDI
Kagawa	High SDI
Kagoshima	High SDI
Kanagawa	High SDI
Kansas	High SDI
Kensington and Chelsea	High SDI
Kent	High SDI
Kentucky	High SDI
Kingston upon Hull, City of	High SDI
Kingston upon Thames	High SDI
Kirklees	High SDI
Knowsley	High SDI
Kōchi	High SDI
Kujawsko-Pomorskie	High SDI
Kumamoto	High SDI
Kuwait	High SDI

Kyōto	High SDI
Lambeth	High SDI
Lancashire	High SDI
Latvia	High SDI
Leeds	High SDI
Leicester	High SDI
Leicestershire	High SDI
Lewisham	High SDI
Lincolnshire	High SDI
Lithuania	High SDI
Liverpool	High SDI
Łódzkie	High SDI
Louisiana	High SDI
Lubelskie	High SDI
Lubuskie	High SDI
Luton	High SDI
Luxembourg	High SDI
Macao Special Administrative Region of China	High SDI
Maine	High SDI
Małopolskie	High SDI
Manchester	High SDI
Maryland	High SDI
Massachusetts	High SDI
Mazowieckie	High SDI
Medway	High SDI
Merton	High SDI
Michigan	High SDI
Middlesbrough	High SDI
Mie	High SDI
Milton Keynes	High SDI
Minnesota	High SDI
Mississippi	High SDI
Missouri	High SDI
Miyagi	High SDI
Miyazaki	High SDI
Monaco	High SDI
Montana	High SDI
Møre og Romsdal	High SDI
Nagano	High SDI
Nagasaki	High SDI
Nara	High SDI
Nebraska	High SDI
Netherlands	High SDI
Nevada	High SDI
New Hampshire	High SDI
New Jersey	High SDI
New Mexico	High SDI

New York	High SDI
New Zealand non-Maori population	High SDI
Newcastle upon Tyne	High SDI
Newham	High SDI
Niigata	High SDI
Nordland	High SDI
Norfolk	High SDI
North Carolina	High SDI
North Dakota	High SDI
North East Lincolnshire	High SDI
North Lincolnshire	High SDI
North Somerset	High SDI
North Tyneside	High SDI
North Yorkshire	High SDI
Northamptonshire	High SDI
Northern Ireland	High SDI
Northumberland	High SDI
Nottingham	High SDI
Nottinghamshire	High SDI
Ohio	High SDI
Ōita	High SDI
Okayama	High SDI
Okinawa	High SDI
Oklahoma	High SDI
Oldham	High SDI
Opolskie	High SDI
Oregon	High SDI
Ōsaka	High SDI
Oslo	High SDI
Oxfordshire	High SDI
Pennsylvania	High SDI
Peterborough	High SDI
Plymouth	High SDI
Podkarpackie	High SDI
Podlaskie	High SDI
Pomorskie	High SDI
Poole	High SDI
Portsmouth	High SDI
Puerto Rico	High SDI
Qatar	High SDI
Reading	High SDI
Redbridge	High SDI
Redcar and Cleveland	High SDI
Republic of Korea	High SDI
Rhode Island	High SDI
Richmond upon Thames	High SDI
Rochdale	High SDI

Rogaland	High SDI
Rotherham	High SDI
Rutland	High SDI
Saga	High SDI
Saitama	High SDI
Salford	High SDI
San Marino	High SDI
Sandwell	High SDI
Saudi Arabia	High SDI
Scotland	High SDI
Sefton	High SDI
Shanghai	High SDI
Sheffield	High SDI
Shiga	High SDI
Shimane	High SDI
Shizuoka	High SDI
Shropshire	High SDI
Singapore	High SDI
Śląskie	High SDI
Slough	High SDI
Slovenia	High SDI
Solihull	High SDI
Somerset	High SDI
South Carolina	High SDI
South Dakota	High SDI
South Gloucestershire	High SDI
South Tyneside	High SDI
Southampton	High SDI
Southend-on-Sea	High SDI
Southwark	High SDI
St Helens	High SDI
Staffordshire	High SDI
Stockholm	High SDI
Stockport	High SDI
Stockton-on-Tees	High SDI
Stoke-on-Trent	High SDI
Suffolk	High SDI
Sunderland	High SDI
Surrey	High SDI
Sutton	High SDI
Sweden except Stockholm	High SDI
Świętokrzyskie	High SDI
Swindon	High SDI
Switzerland	High SDI
Taiwan (Province of China)	High SDI
Tameside	High SDI
Telford and Wrekin	High SDI

Tennessee	High SDI
Texas	High SDI
Thurrock	High SDI
Tianjin	High SDI
Tochigi	High SDI
Tokushima	High SDI
Tōkyō	High SDI
Torbay	High SDI
Tottori	High SDI
Tower Hamlets	High SDI
Toyama	High SDI
Trafford	High SDI
Troms og Finnmark	High SDI
Trøndelag	High SDI
United Arab Emirates	High SDI
United States Virgin Islands	High SDI
Utah	High SDI
Vermont	High SDI
Vestfold og Telemark	High SDI
Vestland	High SDI
Viken	High SDI
Virginia	High SDI
Wakayama	High SDI
Wakefield	High SDI
Wales	High SDI
Walsall	High SDI
Waltham Forest	High SDI
Wandsworth	High SDI
Warmińsko-Mazurskie	High SDI
Warrington	High SDI
Warwickshire	High SDI
Washington	High SDI
West Berkshire	High SDI
West Sussex	High SDI
West Virginia	High SDI
Westminster	High SDI
Wielkopolskie	High SDI
Wigan	High SDI
Wiltshire	High SDI
Windsor and Maidenhead	High SDI
Wirral	High SDI
Wisconsin	High SDI
Wokingham	High SDI
Wolverhampton	High SDI
Worcestershire	High SDI
Wyoming	High SDI
Yamagata	High SDI

Yamaguchi	High SDI
Yamanashi	High SDI
York	High SDI
Zachodniopomorskie	High SDI

Table 7. GBD 2021 SDI quantification

Location Name	Value
Abra	0.654174
Abruzzo	0.816497
Aceh	0.671759
Acre	0.562075
Addis Ababa	0.695288
Afar	0.286503
Afghanistan	0.335068
Agder	0.907093
Aguascalientes	0.682557
Agusan Del Norte	0.615708
Agusan Del Sur	0.541666
Aichi	0.883329
Akita	0.832381
Aklan	0.640421
Alabama	0.826418
Alagoas	0.529743
Alaska	0.857126
Albania	0.706889
Albay	0.640151
Alborz	0.748209
Algeria	0.65972
AmapÃ¡	0.629808
Amazonas	0.603586
American Samoa	0.726268
Amhara	0.322156
Andorra	0.869895
Angola	0.482946
Antigua and Barbuda	0.74985
Antique	0.569823
ÅŒita	0.848716
ÅŒsaka	0.876433
Aomori	0.828694
Apayao	0.607701
Ardebil	0.658778
Argentina	0.733528
Arizona	0.847683
Arkansas	0.816754
Armenia	0.702497
Aurora	0.614063

Australia	0.844269
Austria	0.854558
Azad Jammu & Kashmir	0.541343
Azerbaijan	0.695411
B angka-Belitung Islands	0.644388
B uckinghamshire	0.888192
Bahamas	0.805144
Bahia	0.574142
Bahrain	0.752218
Baja California	0.704777
Baja California Sur	0.710175
Bali	0.652383
Balochistan	0.41711
Bangladesh	0.493106
Banten	0.641544
Barbados	0.747065
Baringo	0.514622
Barking and Dagenham	0.806426
Barnet	0.885111
Barnsley	0.788112
Basilan	0.546109
Basilicata	0.783798
Bataan	0.660374
Batanes	0.682566
Batangas	0.686055
Bath and North East Somerset	0.895076
Bedford	0.856962
Belarus	0.784114
Belgium	0.853674
Belize	0.610552
Bengkulu	0.613885
Benguet	0.716293
Benin	0.374522
Benishangul-Gumuz	0.323655
Bermuda	0.82132
Bexley	0.844098
Bhutan	0.476725
Biliran	0.643028
Birmingham	0.836949
Blackburn with Darwen	0.810134
Blackpool	0.788905
Bohol	0.604934
Bolivia (Plurinational State of)	0.604497
Bolton	0.812596
Bomet	0.530036
Bosnia and Herzegovina	0.722964
Botswana	0.643078

Bournemouth	0.87015
Bracknell Forest	0.890716
Bradford	0.814924
Brent	0.858684
Brighton and Hove	0.897901
Bristol, City of	0.896566
Bromley	0.869194
Brunei Darussalam	0.810289
Bukidnon	0.551059
Bulacan	0.708003
Bulgaria	0.764641
Bungoma	0.488275
Burkina Faso	0.284471
Burundi	0.291289
Bury	0.828817
Bushehr	0.708677
Busia	0.47817
CÃ´te d'Ivoire	0.424541
Cabo Verde	0.533601
Cagayan	0.634333
Calabria	0.775305
Calderdale	0.836996
California	0.87109
Camarines Norte	0.594459
Camarines Sur	0.633744
Cambodia	0.474
Cambridgeshire	0.88763
Camden	0.936076
Cameroon	0.480365
Camiguin	0.63209
Campania	0.766832
Campeche	0.665088
Canada	0.873182
Capiz	0.5714
Catanduanes	0.609423
Cavite	0.729179
CearÃ¡	0.563913
Cebu	0.658155
Central African Republic	0.311027
Central Bedfordshire	0.851319
Central Java	0.61378
Central Kalimantan	0.639931
Central Sulawesi	0.617545
Chad	0.243517
Chahar Mahaal and Bakhtiari	0.678339
Cheshire East	0.884279
Cheshire West and Chester	0.870242

Chiapas	0.569757
Chiba	0.861645
Chihuahua	0.674472
Chile	0.770149
Coahuila	0.678075
Colima	0.699338
Colombia	0.65664
Colorado	0.875986
Comoros	0.476956
Congo	0.586909
Connecticut	0.901972
Cook Islands	0.778252
Cornwall	0.839219
Costa Rica	0.70437
Cotabato (North Cotabato)	0.552112
County Durham	0.810755
Coventry	0.847336
Croatia	0.799069
Croydon	0.85144
Cuba	0.669332
Cumbria	0.842326
Cyprus	0.835649
Czechia	0.82851
Darlington	0.835428
Davao de Oro	0.533099
Davao Del Norte	0.636971
Davao Del Sur	0.659918
Davao Occidental	0.578315
Davao Oriental	0.547237
Delaware	0.866079
Democratic People's Republic of Korea	0.569455
Democratic Republic of the Congo	0.390178
Denmark	0.897314
Derby	0.844077
Derbyshire	0.823175
Devon	0.854622
Dinagat Islands	0.622041
Dire Dawa	0.542618
District of Columbia	0.907427
Distrito Federal	0.776152
Djibouti	0.4892
Dominica	0.747382
Dominican Republic	0.619171
Doncaster	0.793628
Dorset	0.851417
Dudley	0.802586
Durango	0.640563

Ealing	0.882007
East Azarbayejan	0.667934
East Java	0.64654
East Kalimantan	0.761652
East Nusa Tenggara	0.550546
East Riding of Yorkshire	0.835194
East Sussex	0.838987
Eastern Cape	0.619101
Eastern Samar	0.4998
Ecuador	0.665675
Egypt	0.603962
Ehime	0.84378
El Salvador	0.56557
Elgeyo -Marakwet	0.525285
Embu	0.548378
Emilia-Romagna	0.829695
Enfield	0.845517
Equatorial Guinea	0.663978
Eritrea	0.404572
EspÃ-rito Santo	0.667429
Essex	0.844953
Estonia	0.845787
Eswatini	0.586217
Fars	0.715109
Fiji	0.669069
Finland	0.860244
Florida	0.861825
France	0.837816
Free State	0.678894
Friuli-Venezia Giulia	0.819858
Fukui	0.85658
Fukuoka	0.858415
Fukushima	0.841218
Gabon	0.639081
Gambella	0.460576
Gambia	0.410077
Garissa	0.32165
Gateshead	0.82858
Gauteng	0.736905
Georgia	0.733124
Georgia	0.847268
Germany	0.903516
Ghana	0.563348
Gifu	0.853448
Gilan	0.712362
Gilgit-Baltistan	0.399312
Gloucestershire	0.870918

GoiÃ¡s	0.639348
Golestan	0.656422
Gorontalo	0.57105
Greece	0.791882
Greenland	0.83564
Greenwich	0.845444
Grenada	0.669351
Guam	0.802168
Guanajuato	0.647045
Guatemala	0.540099
Guerrero	0.584127
Guimaras	0.60986
Guinea	0.336555
Guinea-Bissau	0.353448
Gunma	0.86177
Guyana	0.650902
Hackney	0.891329
Haiti	0.448751
Halton	0.835688
Hamadan	0.666969
Hammersmith and Fulham	0.934892
Hampshire	0.871999
Harari	0.53974
Haringey	0.871466
Harrow	0.858245
Hartlepool	0.797297
Havering	0.834177
Hawaii	0.87084
Herefordshire, County of	0.846195
Hertfordshire	0.886963
Hidalgo	0.633128
Hillingdon	0.892536
Hiroshima	0.87017
HokkaidA	0.844009
Homa Bay	0.507695
Honduras	0.513586
Hormozgan	0.670775
Hounslow	0.896361
Hungary	0.791025
HyÅg o	0.868491
Ibaraki	0.860523
Iceland	0.874629
Idaho	0.836495
Ifugao	0.597326
Ilam	0.705185
Illinois	0.880611
Ilocos Norte	0.687657

Ilocos Sur	0.671837
Iloilo	0.673508
Indiana	0.844051
Innlandet	0.899977
Iowa	0.864342
Iraq	0.662777
Ireland	0.87399
Isabela	0.636784
Isfahan	0.709894
Ishikawa	0.860215
Isiolo	0.435134
Islamabad Capital Territory	0.695559
Isle of Wight	0.826024
Islington	0.924303
Israel	0.809091
Iwate	0.835653
Jakarta	0.801238
Jalisco	0.677078
Jamaica	0.683064
Jambi	0.640779
Jordan	0.72542
KAchi	0.83589
Kagawa	0.857948
Kagoshima	0.832472
Kajiado	0.501635
Kakamega	0.508519
Kalinga	0.575239
Kanagawa	0.882744
Kansas	0.858891
Kazakhstan	0.718332
Kensington and Chelsea	0.946366
Kent	0.844501
Kentucky	0.821721
Kericho	0.520412
Kerman	0.668784
Kermanshah	0.674511
Khorasan-e-Razavi	0.67054
Khuzestan	0.669817
Khyber Pakhtunkhwa	0.451366
Kiambu	0.593046
Kilifi	0.486687
Kingston upon Hull, City of	0.797611
Kingston upon Thames	0.908394
Kiribati	0.525958
Kirinyaga	0.546741
Kirklees	0.823525
Kisii	0.549399

Kisumu	0.548721
Kitui	0.474177
Knowsley	0.811985
Kohgiluyeh and Boyer-Ahmad	0.694488
Kumamoto	0.834728
Kurdistan	0.642334
Kuwait	0.846802
Kwale	0.479657
KwaZulu-Natal	0.662386
KyÅt o	0.876289
Kyrgyzstan	0.609181
La Union	0.661006
Laguna	0.70162
Laikipia	0.576687
Lambeth	0.916016
Lampung	0.608913
Lamu	0.505575
Lanao Del Norte	0.587617
Lanao Del Sur	0.53291
Lancashire	0.839626
Lao People's Democratic Republic	0.489281
Latvia	0.830715
Lazio	0.827196
Lebanon	0.741226
Leeds	0.86788
Leicester	0.828051
Leicestershire	0.851101
Lesotho	0.511571
Lewisham	0.856729
Leyte	0.611702
Liberia	0.353229
Libya	0.735084
Liguria	0.821521
Limpopo	0.613432
Lincolnshire	0.820523
Lithuania	0.857613
Liverpool	0.847483
Lombardia	0.829091
Lorestan	0.669421
Louisiana	0.82667
Luton	0.838003
Luxembourg	0.884636
MÃ¸re og Romsdal	0.908149
MÃ©xico	0.681505
Machakos	0.550158
Madagascar	0.401385
Maguindanao	0.510702

Maine	0.866793
Makueni	0.514231
Malawi	0.381986
Malaysia	0.742553
Maldives	0.657665
Mali	0.271176
Malta	0.801854
Maluku	0.581624
Manchester	0.880573
Mandera	0.239927
MaranhÃ£o	0.492162
Marche	0.803749
Marinduque	0.54965
Markazi	0.682789
Marsabit	0.399977
Marshall Islands	0.573525
Maryland	0.891056
Masbate	0.45806
Massachusetts	0.90725
Mato Grosso	0.647044
Mato Grosso do Sul	0.642693
Mauritania	0.495267
Mauritius	0.717977
Mazandaran	0.729936
Medway	0.819462
Merton	0.887252
Meru	0.509152
Mexico City	0.759378
Michigan	0.864941
MichoacAjn de Ocampo	0.613949
Micronesia (Federated States of)	0.588013
Middlesbrough	0.798977
Mie	0.860795
Migori	0.482207
Milton Keynes	0.886756
Minas Gerais	0.648905
Minnesota	0.887884
Misamis Occidental	0.588185
Misamis Oriental	0.662873
Mississippi	0.811867
Missouri	0.849044
Miyagi	0.860059
Miyazaki	0.826769
Molise	0.787791
Mombasa	0.598167
Monaco	0.909519
Mongolia	0.618744

Montana	0.859517
Montenegro	0.796533
Morelos	0.670105
Morocco	0.56168
Mountain Province	0.519219
Mozambique	0.327475
Mpumalanga	0.648325
Murang'a	0.552922
Myanmar	0.528492
Nagano	0.858967
Nagasaki	0.829106
Nairobi	0.684189
Nakuru	0.572109
Namibia	0.618074
Nandi	0.51604
Nara	0.851417
Narok	0.458227
National Capital Region	0.751536
Nauru	0.62755
Nayarit	0.657929
Nebraska	0.865629
Negros Occidental	0.604235
Negros Oriental	0.578181
Nepal	0.433953
Netherlands	0.888376
Nevada	0.847864
New Hampshire	0.898526
New Jersey	0.891851
New Mexico	0.832846
New York	0.885926
Newcastle upon Tyne	0.871588
Newham	0.840478
Nicaragua	0.523647
Niger	0.17031
Niigata	0.845234
Niue	0.726219
Nordland	0.898214
Norfolk	0.836989
North Carolina	0.846174
North Dakota	0.876135
North East Lincolnshire	0.803564
North Kalimantan	0.754016
North Khorasan	0.651483
North Lincolnshire	0.824583
North Macedonia	0.750955
North Maluku	0.563445
North Somerset	0.858649

North Sulawesi	0.652615
North Sumatra	0.669499
North Tyneside	0.835248
North Yorkshire	0.855521
Northamptonshire	0.839033
Northern Cape	0.665813
Northern Ireland	0.84153
Northern Mariana Islands	0.777505
Northern Samar	0.523968
Northumberland	0.822334
North-West	0.654616
Nottingham	0.858455
Nottinghamshire	0.822433
Nueva Ecija	0.650592
Nueva Vizcaya	0.61661
Nuevo LeÃ³n	0.712153
Nyamira	0.593029
Nyandarua	0.57754
Nyeri	0.579363
Oaxaca	0.588389
Occidental Mindoro	0.460289
Ohio	0.851227
Okayama	0.86235
Okinawa	0.82164
Oklahoma	0.828145
Oldham	0.796705
Oman	0.773801
Oregon	0.87019
Oriental Mindoro	0.605552
Oromia	0.337961
Oslo	0.947286
Oxfordshire	0.899135
Palau	0.75459
Palawan	0.527211
Palestine	0.629202
Pampanga	0.697271
Panama	0.70666
Pangasinan	0.666136
Papua	0.646554
Papua New Guinea	0.418098
ParÃ¡	0.577314
ParaÃ-ba	0.557922
Paraguay	0.650488
ParanÃ¡	0.669861
Pennsylvania	0.87395
Pernambuco	0.583215
Peru	0.662036

Peterborough	0.837008
Piaui	0.520292
Piemonte	0.806813
Plymouth	0.842177
Poole	0.862991
Portsmouth	0.864778
Portugal	0.745395
Provincia autonoma di Bolzano	0.838907
Provincia autonoma di Trento	0.829532
Puebla	0.622885
Puerto Rico	0.824544
Puglia	0.763947
Punjab	0.520053
Qatar	0.846704
Qazvin	0.687516
Qom	0.694134
QuerÃ©taro	0.684048
Quezon	0.630169
Quintana Roo	0.682591
Quirino	0.575885
Reading	0.905272
Redbridge	0.849278
Redcar and Cleveland	0.79636
Republic of Korea	0.887196
Republic of Moldova	0.732393
Rhode Island	0.884284
Riau	0.724215
Riau Islands	0.749803
Richmond upon Thames	0.932022
Rio de Janeiro	0.710471
Rio Grande do Norte	0.585328
Rio Grande do Sul	0.689723
Rizal	0.710524
Rochdale	0.800322
Rogaland	0.916765
Romania	0.766321
Romblon	0.527573
RondÃ´nia	0.61851
Roraima	0.609883
Rotherham	0.803331
Rutland	0.852196
Rwanda	0.43614
SÃ£o Paulo	0.711183
Saga	0.835873
Saint Kitts and Nevis	0.756333
Saint Lucia	0.672602
Saint Vincent and the Grenadines	0.640887

Saitama	0.856554
Salford	0.837393
Samar (Western Samar)	0.527591
Samburu	0.371476
Samoa	0.59234
San Luis PotosÃ-	0.64758
San Marino	0.887884
Sandwell	0.793669
Santa Catarina	0.69484
Sao Tome and Principe	0.503306
Sarangani	0.582419
Sardegna	0.772644
Saudi Arabia	0.814516
Scotland	0.853887
Sefton	0.825438
Semnan	0.724002
Senegal	0.409005
Serbia	0.792213
Sergipe	0.59059
Seychelles	0.727579
Sheffield	0.854247
Shiga	0.874378
Shimane	0.838874
Shizuoka	0.865721
Shropshire	0.84238
Siaya	0.484422
Sicilia	0.762559
Sierra Leone	0.359009
Sinaloa	0.678348
Sindh	0.513737
Singapore	0.856235
Siquijor	0.600945
Sistan and Baluchistan	0.549869
Slough	0.877374
Slovakia	0.808329
Slovenia	0.842633
Solihull	0.871567
Solomon Islands	0.429542
Somali	0.270148
Somalia	0.077434
Somerset	0.842821
Sonora	0.709904
Sorsogon	0.600776
South Carolina	0.838586
South Cotabato	0.632537
South Dakota	0.856264
South Gloucestershire	0.884119

South Kalimantan	0.622222
South Khorasan	0.653365
South Sudan	0.278378
South Sulawesi	0.622994
South Sumatra	0.646421
South Tyneside	0.79949
Southampton	0.860211
Southeast Sulawesi	0.618325
Southend-on-Sea	0.825019
Southern Leyte	0.601491
Southern Nations, Nationalities, and Peoples	0.357248
Southwark	0.919165
Spain	0.769483
Sri Lanka	0.701372
St Helens	0.810914
Staffordshire	0.828182
Stockholm	0.916765
Stockport	0.861015
Stockton-on-Tees	0.829518
Stoke-on-Trent	0.796442
Sudan	0.542748
Suffolk	0.840389
Sultan Kudarat	0.519757
Sulu	0.483999
Sunderland	0.817933
Surigao Del Norte	0.627546
Surigao Del Sur	0.587487
Suriname	0.641163
Surrey	0.904484
Sutton	0.85753
Sweden except Stockholm	0.875759
Swindon	0.866702
Switzerland	0.933532
Syrian Arab Republic	0.622856
TÃ¼rkiye	0.713246
Tabasco	0.649851
Taita Taveta	0.542579
Taiwan (Province of China)	0.87514
Tajikistan	0.536613
TÅk yÅ	0.929043
Tamaulipas	0.682912
Tameside	0.798558
Tana River	0.389359
Tarlac	0.65076
Tawi-Tawi	0.535621
Tehran	0.776103
Telford and Wrekin	0.826094

Tennessee	0.831969
Texas	0.836777
Thailand	0.682657
Tharaka Nithi	0.528625
Thurrock	0.81863
Tigray	0.384003
Timor-Leste	0.450689
Tlaxcala	0.648989
Tocantins	0.601403
Tochigi	0.861256
Togo	0.410016
Tokelau	0.687018
Tokushima	0.857298
Tonga	0.629101
Torbay	0.81246
Toscana	0.811485
Tottori	0.836091
Tower Hamlets	0.903701
Toyama	0.865454
TrÃ¸ndelag	0.916774
Trafford	0.896679
Trans Nzoia	0.549785
Trinidad and Tobago	0.769401
Troms og Finnmark	0.904454
Tunisia	0.681701
Turkana	0.368386
Turkmenistan	0.68304
Tuvalu	0.578627
Uasin Gishu	0.567923
Uganda	0.426554
Umbria	0.799092
United Arab Emirates	0.84974
United Republic of Tanzania	0.448566
United States Virgin Islands	0.822988
Uruguay	0.721713
Utah	0.854829
Uzbekistan	0.664965
Valle d'Aosta	0.812706
Vanuatu	0.472796
Veneto	0.808197
Venezuela (Bolivarian Republic of)	0.5966
Veracruz de Ignacio de la Llave	0.627367
Vermont	0.891522
Vestfold og Telemark	0.907352
Vestland	0.917642
Viet Nam	0.621621
Vihiga	0.527055

Viken	0.914602
Virginia	0.881907
Wajir	0.258714
Wakayama	0.84733
Wakefield	0.804507
Wales	0.833275
Walsall	0.790655
Waltham Forest	0.840039
Wandsworth	0.924121
Warrington	0.878129
Warwickshire	0.865694
Washington	0.878014
West Azarbayejan	0.626919
West Berkshire	0.897125
West Java	0.644279
West Kalimantan	0.587439
West Nusa Tenggara	0.587663
West Papua	0.676555
West Pokot	0.44769
West Sulawesi	0.576459
West Sumatra	0.667845
West Sussex	0.863886
West Virginia	0.820334
Western Cape	0.719732
Westminster	0.93701
Wigan	0.805483
Wiltshire	0.859021
Windsor and Maidenhead	0.915543
Wirral	0.818112
Wisconsin	0.873096
Wokingham	0.910822
Wolverhampton	0.810888
Worcestershire	0.842681
Wyoming	0.863143
Yamagata	0.838535
Yamaguchi	0.856636
Yamanashi	0.858174
Yazd	0.713638
Yemen	0.45354
Yogyakarta	0.67683
York	0.88811
YucatÃ¡n	0.654459
Zacatecas	0.636671
Zambales	0.654254
Zambia	0.51023
Zamboanga Del Norte	0.538032
Zamboanga Del Sur	0.630995

Zamboanga Sibugay	0.549421
Zanjan	0.661464
Zimbabwe	0.475577

12. Reference

1. Murray CJL. The Global Burden of Disease Study at 30 years. *Nature medicine* 2022; **28**(10): 2019-26.
2. Global burden of 288 causes of death and life expectancy decomposition in 204 countries and territories and 811 subnational locations, 1990-2021: a systematic analysis for the Global Burden of Disease Study 2021. *Lancet (London, England)* 2024; **403**(10440): 2100-32.
3. Global incidence, prevalence, years lived with disability (YLDs), disability-adjusted life-years (DALYs), and healthy life expectancy (HALE) for 371 diseases and injuries in 204 countries and territories and 811 subnational locations, 1990-2021: a systematic analysis for the Global Burden of Disease Study 2021. *Lancet (London, England)* 2024; **403**(10440): 2133-61.
4. Global age-sex-specific mortality, life expectancy, and population estimates in 204 countries and territories and 811 subnational locations, 1950-2021, and the impact of the COVID-19 pandemic: a comprehensive demographic analysis for the Global Burden of Disease Study 2021. *Lancet (London, England)* 2024; **403**(10440): 1989-2056.
5. Vos T, Flaxman AD, Naghavi M, et al. Years lived with disability (YLDs) for 1160 sequelae of 289 diseases and injuries 1990-2010: a systematic analysis for the Global Burden of Disease Study 2010. *Lancet (London, England)* 2012; **380**(9859): 2163-96.
6. Viitanen M, Winblad B, Asplund K. Autopsy-verified causes of death after stroke. *Acta medica Scandinavica* 1987; **222**(5): 401-8.
7. Brønnum-Hansen H, Davidsen M, Thorvaldsen P. Long-term survival and causes of death after stroke. *Stroke* 2001; **32**(9): 2131-6.
8. Global, regional, and national comparative risk assessment of 84 behavioural, environmental and occupational, and metabolic risks or clusters of risks for 195 countries and territories, 1990-2017: a systematic analysis for the Global Burden of Disease Study 2017. *Lancet (London, England)* 2018; **392**(10159): 1923-94.
9. Global burden and strength of evidence for 88 risk factors in 204 countries and 811 subnational locations, 1990-2021: a systematic analysis for the Global Burden of Disease Study 2021. *Lancet (London, England)* 2024; **403**(10440): 2162-203.
10. Organization WH. Health Inequality Monitor. *World Health Organization* 2024; **https://**www.who.int/data/inequality-monitor.
11. Organization WH. Handbook on health inequality monitoring: with a special focus on low- and middle-income countries. *World Health Organization* 2013.
12. Hosseinpoor AR, Schlotheuber A, Nambiar D. Health Equity Assessment Toolkit Plus (HEAT Plus): software for exploring and comparing health inequalities using uploaded datasets. 2018; **11**(sup1): 1440783.
13. Hosseinpoor AR, Bergen N, Kirkby K, Schlotheuber A. Strengthening and expanding health inequality monitoring for the advancement of health equity: a review of WHO resources and

contributions. *International journal for equity in health* 2023; **22**(1): 49.
14. Riebler A, Held L. Projecting the future burden of cancer: Bayesian age-period-cohort analysis with integrated nested Laplace approximations. *Biometrical journal Biometrische Zeitschrift* 2017; **59**(3): 531-49.
15. Fosse E. Bayesian age–period–cohort models: Age, Period and Cohort Effects; 2020.
16. Thiago, G., Martins, et al. Bayesian computing with INLA: New features. *Computational Statistics & Data Analysis* 2013.
17. Lindgren F, Rue H. Bayesian Spatial and Spatio-temporal Modelling with R-INLA. *Journal of statistical software* 2015; **63**.
18. Global burden of 87 risk factors in 204 countries and territories, 1990-2019: a systematic analysis for the Global Burden of Disease Study 2019. *Lancet (London, England)* 2020; **396**(10258): 1223-49.
19. Vasudevan S, Ramos F, Nettleton E, Durrant ‐ Whyte H. Gaussian process modeling of large ‐ scale terrain. *Journal of Field Robotics* 2009; **26**(10): 812-40.
20. CE R. Gaussian Processes for Machine Learning. *Boston, MA: The MIT Press* 2005.

Additional Results in Tables and Figures

eTable 1. Age standardised mortality of ischemic stroke in people aged ≥20 years and their AAPC at global and regional level, 1990-2021

	No in 1990 (000s)	Age standardised rate in 1990 (per 100 000)	No in 2021 (000s)	Age standardised rate in 2021 (per 100 000)	AAPC (95% CI)	P value
Global	231 (210 to 249)	116 (104 to 125)	359 (317 to 392)	70 (62 to 77)	-1.60 (-1.81 to -1.39)	<0.001
Sex:						
Female	131 (117 to 140)	111 (98 to 119)	181 (154 to 202)	61 (52 to 68)	-1.88 (-2.10 to -1.66)	<0.001
Male	100 (91 to 111)	121 (109 to 133)	178 (158 to 198)	81 (72 to 90)	-1.28 (-1.46 to -1.09)	<0.001
Age group (years):						
20-24	1.6 (1.4 to 1.7)	0.32 (0.28 to 0.35)	1.4 (1.2 to 1.7)	0.24 (0.21 to 0.28)	-0.86 (-1.08 to -0.63)	<0.001
25-29	2.1 (1.9 to 2.4)	0.48 (0.43 to 0.54)	2.1 (1.8 to 2.4)	0.35 (0.3 to 0.41)	-1.08 (-1.33 to -0.84)	<0.001
30-34	3.4 (3.1 to 3.8)	0.89 (0.81 to 0.99)	4.1 (3.6 to 4.6)	0.67 (0.6 to 0.77)	-0.89 (-1.18 to -0.60)	<0.001
35-39	5.9 (5.4 to 6.6)	1.7 (1.5 to 1.9)	6.9 (6.1 to 7.8)	1.2 (1.1 to 1.4)	-0.94 (-1.06 to -0.82)	<0.001
40-44	10 (9 to 11)	3.4 (3.1 to 3.8)	13 (11 to 14)	2.5 (2.3 to 2.9)	-0.96 (-1.15 to -0.77)	<0.001
45-49	18 (16 to 20)	7.8 (7 to 8.7)	26 (23 to 29)	5.5 (4.9 to 6.1)	-1.12 (-1.59 to -0.65)	<0.001
50-54	40 (36 to 44)	19 (17 to 21)	53 (47 to 59)	12 (11 to 13)	-1.44 (-1.64 to -1.24)	<0.001
55-59	67 (601 to 74)	36 (33 to 40)	91 (81 to 101)	23 (21 to 26)	-1.43 (-1.83 to -1.03)	<0.001
60-64	152 (141 to 168)	95 (88 to 104)	188 (173 to 206)	59 (54 to 64)	-1.50 (-1.73 to -1.28)	<0.001
65-69	210 (195 to 230)	170 (158 to 186)	304 (278 to 334)	110 (101 to 121)	-1.33 (-1.57 to -1.09)	<0.001
70-74	327 (305 to 354)	386 (361 to 418)	503 (464 to 545)	244 (225 to 265)	-1.44 (-1.69 to -1.19)	<0.001
75-79	451 (422 to 480)	733 (686 to 779)	558 (511 to 606)	423 (387 to 460)	-1.78 (-1.92 to -1.65)	<0.001

80-84	490 (449 to 519)	1385 (1270 to 1466)	724 (643 to 780)	827 (734 to 890)	-1.63 (-1.91 to -1.36)	<0.001
85-89	346 (303 to 367)	2290 (2005 to 2431)	625 (537 to 681)	1367 (1174 to 1490)	-1.64 (-1.80 to -1.49)	<0.001
90-94	143 (121 to 155)	3348 (2818 to 3612)	352 (286 to 389)	1967 (1598 to 2174)	-1.69 (-1.95 to -1.42)	<0.001
≥95	44 (34 to 48)	4300 (3353 to 4749)	138 (102 to 157)	2530 (1872 to 2879)	-1.69 (-1.91 to -1.46)	<0.001
SDI level:						
High	60 (53 to 63)	85 (76 to 90)	51 (42 to 56)	31 (26 to 34)	-3.26 (-3.49 to -3.03)	<0.001
High-middle	89 (82 to 93)	178 (162 to 187)	115 (101 to 127)	95 (83 to 105)	-1.98 (-2.29 to -1.66)	<0.001
Middle	48 (43 to 55)	105 (93 to 119)	117 (102 to 131)	82 (71 to 91)	-0.85 (-1.09 to -0.61)	<0.001
Low-middle	26 (22 to 30)	93 (80 to 109)	58 (51 to 67)	81 (71 to 92)	-0.44 (-0.55 to -0.32)	<0.001
Low	9 (7 to 11)	90 (74 to 115)	17 (14 to 22)	78 (65 to 97)	-0.43 (-0.56 to -0.30)	<0.001

Note: Estimates are for individuals aged over 20 years. AAPCs=average annual percent changes. CI=confidence interval. P=P value for the significant test of AAPCs. SDI=Socio-demographic Index. Numbers in parentheses are 95% uncertainty intervals (Cases and age-standardized rate) and 95% confidence interval (AAPCs).

eTable 2. Age standardised DALYs of ischemic stroke in people aged ≥20 years and their AAPC at global and regional level, 1990-2021

	No in 1990 (0000s)	Age standardised rate in 1990 (per 100 000)	No in 2021 (0000s)	Age standardised rate in 2021 (per 100 000)	AAPC (95% CI)	P value
Global	4535 (4167 to 4910)	2020 (1846 to 2182)	6983 (6260 to 7617)	1317 (1178 to 1437)	-1.36 (-1.52 to -1.20)	<0.001
Sex:						
Female	2386 (2161 to 2581)	1881 (1695 to 2034)	3309 (2877 to 3698)	1130 (982 to 1263)	-1.62 (-1.82 to -1.43)	<0.001
Male	2149 (1944 to 2388)	2176 (1970 to 2399)	3674 (3294 to 4065)	1536 (1376 to 1696)	-1.11 (-1.26 to -0.96)	<0.001
Age group (years):						
20-24	23 (19 to 27)	46 (38 to 55)	22 (18 to 27)	37 (31 to 45)	-0.69 (-0.75 to -0.64)	<0.001
25-29	28 (23 to 33)	63 (52 to 75)	30 (24 to 36)	50 (41 to 60)	-0.73 (-0.82 to -0.64)	<0.001
30-34	36 (30 to 42)	94 (79 to 109)	47 (39 to 55)	77 (65 to 91)	-0.61 (-0.78 to -0.44)	<0.001
35-39	51 (44 to 58)	144 (125 to 164)	65 (56 to 76)	117 (100 to 135)	-0.66 (-0.73 to -0.58)	<0.001
40-44	68 (61 to 77)	239 (212 to 269)	96 (84 to 109)	192 (167 to 217)	-0.68 (-0.81 to -0.54)	<0.001
45-49	103 (93 to 114)	443 (402 to 492)	162 (142 to 181)	341 (300 to 382)	-0.82 (-1.22 to -0.42)	<0.001
50-54	188 (173 to 207)	887 (813 to 973)	275 (244 to 305)	619 (548 to 686)	-1.15 (-1.31 to -0.99)	<0.001
55-59	272 (249 to 301)	1468 (1347 to 1628)	404 (361 to 446)	1021 (911 to 1128)	-1.13 (-1.33 to -0.94)	<0.001
60-64	503 (467 to 548)	3133 (2910 to 3413)	660 (601 to 719)	2062 (1879 to 2245)	-1.31 (-1.49 to -1.14)	<0.001
65-69	579 (538 to 635)	4687 (4355 to 5135)	890 (810 to 968)	3227 (2937 to 3509)	-1.13 (-1.32 to -0.93)	<0.001
70-74	718 (672 to 779)	8486 (7941 to 9197)	1163 (1072 to 1256)	5652 (5208 to 6102)	-1.29 (-1.51 to -1.06)	<0.001
75-79	782 (736 to 830)	12698 (11962 to 13487)	1026 (9397 to 1108)	7782 (7125 to 8404)	-1.59 (-1.71 to -1.47)	<0.001
80-84	655 (601 to 692)	18502 (16985 to 19564)	1013 (910 to 1093)	11566 (10395 to 12475)	-1.49 (-1.80 to -1.18)	<0.001
85-89	363 (320 to 386)	23996 (21189 to 25526)	681 (594 to 741)	14895 (12996 to 16201)	-1.53 (-1.68 to -1.39)	<0.001

90-94	129 (110 to 140)	30170 (25599 to 32556)	329 (272 to 362)	18374 (15207 to 20248)	-1.58 (-1.84 to -1.32)	<0.001
≥95	37 (29 to 41)	36417 (28864 to 40144)	120 (92 to 136)	22001 (16900 to 24937)	-1.55 (-1.72 to -1.38)	<0.001
SDI level:						
High	1018 (925 to 1086)	1440 (1302 to 1541)	895 (766 to 997)	620 (534 to 694)	-2.70 (-2.87 to -2.53)	<0.001
High-middle	1676 (1555 to 1779)	2974 (2742 to 3156)	2101 (1869 to 2319)	1700 (1508 to 1878)	-1.76 (-2.07 to -1.45)	<0.001
Middle	1070 (956 to 1211)	1920 (1711 to 2166)	2378 (2101 to 2641)	1515 (1335 to 1682)	-0.80 (-0.99 to -0.60)	<0.001
Low-middle	565 (491 to 660)	1713 (1489 to 1990)	1214 (1069 to 1397)	1483 (1305 to 1697)	-0.43 (-0.56 to -0.30)	<0.001
Low	199 (165 to 254)	1681 (1395 to 2125)	389 (328 to 483)	1434 (1209 to 1766)	-0.49 (-0.58 to -0.40)	<0.001

Note: Estimates are for individuals aged over 20 years. AAPCs=average annual percent changes. CI=confidence interval. DALYs=disability-adjusted life years. P=P value for the significant test of AAPCs. SDI=Socio-demographic Index. Numbers in parentheses are 95% uncertainty intervals (Cases and age-standardized rate) and 95% confidence interval (AAPCs).

eTable 3. Age standardized prevalence of ischemic stroke in people aged ≥20 years and their AAPCs at regional levels, 1990-2021

Regions	No in 1990 (000s)	Age standardised rate in 1990 (per 100 000)	No in 2021 (000s)	Age standardised rate in 2021 (per 100 000)	AAPC (95% CI)	P value
Asia						
Central Asia	533 (491 to 576)	1724 (1585 to 1869)	850 (794 to 909)	1580 (1471 to 1698)	-0.27 (-0.29 to -0.25)	<0.001
East Asia	6561 (5446 to 7779)	1185 (981 to 1412)	21292 (18124 to 24751)	1581 (1344 to 1839)	0.89 (0.85 to 0.94)	<0.001
South Asia	3225 (2634 to 3851)	810 (657 to 975)	7544 (6311 to 8856)	767 (639 to 905)	-0.17 (-0.20 to -0.14)	<0.001
Southeast Asia	2417 (2101 to 2751)	1424 (1236 to 1625)	5975 (5287 to 6698)	1427 (1261 to 1602)	0 (0 to 0.01)	0.108
High-income Asia Pacific	2031 (1781 to 2295)	1607 (1407 to 1819)	3281 (2917 to 3678)	1205 (1070 to 1350)	-0.92 (-0.95 to -0.89)	<0.001
Europe						
Central Europe	1396 (1253 to 1551)	1492 (1335 to 1662)	1620 (1471 to 1777)	1205 (1095 to 1322)	-0.68 (-0.70 to -0.65)	<0.001
Eastern Europe	2830 (2387 to 3299)	1598 (1346 to 1865)	3025 (2589 to 3490)	1431 (1224 to 1653)	-0.35 (-0.36 to -0.34)	<0.001
Western Europe	4507 (4104 to 4934)	1249 (1134 to 1371)	5498 (5128 to 5885)	968 (900 to 1039)	-0.80 (-0.83 to -0.77)	<0.001
Africa						
Central Sub-Saharan Africa	246 (225 to 268)	1724 (1566 to 1888)	554 (513 to 597)	1545 (1424 to 1671)	-0.35 (-0.37 to -0.34)	<0.001
Eastern Sub-Saharan Africa	798 (713 to 889)	1652 (1472 to 1845)	1731 (1575 to 1900)	1538 (1395 to 1693)	-0.23 (-0.24 to -0.23)	<0.001
Southern Sub-Saharan Africa	343 (290 to 401)	1975 (1667 to 2311)	619 (535 to 711)	1741 (1501 to 2006)	-0.40 (-0.43 to -0.38)	<0.001
Western Sub-Saharan Africa	1006 (892 to 1130)	1698 (1498 to 1916)	2231 (2013 to 2464)	1603 (1441 to 1777)	-0.18 (-0.19 to -0.18)	<0.001
North Africa and Middle East	1671 (1511 to 1839)	1382 (1243 to 1528)	4165 (3844 to 4499)	1325 (1218 to 1438)	-0.13 (-0.15 to -0.12)	<0.001
North America						
Caribbean	169 (156 to 183)	991 (914 to 1077)	318 (296 to 341)	944 (878 to 1014)	-0.16 (-0.17 to -0.15)	<0.001
High-income North America	3302 (2819 to 3813)	1501 (1280 to 1736)	5604 (4929 to 6309)	1449 (1271 to 1637)	-0.15 (-0.25 to -0.05)	0.003

South America						
Andean Latin America	129 (118 to 139)	929 (855 to 1006)	302 (281 to 324)	792 (736 to 850)	-0.52 (-0.53 to -0.51)	<0.001
Central Latin America	625 (551 to 703)	1113 (985 to 1248)	1342 (1198 to 1498)	847 (757 to 945)	-0.88 (-0.90 to -0.86)	<0.001
Southern Latin America	390 (361 to 421)	1336 (1235 to 1445)	538 (502 to 575)	992 (926 to 1062)	-0.95 (-0.98 to -0.92)	<0.001
Tropical Latin America	794 (660 to 939)	1321 (1099 to 1564)	1519 (1287 to 1770)	940 (796 to 1096)	-1.09 (-1.12 to -1.06)	<0.001
Oceania						
Oceania	23 (22 to 25)	1230 (1124 to 1339)	54 (51 to 58)	1127 (1046 to 1210)	-0.28 (-0.29 to -0.28)	<0.001
Australasia	173 (162 to 185)	1167 (1092 to 1250)	294 (276 to 313)	900 (843 to 959)	-0.83 (-0.87 to -0.80)	<0.001

Note: Estimates are for individuals aged over 20 years. AAPCs=average annual percent changes. CI=confidence interval. P=P value for the significant test of AAPCs. SDI=Socio-demographic Index. Numbers in parentheses are 95% uncertainty intervals (Cases and age-standardized rate) and 95% confidence interval (AAPCs).

eTable 4. Age standardized prevalence, mortality and DALYs of ischemic stroke in people aged ≥20 years at regional levels by sex, 2021

Regions	Age-standardized rate in 2021 (per 100,000)					
	Male			Female		
	Prevalence	Mortality	DALYs	Prevalence	Mortality	DALYs
Asia						
Central Asia	1551 (1431 to 1685)	132 (118 to 145)	2518 (2256 to 2776)	1608 (1489 to 1732)	100 (87 to 110)	1869 (1653 to 2074)
East Asia	1678 (1436 to 1942)	136 (110 to 166)	2361 (1916 to 2858)	1489 (1246 to 1755)	76 (59 to 94)	1438 (1152 to 1745)
South Asia	863 (716 to 1021)	66 (53 to 87)	1193 (969 to 1602)	680 (568 to 802)	55 (44 to 70)	994 (795 to 1262)
Southeast Asia	1479 (1304 to 1668)	123 (97 to 146)	2334 (1854 to 2777)	1393 (1228 to 1567)	96 (78 to 114)	1711 (1416 to 2036)
High-income Asia Pacific	1577 (1397 to 1767)	33 (29 to 37)	716 (616 to 814)	880 (778 to 992)	19 (13 to 22)	373 (287 to 444)
Europe						
Central Europe	1383 (1251 to 1525)	116 (105 to 126)	2050 (1860 to 2225)	1083 (979 to 1192)	94 (83 to 103)	1495 (1323 to 1633)
Eastern Europe	1566 (1321 to 1829)	162 (143 to 178)	3081 (2753 to 3406)	1357 (1166 to 1563)	131 (114 to 146)	2148 (1887 to 2385)
Western Europe	1142 (1051 to 1236)	29 (25 to 31)	537 (472 to 596)	821 (753 to 890)	24 (19 to 28)	408 (333 to 464)
Africa						
Central Sub-Saharan Africa	1275 (1160 to 1396)	93 (65 to 134)	1663 (1185 to 2369)	1744 (1604 to 1892)	95 (60 to 143)	1708 (1151 to 2483)
Eastern Sub-Saharan Africa	1387 (1247 to 1538)	76 (60 to 95)	1407 (1136 to 1760)	1665 (1507 to 1835)	72 (51 to 96)	1345 (1024 to 1736)
Southern Sub-Saharan Africa	1434 (1221 to 1679)	92 (81 to 104)	1679 (1482 to 1890)	1944 (1673 to 2231)	96 (81 to 108)	1674 (1451 to 1897)
Western Sub-Saharan Africa	1502 (1343 to 1671)	131 (108 to 159)	2428 (2001 to 2946)	1694 (1519 to 1881)	88 (64 to 116)	1557 (1163 to 2031)
North Africa and Middle East	1418 (1298 to 1545)	113 (95 to 131)	2070 (1760 to 2398)	1236 (1129 to 1347)	121 (99 to 142)	2100 (1741 to 2475)
North America						
Caribbean	948 (874 to 1025)	59 (50 to 70)	1071 (905 to 1265)	947 (877 to 1022)	56 (47 to 66)	981 (817 to 1187)

High-income North America	1576 (1383 to 1782)	26 (22 to 28)	565 (489 to 637)	1342 (1170 to 1524)	27 (21 to 30)	530 (438 to 609)
South America						
Andean Latin America	787 (725 to 850)	30 (22 to 38)	536 (411 to 681)	798 (736 to 864)	27 (20 to 34)	469 (373 to 596)
Central Latin America	824 (731 to 923)	32 (28 to 37)	583 (512 to 659)	872 (778 to 972)	27 (23 to 31)	483 (419 to 548)
Southern Latin America	1161 (1075 to 1249)	39 (34 to 44)	743 (657 to 834)	861 (794 to 930)	29 (24 to 33)	518 (438 to 592)
Tropical Latin America	1100 (924 to 1292)	57 (51 to 62)	1027 (934 to 1103)	819 (691 to 958)	39 (32 to 43)	662 (569 to 727)
Oceania						
Oceania	1121 (1034 to 1211)	54 (38 to 78)	1064 (787 to 1482)	1133 (1046 to 1222)	74 (54 to 101)	1384 (1046 to 1853)
Australasia	1123 (1049 to 1203)	22 (19 to 25)	430 (362 to 500)	703 (647 to 762)	22 (17 to 25)	354 (281 to 418)

Note: Estimates are for individuals aged over 20 years. CI=confidence interval. Numbers in parentheses are 95% uncertainty intervals (Cases and age-standardized rate).

eTable 5. Age standardized mortality of ischemic stroke in people aged ≥20 years and their AAPCs at regional levels, 1990-2021

Regions	No in 1990 (000s)	Age standardised rate in 1990 (per 100 000)	No in 2021 (000s)	Age standardised rate in 2021 (per 100 000)	AAPC (95% CI)	P value
Asia						
Central Asia	33 (30 to 35)	130 (117 to 139)	45 (40 to 49)	113 (100 to 124)	-0.32 (-1.02 to 0.38)	0.373
East Asia	442 (374 to 525)	118 (100 to 139)	1202 (987 to 1411)	100 (82 to 117)	-0.52 (-0.78 to -0.26)	<0.001
South Asia	172 (138 to 220)	69 (56 to 86)	441 (373 to 552)	60 (51 to 74)	-0.38 (-0.89 to 0.13)	0.14
Southeast Asia	131 (112 to 150)	115 (98 to 132)	341 (283 to 396)	108 (90 to 124)	-0.20 (-0.28 to -0.13)	<0.001
High-income Asia Pacific	106 (93 to 114)	100 (86 to 108)	113 (86 to 129)	25 (20 to 28)	-4.41 (-4.85 to -3.97)	<0.001
Europe						
Central Europe	181 (171 to 188)	224 (208 to 233)	160 (143 to 173)	104 (93 to 113)	-2.49 (-2.71 to -2.27)	<0.001
Eastern Europe	405 (382 to 418)	267 (248 to 276)	329 (293 to 358)	144 (128 to 157)	-1.96 (-2.51 to -1.40)	<0.001
Western Europe	380 (338 to 402)	100 (88 to 106)	216 (175 to 238)	27 (22 to 29)	-4.19 (-4.35 to -4.02)	<0.001
Africa						
Central Sub-Saharan Africa	8 (6 to 11)	102 (76 to 135)	19 (13 to 26)	95 (67 to 131)	-0.25 (-0.33 to -0.17)	<0.001
Eastern Sub-Saharan Africa	25 (20 to 32)	82 (65 to 105)	52 (42 to 63)	73 (59 to 90)	-0.36 (-0.42 to -0.30)	<0.001
Southern Sub-Saharan Africa	10 (8 to 11)	75 (60 to 87)	25 (22 to 28)	96 (84 to 106)	0.82 (0.32 to 1.31)	0.001
Western Sub-Saharan Africa	49 (37 to 64)	121 (94 to 157)	92 (75 to 111)	108 (89 to 130)	-0.36 (-0.43 to -0.29)	<0.001
North Africa and Middle East	129 (109 to 149)	168 (142 to 194)	252 (214 to 289)	117 (99 to 133)	-1.15 (-1.33 to -0.97)	<0.001
North America						
Caribbean	12 (11 to 13)	84 (75 to 91)	20 (17 to 23)	58 (49 to 66)	-1.15 (-1.7 to -0.60)	<0.001

High-income North America	110 (95 to 118)	47 (40 to 50)	126 (102 to 139)	27 (22 to 29)	-1.77 (-2.09 to -1.45)	<0.001
South America						
Andean Latin America	5.4 (4.6 to 6.4)	50 (42 to 59)	10 (8 to 12)	28 (22 to 35)	-1.83 (-2.38 to -1.28)	<0.001
Central Latin America	24 (22 to 25)	59 (55 to 62)	43 (37 to 48)	30 (26 to 33)	-2.25 (-2.57 to -1.93)	<0.001
Southern Latin America	23 (21 to 25)	91 (81 to 99)	20 (17 to 22)	34 (29 to 37)	-3.19 (-3.53 to -2.86)	<0.001
Tropical Latin America	55 (51 to 58)	127 (114 to 135)	72 (62 to 77)	47 (41 to 51)	-3.11 (-3.31 to -2.92)	<0.001
Oceania						
Oceania	0.8 (0.6 to 1.1)	77 (59 to 102)	1.8 (1.4 to 2.5)	64 (50 to 86)	-0.59 (-0.64 to -0.54)	<0.001
Australasia	10 (9 to 11)	73 (63 to 80)	9 (8 to 11)	22 (18 to 25)	-3.78 (-4.01 to -3.56)	<0.001

Note: Estimates are for individuals aged over 20 years. AAPCs=average annual percent changes. CI=confidence interval. P=P value for the significant test of AAPCs. SDI=Socio-demographic Index. Numbers in parentheses are 95% uncertainty intervals (Cases and age-standardized rate) and 95% confidence interval (AAPCs).

eTable 6. Age standardized DALYs of ischemic stroke in people aged ≥20 years and their AAPCs at regional levels, 1990-2021

Regions	No in 1990 (000s)	Age standardised rate in 1990 (per 100 000)	No in 2021 (000s)	Age standardised rate in 2021 (per 100 000)	AAPC (95% CI)	P value
Asia						
Central Asia	713 (651 to 768)	2567 (2339 to 2759)	981 (876 to 1083)	2143 (1917 to 2357)	-0.49 (-0.76 to -0.21)	0.001
East Asia	10139 (8626 to 11966)	2177 (1854 to 2556)	23967 (20100 to 27749)	1841 (1541 to 2130)	-0.54 (-0.72 to -0.36)	<0.001
South Asia	3884 (3163 to 4947)	1271 (1041 to 1600)	9110 (7732 to 11563)	1088 (927 to 1361)	-0.44 (-0.68 to -0.19)	0.001
Southeast Asia	2924 (2539 to 3322)	2129 (1844 to 2418)	7268 (6057 to 8424)	1997 (1677 to 2300)	-0.21 (-0.29 to -0.13)	<0.001
High-income Asia Pacific	1920 (1714 to 2085)	1653 (1460 to 1798)	1860 (1521 to 2127)	527 (436 to 607)	-3.66 (-3.93 to -3.38)	<0.001
Europe						
Central Europe	3197 (3020 to 3339)	3640 (3418 to 3808)	2591 (2340 to 2803)	1742 (1571 to 1889)	-2.39 (-2.56 to -2.22)	<0.001
Eastern Europe	7411 (7017 to 7696)	4474 (4208 to 4655)	5708 (5160 to 6200)	2534 (2286 to 2757)	-1.79 (-2.30 to -1.27)	<0.001
Western Europe	5836 (5294 to 6196)	1520 (1371 to 1619)	3302 (2790 to 3677)	469 (398 to 525)	-3.73 (-3.85 to -3.60)	<0.001
Africa						
Central Sub-Saharan Africa	205 (155 to 268)	1902 (1449 to 2470)	434 (321 to 585)	1698 (1253 to 2289)	-0.35 (-0.41 to -0.29)	<0.001
Eastern Sub-Saharan Africa	588 (475 to 740)	1544 (1251 to 1932)	1196 (992 to 1430)	1377 (1139 to 1643)	-0.37 (-0.44 to -0.30)	<0.001
Southern Sub-Saharan Africa	219 (183 to 252)	1443 (1200 to 1656)	518 (463 to 577)	1695 (1511 to 1881)	0.52 (0.07 to 0.98)	0.022
Western Sub-Saharan Africa	1060 (840 to 1378)	2216 (1759 to 2859)	2057 (1701 to 2470)	1967 (1639 to 2344)	-0.39 (-0.43 to -0.34)	<0.001
North Africa and Middle East	2779 (2385 to 3205)	3007 (2575 to 3456)	5311 (4540 to 6080)	2086 (1785 to 2374)	-1.14 (-1.34 to -0.94)	<0.001
North America						
Caribbean	218 (196 to 239)	1408 (1267 to 1543)	349 (299 to 405)	1025 (879 to 1190)	-0.98 (-1.38 to -0.58)	<0.001
High-income North America	1990 (1751 to 2196)	861 (755 to 953)	2369 (2003 to 2676)	549 (464 to 624)	-1.37 (-1.52 to -1.23)	<0.001

South America						
Andean Latin America	107 (90 to 124)	890 (757 to 1031)	181 (146 to 221)	501 (407 to 613)	-1.81 (-2.31 to -1.31)	<0.001
Central Latin America	470 (439 to 497)	1008 (940 to 1063)	797 (706 to 891)	529 (469 to 591)	-2.07 (-2.38 to -1.76)	<0.001
Southern Latin America	421 (380 to 459)	1536 (1379 to 1670)	351 (309 to 390)	616 (541 to 686)	-2.93 (-3.18 to -2.67)	<0.001
Tropical Latin America	1086 (1010 to 1142)	2163 (1990 to 2281)	1289 (1156 to 1387)	822 (735 to 886)	-3.00 (-3.32 to -2.67)	<0.001
Oceania						
Oceania	21 (16 to 27)	1455 (1132 to 1887)	45 (36 to 59)	1223 (971 to 1586)	-0.57 (-0.62 to -0.53)	<0.001
Australasia	162 (144 to 178)	1127 (994 to 1239)	149 (123 to 170)	392 (324 to 451)	-3.36 (-3.59 to -3.13)	<0.001

Note: Estimates are for individuals aged over 20 years. AAPCs=average annual percent changes. CI=confidence interval. P=P value for the significant test of AAPCs. SDI=Socio-demographic Index. Numbers in parentheses are 95% uncertainty intervals (Cases and age-standardized rate) and 95% confidence interval (AAPCs).

eTable 7. Age standardized prevalence, mortality and DALYs of ischemic stroke in people aged ≥20 years in 2021 and their AAPCs between 1990-2021 in 204 countries and territories

Country or territory	Age-standardized rate in 2021 (per 100,000)			AAPCs 1990-2021 (%/year)		
	Prevalence	Mortality	DALYs	Prevalence	Mortality	DALYs
Afghanistan	1258 (1141 to 1383)	172 (118 to 237)	3214 (2205 to 4500)	-0.10 (-0.13 to -0.08)	-0.27 (-0.32 to -0.21)	-0.28 (-0.36 to -0.20)
Albania	885 (810 to 962)	71 (51 to 96)	1095 (807 to 1444)	-0.34 (-0.37 to -0.31)	-0.67 (-1.31 to -0.03)	-0.95 (-1.47 to -0.43)
Algeria	1514 (1399 to 1636)	130 (95 to 168)	1987 (1468 to 2566)	-0.13 (-0.14 to -0.11)	-1.09 (-1.24 to -0.93)	-1.14 (-1.25 to -1.04)
American Samoa	1301 (1209 to 1398)	57 (42 to 74)	1104 (855 to 1399)	-0.52 (-0.53 to -0.51)	-0.87 (-1.02 to -0.72)	-0.83 (-0.95 to -0.71)
Andorra	736 (673 to 801)	22 (15 to 30)	376 (272 to 496)	-0.81 (-0.84 to -0.77)	-2.47 (-2.9 to -2.03)	-2.28 (-2.63 to -1.93)
Angola	1809 (1673 to 1952)	101 (72 to 136)	1848 (1368 to 2425)	-0.23 (-0.24 to -0.22)	-0.07 (-0.15 to 0)	-0.23 (-0.30 to -0.15)
Antigua and Barbuda	888 (819 to 961)	59 (52 to 67)	928 (813 to 1051)	-0.48 (-0.5 to -0.46)	-1.28 (-1.86 to -0.69)	-1.43 (-2.12 to -0.73)
Argentina	997 (928 to 1070)	30 (26 to 34)	585 (507 to 664)	-0.87 (-0.92 to -0.83)	-3.31 (-3.94 to -2.69)	-2.91 (-3.39 to -2.43)
Armenia	1285 (1199 to 1378)	81 (70 to 91)	1513 (1331 to 1699)	-0.49 (-0.53 to -0.45)	-1.00 (-2.12 to 0.13)	-1.03 (-1.93 to -0.13)
Australia	909 (857 to 961)	21 (17 to 24)	377 (308 to 438)	-0.85 (-0.89 to -0.80)	-3.96 (-4.24 to -3.68)	-3.52 (-3.73 to -3.31)
Austria	1335 (1248 to 1429)	21 (17 to 24)	457 (380 to 531)	0.53 (0.45 to 0.60)	-4.99 (-5.70 to -4.27)	-3.97 (-4.38 to -3.55)
Azerbaijan	1224 (1121 to 1331)	62 (47 to 80)	1224 (959 to 1544)	0.02 (0 to 0.03)	-0.44 (-1.05 to 0.17)	-0.57 (-0.96 to -0.17)
Bahamas	937 (868 to 1011)	46 (38 to 55)	775 (640 to 928)	-0.34 (-0.36 to -0.33)	-1.11 (-1.94 to -0.27)	-1.25 (-1.83 to -0.67)
Bahrain	811 (745 to 885)	91 (72 to 112)	1392 (1103 to 1723)	-0.61 (-0.62 to -0.59)	-1.75 (-2.44 to -1.05)	-1.95 (-2.5 to -1.40)
Bangladesh	933 (846 to 1025)	116 (85 to 155)	1825 (1339 to 2469)	-0.12 (-0.16 to -0.07)	-0.07 (-0.83 to 0.7)	-0.30 (-0.94 to 0.34)
Barbados	962 (888 to 1042)	70 (57 to 85)	1077 (869 to 1312)	-0.40 (-0.41 to -0.4)	-1.53 (-2.38 to -0.67)	-1.59 (-2.32 to -0.85)
Belarus	1540 (1415 to 1676)	118 (97 to 139)	2194 (1831 to 2585)	-0.28 (-0.35 to -0.22)	-1.08 (-1.58 to -0.58)	-1.21 (-1.68 to -0.74)
Belgium	857 (803 to 913)	24 (19 to 27)	429 (356 to 493)	-0.56 (-0.57 to -0.55)	-4.08 (-4.24 to -3.92)	-3.59 (-3.69 to -3.49)
Belize	761 (701 to 825)	42 (35 to 48)	685 (581 to 793)	-0.15 (-0.17 to -0.12)	-0.19 (-1.05 to 0.67)	-0.30 (-1.04 to 0.44)
Benin	1560 (1446 to 1681)	121 (90 to 159)	2135 (1607 to 2778)	-0.44 (-0.45 to -0.43)	-0.40 (-0.56 to -0.23)	-0.46 (-0.65 to -0.27)
Bermuda	842 (777 to 912)	32 (26 to 39)	532 (440 to 646)	-0.79 (-0.81 to -0.77)	-3.00 (-3.60 to -2.40)	-2.91 (-3.35 to -2.47)
Bhutan	833 (754 to 912)	60 (43 to 81)	1062 (766 to 1399)	-0.09 (-0.10 to -0.09)	-0.50 (-0.59 to -0.41)	-0.65 (-0.73 to -0.58)

Bolivia (Plurinational State of)	724 (659 to 793)	48 (32 to 70)	800 (533 to 1148)	-0.70 (-0.71 to -0.69)	-1.56 (-1.65 to -1.47)	-1.69 (-1.78 to -1.6)
Bosnia and Herzegovina	1727 (1598 to 1862)	143 (112 to 174)	2486 (1970 to 2984)	-0.08 (-0.09 to -0.07)	-0.97 (-1.22 to -0.71)	-1.09 (-1.41 to -0.77)
Botswana	2084 (1921 to 2257)	90 (66 to 121)	1686 (1297 to 2188)	0.03 (0.03 to 0.04)	-1.34 (-2.35 to -0.33)	-1.16 (-1.98 to -0.32)
Brazil	940 (794 to 1099)	47 (40 to 51)	819 (732 to 881)	-1.10 (-1.13 to -1.07)	-3.17 (-3.36 to -2.97)	-3.04 (-3.37 to -2.72)
Brunei Darussalam	1489 (1388 to 1593)	61 (47 to 76)	1080 (860 to 1316)	-1.67 (-1.76 to -1.58)	-2.34 (-2.76 to -1.92)	-2.45 (-2.98 to -1.92)
Bulgaria	1607 (1401 to 1823)	234 (205 to 265)	3774 (3278 to 4303)	-0.08 (-0.10 to -0.06)	-0.67 (-1.54 to 0.21)	-0.58 (-1.22 to 0.07)
Burkina Faso	1186 (1103 to 1275)	75 (53 to 104)	1361 (973 to 1873)	-0.28 (-0.29 to -0.26)	0.06 (-0.08 to 0.19)	-0.04 (-0.15 to 0.07)
Burundi	1485 (1359 to 1612)	83 (57 to 119)	1517 (1077 to 2110)	-0.95 (-0.98 to -0.93)	-1.54 (-1.73 to -1.35)	-1.65 (-1.84 to -1.47)
Cabo Verde	1551 (1445 to 1660)	105 (78 to 137)	1883 (1419 to 2424)	-0.01 (-0.01 to 0)	0.97 (0.54 to 1.40)	0.66 (0.26 to 1.06)
Cambodia	1106 (1010 to 1210)	119 (89 to 154)	1974 (1499 to 2525)	-0.01 (-0.02 to 0)	-0.04 (-0.11 to 0.02)	-0.28 (-0.35 to -0.22)
Cameroon	1560 (1444 to 1677)	113 (79 to 159)	2055 (1464 to 2862)	-0.02 (-0.04 to -0.01)	0.32 (0.20 to 0.45)	0.32 (0.17 to 0.46)
Canada	1297 (1242 to 1351)	19 (16 to 22)	421 (346 to 491)	-0.35 (-0.38 to -0.33)	-3.14 (-3.51 to -2.76)	-2.41 (-2.56 to -2.26)
Central African Republic	1497 (1361 to 1639)	113 (72 to 168)	2034 (1354 to 2952)	-0.28 (-0.29 to -0.27)	-0.29 (-0.38 to -0.19)	-0.36 (-0.43 to -0.29)
Chad	1592 (1473 to 1712)	124 (88 to 171)	2293 (1652 to 3141)	-0.16 (-0.18 to -0.15)	0.46 (0.32 to 0.6)	0.42 (0.26 to 0.58)
Chile	963 (893 to 1037)	35 (30 to 40)	620 (535 to 704)	-0.79 (-0.83 to -0.75)	-3.05 (-3.65 to -2.45)	-2.98 (-3.46 to -2.49)
China	1582 (1340 to 1848)	102 (84 to 120)	1865 (1555 to 2164)	0.96 (0.91 to 1.01)	-0.48 (-0.74 to -0.22)	-0.51 (-0.69 to -0.34)
Colombia	763 (711 to 822)	23 (18 to 28)	428 (353 to 508)	-1.34 (-1.39 to -1.29)	-3.19 (-3.74 to -2.63)	-3.00 (-3.41 to -2.59)
Comoros	1724 (1596 to 1858)	77 (53 to 106)	1426 (1029 to 1919)	-0.56 (-0.59 to -0.53)	-1.04 (-1.10 to -0.97)	-1.09 (-1.17 to -1.01)
Congo	1902 (1757 to 2053)	110 (79 to 147)	2008 (1478 to 2616)	-0.49 (-0.50 to -0.48)	-0.58 (-0.74 to -0.42)	-0.66 (-0.83 to -0.49)
Cook Islands	1398 (1305 to 1493)	37 (26 to 51)	817 (623 to 1046)	0.07 (0.05 to 0.08)	-1.96 (-2.04 to -1.89)	-1.54 (-1.60 to -1.49)
Costa Rica	825 (764 to 889)	25 (21 to 29)	432 (363 to 496)	-0.59 (-0.60 to -0.58)	-1.45 (-2.19 to -0.71)	-1.50 (-2.12 to -0.88)
Coted'Ivoire	1699 (1576 to 1825)	123 (89 to 165)	2290 (1659 to 3095)	-0.52 (-0.54 to -0.51)	-0.22 (-0.32 to -0.11)	-0.28 (-0.45 to -0.11)
Croatia	1155 (1092 to 1218)	76 (65 to 87)	1235 (1067 to 1404)	-0.51 (-0.53 to -0.49)	-3.46 (-3.69 to -3.24)	-3.39 (-3.60 to -3.17)
Cuba	882 (816 to 951)	54 (46 to 63)	923 (786 to 1067)	-0.28 (-0.30 to -0.26)	-0.81 (-0.94 to -0.68)	-0.67 (-0.78 to -0.55)
Cyprus	475 (417 to 545)	53 (40 to 68)	655 (498 to 827)	-1.92 (-2.00 to -1.84)	-4.12 (-4.92 to -3.32)	-4.21 (-4.94 to -3.48)

Czechia	1211 (1131 to 1296)	48 (41 to 55)	893 (763 to 1018)	-0.89 (-0.95 to -0.84)	-5.20 (-5.75 to -4.65)	-4.80 (-5.27 to -4.32)
Democratic People's Republic of Korea	1672 (1532 to 1823)	102 (71 to 141)	2145 (1582 to 2877)	0 (0 to 0.01)	-0.23 (-0.27 to -0.20)	-0.11 (-0.16 to -0.07)
Democratic Republic of the Congo	1427 (1305 to 1558)	91 (56 to 137)	1612 (1056 to 2363)	-0.40 (-0.42 to -0.37)	-0.26 (-0.40 to -0.12)	-0.39 (-0.50 to -0.29)
Denmark	844 (782 to 911)	30 (24 to 34)	494 (416 to 561)	-1.51 (-1.58 to -1.44)	-3.11 (-3.43 to -2.78)	-3.09 (-3.41 to -2.76)
Djibouti	1905 (1765 to 2052)	85 (59 to 119)	1613 (1164 to 2202)	-0.14 (-0.15 to -0.14)	-0.11 (-0.22 to 0.01)	-0.14 (-0.21 to -0.07)
Dominica	821 (751 to 892)	86 (66 to 107)	1321 (1027 to 1658)	-0.33 (-0.34 to -0.31)	-0.66 (-0.74 to -0.58)	-0.66 (-0.75 to -0.57)
Dominican Republic	1141 (1051 to 1238)	55 (39 to 73)	1001 (739 to 1331)	0.41 (0.40 to 0.42)	-0.54 (-1.06 to 0)	-0.19 (-0.62 to 0.25)
Ecuador	899 (833 to 967)	31 (25 to 37)	515 (420 to 621)	-0.61 (-0.64 to -0.58)	-2.07 (-2.97 to -1.17)	-2.28 (-3.05 to -1.50)
Egypt	1501 (1360 to 1647)	221 (168 to 282)	3875 (2932 to 4992)	0.66 (0.64 to 0.68)	-0.92 (-1.40 to -0.45)	-0.61 (-1.23 to 0.01)
El Salvador	713 (658 to 772)	25 (18 to 33)	456 (346 to 588)	-0.60 (-0.64 to -0.57)	-1.45 (-2.25 to -0.64)	-1.52 (-2.23 to -0.81)
Equatorial Guinea	1642 (1520 to 1771)	91 (59 to 131)	1646 (1126 to 2322)	-0.39 (-0.41 to -0.36)	-0.63 (-0.82 to -0.44)	-0.76 (-0.92 to -0.60)
Eritrea	1673 (1540 to 1811)	88 (59 to 123)	1605 (1129 to 2201)	-0.24 (-0.25 to -0.23)	-0.36 (-0.44 to -0.27)	-0.52 (-0.6 to -0.44)
Estonia	993 (906 to 1083)	42 (35 to 48)	811 (684 to 937)	-1.05 (-1.07 to -1.02)	-5.18 (-6.06 to -4.29)	-4.81 (-5.56 to -4.06)
Eswatini	1654 (1510 to 1800)	118 (79 to 169)	2116 (1445 to 2997)	0.05 (0.04 to 0.06)	0.08 (-0.09 to 0.25)	0.14 (-0.02 to 0.30)
Ethiopia	1064 (875 to 1266)	43 (32 to 59)	811 (626 to 1067)	-0.71 (-0.73 to -0.68)	-0.77 (-0.88 to -0.65)	-0.95 (-1.05 to -0.86)
Fiji	1573 (1462 to 1690)	68 (50 to 91)	1323 (1007 to 1699)	-0.35 (-0.35 to -0.34)	-0.22 (-0.56 to 0.13)	-0.39 (-0.57 to -0.21)
Finland	1247 (1168 to 1332)	30 (24 to 34)	563 (471 to 646)	-0.86 (-0.92 to -0.79)	-3.64 (-4.03 to -3.25)	-3.44 (-3.65 to -3.23)
France	870 (821 to 921)	21 (17 to 24)	385 (319 to 444)	0 (-0.05 to 0.04)	-3.82 (-4.14 to -3.49)	-3.11 (-3.35 to -2.87)
Gabon	1864 (1731 to 2005)	92 (64 to 126)	1684 (1223 to 2225)	-0.42 (-0.43 to -0.40)	-0.23 (-0.42 to -0.05)	-0.34 (-0.51 to -0.17)
Gambia	1767 (1645 to 1895)	144 (100 to 200)	2639 (1850 to 3614)	-0.26 (-0.27 to -0.24)	0.43 (0.14 to 0.73)	0.35 (0.06 to 0.64)
Georgia	1440 (1319 to 1569)	152 (131 to 171)	2616 (2269 to 2938)	0.27 (0.25 to 0.30)	0.81 (-0.80 to 2.44)	0.60 (-0.64 to 1.86)
Germany	1379 (1296 to 1465)	27 (22 to 31)	571 (480 to 655)	-0.66 (-0.69 to -0.63)	-4.28 (-4.54 to -4.02)	-3.5 (-3.82 to -3.17)
Ghana	2475 (2311 to 2646)	162 (118 to 217)	3052 (2264 to 4025)	0.08 (0.07 to 0.09)	0.17 (0.10 to 0.24)	0.08 (0.01 to 0.16)
Greece	953 (878 to 1035)	47 (39 to 53)	700 (589 to 788)	-0.87 (-0.94 to -0.81)	-4.12 (-4.43 to -3.81)	-3.84 (-4.16 to -3.52)
Greenland	1502 (1395 to 1610)	57 (43 to 73)	962 (750 to 1217)	-1.72 (-1.78 to -1.66)	-3.21 (-3.49 to -2.94)	-3.12 (-3.30 to -2.95)
Grenada	1051 (922 to 1196)	83 (70 to 94)	1317 (1126 to 1495)	-0.62 (-0.63 to -0.61)	-2.04 (-2.57 to -1.50)	-2.32 (-2.81 to -1.82)
Guam	1470 (1378 to 1564)	19 (14 to 24)	643 (515 to 786)	-0.04 (-0.06 to -0.01)	-4.41 (-5.61 to -3.18)	-2.44 (-3.03 to -1.85)
Guatemala	678 (625 to 733)	30 (25 to 35)	477 (402 to 557)	-0.58 (-0.59 to -0.56)	-1.48 (-2.48 to -0.48)	-1.60 (-2.54 to -0.65)
Guinea	1686 (1562 to 1811)	126 (89 to 172)	2332 (1674 to 3162)	0 (-0.01 to 0)	0.41 (0.34 to 0.48)	0.40 (0.31 to 0.49)
Guinea-Bissau	1847 (1712 to 1984)	167 (120 to 224)	3117 (2271 to 4177)	-0.25 (-0.27 to -0.24)	-0.03 (-0.1 to 0.05)	-0.17 (-0.26 to -0.08)

Guyana	1155 (1062 to 1258)	113 (89 to 141)	1918 (1501 to 2392)	-0.78 (-0.81 to -0.75)	-1.38 (-2.03 to -0.72)	-1.69 (-2.24 to -1.13)
Haiti	970 (875 to 1070)	136 (94 to 190)	2248 (1542 to 3197)	-0.30 (-0.32 to -0.29)	-0.85 (-0.90 to -0.81)	-0.90 (-0.97 to -0.83)
Honduras	787 (715 to 863)	87 (62 to 117)	1402 (988 to 1892)	-0.25 (-0.27 to -0.24)	0.99 (0.65 to 1.34)	0.68 (0.32 to 1.04)
Hungary	1208 (1118 to 1305)	67 (56 to 78)	1274 (1079 to 1466)	-1.58 (-1.63 to -1.54)	-3.52 (-4.10 to -2.94)	-3.41 (-3.92 to -2.89)
Iceland	914 (847 to 985)	21 (16 to 25)	379 (309 to 445)	-1.22 (-1.25 to -1.18)	-3.40 (-4.17 to -2.62)	-3.25 (-3.70 to -2.80)
India	709 (579 to 847)	52 (43 to 65)	965 (813 to 1232)	-0.17 (-0.20 to -0.14)	-0.45 (-1.06 to 0.16)	-0.52 (-0.84 to -0.19)
Indonesia	1726 (1424 to 2050)	156 (119 to 194)	2747 (2099 to 3400)	0.09 (0.08 to 0.09)	1.02 (0.97 to 1.07)	0.74 (0.69 to 0.79)
Iran (Islamic Republic of)	1198 (1013 to 1399)	84 (73 to 93)	1504 (1335 to 1660)	-0.39 (-0.41 to -0.36)	-2.03 (-2.16 to -1.91)	-2.05 (-2.16 to -1.94)
Iraq	1547 (1419 to 1683)	195 (149 to 244)	3407 (2573 to 4316)	0 (-0.03 to 0.02)	0.04 (-0.20 to 0.28)	-0.34 (-0.53 to -0.15)
Ireland	602 (549 to 659)	23 (18 to 26)	354 (286 to 411)	-2.06 (-2.09 to -2.02)	-4.64 (-5 to -4.28)	-4.58 (-4.90 to -4.26)
Israel	945 (879 to 1015)	17 (14 to 20)	347 (286 to 406)	-1.10 (-1.21 to -0.99)	-3.99 (-4.85 to -3.13)	-3.38 (-4.25 to -2.49)
Italy	657 (553 to 768)	30 (24 to 34)	450 (370 to 508)	-1.13 (-1.19 to -1.08)	-3.89 (-4.29 to -3.49)	-3.82 (-4.14 to -3.50)
Jamaica	808 (741 to 883)	75 (59 to 94)	1181 (927 to 1473)	-0.38 (-0.39 to -0.37)	-0.91 (-1.49 to -0.32)	-0.9 (-1.51 to -0.28)
Japan	1160 (995 to 1338)	22 (18 to 25)	484 (400 to 562)	-0.63 (-0.65 to -0.62)	-4.37 (-4.81 to -3.92)	-3.49 (-3.81 to -3.17)
Jordan	1829 (1695 to 1966)	86 (66 to 108)	1582 (1252 to 1949)	0.06 (0.01 to 0.11)	-2.40 (-3.10 to -1.69)	-2.19 (-2.77 to -1.60)
Kazakhstan	1893 (1748 to 2050)	160 (139 to 181)	2780 (2412 to 3154)	-0.69 (-0.75 to -0.64)	-0.44 (-1.31 to 0.43)	-0.76 (-1.34 to -0.17)
Kenya	1515 (1256 to 1796)	66 (48 to 86)	1198 (925 to 1504)	-0.19 (-0.20 to -0.18)	0.41 (0.32 to 0.50)	0.26 (0.21 to 0.31)
Kiribati	1779 (1646 to 1921)	86 (62 to 115)	1752 (1311 to 2275)	-0.29 (-0.29 to -0.28)	0.21 (0.18 to 0.25)	0.02 (-0.01 to 0.05)
Kuwait	1317 (1227 to 1413)	41 (32 to 50)	814 (662 to 981)	-0.22 (-0.28 to -0.17)	-0.92 (-3.01 to 1.23)	-1.05 (-2.82 to 0.75)
Kyrgyzstan	1241 (1143 to 1347)	94 (77 to 110)	2016 (1689 to 2357)	-1.09 (-1.12 to -1.06)	-2.10 (-2.54 to -1.64)	-1.57 (-2.03 to -1.11)
Lao People's Democratic Republic	1322 (1211 to 1440)	124 (91 to 164)	2237 (1671 to 2945)	-0.14 (-0.14 to -0.13)	-1.02 (-1.07 to -0.96)	-1.16 (-1.20 to -1.13)
Latvia	1459 (1344 to 1581)	139 (119 to 156)	2310 (1995 to 2595)	-0.15 (-0.23 to -0.07)	-1.42 (-2.42 to -0.40)	-1.51 (-2.48 to -0.53)
Lebanon	1342 (1232 to 1455)	45 (34 to 57)	840 (657 to 1049)	0.24 (0.21 to 0.27)	-3.11 (-3.32 to -2.89)	-2.92 (-3.12 to -2.72)
Lesotho	1540 (1394 to 1694)	142 (98 to 197)	2502 (1765 to 3475)	0.62 (0.60 to 0.64)	1.52 (1.06 to 1.98)	1.61 (1.22 to 1.99)
Liberia	1463 (1355 to 1573)	119 (85 to 164)	2145 (1535 to 2940)	-0.49 (-0.5 to -0.47)	-0.11 (-0.27 to 0.04)	-0.15 (-0.32 to 0.01)
Libya	1265 (1168 to 1367)	91 (62 to 127)	1746 (1233 to 2421)	0.38 (0.36 to 0.41)	0.36 (-0.13 to 0.85)	0.50 (0.17 to 0.82)
Lithuania	1344 (1126 to 1580)	86 (73 to 97)	1533 (1312 to 1751)	0 (-0.23 to 0.23)	-1.07 (-1.53 to -0.6)	-1.26 (-1.99 to -0.52)

Luxembourg	626 (581 to 673)	25 (20 to 28)	396 (332 to 453)	-1.78 (-1.82 to -1.74)	-5.59 (-6.27 to -4.9)	-5.34 (-5.93 to -4.75)
Madagascar	2127 (1969 to 2294)	113 (76 to 156)	2113 (1482 to 2846)	-0.11 (-0.11 to -0.1)	-0.21 (-0.34 to -0.09)	-0.28 (-0.45 to -0.1)
Malawi	1611 (1484 to 1738)	106 (75 to 143)	1908 (1399 to 2549)	-0.23 (-0.24 to -0.22)	0.41 (0.21 to 0.61)	0.31 (0.19 to 0.43)
Malaysia	1519 (1410 to 1634)	67 (52 to 85)	1360 (1085 to 1665)	-0.06 (-0.07 to -0.06)	-0.64 (-1.05 to -0.23)	-0.64 (-0.76 to -0.52)
Maldives	1086 (1002 to 1176)	64 (48 to 79)	1106 (868 to 1363)	-1.49 (-1.55 to -1.43)	-2.33 (-2.49 to -2.16)	-2.66 (-2.82 to -2.49)
Mali	1252 (1157 to 1347)	87 (60 to 124)	1574 (1103 to 2240)	-0.45 (-0.47 to -0.43)	-0.17 (-0.37 to 0.03)	-0.20 (-0.34 to -0.06)
Malta	587 (536 to 641)	24 (19 to 28)	386 (317 to 448)	-1.68 (-1.74 to -1.63)	-4.81 (-5.22 to -4.40)	-4.50 (-4.92 to -4.08)
Marshall Islands	1499 (1379 to 1628)	86 (60 to 120)	1644 (1193 to 2234)	-0.11 (-0.12 to -0.10)	-0.49 (-0.63 to -0.36)	-0.44 (-0.53 to -0.35)
Mauritania	1648 (1533 to 1770)	115 (79 to 166)	2047 (1439 to 2939)	-0.84 (-0.86 to -0.82)	-0.66 (-0.81 to -0.50)	-0.88 (-0.98 to -0.78)
Mauritius	1126 (1043 to 1215)	57 (49 to 64)	1139 (1001 to 1280)	-1.52 (-1.6 to -1.44)	-3.26 (-4.28 to -2.22)	-3.17 (-3.97 to -2.37)
Mexico	914 (775 to 1063)	28 (25 to 31)	510 (451 to 574)	-0.83 (-0.85 to -0.82)	-2.78 (-3.37 to -2.19)	-2.45 (-2.96 to -1.94)
Micronesia (Federated States of)	1541 (1420 to 1668)	87 (61 to 122)	1693 (1227 to 2313)	-0.21 (-0.23 to -0.20)	-0.58 (-0.61 to -0.55)	-0.56 (-0.59 to -0.53)
Monaco	990 (908 to 1075)	50 (36 to 65)	776 (585 to 989)	-1.13 (-1.15 to -1.10)	-2.92 (-3.06 to -2.79)	-2.77 (-2.87 to -2.67)
Mongolia	1292 (1190 to 1395)	37 (26 to 51)	863 (641 to 1114)	0.38 (0.32 to 0.45)	-0.08 (-0.33 to 0.17)	-0.04 (-0.32 to 0.25)
Montenegro	937 (856 to 1023)	102 (77 to 132)	1495 (1144 to 1908)	-0.04 (-0.07 to -0.01)	1.77 (0.97 to 2.58)	1.27 (0.85 to 1.69)
Morocco	1416 (1296 to 1545)	148 (109 to 194)	2629 (1930 to 3465)	0.17 (0.16 to 0.18)	-0.10 (-0.21 to 0.01)	-0.22 (-0.35 to -0.09)
Mozambique	1942 (1782 to 2109)	141 (95 to 195)	2716 (1877 to 3681)	0.37 (0.35 to 0.40)	0.69 (0.59 to 0.79)	0.72 (0.58 to 0.85)
Myanmar	1241 (1139 to 1347)	117 (86 to 156)	2099 (1575 to 2765)	-0.27 (-0.28 to -0.27)	-0.97 (-1.03 to -0.92)	-1.14 (-1.18 to -1.09)
Namibia	1706 (1564 to 1852)	127 (92 to 166)	2193 (1636 to 2835)	-0.46 (-0.48 to -0.44)	-0.26 (-0.48 to -0.04)	-0.33 (-0.57 to -0.08)
Nauru	2088 (1959 to 2224)	125 (88 to 169)	2535 (1851 to 3385)	-0.17 (-0.18 to -0.16)	-0.37 (-0.43 to -0.31)	-0.35 (-0.42 to -0.28)
Nepal	701 (628 to 776)	66 (46 to 95)	1145 (806 to 1639)	-0.32 (-0.34 to -0.31)	-0.84 (-0.92 to -0.76)	-1.01 (-1.06 to -0.95)
Netherlands	945 (871 to 1023)	33 (26 to 37)	529 (439 to 606)	-1.63 (-1.72 to -1.54)	-2.73 (-3.14 to -2.32)	-2.74 (-3.10 to -2.39)
New Zealand	856 (718 to 1012)	30 (24 to 34)	478 (395 to 551)	-0.79 (-0.80 to -0.77)	-2.84 (-3.10 to -2.58)	-2.83 (-3.03 to -2.64)
Nicaragua	843 (773 to 916)	27 (20 to 35)	489 (375 to 624)	-0.74 (-0.77 to -0.72)	-1.82 (-2.22 to -1.43)	-1.80 (-2.23 to -1.38)
Niger	1389 (1281 to 1501)	94 (62 to 140)	1641 (1109 to 2418)	-0.53 (-0.55 to -0.51)	0.04 (-0.06 to 0.14)	-0.13 (-0.22 to -0.05)
Nigeria	1510 (1255 to 1785)	95 (75 to 120)	1712 (1357 to 2141)	-0.17 (-0.17 to -0.16)	-0.84 (-0.97 to -0.7)	-0.84 (-0.95 to -0.72)
Niue	1620 (1506 to 1737)	75 (55 to 98)	1451 (1117 to 1846)	-0.31 (-0.33 to -0.30)	-0.66 (-0.75 to -0.57)	-0.63 (-0.71 to -0.56)
North Macedonia	1576 (1336 to 1841)	344 (288 to 404)	4812 (3978 to 5701)	-0.58 (-0.6 to -0.57)	0.47 (-0.15 to 1.09)	-0.17 (-0.65 to 0.31)

Northern Mariana Islands	1259 (1172 to 1351)	54 (40 to 68)	1035 (808 to 1284)	-0.35 (-0.38 to -0.32)	-1.38 (-2.11 to -0.65)	-1.19 (-1.52 to -0.86)
Norway	1176 (996 to 1366)	23 (19 to 25)	442 (370 to 511)	-1.20 (-1.23 to -1.17)	-4.28 (-4.65 to -3.90)	-3.78 (-4.07 to -3.48)
Oman	1403 (1296 to 1518)	88 (66 to 114)	1612 (1233 to 2051)	0.07 (0.04 to 0.11)	-1.04 (-2.25 to 0.18)	-1.33 (-1.96 to -0.69)
Pakistan	1146 (935 to 1375)	77 (58 to 100)	1423 (1095 to 1858)	-0.01 (-0.01 to 0)	0.10 (0.03 to 0.17)	0.10 (0.03 to 0.17)
Palau	1837 (1702 to 1972)	90 (67 to 118)	1746 (1333 to 2241)	0.04 (0.04 to 0.05)	-0.53 (-0.66 to -0.4)	-0.53 (-0.70 to -0.36)
Palestine	1042 (949 to 1138)	141 (113 to 172)	2238 (1805 to 2735)	-0.28 (-0.32 to -0.24)	-1.55 (-1.69 to -1.41)	-1.60 (-1.88 to -1.33)
Panama	829 (767 to 896)	37 (28 to 44)	596 (467 to 717)	-0.57 (-0.59 to -0.55)	-1.44 (-2.14 to -0.73)	-1.53 (-2.06 to -0.99)
Papua New Guinea	931 (856 to 1008)	67 (45 to 98)	1207 (856 to 1711)	-0.16 (-0.17 to -0.15)	-0.37 (-0.41 to -0.33)	-0.43 (-0.48 to -0.38)
Paraguay	942 (867 to 1022)	61 (45 to 79)	1003 (746 to 1288)	-0.51 (-0.53 to -0.5)	-1.19 (-1.76 to -0.61)	-1.04 (-1.35 to -0.72)
Peru	755 (700 to 813)	23 (16 to 32)	431 (317 to 572)	-0.44 (-0.46 to -0.43)	-1.67 (-3.13 to -0.20)	-1.51 (-2.62 to -0.40)
Philippines	1241 (1036 to 1463)	73 (62 to 84)	1439 (1235 to 1651)	0.5 (0.47 to 0.53)	-0.67 (-1.1 to -0.24)	-0.13 (-0.47 to 0.2)
Poland	934 (780 to 1102)	69 (59 to 76)	1178 (1035 to 1304)	-0.30 (-0.33 to -0.27)	-3.50 (-4.06 to -2.94)	-3.23 (-3.59 to -2.87)
Portugal	633 (578 to 691)	44 (36 to 50)	634 (533 to 714)	-2.82 (-2.91 to -2.73)	-5.64 (-6.17 to -5.1)	-5.59 (-6.05 to -5.12)
Puerto Rico	753 (696 to 811)	15 (12 to 18)	284 (231 to 336)	-0.25 (-0.27 to -0.23)	-3.90 (-4.79 to -3.00)	-3.20 (-3.70 to -2.69)
Qatar	1131 (1039 to 1227)	56 (40 to 74)	930 (687 to 1209)	-1.48 (-1.69 to -1.28)	-2.91 (-4.3 to -1.5)	-2.87 (-3.98 to -1.74)
Republic of Korea	1342 (1256 to 1436)	41 (31 to 50)	746 (597 to 897)	-2.21 (-2.29 to -2.13)	-4.94 (-5.36 to -4.51)	-4.63 (-4.93 to -4.33)
Republic of Moldova	1198 (1098 to 1304)	82 (71 to 92)	1654 (1452 to 1870)	0.09 (0.06 to 0.12)	-1.74 (-2.82 to -0.65)	-1.16 (-1.90 to -0.42)
Romania	1406 (1300 to 1517)	142 (122 to 160)	2303 (1999 to 2598)	-0.48 (-0.53 to -0.43)	-1.99 (-2.48 to -1.50)	-1.83 (-2.26 to -1.4)
Russian Federation	1400 (1183 to 1628)	157 (140 to 170)	2712 (2454 to 2939)	-0.29 (-0.32 to -0.26)	-1.94 (-2.69 to -1.19)	-1.84 (-2.51 to -1.16)
Rwanda	1439 (1317 to 1566)	77 (50 to 110)	1361 (933 to 1882)	-1.03 (-1.05 to -1.01)	-1.86 (-2.01 to -1.70)	-2.02 (-2.19 to -1.85)
Saint Kitts and Nevis	1129 (1036 to 1232)	117 (99 to 133)	1814 (1522 to 2092)	-1.34 (-1.39 to -1.28)	-1.91 (-2.78 to -1.03)	-2.01 (-2.80 to -1.22)
Saint Lucia	981 (907 to 1063)	84 (69 to 100)	1240 (1022 to 1471)	-0.89 (-0.93 to -0.85)	-2.93 (-3.37 to -2.48)	-2.73 (-3.49 to -1.97)
Saint Vincent and the Grenadines	931 (857 to 1009)	79 (68 to 89)	1203 (1040 to 1372)	-0.79 (-0.82 to -0.76)	-1.36 (-1.89 to -0.84)	-1.48 (-2.16 to -0.79)
Samoa	1582 (1470 to 1698)	69 (50 to 91)	1354 (1044 to 1724)	-0.04 (-0.04 to -0.04)	-0.60 (-0.66 to -0.55)	-0.49 (-0.53 to -0.45)
San Marino	871 (798 to 949)	23 (15 to 34)	424 (303 to 573)	-0.99 (-1.03 to -0.96)	-4.11 (-4.79 to -3.43)	-3.46 (-3.86 to -3.05)
Sao Tome and Principe	2197 (2046 to 2351)	122 (93 to 157)	2281 (1772 to 2930)	0 (-0.01 to 0.02)	0.41 (0.22 to 0.61)	0.40 (0.20 to 0.60)
Saudi Arabia	1162 (1064 to 1266)	108 (82 to 138)	1957 (1495 to 2507)	0.1 (0.07 to 0.12)	-1.23 (-1.37 to -1.09)	-1.15 (-1.27 to -1.03)

Senegal	1710 (1590 to 1833)	126 (91 to 173)	2263 (1658 to 3075)	-0.47 (-0.48 to -0.46)	-0.16 (-0.37 to 0.06)	-0.40 (-0.64 to -0.16)
Serbia	1297 (1118 to 1489)	202 (165 to 242)	3116 (2546 to 3735)	-0.81 (-0.83 to -0.79)	-1.96 (-2.21 to -1.7)	-1.89 (-2.15 to -1.63)
Seychelles	1294 (1199 to 1393)	69 (52 to 86)	1315 (1020 to 1622)	-0.45 (-0.46 to -0.43)	-1.15 (-1.73 to -0.57)	-1.44 (-2.14 to -0.74)
Sierra Leone	1757 (1632 to 1891)	129 (92 to 175)	2414 (1721 to 3270)	-0.30 (-0.32 to -0.29)	-0.23 (-0.43 to -0.04)	-0.27 (-0.43 to -0.12)
Singapore	1038 (964 to 1115)	11 (9 to 13)	322 (261 to 383)	-2.32 (-2.38 to -2.26)	-6.58 (-7.62 to -5.51)	-5.44 (-6.51 to -4.36)
Slovakia	1610 (1497 to 1724)	77 (61 to 95)	1466 (1180 to 1767)	-0.89 (-1.05 to -0.74)	-2.25 (-2.67 to -1.82)	-2.17 (-2.53 to -1.82)
Slovenia	759 (706 to 815)	40 (33 to 46)	660 (553 to 765)	-1.12 (-1.2 to -1.04)	-3.97 (-4.78 to -3.15)	-3.90 (-4.28 to -3.53)
Solomon Islands	1743 (1593 to 1894)	87 (59 to 126)	1671 (1198 to 2324)	-0.01 (-0.02 to 0)	-0.18 (-0.28 to -0.08)	-0.18 (-0.35 to 0)
Somalia	1435 (1300 to 1569)	67 (39 to 106)	1295 (817 to 1965)	-0.33 (-0.36 to -0.31)	-0.65 (-0.76 to -0.54)	-0.64 (-0.74 to -0.54)
South Africa	1742 (1466 to 2045)	91 (79 to 103)	1609 (1415 to 1807)	-0.60 (-0.63 to -0.56)	0.88 (0.24 to 1.53)	0.49 (-0.11 to 1.08)
South Sudan	1444 (1327 to 1563)	70 (47 to 100)	1319 (920 to 1860)	-0.38 (-0.40 to -0.36)	-0.54 (-0.65 to -0.44)	-0.54 (-0.63 to -0.45)
Spain	943 (891 to 995)	22 (17 to 25)	406 (335 to 468)	-0.5 (-0.57 to -0.42)	-5.04 (-5.22 to -4.86)	-4.29 (-4.57 to -4.02)
Sri Lanka	1192 (1098 to 1287)	112 (79 to 148)	1764 (1270 to 2317)	-0.39 (-0.40 to -0.38)	-1.24 (-1.80 to -0.68)	-1.29 (-1.89 to -0.68)
Sudan	1371 (1250 to 1497)	129 (88 to 175)	2385 (1639 to 3278)	0.27 (0.26 to 0.29)	-0.79 (-0.85 to -0.73)	-0.78 (-0.85 to -0.72)
Suriname	1012 (929 to 1097)	67 (47 to 90)	1162 (848 to 1543)	-0.28 (-0.29 to -0.26)	-0.80 (-1.40 to -0.20)	-0.85 (-1.35 to -0.35)
Sweden	1070 (897 to 1252)	25 (20 to 29)	451 (370 to 531)	-0.71 (-0.74 to -0.69)	-3.58 (-4.09 to -3.06)	-3.21 (-3.62 to -2.79)
Switzerland	714 (662 to 770)	18 (14 to 21)	318 (260 to 369)	-0.70 (-0.73 to -0.67)	-4.23 (-4.37 to -4.08)	-3.68 (-3.82 to -3.54)
Syrian Arab Republic	1143 (1047 to 1244)	107 (80 to 139)	1859 (1393 to 2428)	-0.63 (-0.67 to -0.58)	-0.63 (-0.90 to -0.36)	-0.84 (-1.07 to -0.60)
Taiwan (Province of China)	1342 (1255 to 1431)	23 (19 to 26)	600 (501 to 698)	-0.94 (-0.98 to -0.90)	-4.33 (-5.20 to -3.44)	-3.46 (-3.97 to -2.94)
Tajikistan	1246 (1125 to 1372)	115 (89 to 144)	2107 (1655 to 2627)	-0.09 (-0.13 to -0.05)	-0.49 (-0.92 to -0.05)	-0.71 (-1.11 to -0.30)
Thailand	1123 (1046 to 1203)	42 (29 to 56)	908 (683 to 1171)	-0.51 (-0.54 to -0.48)	-1.67 (-2.11 to -1.22)	-1.43 (-1.76 to -1.09)
Timor-Leste	1140 (1041 to 1243)	112 (77 to 153)	1963 (1369 to 2662)	0.19 (0.17 to 0.22)	0.34 (0.21 to 0.46)	0.28 (0.16 to 0.40)
Togo	1699 (1577 to 1827)	131 (91 to 179)	2425 (1720 to 3272)	-0.41 (-0.42 to -0.39)	-0.02 (-0.12 to 0.08)	-0.07 (-0.16 to 0.02)
Tokelau	1329 (1236 to 1425)	68 (47 to 92)	1302 (961 to 1710)	-0.07 (-0.08 to -0.06)	-1.21 (-1.23 to -1.19)	-1.07 (-1.1 to -1.04)
Tonga	1185 (1099 to 1274)	48 (34 to 64)	939 (707 to 1213)	-0.13 (-0.14 to -0.13)	-0.14 (-0.38 to 0.11)	-0.24 (-0.49 to 0.01)
Trinidad and Tobago	1060 (979 to 1145)	68 (53 to 84)	1131 (887 to 1408)	-0.98 (-1.02 to -0.95)	-2.22 (-2.47 to -1.98)	-2.04 (-2.34 to -1.73)
Tunisia	1114 (1021 to 1214)	95 (63 to 134)	1617 (1101 to 2244)	0.27 (0.24 to 0.30)	-1.03 (-1.34 to -0.71)	-0.94 (-1.06 to -0.83)
Turkey	1157 (1071 to 1249)	75 (57 to 95)	1232 (958 to 1549)	-1.01 (-1.07 to -0.95)	-1.70 (-2.10 to -1.31)	-1.98 (-2.27 to -1.69)
Turkmenistan	1931 (1772 to 2098)	142 (109 to 178)	3053 (2370 to 3804)	0.67 (0.62 to 0.73)	0.72 (-0.15 to 1.61)	0.71 (-0.05 to 1.47)

Tuvalu	1439 (1332 to 1552)	88 (64 to 117)	1672 (1264 to 2165)	-0.04 (-0.05 to -0.03)	-0.76 (-0.80 to -0.73)	-0.74 (-0.78 to -0.71)
Uganda	1699 (1569 to 1834)	62 (43 to 87)	1192 (873 to 1613)	-0.32 (-0.35 to -0.29)	-0.88 (-0.98 to -0.77)	-0.81 (-0.9 to -0.72)
Ukraine	1538 (1286 to 1824)	127 (97 to 162)	2313 (1785 to 2910)	-0.51 (-0.56 to -0.47)	-2.07 (-2.68 to -1.46)	-1.84 (-2.41 to -1.26)
United Arab Emirates	1843 (1684 to 2010)	99 (74 to 129)	1756 (1350 to 2226)	-0.42 (-0.44 to -0.41)	-1.15 (-4.13 to 1.93)	-1.45 (-3.66 to 0.8)
United Kingdom	896 (789 to 1010)	24 (20 to 26)	424 (363 to 474)	-1.01 (-1.05 to -0.96)	-4.40 (-4.86 to -3.94)	-4.07 (-4.42 to -3.71)
United Republic of Tanzania	1803 (1665 to 1942)	84 (57 to 116)	1536 (1101 to 2084)	0.46 (0.41 to 0.51)	0.76 (0.60 to 0.92)	0.64 (0.45 to 0.83)
United States Virgin Islands	1468 (1270 to 1676)	27 (22 to 30)	565 (478 to 643)	-0.05 (-0.07 to -0.03)	-2.65 (-2.94 to -2.36)	-2.48 (-2.80 to -2.17)
United States of America	813 (746 to 885)	31 (23 to 41)	515 (382 to 675)	-0.13 (-0.24 to -0.02)	-1.62 (-1.94 to -1.29)	-1.26 (-1.41 to -1.11)
Uruguay	1088 (1010 to 1171)	52 (44 to 59)	892 (773 to 998)	-1.62 (-1.65 to -1.59)	-2.74 (-3.11 to -2.36)	-2.6 (-2.87 to -2.34)
Uzbekistan	1698 (1566 to 1839)	97 (82 to 113)	2005 (1720 to 2329)	-0.02 (-0.03 to -0.01)	0.05 (-0.82 to 0.93)	-0.28 (-1.27 to 0.73)
Vanuatu	1858 (1718 to 1999)	86 (60 to 115)	1717 (1277 to 2228)	0.02 (0.01 to 0.03)	-0.67 (-0.76 to -0.58)	-0.59 (-0.69 to -0.49)
Venezuela (Bolivarian Republic of)	830 (763 to 898)	44 (33 to 56)	719 (552 to 917)	-0.65 (-0.67 to -0.62)	-0.52 (-0.83 to -0.21)	-0.74 (-1.02 to -0.45)
Viet Nam	1369 (1272 to 1472)	152 (114 to 192)	2597 (1960 to 3289)	0.36 (0.33 to 0.39)	0.22 (0.15 to 0.30)	0.19 (0.14 to 0.25)
Yemen	1150 (1042 to 1264)	171 (117 to 240)	3064 (2104 to 4301)	0.07 (0.05 to 0.09)	-0.18 (-0.27 to -0.09)	-0.27 (-0.40 to -0.14)
Zambia	1799 (1658 to 1945)	103 (71 to 143)	1846 (1323 to 2510)	0.13 (0.12 to 0.14)	0.52 (0.4 to 0.63)	0.46 (0.31 to 0.60)
Zimbabwe	1643 (1509 to 1779)	113 (83 to 147)	2050 (1544 to 2664)	0.34 (0.33 to 0.35)	1.19 (0.74 to 1.64)	1.16 (0.51 to 1.81)

Note: Estimates are for individuals aged over 20 years. AAPCs=average annual percent changes. DALYs=disability-adjusted life years.

eTable 8. Risk factors for ischemic stroke related DALYs among people aged ≥20 years in 1990

	Globally		High SDI		High-middle SDI		Middle SDI		Low-middle SDI		Low SDI	
	Absolute NO (00000s)	Percent	Absolute NO (00000s)	Percent	Absolute NO (00000s)	Percent	Absolute NO (00000s)	Percent	Absolute NO (00000s)	Percent	Absolute NO (00000s)	Percent
**Environmental risks**	179.9 (146.1 to 215.3)	39.7% (32.5 to 46.7)	25.5 (19.4 to 32.4)	25.0% (19.5 to 31.5)	60.7 (48.4 to 74.5)	36.2% (29.1 to 44.1)	52.5 (42.6 to 64.0)	49.0% (40.6 to 57.0)	29.8 (23.4 to 37.1)	52.7% (43.6 to 61.2)	11.3 (8.7 to 14.7)	56.5% (47.3 to 65.0)
Ambient particulate matter pollution	60.0 (42.3 to 81.3)	13.2% (9.4 to 17.8)	14.1 (9.3 to 20.3)	13.8% (9.2 to 20.0)	27.0 (17.8 to 38.3)	16.1% (10.7 to 22.9)	11.8 (7.4 to 17.4)	11.0% (7.2 to 16.4)	5.5 (3.4 to 8.0)	9.7% (6.2 to 13.7)	1.6 (1.0 to 2.3)	7.8% (5.0 to 11.3)
Household air pollution from solid fuels	81.4 (61.4 to 107.0)	18.0% (13.7 to 23.4)	1.9 (0.6 to 4.4)	1.9% (0.6 to 4.4)	18.6 (12.6 to 28.5)	11.1% (7.6 to 16.9)	32.5 (25.1 to 40.9)	30.4% (23.9 to 37.3)	20.0 (15.7 to 25.1)	35.5% (28.5 to 42.2)	8.3 (6.4 to 10.6)	41.8% (34.8 to 49.1)
Low temperature	28.7 (24.7 to 33.4)	6.3% (5.5 to 7.1)	7.6 (6.6 to 9.1)	7.5% (6.6 to 9.1)	13.9 (11.9 to 15.8)	8.3% (7.1 to 9.6)	5.3 (4.4 to 6.6)	5.0% (4.3 to 6.0)	1.4 (0.9 to 2.1)	2.5% (1.6 to 3.6)	0.5 (0.3 to 0.7)	2.4% (1.8 to 3.1)
High temperature	2.2 (-0.03 to 5.7)	0.5% (-0.01 to 1.3)	0.2 (-0.18 to 0.7)	0.2% (-0.17 to 0.7)	0.2 (-0.36 to 1.0)	0.09% (-0.22 to 0.6)	0.7 (0.00 to 1.8)	0.6% (0.00 to 1.7)	0.9 (0.3 to 1.9)	1.6% (0.5 to 3.1)	0.3 (0.1 to 0.5)	1.3% (0.6 to 2.4)
Lead exposure	28.2 (-3.71 to 62.3)	6.2% (-0.81 to 14.0)	3.9 (-0.52 to 8.9)	3.8% (-0.49 to 8.7)	7.8 (-1.02 to 17.6)	4.6% (-0.60 to 10.6)	8.6 (-1.15 to 19.2)	8.1% (-1.05 to 18.3)	5.8 (-0.76 to 12.8)	10.2% (-1.36 to 22.6)	2.1 (-0.27 to 4.6)	10.5% (-1.42 to 23.2)
**Dietary risks**	93.6 (29.8 to 154.5)	20.6% (6.5 to 33.9)	15.9 (3.8 to 28.1)	15.6% (3.7 to 28.2)	34.5 (7.8 to 58.4)	20.6% (4.7 to 34.7)	26.0 (10.4 to 41.0)	24.3% (10.1 to 38.1)	12.5 (4.7 to 20.3)	22.1% (8.1 to 34.8)	4.5 (1.5 to 7.7)	22.6% (7.4 to 35.9)
Diet high in sodium	45.4 (11.7 to 97.9)	10.0% (2.6 to 21.7)	8.2 (1.6 to 19.5)	8.0% (1.6 to 19.0)	17.5 (4.8 to 37.1)	10.4% (2.9 to 22.2)	14.8 (4.6 to 28.2)	13.8% (4.3 to 26.8)	3.9 (0.6 to 10.0)	6.8% (1.1 to 17.4)	1.0 (0.05 to 3.1)	5.2% (0.3 to 15.5)
Diet high in red meat	-0.58 (-3.38 to 6.1)	-0.13% (-0.74 to 1.4)	0.6 (-0.39 to 2.5)	0.6% (-0.41 to 2.4)	0.2 (-0.55 to 3.3)	0.10% (-0.33 to 2.0)	-0.97 (-2.88 to 0.5)	-0.90% (-2.68 to 0.5)	-0.28 (-0.80 to 0.1)	-0.49% (-1.39 to 0.3)	-0.14 (-0.40 to 0.07)	-0.69% (-1.88 to 0.4)

Diet low in fruits	17.4 (9.8 to 26.5)	3.8% (2.1 to 5.8)	1.8 (0.7 to 3.0)	1.7% (0.7 to 2.9)	4.9 (2.6 to 7.4)	2.9% (1.6 to 4.4)	5.1 (2.9 to 7.6)	4.8% (2.7 to 7.0)	4.4 (2.5 to 6.8)	7.7% (4.4 to 11.3)	1.3 (0.7 to 2.0)	6.3% (3.6 to 9.1)
Diet high in processed meat	5.1 (1.1 to 9.2)	1.1% (0.3 to 2.0)	1.7 (0.4 to 2.9)	1.7% (0.4 to 2.9)	3.0 (0.7 to 5.5)	1.8% (0.4 to 3.3)	0.2 (0.05 to 0.3)	0.2% (0.04 to 0.3)	0.1 (0.04 to 0.3)	0.3% (0.06 to 0.4)	0.06 (0.02 to 0.1)	0.3% (0.07 to 0.5)
Diet low in vegetables	11.7 (6.6 to 17.6)	2.6% (1.5 to 3.8)	1.1 (0.2 to 2.0)	1.1% (0.2 to 1.9)	2.0 (1.1 to 3.2)	1.2% (0.6 to 1.9)	3.8 (2.3 to 5.6)	3.6% (2.2 to 5.0)	3.0 (1.8 to 4.6)	5.4% (3.3 to 7.7)	1.7 (1.0 to 2.5)	8.4% (5.2 to 11.3)
Diet low in whole grains	23.4 (-24.92 to 64.0)	5.1% (-5.43 to 14.1)	3.4 (-3.35 to 10.2)	3.3% (-3.30 to 9.9)	10.2 (-10.98 to 28.0)	6.1% (-6.53 to 17.0)	5.7 (-5.77 to 15.0)	5.3% (-5.60 to 13.7)	2.9 (-3.09 to 7.8)	5.1% (-5.38 to 13.2)	1.2 (-1.28 to 3.4)	6.0% (-6.33 to 15.6)
High alcohol use	27.8 (-3.71 to 68.0)	6.1% (-0.78 to 14.9)	9.3 (-1.05 to 22.0)	9.1% (-1.02 to 21.8)	12.5 (-1.48 to 30.6)	7.4% (-0.86 to 18.2)	4.4 (-0.79 to 10.9)	4.1% (-0.72 to 9.8)	1.1 (-0.17 to 3.0)	1.9% (-0.30 to 5.0)	0.5 (-0.09 to 1.4)	2.6% (-0.45 to 6.7)
Diet high in sugar-sweetened beverages	0.8 (0.4 to 1.2)	0.2% (0.08 to 0.3)	0.4 (0.2 to 0.6)	0.4% (0.2 to 0.6)	0.3 (0.1 to 0.4)	0.2% (0.08 to 0.3)	0.09 (0.04 to 0.1)	0.08% (0.04 to 0.1)	0.04 (0.02 to 0.06)	0.07% (0.03 to 0.1)	0.01 (0.00 to 0.02)	0.05% (0.02 to 0.07)
Diet low in fiber	8.9 (-0.40 to 18.6)	2.0% (-0.09 to 4.0)	1.3 (-0.08 to 3.1)	1.3% (-0.08 to 3.0)	2.3 (-0.09 to 4.9)	1.4% (-0.05 to 2.9)	3.1 (-0.15 to 6.3)	2.9% (-0.14 to 5.7)	1.7 (-0.09 to 3.5)	3.1% (-0.17 to 6.0)	0.3 (-0.02 to 0.7)	1.7% (-0.09 to 3.3)
Diet low in polyunsaturated fatty acids	0.1 (0.04 to 0.2)	0.03% (0.01 to 0.05)	0.02 (0.01 to 0.04)	0.02% (0.01 to 0.04)	0.04 (0.01 to 0.08)	0.03% (0.01 to 0.05)	0.03 (0.01 to 0.06)	0.03% (0.01 to 0.06)	0.02 (0.01 to 0.03)	0.03% (0.01 to 0.06)	0.01 (0.00 to 0.01)	0.04% (0.01 to 0.06)
**Physical activity**												
Low physical activity	21.9 (6.5 to 39.2)	4.8% (1.5 to 8.6)	4.5 (0.1 to 9.4)	4.4% (0.1 to 9.0)	7.3 (1.9 to 13.4)	4.4% (1.2 to 8.0)	6.0 (2.4 to 10.0)	5.6% (2.3 to 9.3)	3.2 (1.4 to 5.2)	5.6% (2.4 to 9.2)	0.8 (0.3 to 1.5)	4.2% (1.8 to 7.0)
**Tobacco smoking**	81.1 (66.1 to 98.4)	17.9% (14.8 to 21.1)	16.7 (13.7 to 20.1)	16.4% (13.5 to 19.4)	30.0 (24.7 to 35.9)	17.9% (14.9 to 21.1)	22.7 (18.3 to 28.1)	21.2% (17.8 to 24.9)	9.5 (7.4 to 12.4)	16.8% (13.7 to 20.1)	2.1 (1.6 to 2.9)	10.5% (8.5 to 12.7)
Smoking	63.7 (53.8 to 75.3)	14.0% (11.9 to 16.3)	14.2 (12.0 to 16.9)	14.0% (11.8 to 16.4)	23.4 (19.9 to 27.4)	13.9% (12.0 to 16.1)	17.5 (14.6 to 21.4)	16.4% (14.0 to 18.9)	7.0 (5.5 to 9.0)	12.4% (10.3 to 14.7)	1.5 (1.2 to 2.0)	7.4% (6.1 to 8.9)
Secondhand smoke	20.1 (13.9 to 27.1)	4.4% (3.1 to 5.9)	2.9 (2.0 to 4.0)	2.9% (2.0 to 3.8)	7.7 (5.3 to 10.3)	4.6% (3.1 to 6.1)	6.0 (4.1 to 8.1)	5.6% (3.9 to 7.3)	2.8 (1.9 to 3.9)	5.0% (3.4 to 6.6)	0.7 (0.4 to 1.0)	3.4% (2.3 to 4.5)

**Physiological factors**	353.9 (301.5 to 399.8)	78.0% (66.4 to 86.6)	82.4 (70.9 to 91.9)	81.0% (70.2 to 88.9)	133.2 (114.8 to 148.7)	79.5% (67.9 to 87.9)	80.5 (67.1 to 95.3)	75.3% (63.6 to 84.6)	42.6 (34.6 to 51.7)	75.5% (64.2 to 84.4)	14.5 (11.5 to 18.6)	72.8% (61.1 to 82.2)
High body-mass index	21.3 (3.1 to 41.8)	4.7% (0.7 to 8.9)	5.4 (0.8 to 10.6)	5.3% (0.8 to 10.3)	9.9 (1.4 to 19.4)	5.9% (0.9 to 11.4)	3.5 (0.5 to 6.9)	3.3% (0.5 to 6.3)	1.9 (0.3 to 3.8)	3.4% (0.5 to 6.5)	0.5 (0.06 to 0.9)	2.4% (0.3 to 4.4)
High fasting plasma glucose	61.9 (48.3 to 78.2)	13.7% (10.8 to 16.9)	15.8 (12.3 to 19.5)	15.5% (12.3 to 19.0)	21.5 (16.9 to 26.9)	12.8% (10.1 to 16.0)	14.7 (11.4 to 19.0)	13.8% (10.9 to 17.1)	7.4 (5.7 to 9.5)	13.1% (10.3 to 16.2)	2.3 (1.7 to 3.2)	11.7% (9.1 to 14.5)
High systolic blood pressure	264.2 (198.4 to 317.9)	58.3% (44.4 to 69.5)	64.0 (48.7 to 76.1)	62.9% (48.3 to 73.7)	101.3 (76.2 to 121.8)	60.5% (46.5 to 71.7)	56.6 (41.5 to 71.3)	53.0% (39.6 to 65.2)	31.1 (22.4 to 39.5)	55.1% (40.7 to 66.3)	10.6 (7.5 to 14.2)	53.3% (39.4 to 64.2)
High LDL cholesterol	145.1 (51.9 to 230.7)	32.0% (11.3 to 51.0)	33.2 (11.4 to 53.9)	32.6% (11.1 to 53.0)	56.5 (20.3 to 88.9)	33.7% (12.0 to 53.4)	33.5 (12.5 to 53.7)	31.3% (11.5 to 49.5)	16.4 (5.9 to 27.0)	29.0% (10.5 to 46.7)	5.3 (1.7 to 8.9)	26.6% (9.4 to 43.8)
Kidney dysfunction	46.7 (33.6 to 60.9)	10.3% (7.4 to 13.2)	9.5 (6.3 to 13.0)	9.4% (6.2 to 12.6)	16.6 (11.8 to 21.7)	9.9% (7.1 to 12.8)	11.8 (8.7 to 15.5)	11.1% (8.2 to 14.0)	6.4 (4.7 to 8.5)	11.4% (8.5 to 14.6)	2.2 (1.5 to 3.1)	10.9% (8.1 to 13.8)
**Combined risk factors**												
All factors	409.3 (372.9 to 447.0)	90.3% (84.5 to 94.3)	90.9 (81.7 to 98.3)	89.3% (82.8 to 94.2)	151.6 (138.4 to 163.2)	90.5% (84.3 to 94.7)	96.9 (86.5 to 110.4)	90.6% (85.1 to 94.3)	51.3 (44.9 to 59.7)	90.7% (85.6 to 94.2)	17.9 (15.0 to 22.8)	90.0% (84.5 to 93.6)

Note: Data in parentheses are 95% uncertainty intervals. Count data in 100000 are presented to two decimal places and percentage data are presented to one decimal place. Percentages and number of DALYs are not mutually exclusive: the sum of percentages and number of DALYs in the columns exceeds the totals for all risk factors combined because of overlap between various risk factors. The crude sum of population attributable fraction (PAF) of the risk factors might exceed 100% because the effects of many of these risk factors are mediated partly or wholly through another risk factor or risk factors. DALY=disability-adjusted life-year.

eTable 9. Ischemic stroke related DALYs (absolute numbers [in 100000 with 95% UI] and percentage [with 95% UI]) associated with risk factors and their clusters in 1990 and 2021, by SDI, aged ≥20 years, both sexes

	1990		2021	
	Absolute NO (00000s)	Percent	Absolute NO (00000s)	Percent
Globally	409.3 (372.9 to 447.0)	90.3% (84.5 to 94.3)	622.5 (557.3 to 686.7)	89.2% (82.8 to 93.6)
SDI regions				
High SDI	90.9 (81.7 to 98.3)	89.3% (82.8 to 94.2)	76.4 (66.1 to 86.4)	85.4% (77.3 to 91.4)
High-middle SDI	151.6 (138.4 to 163.2)	90.5% (84.3 to 94.7)	186.8 (166.1 to 208.5)	88.9% (82.0 to 93.6)
Middle SDI	96.9 (86.5 to 110.4)	90.6% (85.1 to 94.3)	213.1 (189.0 to 238.3)	89.6% (83.7 to 94.0)
Low-middle SDI	51.3 (44.9 to 59.7)	90.7% (85.6 to 94.2)	110.4 (97.8 to 126.8)	90.9% (85.5 to 94.5)
Low SDI	17.9 (15.0 to 22.8)	90.0% (84.5 to 93.6)	35.3 (30.0 to 43.6)	90.6% (85.0 to 94.1)
21 GBD regions				
Andean Latin America	0.9 (0.8 to 1.0)	84.2% (76.6 to 89.7)	1.5 (1.3 to 1.8)	84.0% (75.6 to 90.4)
Australasia	1.4 (1.2 to 1.6)	87.1% (78.5 to 93.3)	1.2 (1.0 to 1.4)	83.1% (73.2 to 90.5)
Caribbean	1.9 (1.7 to 2.1)	85.7% (77.5 to 91.3)	3.0 (2.6 to 3.5)	85.6% (77.9 to 91.3)

Central Asia	6.4 (5.8 to 6.9)	89.4% (82.3 to 93.9)	8.9 (7.9 to 9.9)	90.6% (84.1 to 94.7)
Central Europe	29.4 (27.4 to 31.2)	92.0% (85.8 to 95.9)	23.1 (20.9 to 25.2)	89.2% (82.7 to 94.1)
Central Latin America	4.1 (3.7 to 4.4)	87.2% (80.3 to 92.2)	6.9 (6.0 to 7.8)	86.2% (78.3 to 91.9)
Central Sub-Saharan Africa	1.9 (1.5 to 2.4)	91.8% (86.9 to 95.0)	3.9 (3.0 to 5.1)	90.3% (84.5 to 93.9)
East Asia	92.4 (79.0 to 109.3)	91.2% (85.9 to 94.7)	215.1 (182.0 to 249.0)	89.7% (83.7 to 94.1)
Eastern Europe	66.9 (61.5 to 70.9)	90.3% (83.3 to 94.8)	50.5 (44.9 to 55.5)	88.4% (80.5 to 93.6)
Eastern Sub-Saharan Africa	5.2 (4.2 to 6.4)	87.7% (81.5 to 91.7)	10.7 (9.0 to 12.5)	89.3% (83.4 to 93.1)
High-income Asia Pacific	17.1 (15.3 to 18.6)	89.1% (82.7 to 94.0)	15.5 (13.0 to 17.9)	83.6% (75.7 to 90.1)
High-income North America	17.4 (15.2 to 19.4)	87.4% (79.5 to 93.1)	19.9 (16.8 to 22.7)	83.9% (75.3 to 90.4)
North Africa and Middle East	24.8 (21.5 to 28.5)	89.1% (82.4 to 93.5)	48.0 (42.0 to 54.2)	90.5% (84.1 to 94.4)
Oceania	0.2 (0.1 to 0.2)	88.4% (82.2 to 92.7)	0.4 (0.3 to 0.5)	89.0% (82.8 to 93.1)
South Asia	35.6 (29.1 to 44.8)	91.6% (86.5 to 94.8)	82.8 (70.9 to 105.2)	90.9% (85.5 to 94.5)
Southeast Asia	26.7 (23.7 to 29.8)	91.4% (86.4 to 94.6)	65.5 (55.9 to 75.6)	90.1% (84.4 to 94.4)
Southern Latin America	3.6 (3.2 to 4.0)	86.4% (77.4 to 92.2)	3.1 (2.7 to 3.4)	87.4% (79.6 to 93.0)
Southern Sub-Saharan Africa	2.0 (1.7 to 2.2)	90.6% (85.5 to 94.2)	4.7 (4.2 to 5.1)	89.9% (84.3 to 94.3)

Tropical Latin America	9.7 (8.9 to 10.3)	89.3% (82.5 to 93.8)	11.2 (9.9 to 12.2)	86.6% (78.1 to 92.4)
Western Europe	52.4 (47.1 to 56.6)	89.8% (82.5 to 94.6)	27.9 (23.8 to 31.7)	84.7% (75.8 to 91.4)
Western Sub-Saharan Africa	9.5 (7.6 to 12.3)	89.3% (83.5 to 93.2)	18.8 (15.8 to 22.3)	91.2% (86.0 to 94.8)

Note: Data in parentheses are 95% uncertainty intervals. Count data in 100000 are presented to one decimal places and percentage data are presented to one decimal place. Percentages and number of DALYs are not mutually exclusive: the sum of percentages and number of DALYs in the columns exceeds the totals for all risk factors combined because of overlap between various risk factors. The crude sum of population attributable fraction (PAF) of the risk factors might exceed 100% because the effects of many of these risk factors are mediated partly or wholly through another risk factor or risk factors. DALY=disability-adjusted life-year.

eTable 10. Ischemic stroke related DALYs (absolute numbers [in millions with 95% UI] and percentage [with 95% UI]) associated with risk factors and their clusters in 1990 and 2021, by countries and territories, aged ≥20 years, both sexes

Countries and territories	1990		2021	
	Absolute NO (0000s)	Percent	Absolute NO (0000s)	Percent
Afghanistan	12.7 (9.0 to 17.5)	92.0% (86.6 to 95.3)	16.4 (12.2 to 22.8)	92.0% (86.4 to 95.2)
Albania	1.5 (1.2 to 1.8)	92.3% (86.9 to 95.6)	2.6 (2.0 to 3.4)	89.5% (82.7 to 94.4)
Algeria	14.6 (11.4 to 17.9)	88.6% (81.1 to 93.1)	32.0 (24.9 to 40.0)	88.5% (80.8 to 93.5)
American Samoa	0.01 (0.01 to 0.02)	84.6% (75.6 to 90.6)	0.03 (0.02 to 0.03)	88.2% (80.9 to 93.4)
Andorra	0.02 (0.02 to 0.03)	89.3% (81.5 to 94.5)	0.03 (0.03 to 0.04)	85.9% (76.3 to 92.1)
Angola	3.3 (2.6 to 4.2)	91.2% (85.3 to 94.6)	8.9 (7.1 to 11.2)	88.2% (81.8 to 93.1)
Antigua and Barbuda	0.04 (0.04 to 0.05)	81.8% (70.8 to 89.1)	0.05 (0.04 to 0.05)	84.3% (75.0 to 90.5)
Argentina	24.0 (20.9 to 26.7)	84.9% (75.2 to 91.6)	18.6 (16.5 to 20.8)	86.8% (78.7 to 92.8)
Armenia	3.0 (2.7 to 3.3)	88.5% (81.1 to 93.3)	3.7 (3.3 to 4.1)	89.4% (82.9 to 94.0)
Australia	11.6 (10.2 to 12.9)	86.8% (78.2 to 93.2)	10.0 (8.4 to 11.6)	82.9% (72.7 to 90.5)
Austria	11.4 (9.9 to 12.4)	88.1% (79.6 to 93.7)	5.1 (4.4 to 5.9)	85.2% (76.1 to 92.1)
Azerbaijan	3.8 (3.0 to 4.6)	89.0% (81.6 to 93.7)	6.0 (4.8 to 7.5)	90.1% (83.0 to 94.4)
Bahamas	0.09 (0.07 to 0.10)	83.9% (74.7 to 90.8)	0.15 (0.12 to 0.18)	85.0% (75.9 to 91.3)
Bahrain	0.18 (0.16 to 0.20)	91.9% (86.3 to 95.4)	0.47 (0.39 to 0.55)	92.9% (86.8 to 96.2)
Bangladesh	49.6 (39.4 to 64.4)	89.9% (84.9 to 93.5)	126.7 (96.4 to 164.8)	90.9% (86.2 to 94.3)
Barbados	0.29 (0.25 to 0.32)	83.3% (73.2 to 90.2)	0.31 (0.25 to 0.37)	86.8% (78.7 to 92.4)
Belarus	23.0 (20.7 to 25.1)	91.0% (84.2 to 95.2)	20.2 (16.9 to 23.7)	89.3% (82.2 to 94.3)
Belgium	12.1 (10.6 to 13.2)	87.7% (79.1 to 93.6)	6.4 (5.4 to 7.3)	86.3% (77.9 to 92.4)
Belize	0.04 (0.03 to 0.04)	84.4% (75.9 to 90.2)	0.10 (0.08 to 0.11)	85.4% (77.4 to 91.5)
Benin	2.5 (2.1 to 3.1)	88.9% (82.6 to 93.1)	5.3 (4.3 to 6.6)	90.5% (85.0 to 94.2)
Bermuda	0.04 (0.03 to 0.05)	82.2% (72.0 to 89.4)	0.04 (0.03 to 0.05)	82.3% (72.5 to 89.6)
Bhutan	0.15 (0.10 to 0.19)	91.4% (86.2 to 94.8)	0.33 (0.26 to 0.41)	87.8% (79.9 to 93.0)
Bolivia (Plurinational State of)	2.1 (1.5 to 2.7)	86.2% (78.8 to 91.4)	3.4 (2.4 to 4.7)	84.4% (76.3 to 90.7)

Bosnia and Herzegovina	7.3 (6.4 to 8.4)	94.1% (89.9 to 97.0)	9.1 (7.4 to 10.7)	91.1% (85.0 to 95.3)
Botswana	0.59 (0.45 to 0.72)	90.5% (85.1 to 94.3)	1.1 (0.89 to 1.3)	88.0% (81.2 to 93.3)
Brazil	95.2 (87.1 to 101.2)	89.3% (82.5 to 93.8)	108.7 (96.4 to 118.7)	86.6% (78.0 to 92.4)
Brunei Darussalam	0.11 (0.09 to 0.12)	86.7% (79.4 to 92.1)	0.15 (0.13 to 0.17)	85.8% (77.0 to 91.6)
Bulgaria	27.0 (24.8 to 28.9)	91.6% (85.0 to 95.8)	31.4 (27.2 to 35.9)	89.2% (82.7 to 94.3)
Burkina Faso	2.9 (2.2 to 3.7)	90.1% (84.5 to 93.7)	6.2 (4.9 to 7.9)	90.7% (84.8 to 94.1)
Burundi	2.9 (2.1 to 3.8)	88.5% (82.5 to 92.8)	3.3 (2.5 to 4.3)	89.9% (84.0 to 93.7)
Cabo Verde	0.20 (0.17 to 0.25)	89.3% (83.7 to 93.5)	0.45 (0.37 to 0.54)	89.4% (83.2 to 93.9)
Cambodia	4.5 (3.7 to 5.4)	90.5% (85.1 to 94.0)	10.9 (8.8 to 13.0)	89.9% (84.6 to 94.2)
Cameroon	3.7 (2.9 to 4.8)	86.3% (80.0 to 91.0)	12.4 (9.4 to 16.6)	91.2% (86.0 to 94.7)
Canada	15.8 (13.9 to 17.6)	85.3% (77.2 to 91.9)	16.7 (14.2 to 19.4)	81.4% (72.3 to 89.0)
Central African Republic	1.1 (0.81 to 1.5)	91.8% (86.9 to 95.2)	1.8 (1.3 to 2.5)	91.9% (86.6 to 95.1)
Chad	3.0 (2.2 to 4.1)	90.3% (84.1 to 93.9)	6.5 (5.0 to 8.8)	91.5% (86.3 to 94.8)
Chile	8.2 (7.5 to 8.8)	90.8% (84.1 to 95.1)	9.1 (8.2 to 10.1)	89.6% (82.8 to 94.4)
China	892.7 (758.5 to 1060.7)	91.2% (86.0 to 94.7)	2097.8 (1771.3 to 2433.9)	89.7% (83.6 to 94.1)
Colombia	9.5 (8.8 to 10.3)	87.8% (80.7 to 93.0)	13.0 (10.9 to 15.0)	86.8% (79.1 to 92.6)
Comoros	0.18 (0.14 to 0.23)	88.8% (82.6 to 93.1)	0.34 (0.27 to 0.42)	89.8% (83.8 to 94.0)
Congo	1.2 (0.9 to 1.5)	91.4% (85.8 to 94.8)	2.3 (1.9 to 2.9)	90.6% (84.8 to 94.4)
Cook Islands	0.01 (0.01 to 0.01)	82.2% (72.9 to 89.1)	0.01 (0.01 to 0.01)	87.2% (79.3 to 92.7)
Costa Rica	0.67 (0.60 to 0.73)	88.7% (82.0 to 93.6)	1.3 (1.1 to 1.5)	86.6% (78.7 to 92.4)
Coted'Ivoire	4.7 (3.8 to 5.7)	90.4% (85.1 to 94.0)	12.5 (9.6 to 16.4)	91.8% (86.6 to 95.2)
Croatia	10.8 (9.9 to 11.6)	91.8% (85.8 to 95.9)	6.9 (6.0 to 7.8)	89.0% (81.6 to 94.0)
Cuba	6.0 (5.2 to 6.6)	83.5% (73.7 to 89.9)	9.9 (8.4 to 11.3)	83.0% (74.0 to 89.7)
Cyprus	0.73 (0.61 to 0.86)	87.8% (79.3 to 93.7)	0.61 (0.50 to 0.73)	86.0% (77.8 to 92.2)
Czechia	32.0 (29.2 to 34.8)	90.5% (83.7 to 95.3)	11.5 (10.0 to 13.0)	89.0% (81.9 to 94.2)
Democratic People's Republic of Korea	18.1 (14.2 to 23.5)	92.3% (87.8 to 95.4)	39.4 (31.2 to 49.8)	92.0% (87.0 to 95.6)
Democratic Republic of the Congo	12.4 (9.2 to 16.3)	92.2% (87.6 to 95.3)	25.0 (18.0 to 34.9)	91.1% (85.5 to 94.4)
Denmark	6.5 (5.9 to 7.1)	90.9% (83.8 to 95.4)	3.6 (3.1 to 4.1)	86.9% (78.9 to 92.9)
Djibouti	0.09 (0.07 to 0.13)	88.0% (81.6 to 92.7)	0.43 (0.33 to 0.58)	89.3% (82.8 to 93.6)
Dominica	0.05 (0.04 to 0.06)	85.3% (77.1 to 91.6)	0.05 (0.05 to 0.06)	84.3% (75.2 to 90.8)

Dominican Republic	1.9 (1.6 to 2.2)	86.4% (78.7 to 91.9)	5.2 (4.1 to 6.6)	84.4% (75.4 to 90.9)
Ecuador	2.7 (2.3 to 3.0)	83.4% (74.5 to 89.9)	4.1 (3.3 to 5.0)	81.4% (72.0 to 88.9)
Egypt	55.7 (40.9 to 77.5)	87.5% (80.6 to 92.5)	114.2 (89.4 to 144.3)	92.0% (86.3 to 95.6)
El Salvador	1.2 (1.0 to 1.3)	87.8% (80.9 to 92.6)	1.6 (1.3 to 1.9)	85.2% (76.9 to 91.5)
Equatorial Guinea	0.19 (0.14 to 0.25)	91.5% (86.2 to 95.1)	0.37 (0.26 to 0.50)	88.8% (82.3 to 93.5)
Eritrea	0.81 (0.57 to 1.1)	87.3% (80.5 to 92.2)	1.8 (1.4 to 2.4)	87.9% (81.3 to 92.7)
Estonia	4.3 (3.9 to 4.7)	89.8% (82.0 to 95.1)	1.3 (1.1 to 1.5)	88.0% (79.8 to 93.6)
Eswatini	0.25 (0.20 to 0.32)	89.7% (84.0 to 93.6)	0.51 (0.37 to 0.70)	89.6% (83.4 to 94.0)
Ethiopia	9.2 (6.3 to 13.8)	84.7% (77.4 to 89.8)	16.4 (13.2 to 21.1)	86.6% (80.3 to 91.2)
Fiji	0.25 (0.21 to 0.29)	89.3% (82.2 to 93.4)	0.46 (0.38 to 0.56)	90.8% (84.3 to 94.8)
Finland	6.8 (6.1 to 7.4)	90.6% (83.8 to 95.3)	4.5 (3.8 to 5.1)	84.8% (75.4 to 91.8)
France	50.8 (44.6 to 55.6)	87.1% (78.3 to 93.1)	35.9 (30.3 to 41.0)	84.5% (74.4 to 91.6)
Gabon	0.52 (0.41 to 0.64)	86.9% (78.7 to 92.7)	0.77 (0.60 to 1.0)	88.0% (80.0 to 93.3)
Gambia	0.43 (0.33 to 0.55)	91.8% (87.1 to 94.9)	1.3 (1.0 to 1.7)	92.5% (87.8 to 95.3)
Georgia	7.0 (5.7 to 8.2)	89.0% (81.9 to 94.0)	9.3 (8.2 to 10.4)	89.2% (82.6 to 94.0)
Germany	133.9 (119.6 to 145.9)	91.3% (84.7 to 95.6)	70.0 (60.3 to 79.1)	88.0% (79.9 to 93.6)
Ghana	9.2 (7.4 to 11.8)	91.2% (85.8 to 94.8)	25.6 (20.2 to 31.9)	91.9% (86.4 to 95.3)
Greece	18.8 (16.7 to 20.3)	89.2% (82.0 to 94.1)	11.7 (9.9 to 13.3)	82.6% (74.1 to 89.7)
Greenland	0.03 (0.03 to 0.04)	86.1% (76.6 to 92.4)	0.03 (0.02 to 0.03)	83.8% (74.0 to 91.2)
Grenada	0.11 (0.09 to 0.12)	85.2% (75.7 to 91.3)	0.07 (0.06 to 0.08)	85.6% (76.2 to 91.8)
Guam	0.04 (0.03 to 0.05)	81.1% (70.6 to 88.3)	0.07 (0.06 to 0.09)	86.7% (78.0 to 92.3)
Guatemala	1.2 (1.1 to 1.3)	86.0% (79.3 to 90.9)	2.6 (2.2 to 2.9)	85.9% (78.6 to 91.6)
Guinea	3.5 (2.6 to 4.5)	88.4% (82.1 to 92.4)	6.6 (5.0 to 8.5)	89.9% (84.4 to 93.7)
Guinea-Bissau	0.65 (0.48 to 0.86)	90.1% (84.6 to 93.8)	1.1 (0.83 to 1.4)	91.7% (87.0 to 94.7)
Guyana	0.63 (0.56 to 0.71)	87.7% (80.2 to 92.9)	0.59 (0.47 to 0.73)	87.5% (80.0 to 92.6)
Haiti	4.4 (3.3 to 5.6)	90.4% (84.3 to 94.4)	7.4 (5.2 to 10.6)	91.6% (86.4 to 95.1)
Honduras	1.2 (0.9 to 1.5)	89.2% (83.4 to 93.2)	4.3 (3.3 to 5.5)	89.1% (82.9 to 93.7)
Hungary	31.5 (29.1 to 33.5)	93.6% (87.9 to 97.0)	15.1 (13.0 to 17.1)	91.1% (84.7 to 95.5)
Iceland	0.17 (0.15 to 0.19)	85.8% (76.1 to 92.3)	0.13 (0.11 to 0.15)	84.3% (74.9 to 91.2)
India	258.3 (210.6 to 328.8)	91.9% (86.9 to 94.9)	602.2 (509.2 to 775.4)	90.8% (85.2 to 94.4)

Indonesia	104.2 (87.1 to 121.9)	91.9% (87.2 to 95.0)	301.3 (228.1 to 374.4)	90.9% (85.0 to 94.9)
Iran (Islamic Republic of)	34.5 (30.7 to 38.6)	87.9% (80.1 to 92.9)	59.8 (53.1 to 65.9)	88.4% (81.4 to 93.2)
Iraq	17.1 (14.3 to 20.2)	92.4% (87.3 to 95.6)	40.7 (32.2 to 49.4)	93.5% (88.7 to 96.5)
Ireland	3.5 (3.1 to 3.8)	89.8% (82.3 to 94.6)	1.6 (1.3 to 1.8)	85.4% (76.3 to 91.8)
Israel	2.6 (2.3 to 2.9)	88.3% (80.8 to 93.5)	2.5 (2.2 to 2.9)	86.3% (78.1 to 91.9)
Italy	78.0 (69.6 to 84.7)	90.9% (84.4 to 95.3)	44.2 (36.1 to 51.1)	82.0% (72.2 to 89.0)
Jamaica	1.7 (1.5 to 1.9)	83.7% (75.6 to 90.2)	2.0 (1.5 to 2.4)	81.8% (72.1 to 89.1)
Japan	128.0 (114.5 to 139.5)	89.3% (83.0 to 94.2)	117.6 (97.3 to 136.1)	83.6% (75.6 to 90.5)
Jordan	2.1 (1.7 to 2.5)	89.3% (82.2 to 93.6)	5.7 (4.6 to 6.7)	90.9% (84.7 to 95.0)
Kazakhstan	24.3 (21.4 to 27.1)	90.7% (83.8 to 95.0)	24.6 (21.1 to 27.9)	91.3% (85.3 to 95.3)
Kenya	4.2 (3.4 to 5.3)	88.0% (82.3 to 92.0)	11.9 (9.4 to 14.5)	88.9% (83.2 to 93.1)
Kiribati	0.03 (0.03 to 0.04)	91.2% (86.5 to 94.3)	0.06 (0.05 to 0.07)	91.5% (86.1 to 95.0)
Kuwait	0.38 (0.34 to 0.43)	91.7% (85.8 to 95.2)	1.3 (1.1 to 1.5)	93.0% (87.6 to 96.3)
Kyrgyzstan	5.3 (4.7 to 5.9)	89.4% (82.3 to 93.9)	5.3 (4.4 to 6.2)	91.4% (85.2 to 95.3)
Lao People's Democratic Republic	3.3 (2.7 to 4.1)	92.6% (88.1 to 95.5)	5.0 (3.9 to 6.2)	91.1% (85.6 to 94.9)
Latvia	8.1 (7.4 to 8.7)	90.7% (84.1 to 95.2)	5.8 (5.0 to 6.5)	88.4% (80.5 to 93.8)
Lebanon	2.2 (1.8 to 2.9)	87.6% (79.5 to 92.8)	3.0 (2.5 to 3.5)	87.9% (80.7 to 93.1)
Lesotho	0.64 (0.50 to 0.82)	88.9% (82.9 to 93.2)	1.2 (0.9 to 1.6)	90.0% (84.6 to 94.1)
Liberia	1.3 (1.1 to 1.6)	90.6% (84.7 to 94.2)	2.2 (1.7 to 2.9)	92.0% (86.9 to 95.1)
Libya	1.5 (1.2 to 2.0)	88.1% (81.4 to 92.7)	4.9 (3.7 to 6.6)	91.1% (84.9 to 95.0)
Lithuania	6.0 (5.4 to 6.5)	91.1% (84.0 to 95.7)	5.4 (4.6 to 6.1)	88.5% (80.3 to 94.1)
Luxembourg	0.65 (0.58 to 0.70)	88.3% (80.2 to 93.9)	0.25 (0.22 to 0.29)	86.1% (77.5 to 92.4)
Madagascar	5.4 (4.4 to 6.4)	87.1% (80.9 to 91.7)	10.2 (7.7 to 13.1)	89.5% (83.5 to 93.3)
Malawi	3.0 (2.5 to 3.6)	89.5% (84.3 to 93.4)	6.6 (5.3 to 8.3)	91.3% (86.0 to 94.6)
Malaysia	8.5 (7.5 to 9.7)	90.7% (84.0 to 94.9)	20.2 (17.7 to 22.9)	91.4% (85.1 to 95.3)
Maldives	0.10 (0.09 to 0.12)	92.5% (87.5 to 95.6)	0.18 (0.15 to 0.21)	87.7% (79.9 to 93.1)
Mali	3.1 (2.3 to 4.3)	90.2% (84.6 to 93.9)	6.6 (5.0 to 9.0)	90.9% (85.8 to 94.2)
Malta	0.35 (0.31 to 0.38)	88.3% (80.7 to 93.9)	0.23 (0.19 to 0.26)	84.7% (75.5 to 91.6)
Marshall Islands	0.01 (0.01 to 0.02)	87.4% (80.0 to 92.3)	0.03 (0.02 to 0.03)	90.9% (84.4 to 94.8)
Mauritania	1.4 (1.1 to 1.8)	91.8% (86.7 to 94.9)	2.3 (1.7 to 3.2)	92.1% (86.6 to 95.5)

Mauritius	1.2 (1.1 to 1.3)	89.2% (81.8 to 93.8)	1.1 (1.0 to 1.2)	88.9% (81.3 to 93.7)
Mexico	21.5 (19.7 to 22.9)	86.6% (79.7 to 91.8)	32.7 (28.4 to 37.1)	85.9% (77.7 to 91.7)
Micronesia (Federated States of)	0.05 (0.04 to 0.06)	89.6% (83.0 to 93.7)	0.06 (0.04 to 0.07)	88.9% (81.5 to 93.7)
Monaco	0.08 (0.06 to 0.10)	86.3% (78.0 to 92.6)	0.05 (0.04 to 0.06)	84.7% (76.0 to 91.5)
Mongolia	0.49 (0.40 to 0.59)	92.3% (86.6 to 95.2)	1.1 (0.84 to 1.3)	93.1% (87.4 to 96.3)
Montenegro	0.36 (0.30 to 0.43)	92.6% (85.9 to 96.3)	0.75 (0.60 to 0.9)	90.2% (83.5 to 94.8)
Morocco	21.4 (16.2 to 27.8)	90.6% (84.2 to 94.5)	47.0 (35.8 to 59.9)	90.4% (83.9 to 94.7)
Mozambique	6.1 (5.1 to 7.4)	90.0% (84.0 to 94.0)	14.6 (10.9 to 18.5)	91.9% (86.2 to 95.1)
Myanmar	35.2 (26.7 to 43.7)	93.0% (88.9 to 95.6)	51.4 (41.3 to 63.5)	91.3% (86.3 to 94.9)
Namibia	0.72 (0.60 to 0.85)	90.9% (85.2 to 94.8)	1.3 (1.0 to 1.6)	87.9% (80.8 to 93.3)
Nauru	0.01 (0.00 to 0.01)	88.3% (79.7 to 93.3)	0.01 (0.01 to 0.01)	89.9% (82.4 to 94.3)
Nepal	6.9 (4.9 to 9.2)	90.3% (85.4 to 93.7)	13.1 (9.8 to 18.0)	88.5% (83.0 to 92.6)
Netherlands	14.2 (12.4 to 15.6)	88.3% (80.6 to 93.6)	11.1 (9.5 to 12.5)	85.6% (77.2 to 92.1)
New Zealand	2.5 (2.2 to 2.7)	88.3% (80.3 to 93.8)	2.3 (1.9 to 2.7)	84.0% (74.2 to 90.9)
Nicaragua	0.65 (0.57 to 0.74)	90.2% (84.6 to 93.9)	1.2 (1.0 to 1.5)	89.7% (83.5 to 94.0)
Niger	2.1 (1.5 to 3.1)	89.7% (83.5 to 93.5)	6.1 (4.4 to 8.6)	90.7% (85.4 to 94.1)
Nigeria	47.3 (34.6 to 65.0)	88.3% (81.9 to 92.7)	74.3 (58.9 to 93.5)	90.9% (85.4 to 94.6)
Niue	0.002 (0.002 to 0.003)	84.4% (75.3 to 90.7)	0.002 (0.001 to 0.002)	87.4% (80.2 to 93.0)
North Macedonia	5.0 (4.5 to 5.5)	93.8% (88.7 to 96.8)	7.4 (6.0 to 8.8)	91.1% (84.9 to 95.3)
Northern Mariana Islands	0.01 (0.01 to 0.01)	85.0% (75.8 to 90.9)	0.02 (0.02 to 0.03)	88.1% (80.5 to 93.4)
Norway	6.2 (5.5 to 6.8)	89.1% (82.2 to 94.2)	2.7 (2.3 to 3.1)	84.5% (76.1 to 91.3)
Oman	0.85 (0.64 to 1.1)	87.3% (79.0 to 92.8)	1.6 (1.3 to 1.9)	91.8% (85.7 to 95.7)
Pakistan	40.9 (31.2 to 53.3)	92.3% (87.2 to 95.4)	85.7 (69.1 to 109.7)	92.3% (86.8 to 95.5)
Palau	0.01 (0.01 to 0.01)	82.8% (73.2 to 89.7)	0.02 (0.01 to 0.02)	88.8% (81.4 to 93.6)
Palestine	1.5 (1.2 to 1.9)	87.5% (80.6 to 92.5)	2.5 (2.1 to 2.9)	87.9% (80.1 to 92.9)
Panama	0.75 (0.66 to 0.84)	86.2% (78.6 to 91.7)	1.4 (1.1 to 1.7)	84.6% (75.8 to 90.8)
Papua New Guinea	1.0 (0.71 to 1.4)	88.1% (81.7 to 92.6)	2.5 (1.9 to 3.4)	88.3% (82.1 to 92.6)
Paraguay	1.7 (1.4 to 1.9)	90.5% (83.6 to 94.8)	3.0 (2.4 to 3.7)	87.8% (79.1 to 93.3)
Peru	4.2 (3.6 to 4.9)	83.7% (75.8 to 89.4)	7.7 (6.2 to 9.6)	85.3% (77.1 to 91.4)
Philippines	20.5 (17.8 to 22.6)	90.1% (84.3 to 93.8)	58.4 (50.3 to 67.4)	88.1% (81.0 to 93.0)

Poland	76.7 (71.2 to 81.3)	91.6% (85.6 to 95.5)	49.0 (43.2 to 54.8)	87.1% (79.7 to 92.5)
Portugal	26.2 (23.5 to 28.4)	86.6% (78.4 to 92.8)	10.2 (8.5 to 11.6)	82.6% (72.4 to 90.1)
Puerto Rico	1.3 (1.2 to 1.5)	82.6% (72.8 to 89.8)	1.2 (1.0 to 1.4)	83.0% (73.6 to 90.1)
Qatar	0.10 (0.08 to 0.12)	92.2% (86.9 to 95.6)	0.39 (0.32 to 0.47)	93.4% (88.0 to 96.5)
Republic of Korea	41.0 (36.0 to 46.1)	88.3% (81.5 to 93.4)	36.3 (30.4 to 42.3)	83.8% (75.5 to 90.1)
Republic of Moldova	4.8 (4.2 to 5.3)	91.5% (85.9 to 95.6)	5.7 (5.2 to 6.5)	91.1% (84.6 to 95.5)
Romania	55.4 (49.9 to 60.6)	91.8% (85.1 to 95.8)	53.0 (46.4 to 59.3)	89.4% (82.3 to 94.5)
Russian Federation	461.3 (422.7 to 489.2)	90.0% (82.8 to 94.6)	363.8 (324.1 to 398.1)	88.2% (80.3 to 93.5)
Rwanda	3.2 (2.5 to 4.1)	88.5% (82.6 to 92.7)	3.7 (2.8 to 4.7)	89.2% (83.4 to 93.1)
Saint Kitts and Nevis	0.07 (0.06 to 0.08)	82.3% (72.2 to 89.7)	0.05 (0.04 to 0.06)	83.7% (74.1 to 90.5)
Saint Lucia	0.12 (0.11 to 0.13)	87.3% (79.7 to 92.7)	0.15 (0.12 to 0.18)	84.2% (74.8 to 90.4)
Saint Vincent and the Grenadines	0.07 (0.06 to 0.08)	83.8% (74.5 to 90.5)	0.08 (0.07 to 0.09)	83.8% (75.6 to 90.0)
Samoa	0.06 (0.05 to 0.08)	89.9% (84.5 to 93.9)	0.10 (0.08 to 0.12)	90.3% (84.2 to 94.3)
San Marino	0.02 (0.02 to 0.03)	87.5% (79.4 to 93.2)	0.02 (0.02 to 0.03)	85.1% (76.3 to 91.9)
Sao Tome and Principe	0.07 (0.06 to 0.08)	90.0% (84.0 to 94.1)	0.13 (0.10 to 0.15)	90.0% (83.6 to 94.2)
Saudi Arabia	8.0 (6.1 to 10.0)	88.3% (81.4 to 92.9)	19.8 (15.9 to 24.3)	92.4% (86.5 to 96.0)
Senegal	4.2 (3.5 to 5.2)	92.1% (87.2 to 95.1)	8.9 (6.9 to 11.7)	91.9% (86.9 to 95.2)
Serbia	28.7 (25.1 to 32.1)	92.8% (87.2 to 96.7)	31.2 (26.4 to 36.3)	90.7% (84.3 to 95.2)
Seychelles	0.06 (0.05 to 0.07)	87.2% (79.6 to 92.4)	0.08 (0.06 to 0.09)	88.7% (81.1 to 93.7)
Sierra Leone	2.9 (2.3 to 3.5)	92.7% (88.1 to 95.7)	4.7 (3.6 to 6.0)	92.5% (87.9 to 95.5)
Singapore	2.0 (1.8 to 2.2)	88.8% (81.7 to 94.1)	1.4 (1.1 to 1.6)	81.4% (70.3 to 89.0)
Slovakia	9.8 (8.7 to 11.2)	91.7% (84.9 to 96.0)	8.0 (6.7 to 9.4)	89.6% (82.4 to 94.4)
Slovenia	3.2 (2.9 to 3.4)	91.4% (84.9 to 95.6)	1.9 (1.6 to 2.1)	87.2% (78.7 to 92.9)
Solomon Islands	0.10 (0.08 to 0.13)	89.3% (82.9 to 93.2)	0.26 (0.20 to 0.35)	90.5% (84.7 to 94.1)
Somalia	1.6 (1.1 to 2.2)	87.3% (81.1 to 91.7)	3.3 (2.3 to 4.9)	89.5% (83.6 to 93.4)
South Africa	14.9 (12.2 to 16.9)	90.5% (85.3 to 94.3)	36.0 (32.0 to 40.3)	89.5% (83.3 to 94.2)
South Sudan	1.9 (1.4 to 2.5)	85.1% (78.0 to 90.3)	2.2 (1.7 to 3.0)	89.2% (83.5 to 93.2)
Spain	48.7 (43.0 to 52.7)	89.1% (81.3 to 94.3)	25.5 (21.4 to 29.4)	83.4% (74.1 to 90.4)
Sri Lanka	12.5 (11.2 to 13.9)	90.6% (85.1 to 94.3)	23.5 (16.9 to 29.6)	88.1% (80.9 to 93.4)
Sudan	15.1 (10.9 to 19.8)	92.7% (87.7 to 95.7)	25.1 (17.8 to 33.6)	92.8% (87.8 to 95.8)

Suriname	0.20 (0.17 to 0.22)	85.9% (77.6 to 91.8)	0.37 (0.29 to 0.46)	85.2% (76.5 to 91.3)
Sweden	11.2 (9.9 to 12.4)	87.9% (79.6 to 93.8)	6.2 (5.2 to 7.2)	84.8% (75.5 to 91.8)
Switzerland	6.4 (5.7 to 7.1)	87.6% (79.5 to 93.5)	3.7 (3.1 to 4.3)	84.4% (74.6 to 91.2)
Syrian Arab Republic	6.5 (5.3 to 7.9)	87.5% (80.1 to 92.2)	11.8 (9.2 to 15.2)	89.6% (82.1 to 94.2)
Taiwan (Province of China)	13.6 (12.3 to 14.9)	88.9% (81.6 to 94.1)	13.6 (11.6 to 15.7)	84.6% (74.9 to 91.0)
Tajikistan	3.8 (3.2 to 4.5)	90.7% (84.8 to 94.5)	5.7 (4.6 to 6.9)	90.8% (84.5 to 94.7)
Thailand	25.1 (21.1 to 29.7)	89.9% (84.2 to 94.1)	53.4 (43.1 to 65.9)	87.1% (79.9 to 92.6)
Timor-Leste	0.22 (0.18 to 0.26)	90.9% (85.9 to 94.2)	0.85 (0.62 to 1.1)	90.2% (84.5 to 94.5)
Togo	1.5 (1.2 to 1.9)	92.1% (87.4 to 95.2)	4.5 (3.3 to 5.7)	93.1% (88.3 to 95.9)
Tokelau	0.001 (0.001 to 0.001)	81.0% (70.9 to 88.3)	0.001 (0.001 to 0.001)	86.4% (78.0 to 92.1)
Tonga	0.03 (0.02 to 0.03)	89.3% (83.5 to 93.4)	0.04 (0.03 to 0.05)	89.0% (82.5 to 94.1)
Trinidad and Tobago	0.9 (0.81 to 1.0)	87.0% (77.7 to 92.5)	1.2 (0.9 to 1.4)	87.5% (80.1 to 93.0)
Tunisia	5.0 (3.9 to 6.3)	87.4% (79.7 to 92.5)	10.9 (7.7 to 14.6)	87.1% (78.8 to 92.6)
Turkey	39.3 (33.1 to 45.8)	88.8% (81.8 to 93.5)	58.2 (48.1 to 70.1)	87.5% (80.2 to 92.6)
Turkmenistan	2.6 (2.1 to 3.0)	88.1% (80.6 to 93.2)	6.5 (5.0 to 8.2)	90.5% (82.8 to 94.9)
Tuvalu	0.01 (0.01 to 0.01)	87.2% (78.8 to 92.6)	0.01 (0.01 to 0.01)	86.2% (77.5 to 92.0)
Uganda	4.6 (3.7 to 5.9)	89.9% (84.2 to 93.6)	8.0 (6.3 to 10.0)	89.2% (83.4 to 93.2)
Ukraine	161.5 (147.3 to 173.2)	91.0% (83.8 to 95.6)	102.6 (82.2 to 126.6)	88.9% (81.1 to 94.2)
United Arab Emirates	0.62 (0.50 to 0.77)	92.9% (87.7 to 96.0)	2.6 (2.1 to 3.1)	93.8% (87.9 to 97.1)
United Kingdom	84.3 (76.2 to 90.6)	90.7% (83.9 to 95.2)	32.8 (28.1 to 37.2)	82.9% (73.5 to 90.2)
United Republic of Tanzania	6.3 (5.0 to 8.1)	88.2% (82.3 to 92.6)	18.4 (14.0 to 23.7)	89.5% (84.0 to 93.7)
United States of America	158.2 (138.1 to 177.2)	87.6% (79.7 to 93.2)	182.0 (153.9 to 208.0)	84.1% (75.6 to 90.5)
United States Virgin Islands	0.04 (0.03 to 0.05)	82.2% (71.1 to 89.8)	0.05 (0.04 to 0.06)	83.1% (74.1 to 90.2)
Uruguay	4.2 (3.8 to 4.7)	86.5% (77.4 to 92.6)	3.0 (2.6 to 3.4)	84.9% (75.6 to 91.7)
Uzbekistan	13.5 (11.8 to 14.9)	87.6% (79.4 to 92.7)	26.7 (23.0 to 30.8)	90.3% (83.2 to 94.6)
Vanuatu	0.06 (0.05 to 0.08)	92.4% (87.7 to 95.4)	0.14 (0.12 to 0.18)	92.9% (88.2 to 95.8)
Venezuela (Bolivarian Republic of)	4.3 (3.8 to 4.8)	87.5% (80.3 to 92.6)	10.6 (8.3 to 13.4)	85.5% (77.1 to 91.6)
Viet Nam	51.2 (41.5 to 63.4)	90.8% (85.8 to 94.5)	127.7 (101.3 to 153.7)	90.4% (84.5 to 94.7)
Yemen	8.1 (5.9 to 11.1)	91.2% (85.3 to 95.1)	21.7 (15.3 to 29.3)	90.4% (84.3 to 94.4)
Zambia	2.0 (1.5 to 2.7)	86.9% (81.0 to 91.2)	5.5 (4.2 to 7.2)	88.7% (82.9 to 93.0)

Zimbabwe	2.7 (2.3 to 3.2)	91.7% (86.7 to 94.9)	6.5 (5.3 to 7.9)	92.9% (87.9 to 95.9)

Note: Data in parentheses are 95% uncertainty intervals. Count data in 100000 are presented to one decimal places and percentage data are presented to one decimal place. Percentages and number of DALYs are not mutually exclusive: the sum of percentages and number of DALYs in the columns exceeds the totals for all risk factors combined because of overlap between various risk factors. The crude sum of population attributable fraction (PAF) of the risk factors might exceed 100% because the effects of many of these risk factors are mediated partly or wholly through another risk factor or risk factors. DALY=disability-adjusted life-year.

eTable 11a. Percent of DALYs (with 95% uncertainty intervals) due to IS for environmental risks by country and territory for both sexes combined in 2021, aged ≥20 years

	**Environmental risks**	Ambient particulate matter pollution	Household air pollution from solid fuels	Low temperature	High temperature	Lead exposure
Afghanistan	57.28% (46.19 to 66.39)	46.16% (38.36 to 53.98)	39.12% (32.35 to 46.32)	7.21% (6.13 to 8.15)	0.96% (-0.34 to 2.8)	13.95% (-1.95 to 30.05)
Albania	26.48% (18.85 to 35.58)	15.54% (9.8 to 25.59)	4.38% (0.29 to 17.02)	7.79% (6.8 to 8.98)	0.08% (-0.27 to 0.51)	5.53% (-0.73 to 12.15)
Algeria	29.95% (21.16 to 39.29)	17.95% (12.56 to 24.2)	0.03% (0 to 0.22)	6.08% (5.29 to 6.85)	1.3% (-0.03 to 3.14)	7.95% (-1.05 to 17.58)
American Samoa	5.96% (1.58 to 13.31)	4.4% (0.4 to 11.58)	1.1% (0.01 to 7.25)	0.03% (-0.1 to 0.15)	0.06% (0.01 to 0.12)	1.55% (-0.2 to 3.57)
Andorra	15.77% (11.9 to 20)	5.03% (2.77 to 7.71)	0% (0 to 0)	8.48% (7.22 to 9.57)	0% (-0.02 to 0)	3.09% (-0.4 to 7.1)
Angola	35.25% (24.53 to 45.59)	29.3% (19.6 to 39.01)	14.89% (4.86 to 27.6)	1.34% (1.1 to 1.74)	0.18% (-0.15 to 0.67)	7.04% (-0.96 to 16)
Antigua and Barbuda	18.84% (8.48 to 30.64)	13.78% (5.04 to 24.82)	0.05% (0 to 0.46)	0.02% (-0.09 to 0.13)	0.34% (0.22 to 0.5)	5.53% (-0.69 to 12.5)
Argentina	18.57% (12.41 to 26.19)	10.23% (5.26 to 16.86)	0.18% (0 to 1.57)	5.27% (4.73 to 5.81)	0.17% (-0.19 to 0.7)	4.1% (-0.54 to 9.13)
Armenia	33.09% (25.03 to 41.94)	24.09% (16.59 to 33.09)	1.02% (0.11 to 4)	7.54% (6.31 to 8.55)	0.12% (-0.59 to 0.95)	4.55% (-0.57 to 10.38)
Australia	15.07% (8.3 to 22.58)	5.37% (3.23 to 7.9)	0% (0 to 0.03)	4.48% (3.78 to 5.4)	0.12% (-0.09 to 0.46)	5.94% (-0.79 to 13.48)
Austria	16.12% (11.89 to 20.53)	7.42% (5.26 to 10.04)	0% (0 to 0.01)	6.15% (5.22 to 7.16)	0.01% (-0.2 to 0.26)	3.45% (-0.44 to 7.88)
Azerbaijan	25.8% (17.4 to 34.71)	16.76% (8.66 to 26.74)	0.74% (0.01 to 4.74)	6.92% (5.63 to 7.88)	0.19% (-0.49 to 0.93)	4.07% (-0.51 to 8.99)
Bahamas	17.28% (7.75 to 28.89)	13.3% (4.57 to 26.16)	0.04% (0 to 0.34)	0.37% (0.07 to 0.66)	0.67% (-0.16 to 1.79)	3.6% (-0.45 to 8.49)
Bahrain	44.08% (34.86 to 51.93)	36.2% (28.25 to 44.39)	0.01% (0 to 0.04)	2.1% (0.62 to 3.69)	5.61% (1.92 to 9.89)	5.28% (-0.68 to 11.9)
Bangladesh	55.15% (44.71 to 64.31)	47.16% (39.39 to 54.83)	38.72% (30.15 to 46.93)	1.7% (0.59 to 2.92)	2.14% (0.16 to 4.64)	11.83% (-1.71 to 25.41)
Barbados	21.24% (10.53 to 34.71)	16.98% (6.85 to 30.07)	0.01% (0 to 0.08)	0% (-0.04 to 0.05)	0.38% (0.23 to 0.57)	4.77% (-0.63 to 10.97)
Belarus	21.99% (16.76 to 27)	11.25% (8.11 to 15.28)	0.07% (0 to 0.57)	9% (6.94 to 10.41)	-0.18% (-0.6 to 0.02)	3.58% (-0.45 to 8.42)
Belgium	19.68% (12.17 to 27.81)	7.18% (4.98 to 9.7)	0% (0 to 0.01)	6.83% (5.88 to 8.9)	0.01% (-0.05 to 0.11)	7.12% (-0.94 to 15.9)
Belize	27.12% (14.96 to 42.12)	21.94% (10.48 to 35.72)	4.45% (0.74 to 12.19)	0.26% (0.1 to 0.41)	0.95% (0.63 to 1.48)	5.53% (-0.71 to 12.51)
Benin	54.18% (45.6 to 62.24)	49.12% (41.1 to 56.73)	40.07% (32.13 to 47.79)	0.08% (0.01 to 0.19)	2.51% (1.45 to 3.27)	7.65% (-1.04 to 16.68)
Bermuda	8.6% (3.77 to 13.59)	3.23% (0.6 to 6.19)	0.01% (0 to 0.03)	1.93% (1.55 to 2.37)	0.36% (-0.29 to 1.37)	3.34% (-0.41 to 7.4)

Bhutan	40.58% (29.36 to 51.46)	26.22% (19.4 to 34.03)	6.42% (1.21 to 17.05)	7.46% (6.7 to 8.6)	0.05% (-0.04 to 0.21)	12.98% (-1.75 to 28.36)
Bolivia (Plurinational State of)	34.77% (25.06 to 44.71)	23.85% (16.19 to 33)	8.45% (2.55 to 17.48)	6.31% (5.76 to 7.37)	0.25% (-0.13 to 0.77)	8.37% (-1.07 to 19.01)
Bosnia and Herzegovina	36.03% (26.78 to 46.66)	25.25% (17.5 to 37.4)	6.91% (0.54 to 24.84)	8.46% (7.54 to 10.32)	0.13% (-0.31 to 0.86)	6.4% (-0.84 to 14.13)
Botswana	26.61% (15.45 to 39.63)	17.47% (9.84 to 31.46)	3.63% (0.07 to 21.89)	2.87% (2.33 to 3.56)	0.54% (-0.38 to 1.97)	7.97% (-1.08 to 17.42)
Brazil	18.45% (9.58 to 26.8)	10.03% (5.8 to 15.24)	1.93% (0.43 to 5.43)	2.09% (1.72 to 2.56)	0.34% (0.17 to 0.66)	7.11% (-0.94 to 16.15)
Brunei Darussalam	8.7% (2.73 to 15.29)	3.96% (0.84 to 7.92)	0.01% (0 to 0.01)	0.03% (0 to 0.08)	0.49% (0.29 to 0.71)	4.44% (-0.58 to 10.25)
Bulgaria	24.7% (19.32 to 31.53)	13.86% (10.19 to 21.44)	1.08% (0.01 to 9.04)	8.64% (7.79 to 10.9)	0.22% (-0.37 to 1.24)	4.11% (-0.53 to 9.39)
Burkina Faso	57.91% (48.17 to 66.48)	49.3% (41.58 to 56.82)	40.46% (32.7 to 48.08)	0.24% (0.05 to 0.53)	5.51% (4 to 7.47)	12.12% (-1.62 to 26.55)
Burundi	55.59% (46.33 to 63.61)	50.2% (42.42 to 57.63)	46.66% (38.9 to 54.17)	2.28% (1.68 to 3.4)	0% (-0.02 to 0.04)	8.86% (-1.2 to 19.73)
Cabo Verde	37.48% (28.08 to 47.09)	31.58% (23.11 to 40.01)	8.17% (2.32 to 17.87)	2.16% (1.76 to 3.07)	0.05% (-0.05 to 0.24)	6.58% (-0.88 to 14.68)
Cambodia	50.34% (41.5 to 58.43)	44.83% (36.6 to 52.77)	37.79% (28.89 to 46.02)	0.42% (0.06 to 0.98)	2.15% (1.85 to 2.38)	7.66% (-0.99 to 17.05)
Cameroon	51.09% (41.26 to 59.72)	45.01% (37.06 to 52.8)	33.78% (25.12 to 42.22)	0.8% (0.58 to 1.12)	1.54% (1.06 to 2.32)	9.04% (-1.24 to 19.83)
Canada	10.6% (6.88 to 14.81)	2.32% (0.87 to 4.25)	0% (0 to 0.01)	5.76% (4.77 to 6.69)	-0.06% (-0.21 to 0.05)	2.95% (-0.38 to 6.75)
Central African Republic	55.51% (45.98 to 63.85)	50.54% (42.63 to 58.02)	45.99% (38.61 to 53.34)	0.14% (0.05 to 0.26)	0.92% (0.22 to 1.53)	9.18% (-1.2 to 20.84)
Chad	58.23% (48.2 to 67.08)	49.47% (41.91 to 56.96)	39.89% (31.56 to 47.62)	0.58% (0.14 to 1.23)	4.98% (3.27 to 7.46)	12.72% (-1.83 to 27.63)
Chile	25.28% (19.68 to 32.09)	17.28% (11.33 to 24.46)	0.65% (0 to 6.88)	7.35% (6.59 to 8.33)	0% (-0.01 to 0.01)	2.52% (-0.33 to 5.74)
China	40.74% (31.96 to 49.72)	30.35% (23.94 to 38.24)	5.21% (0.69 to 17.64)	6.58% (5.87 to 7.44)	0.44% (-0.27 to 1.68)	8.61% (-1.13 to 19.02)
Colombia	20.92% (12.55 to 29.2)	11.47% (7.11 to 17.51)	1.27% (0.05 to 6.99)	3.63% (2.95 to 4.46)	0.3% (0.27 to 0.33)	7.05% (-0.94 to 15.62)
Comoros	48.73% (39.5 to 56.71)	44.64% (36.76 to 52)	42.05% (34.59 to 49.44)	0.34% (0.09 to 0.63)	0.02% (-0.03 to 0.06)	7.09% (-0.95 to 16.32)
Congo	39.94% (29.63 to 49.97)	36.53% (26.8 to 46.06)	22.83% (10.72 to 34.66)	0.18% (0.06 to 0.32)	0.16% (0.02 to 0.41)	5.08% (-0.65 to 11.57)
Cook Islands	4.51% (1.26 to 9.58)	2.77% (0 to 7.54)	0.13% (0 to 1.4)	0% (0 to 0.01)	0.18% (-0.06 to 0.41)	1.61% (-0.2 to 3.65)
Costa Rica	18.54% (9.82 to 27.77)	9.91% (6.27 to 14.87)	0.7% (0.01 to 4.75)	2.07% (1.39 to 2.75)	0.03% (0.02 to 0.05)	7.65% (-1.05 to 17.26)
Coted'Ivoire	50.73% (41.13 to 59.38)	45.9% (38.1 to 53.73)	32.6% (22.75 to 41.9)	0.07% (-0.01 to 0.17)	0.77% (0.5 to 0.97)	8.26% (-1.12 to 18.4)
Croatia	22.06% (16.75 to 27.45)	11.67% (8.56 to 15.38)	0.23% (0 to 2.26)	7.68% (6.63 to 9.09)	0.2% (-0.47 to 1.23)	4.24% (-0.54 to 9.43)
Cuba	23.74% (12.14 to 36.21)	15.3% (7.26 to 25.3)	0.59% (0.09 to 2.1)	0.37% (0.07 to 0.66)	0.59% (0.21 to 1.13)	9.11% (-1.22 to 20.28)
Cyprus	20.96% (14.21 to 28.41)	10.65% (7.62 to 14)	0% (0 to 0.03)	4.59% (3.42 to 5.74)	1.46% (-0.39 to 4.63)	5.93% (-0.78 to 13.01)

Czechia	19.65% (15.59 to 24)	10% (7.32 to 12.98)	0.03% (0 to 0.21)	7.96% (6.84 to 9.59)	-0.02% (-0.2 to 0.1)	3.02% (-0.38 to 6.86)
Democratic People's Republic of Korea	55.27% (47.15 to 62.7)	47.8% (39.89 to 55.5)	41.92% (34.45 to 49.25)	7.31% (6.54 to 8.69)	0.24% (-0.39 to 1.38)	7.36% (-0.96 to 16.48)
Democratic Republic of the Congo	53.36% (44.35 to 61.36)	49.04% (41.16 to 56.44)	45.04% (37.6 to 52.23)	0.91% (0.76 to 1.11)	0.22% (-0.06 to 0.61)	7.53% (-1 to 16.96)
Denmark	16.4% (11.71 to 21.63)	5.64% (3.38 to 8.32)	0% (0 to 0)	7.98% (7.01 to 10.54)	0% (-0.02 to 0.02)	3.73% (-0.48 to 8.38)
Djibouti	41.9% (30.12 to 53.9)	32.26% (21.54 to 45.12)	12.18% (6.11 to 19.47)	1.51% (0.48 to 3.19)	5.94% (3.53 to 9.55)	7.54% (-1.05 to 16.68)
Dominica	20.8% (10.63 to 32.85)	16.14% (7.04 to 28.37)	1.85% (0.16 to 6.95)	0.03% (-0.1 to 0.16)	0.25% (0.13 to 0.39)	5.3% (-0.7 to 11.9)
Dominican Republic	24.75% (10.83 to 39.11)	14.84% (5.5 to 26.61)	0.94% (0.02 to 6.18)	0.6% (0.4 to 0.83)	0.18% (0.07 to 0.35)	10.96% (-1.49 to 23.8)
Ecuador	21.39% (13.84 to 29.22)	14.04% (8.69 to 20.72)	1.27% (0.1 to 5.12)	3.44% (2.95 to 4.24)	0.02% (0 to 0.07)	5.29% (-0.67 to 11.92)
Egypt	49.01% (36.7 to 60.27)	38.06% (28.91 to 46.83)	0.06% (0 to 0.28)	2.81% (1.67 to 3.76)	3.35% (-0.79 to 9.18)	12.49% (-1.8 to 27.88)
El Salvador	28.68% (15.65 to 41.78)	17.94% (11 to 25.99)	5.49% (1.18 to 13.31)	0.08% (0.04 to 0.13)	0.54% (0.42 to 0.68)	12.57% (-1.77 to 27.28)
Equatorial Guinea	27.41% (15.99 to 39.44)	22.77% (12.68 to 34.48)	0.66% (0 to 7.32)	0.36% (0.23 to 0.46)	0.02% (-0.09 to 0.07)	5.69% (-0.74 to 13.01)
Eritrea	50.48% (41.78 to 58.28)	44.52% (36.51 to 52.31)	37.41% (30.42 to 44.84)	1.73% (1.36 to 2.24)	2.25% (1.24 to 3.74)	7.17% (-0.93 to 15.92)
Estonia	13.29% (9.34 to 17.75)	3.18% (0.99 to 7.16)	0.21% (0 to 2.29)	8.02% (6.89 to 9.15)	-0.09% (-0.24 to 0.01)	2.73% (-0.35 to 6.28)
Eswatini	37.51% (24 to 50.58)	28.85% (16.09 to 42.49)	15.61% (2.97 to 34.03)	4.8% (4.18 to 5.56)	0.15% (-0.16 to 0.65)	7.59% (-0.98 to 16.54)
Ethiopia	54.62% (45.56 to 62.72)	47.39% (39.94 to 54.86)	41.55% (34.46 to 48.13)	4.04% (3.53 to 4.73)	0.21% (0.09 to 0.41)	10.17% (-1.41 to 22.22)
Fiji	18.65% (7.83 to 32.28)	16.46% (5.09 to 30.55)	7.78% (0.93 to 21.03)	0.4% (-0.17 to 0.69)	0.07% (-0.02 to 0.21)	2.16% (-0.27 to 4.94)
Finland	10.63% (7.8 to 13.73)	1.49% (0.21 to 3.33)	0% (0 to 0)	7.55% (6.02 to 8.69)	-0.07% (-0.28 to 0)	1.94% (-0.25 to 4.5)
France	16.16% (10.75 to 21.95)	5.85% (3.91 to 8.27)	0% (0 to 0.01)	6.65% (5.77 to 8.18)	0.02% (-0.1 to 0.2)	4.59% (-0.6 to 10.32)
Gabon	24.74% (15.19 to 35.06)	20.81% (12.28 to 31.28)	1.06% (0.02 to 7.39)	0.23% (0.1 to 0.38)	0.07% (0.03 to 0.13)	4.69% (-0.61 to 10.8)
Gambia	54.93% (45.96 to 63.13)	49.33% (41.47 to 56.85)	39.47% (31.57 to 47.01)	0.21% (0.03 to 0.42)	1.95% (1.21 to 2.93)	9.23% (-1.25 to 20.26)
Georgia	29.28% (20.4 to 38.67)	17.45% (10.23 to 26.94)	5.01% (0.48 to 16.55)	8.43% (7.66 to 10.52)	0.22% (-0.26 to 1.16)	6.24% (-0.83 to 14.03)
Germany	15.04% (11.48 to 19.07)	6.63% (4.51 to 9.13)	0% (0 to 0)	6.56% (5.65 to 8.49)	0.01% (-0.1 to 0.13)	2.62% (-0.34 to 6.04)
Ghana	48.91% (39.59 to 57.29)	44.85% (36.25 to 52.96)	27.38% (15.19 to 38.71)	0.06% (-0.02 to 0.16)	1.91% (1.65 to 2.31)	5.56% (-0.73 to 12.57)
Greece	21.56% (15.76 to 27.94)	10.63% (7.79 to 13.81)	0.02% (0 to 0.21)	6.8% (6.18 to 7.45)	0.28% (-0.21 to 1.06)	5.58% (-0.73 to 12.6)
Greenland	11.33% (6.09 to 17.8)	2.61% (0.1 to 6.83)	0% (0 to 0)	6.46% (3.82 to 10)	-0.15% (-0.39 to 0.01)	2.81% (-0.35 to 6.47)

Grenada	24.57% (10.67 to 38.91)	17.72% (7.23 to 32.6)	0.3% (0.01 to 1.86)	0% (-0.03 to 0.04)	0.42% (0.26 to 0.62)	7.96% (-1.03 to 18.03)
Guam	7.8% (4.5 to 12.01)	5.92% (2.81 to 9.57)	0.05% (0 to 0.27)	0% (-0.03 to 0)	0.53% (0.24 to 0.85)	1.47% (-0.19 to 3.39)
Guatemala	42.37% (27.13 to 56.66)	31.01% (19.01 to 42.55)	19.15% (5.44 to 34.84)	2.73% (1.54 to 3.64)	0.18% (0.12 to 0.29)	14.04% (-2.02 to 29.54)
Guinea	55.49% (45.97 to 64.19)	49.63% (42.01 to 57.18)	41.22% (33.37 to 48.96)	0.22% (0.07 to 0.39)	1.07% (0.58 to 1.82)	10.61% (-1.49 to 23.22)
Guinea-Bissau	55.16% (45.85 to 63.18)	49.51% (41.89 to 57.06)	40.85% (33.25 to 48.35)	0.08% (0.01 to 0.25)	2.05% (1.52 to 2.5)	9.4% (-1.29 to 21.31)
Guyana	27.47% (14.25 to 42.01)	20.34% (9 to 35.11)	2.48% (0.48 to 6.88)	0.07% (-0.03 to 0.17)	0.31% (0.04 to 0.61)	8.64% (-1.22 to 18.82)
Haiti	55.67% (43.92 to 64.83)	48.57% (40.97 to 56.07)	44.43% (36.72 to 52.21)	0.23% (0.11 to 0.36)	0.37% (0.27 to 0.48)	13.41% (-1.9 to 29.22)
Honduras	43.67% (30.78 to 54.99)	33.87% (25.15 to 41.92)	25.57% (15.45 to 34.56)	1.11% (0.5 to 1.63)	0.4% (0.09 to 0.81)	13.58% (-1.93 to 29.19)
Hungary	21.11% (15.81 to 27.67)	10.91% (7.78 to 17.23)	0.56% (0 to 6.49)	7.52% (6.43 to 8.88)	0.23% (-0.69 to 1.4)	4.03% (-0.52 to 9.19)
Iceland	13.56% (9.11 to 18.44)	1.57% (0.31 to 3.5)	0% (0 to 0)	8.74% (7.42 to 9.95)	0% (0 to 0)	3.77% (-0.47 to 8.44)
India	48.71% (38.56 to 57.94)	40.34% (33.09 to 47.83)	21.25% (12.86 to 31.92)	1.86% (0.7 to 3.26)	2.37% (0.82 to 4.36)	10.64% (-1.44 to 23.61)
Indonesia	27.48% (17.84 to 37.24)	21.46% (14.49 to 29.53)	8.84% (2.81 to 19.12)	0.34% (0.26 to 0.62)	0.36% (0.27 to 0.49)	7.05% (-0.95 to 15.89)
Iran (Islamic Republic of)	38.68% (27.82 to 49.78)	23.82% (19.01 to 28.59)	0.04% (0 to 0.21)	6.28% (5.12 to 7.35)	1.94% (-0.24 to 4.79)	12.55% (-1.78 to 27.3)
Iraq	41.62% (30.23 to 52.98)	26.4% (19.35 to 34.17)	0.14% (0 to 0.93)	5.64% (2.57 to 9.61)	9.1% (0.78 to 18.66)	7.74% (-1.02 to 17.32)
Ireland	14.44% (9.19 to 20.23)	3.93% (2 to 6.25)	0% (0 to 0)	6.88% (5.62 to 9.53)	0% (-0.03 to 0.03)	4.37% (-0.56 to 9.82)
Israel	20% (14.8 to 25.37)	14.45% (11.1 to 18.26)	0% (0 to 0.03)	2.25% (1.45 to 2.89)	1.04% (-0.48 to 3.83)	3.36% (-0.43 to 7.67)
Italy	20.99% (15.69 to 26.72)	10.04% (7.56 to 13.03)	0.01% (0 to 0.09)	7.45% (6.61 to 8.48)	0.1% (-0.28 to 0.64)	5.02% (-0.65 to 11.35)
Jamaica	22.83% (12.46 to 33.31)	15.71% (9.08 to 24.15)	3.74% (0.64 to 10.33)	0.1% (-0.03 to 0.21)	0.45% (0.13 to 0.93)	7.94% (-1.02 to 17.99)
Japan	16.6% (11.42 to 22.24)	8.57% (4.74 to 13.46)	0.01% (0 to 0.04)	5.91% (5.1 to 6.7)	0.06% (-0.16 to 0.39)	3.01% (-0.39 to 6.95)
Jordan	31.19% (23.54 to 38.87)	22.6% (17.2 to 28.59)	0.02% (0 to 0.07)	4.04% (3.2 to 4.98)	0.84% (-0.53 to 3.24)	6.64% (-0.87 to 14.88)
Kazakhstan	24.94% (18.88 to 30.99)	15.66% (10.63 to 21.8)	0.3% (0 to 3.07)	8.01% (6.88 to 9.28)	0.2% (-0.51 to 1.18)	3.07% (-0.42 to 6.79)
Kenya	49.59% (41.28 to 57.5)	44.6% (37.18 to 52.05)	39.15% (31.39 to 46.48)	3.03% (2.49 to 3.91)	0.14% (0.09 to 0.17)	6.22% (-0.81 to 14.1)
Kiribati	34.58% (27.3 to 42.63)	32.53% (25.14 to 40.71)	29.83% (23.71 to 36.51)	0.02% (-0.05 to 0.09)	0.15% (0 to 0.36)	2.9% (-0.38 to 6.69)
Kuwait	44.14% (34.44 to 53.06)	33.55% (26.56 to 40.32)	0% (0 to 0)	3.61% (0.89 to 6.77)	8.67% (1.92 to 15.79)	4.85% (-0.62 to 11.03)
Kyrgyzstan	38.11% (30.17 to 45.9)	29.06% (22.1 to 36.71)	16.79% (9.92 to 24.49)	8.43% (6.85 to 9.75)	0.03% (-0.49 to 0.62)	4.73% (-0.6 to 10.83)
Lao People's Democratic Republic	47.91% (33.65 to 59.27)	41.68% (26.37 to 53.26)	30.98% (13.51 to 46.51)	1.43% (1.01 to 1.91)	0.89% (0.21 to 1.85)	8.6% (-1.15 to 18.89)

Latvia	19.92% (15.34 to 25.11)	8.92% (5.82 to 14.64)	0.49% (0 to 5.26)	9.64% (8.04 to 10.74)	-0.09% (-0.28 to 0.02)	2.79% (-0.36 to 6.46)
Lebanon	23.59% (16.88 to 31.09)	15.23% (9.82 to 21.69)	0.06% (0 to 0.38)	4.84% (4.11 to 5.67)	0.17% (-0.33 to 0.94)	5.15% (-0.66 to 11.65)
Lesotho	48.86% (39 to 57.57)	38.86% (31.45 to 46.95)	32.94% (25.61 to 40.24)	8.05% (6.88 to 10.24)	0% (-0.01 to 0.01)	9.07% (-1.26 to 20.55)
Liberia	54.17% (44.84 to 62.64)	49.41% (41.72 to 57.01)	43.15% (36.1 to 50.12)	0.11% (0.01 to 0.23)	0.17% (0.06 to 0.37)	9.29% (-1.3 to 20.72)
Libya	34.48% (24.45 to 44.05)	25.28% (17.94 to 33.45)	0.08% (0 to 0.48)	3.41% (2.52 to 4.23)	2.66% (-0.3 to 7.52)	6.83% (-0.93 to 15.45)
Lithuania	17.33% (13.39 to 21.38)	6.36% (3.98 to 9.41)	0.08% (0 to 0.84)	9.26% (7.52 to 10.47)	-0.12% (-0.38 to 0.01)	2.82% (-0.37 to 6.49)
Luxembourg	14.92% (10.49 to 19.84)	4.49% (2.58 to 6.75)	0% (0 to 0)	7.65% (6.68 to 10.29)	0% (-0.06 to 0.08)	3.55% (-0.46 to 8.1)
Madagascar	54.48% (45.96 to 62.03)	49.62% (42.02 to 56.98)	47.33% (39.61 to 54.72)	2.92% (2.52 to 3.51)	0.24% (-0.06 to 0.71)	6.81% (-0.9 to 14.87)
Malawi	55.18% (46.16 to 63.18)	49.75% (42.15 to 57.16)	46.3% (38.83 to 53.56)	2.3% (1.85 to 2.84)	0.59% (-0.37 to 2.02)	8.29% (-1.11 to 18.86)
Malaysia	18.1% (11.32 to 25.07)	13% (8.66 to 18.39)	0.04% (0 to 0.35)	0.06% (0 to 0.13)	0.65% (0.49 to 0.84)	5.21% (-0.66 to 11.84)
Maldives	15.34% (7.89 to 23.42)	9.24% (5.12 to 15.27)	0.97% (0.01 to 6.9)	0% (-0.05 to 0.01)	0.74% (0.44 to 1.16)	6.04% (-0.79 to 13.47)
Mali	58.59% (49.11 to 67.17)	49.69% (42.1 to 57.12)	39.61% (31.43 to 47.06)	0.31% (0.08 to 0.7)	6.56% (4.29 to 9.66)	11.91% (-1.65 to 26.29)
Malta	20.61% (11.01 to 30.91)	8.47% (5.99 to 11.44)	0.01% (0 to 0.05)	3.91% (2.25 to 5.29)	0.53% (-0.53 to 2.35)	9.27% (-1.29 to 20.17)
Marshall Islands	25.98% (18.29 to 34.33)	23.43% (15.97 to 31.91)	18.69% (12.45 to 26.01)	0% (0 to 0)	0.76% (0.36 to 1.2)	2.6% (-0.31 to 5.98)
Mauritania	53.24% (44.15 to 61.45)	44.93% (36.25 to 53.12)	23.03% (14.1 to 32.31)	0.7% (0.19 to 1.43)	9.07% (4.47 to 15.36)	6.09% (-0.81 to 13.39)
Mauritius	10.07% (4.83 to 16.5)	5.37% (1.8 to 9.65)	0.06% (0 to 0.63)	1.01% (0.28 to 1.66)	0.08% (-0.12 to 0.36)	3.93% (-0.51 to 9.08)
Mexico	23.31% (13.65 to 33.2)	12.11% (7.96 to 18.43)	1.94% (0.14 to 8.19)	3.95% (3.33 to 4.54)	0.56% (-0.03 to 1.33)	8.67% (-1.16 to 19.17)
Micronesia (Federated States of)	27.03% (19 to 36.67)	24.54% (16.31 to 34.24)	19.39% (12.44 to 26.88)	0% (0 to 0)	0.76% (0.36 to 1.2)	2.57% (-0.31 to 5.76)
Monaco	15.42% (11.03 to 20.12)	5.66% (3.03 to 8.83)	0% (0 to 0)	7.34% (6.65 to 8.11)	0% (-0.08 to 0.08)	3.24% (-0.44 to 7.32)
Mongolia	36.61% (27.09 to 47.05)	28.05% (18.26 to 40.55)	7.91% (0.6 to 27.51)	6.96% (4.98 to 9.27)	-0.04% (-0.15 to 0)	5.4% (-0.7 to 12.3)
Montenegro	26.12% (19.64 to 38.23)	16.44% (10.72 to 31.08)	3.14% (0.04 to 21.28)	8.54% (7.53 to 9.79)	0.07% (-0.22 to 0.48)	3.28% (-0.42 to 7.5)
Morocco	27.04% (19.73 to 34.32)	16.3% (11.98 to 21.42)	1.1% (0.18 to 3.47)	5.9% (4.85 to 7.07)	0.63% (-0.14 to 1.87)	6.8% (-0.92 to 15.05)
Mozambique	55.73% (46.06 to 63.94)	49.43% (41.69 to 56.94)	46.03% (38.56 to 53.29)	1.36% (0.99 to 1.79)	0.75% (-0.37 to 2.24)	10.69% (-1.52 to 23.19)
Myanmar	48.99% (38.1 to 58)	43% (32.41 to 52.11)	29.45% (15.02 to 42.27)	0.83% (0.53 to 1.22)	2.15% (1.28 to 3.26)	7.82% (-1.02 to 17.57)
Namibia	31.07% (18.61 to 46.2)	23.83% (12.59 to 39.4)	10.68% (1.06 to 30.87)	2.7% (2.26 to 3.28)	0.53% (-0.51 to 1.98)	6.5% (-0.89 to 14.82)
Nauru	5.85% (2.21 to 10.5)	3.34% (0.46 to 7.52)	0.73% (0.02 to 3.45)	0% (0 to 0)	0.85% (0.46 to 1.28)	1.77% (-0.23 to 4.03)
Nepal	56.28% (45.2 to 65.77)	46.42% (38.48 to 54.03)	33.67% (26.19 to 41.34)	4.09% (3.42 to 4.84)	0.8% (-0.5 to 2.59)	14.35% (-2.07 to 31.03)

Netherlands	16.78% (12.08 to 21.9)	7.33% (5.09 to 9.97)	0% (0 to 0)	6.76% (5.68 to 9.24)	0.01% (-0.04 to 0.09)	3.69% (-0.47 to 8.52)
New Zealand	14.43% (8.52 to 20.91)	3.46% (1.43 to 5.78)	0% (0 to 0.03)	6.41% (5.37 to 7.79)	0% (-0.01 to 0.01)	5.31% (-0.69 to 11.86)
Nicaragua	38.08% (26.87 to 48.26)	30.68% (21.65 to 38.82)	23.22% (12.66 to 32.78)	0.29% (0.15 to 0.43)	0.65% (0.59 to 0.72)	9.88% (-1.39 to 21.87)
Niger	60.42% (50.3 to 69.27)	50.34% (42.53 to 57.82)	41.48% (33.37 to 49.71)	1.18% (0.32 to 2.52)	6.81% (4.24 to 10.22)	13.76% (-1.99 to 29.53)
Nigeria	46.94% (37.41 to 55.62)	42.35% (34.12 to 50.51)	24.77% (14.06 to 35.84)	0.3% (0.07 to 0.59)	1.97% (1.47 to 2.68)	6.23% (-0.82 to 13.71)
Niue	5.99% (1.93 to 11.44)	3.85% (0.41 to 9.08)	0.74% (0.01 to 4.28)	0.36% (0.19 to 0.52)	0.17% (0.14 to 0.21)	1.71% (-0.22 to 3.95)
North Macedonia	31.77% (25.59 to 39.32)	21.46% (16.01 to 29.39)	2.79% (0.1 to 14.98)	8.95% (8.06 to 11.54)	0.24% (-0.28 to 1.23)	4.36% (-0.56 to 9.84)
Northern Mariana Islands	8.03% (4.56 to 12.24)	6.07% (2.89 to 10.5)	0.19% (0 to 1.85)	0.17% (0.08 to 0.27)	0.59% (0.51 to 0.71)	1.34% (-0.17 to 3.1)
Norway	12.8% (8.46 to 17.43)	2.38% (0.9 to 4.3)	0% (0 to 0)	7.37% (6.36 to 8.39)	-0.01% (-0.02 to 0)	3.56% (-0.45 to 8.08)
Oman	40.23% (30.16 to 50.32)	29.72% (21.65 to 38.49)	0.01% (0 to 0.05)	2.56% (0.61 to 5.26)	7.51% (3.44 to 12.38)	5.86% (-0.75 to 13.02)
Pakistan	53.23% (42.72 to 62.76)	42.12% (33.49 to 49.99)	24.48% (13.79 to 35.09)	4.52% (2.01 to 7.64)	5.2% (0.74 to 10.8)	10.96% (-1.53 to 24.09)
Palau	5.64% (1.71 to 9.77)	3.76% (0.35 to 7.74)	0.01% (0 to 0.04)	0% (-0.02 to 0.02)	0.52% (0.23 to 0.82)	1.44% (-0.18 to 3.28)
Palestine	32.52% (22.7 to 42.35)	21.89% (14.95 to 29.83)	1.29% (0.5 to 2.73)	3.53% (2.73 to 4.46)	1.7% (-0.58 to 5.64)	8.97% (-1.2 to 19.44)
Panama	15.85% (6.77 to 25.14)	8.26% (4.48 to 13.6)	0.42% (0 to 4.26)	0.29% (0.21 to 0.4)	0.2% (0.09 to 0.41)	7.82% (-1.05 to 16.95)
Papua New Guinea	49.38% (40.63 to 56.87)	46.14% (37.28 to 54.03)	41.17% (30.99 to 49.79)	2.85% (2.46 to 3.54)	0.12% (0.08 to 0.18)	3.2% (-0.39 to 7.42)
Paraguay	22.12% (9.85 to 34.78)	13.88% (4.05 to 27.26)	7% (0.57 to 21.97)	2.44% (1.74 to 3.17)	0.94% (-0.67 to 3.23)	6.46% (-0.83 to 14.45)
Peru	32.51% (22.79 to 42.46)	23.39% (15.86 to 31.91)	3.09% (0.18 to 13.7)	5.62% (4.63 to 6.69)	0.04% (0 to 0.1)	6.66% (-0.85 to 15.16)
Philippines	34.4% (25.29 to 43.47)	30.58% (22 to 39.66)	18.03% (9.22 to 29.14)	0.11% (0.02 to 0.21)	0.88% (0.78 to 1.02)	4.62% (-0.58 to 10.25)
Poland	25.49% (20.07 to 31.45)	14.38% (10.86 to 19.35)	0.48% (0 to 4.73)	8.81% (7.87 to 10.96)	-0.02% (-0.2 to 0.13)	4.6% (-0.6 to 10.48)
Portugal	18.59% (10.44 to 27.16)	4.05% (2.14 to 6.21)	0.01% (0 to 0.14)	8.18% (7.7 to 8.8)	0.01% (-0.02 to 0.05)	7.6% (-0.99 to 16.86)
Puerto Rico	6.89% (1.81 to 12.22)	2.66% (0.7 to 4.87)	0% (0 to 0.01)	0.01% (-0.06 to 0.09)	0.41% (0.29 to 0.58)	3.94% (-0.5 to 8.89)
Qatar	47.85% (38.56 to 56.39)	41.28% (32.69 to 49.26)	0% (0 to 0)	2.21% (0.55 to 4.48)	6.07% (2.03 to 10.87)	3.61% (-0.45 to 8.23)
Republic of Korea	28.25% (20.28 to 36.75)	19.26% (13.13 to 26.42)	0% (0 to 0.03)	5.87% (5.03 to 6.85)	0.28% (-0.41 to 1.49)	5.36% (-0.69 to 11.97)
Republic of Moldova	24.58% (17.81 to 31.63)	14.83% (9.71 to 21.62)	5.77% (2 to 10.99)	7.73% (6.62 to 9.47)	0.1% (-0.66 to 1.04)	3.94% (-0.5 to 8.96)
Romania	22.51% (17.15 to 28.76)	11.26% (7.96 to 16.55)	0.59% (0 to 5.99)	8.61% (7.67 to 10.23)	0.21% (-0.36 to 1.19)	4.26% (-0.57 to 9.31)
Russian Federation	18.2% (13.61 to 22.92)	7.76% (4.69 to 12.06)	0.11% (0 to 0.96)	8.62% (6.92 to 10.27)	-0.09% (-0.51 to 0.29)	3.04% (-0.39 to 6.88)

Rwanda	55.86% (47.12 to 63.57)	50.47% (42.63 to 57.97)	46.69% (39.09 to 54.44)	3.65% (3.09 to 4.78)	0% (0 to 0.02)	7.6% (-1.01 to 17.18)
Saint Kitts and Nevis	9.6% (2.94 to 16.48)	3.98% (1.41 to 6.99)	0.05% (0 to 0.56)	0.02% (-0.1 to 0.13)	0.36% (0.22 to 0.52)	5.5% (-0.71 to 12.44)
Saint Lucia	24.54% (11.87 to 37.83)	17.3% (7.32 to 30.73)	0.66% (0.04 to 3.64)	0.01% (-0.04 to 0.06)	0.43% (0.26 to 0.72)	8.36% (-1.16 to 18.42)
Saint Vincent and the Grenadines	25.61% (10.79 to 40.41)	17.45% (6.69 to 32.64)	0.71% (0.05 to 3.2)	0% (-0.03 to 0.04)	0.43% (0.26 to 0.63)	9.53% (-1.29 to 20.87)
Samoa	35.26% (21.92 to 46.13)	33.66% (20.69 to 44.68)	27.26% (13 to 38.91)	0.1% (-0.06 to 0.26)	0.04% (0.01 to 0.07)	2.28% (-0.29 to 5.4)
San Marino	15.75% (11.17 to 20.26)	6.47% (3.46 to 9.99)	0% (0 to 0)	7.03% (6 to 7.97)	0.06% (-0.24 to 0.48)	3.05% (-0.4 to 6.99)
Sao Tome and Principe	38.26% (29.48 to 47.18)	33.32% (26.18 to 40.61)	24.86% (17.62 to 32.34)	0.03% (-0.03 to 0.09)	0.17% (-0.01 to 0.4)	7.26% (-1 to 16.38)
Saudi Arabia	46.21% (36.39 to 55.36)	35.22% (27.65 to 42.93)	0% (0 to 0.02)	3.07% (1.03 to 5.62)	8.27% (2.37 to 15.01)	6.81% (-0.89 to 15.53)
Senegal	53.2% (44.4 to 60.95)	47.43% (39.39 to 54.98)	38.78% (30.45 to 46.73)	0.27% (0.05 to 0.56)	3.94% (2.79 to 5.56)	7.17% (-0.99 to 15.62)
Serbia	29.32% (23.16 to 37.67)	19.16% (13.86 to 28.51)	2.66% (0.06 to 15.97)	8.71% (7.64 to 10.57)	0.29% (-0.43 to 1.53)	3.96% (-0.5 to 8.99)
Seychelles	8.81% (4.03 to 14.84)	5.44% (1.8 to 10.01)	0.02% (0 to 0.17)	0.09% (-0.12 to 0.27)	0.35% (0.24 to 0.5)	3.14% (-0.41 to 7.26)
Sierra Leone	54.56% (45.17 to 62.91)	49.69% (41.97 to 57.15)	43% (35.45 to 50.19)	0.11% (-0.01 to 0.24)	0.64% (0.47 to 0.94)	9.12% (-1.26 to 19.93)
Singapore	18% (9.16 to 27.16)	12.32% (6.6 to 19.16)	0% (0 to 0)	-0.03% (-0.08 to 0.02)	0.32% (0.26 to 0.4)	6.22% (-0.8 to 14.1)
Slovakia	21.35% (16.9 to 25.91)	11.41% (8.56 to 14.6)	0.02% (0 to 0.26)	8.05% (6.9 to 9.2)	0.02% (-0.33 to 0.42)	3.44% (-0.45 to 7.88)
Slovenia	19.45% (15.4 to 24.17)	9.85% (7.13 to 13.45)	0.16% (0 to 1.57)	7.78% (7 to 9.62)	0.06% (-0.23 to 0.55)	3.05% (-0.38 to 6.94)
Solomon Islands	50.39% (41.78 to 57.89)	47.75% (39.95 to 55.36)	44.86% (36.99 to 52.31)	0.03% (0 to 0.07)	0.24% (0.08 to 0.49)	4.83% (-0.57 to 10.96)
Somalia	56.6% (45.84 to 65.89)	49.51% (41.8 to 56.95)	46.43% (38.89 to 53.35)	0.33% (0.21 to 0.48)	0.77% (-0.02 to 1.24)	13.35% (-1.91 to 28.82)
South Africa	27.74% (20.95 to 35.52)	19.3% (13.64 to 25.93)	4.13% (0.75 to 10.8)	5.55% (4.9 to 6.16)	0.08% (-0.12 to 0.39)	5.15% (-0.65 to 11.49)
South Sudan	56.19% (47.52 to 64.2)	49.8% (41.91 to 57.28)	43.23% (35.02 to 50.69)	0.33% (0.09 to 0.68)	4.5% (3.18 to 6.22)	8.44% (-1.14 to 19.07)
Spain	16.8% (10.08 to 23.93)	5.4% (3.47 to 7.77)	0.01% (0 to 0.11)	6.21% (5.47 to 7.01)	0.08% (-0.12 to 0.39)	6.16% (-0.8 to 13.96)
Sri Lanka	27.55% (15.09 to 44.78)	24.26% (12.63 to 42.36)	10.74% (0.87 to 34.41)	0.37% (0.22 to 0.56)	0.55% (0.42 to 0.69)	3.45% (-0.44 to 7.82)
Sudan	51.87% (40.87 to 61.97)	39.88% (31.49 to 49.02)	19.92% (11.17 to 29.19)	1.26% (0.28 to 2.61)	8.62% (4.58 to 13.85)	11.48% (-1.63 to 25.48)
Suriname	28.35% (14.78 to 42.97)	22.01% (9.86 to 35.83)	1.04% (0.03 to 6.53)	0.02% (-0.06 to 0.09)	0.37% (0.22 to 0.72)	7.78% (-1.03 to 17.42)
Sweden	11.98% (8.68 to 15.51)	2.14% (0.64 to 4.04)	0% (0 to 0)	7.85% (6.76 to 8.88)	-0.02% (-0.05 to 0)	2.42% (-0.31 to 5.54)
Switzerland	16.51% (11.48 to 21.79)	5.48% (3.56 to 7.8)	0% (0 to 0)	7.64% (6.73 to 9.3)	0% (-0.07 to 0.07)	4.37% (-0.54 to 10)
Syrian Arab Republic	32.04% (22.28 to 42.42)	19.5% (13.89 to 25.62)	0.09% (0.03 to 0.21)	5.47% (4.56 to 6.28)	1.45% (-0.2 to 3.99)	9.44% (-1.31 to 21.27)

Taiwan (Province of China)	20.11% (13.1 to 27.65)	12.62% (9.51 to 16.3)	0.1% (0 to 0.57)	1.59% (1.19 to 2)	0.93% (-0.65 to 3.26)	6.26% (-0.81 to 14.07)
Tajikistan	43.96% (35.27 to 52.13)	35.08% (27.17 to 43.13)	21.43% (14.27 to 28.48)	7.39% (6.2 to 8.34)	0.61% (-0.33 to 1.91)	6.28% (-0.83 to 14.32)
Thailand	27.93% (21.24 to 35.89)	23.36% (17.3 to 30.61)	1.53% (0.02 to 9.79)	0.59% (0.17 to 1.18)	1.76% (1.48 to 2.18)	3.72% (-0.46 to 8.69)
Timor-Leste	44.26% (27.86 to 56.8)	38.26% (22.53 to 50.65)	29.24% (11.73 to 45.07)	0.56% (0.39 to 0.81)	0.07% (0.02 to 0.15)	9.15% (-1.24 to 20.17)
Togo	53.92% (44.97 to 62.02)	48.86% (41.12 to 56.54)	39.98% (32.66 to 46.79)	0.07% (0 to 0.17)	2.19% (1.8 to 2.76)	7.91% (-1.03 to 18.02)
Tokelau	5.08% (1.32 to 9.22)	2.55% (0.03 to 6.63)	0.01% (0 to 0.06)	0% (-0.02 to 0.02)	0.56% (0.18 to 0.96)	2.04% (-0.27 to 4.69)
Tonga	27.63% (16.56 to 37.9)	25.55% (15.33 to 36.14)	18.51% (8.37 to 28.59)	0% (-0.03 to 0.04)	0.32% (0.18 to 0.52)	2.48% (-0.32 to 5.85)
Trinidad and Tobago	21.02% (8.13 to 37.36)	17.88% (6.15 to 34.48)	0% (0 to 0.03)	0.01% (-0.07 to 0.09)	0.25% (0.08 to 0.5)	3.57% (-0.47 to 8.11)
Tunisia	29.44% (20.03 to 38.89)	17.67% (12.25 to 24.01)	0.06% (0 to 0.29)	4.84% (3.79 to 5.81)	2.11% (-0.12 to 5.91)	8.04% (-1.1 to 17.9)
Turkey	27.84% (21.86 to 33.84)	17.69% (13.59 to 21.9)	0.09% (0 to 0.84)	7.15% (6.33 to 7.98)	0.25% (-0.31 to 1.01)	5.38% (-0.69 to 11.98)
Turkmenistan	25.07% (18.42 to 32.78)	14.8% (8.4 to 22.53)	0.01% (0 to 0.1)	7.06% (5.74 to 8.52)	1.8% (0.13 to 4.19)	3.67% (-0.48 to 8.23)
Tuvalu	12.45% (7.39 to 17.97)	9.21% (5.52 to 14.03)	7.46% (4.22 to 11.75)	0.01% (-0.01 to 0.02)	0.59% (0.23 to 0.96)	3% (-0.37 to 6.82)
Uganda	54.03% (44.93 to 62.32)	49.43% (41.69 to 56.88)	43.44% (35.95 to 50.75)	1.1% (0.84 to 1.37)	0.05% (-0.04 to 0.17)	8.18% (-1.14 to 18.35)
Ukraine	21.52% (15.71 to 27.67)	12.05% (7.02 to 18.45)	1.02% (0.12 to 3.99)	8.57% (7.06 to 9.92)	-0.09% (-0.76 to 0.47)	2.5% (-0.32 to 5.84)
United Arab Emirates	38.28% (29.07 to 47.2)	28.66% (21.34 to 36.61)	0% (0 to 0)	2.78% (0.65 to 5.82)	7.88% (3.43 to 13.26)	3.7% (-0.46 to 8.4)
United Kingdom	14.84% (10.45 to 19.66)	5.55% (3.63 to 7.83)	0% (0 to 0.01)	6.69% (5.45 to 9.16)	0% (-0.02 to 0.04)	3.38% (-0.43 to 7.67)
United Republic of Tanzania	52.53% (44.1 to 60.11)	48.91% (40.95 to 56.41)	43.79% (35.7 to 51.25)	1.43% (1.15 to 1.73)	0.13% (0 to 0.32)	5.69% (-0.75 to 12.74)
United States of America	11.22% (6.95 to 15.89)	3.25% (1.62 to 5.23)	0% (0 to 0.01)	4.8% (4.05 to 5.51)	0.34% (-0.43 to 1.39)	3.29% (-0.42 to 7.48)
United States Virgin Islands	7.79% (2.78 to 12.94)	3.96% (1.82 to 6.5)	0.01% (0 to 0.02)	0.02% (-0.09 to 0.13)	0.33% (0.21 to 0.49)	3.66% (-0.47 to 8.45)
Uruguay	17.18% (10.54 to 25.12)	6.45% (2.46 to 11.44)	0.14% (0 to 1.27)	6.1% (5.59 to 6.86)	0.06% (-0.09 to 0.32)	5.67% (-0.74 to 12.64)
Uzbekistan	34.93% (27.1 to 44.05)	26.66% (19.43 to 35.49)	4.32% (0.77 to 11.78)	7.04% (5.57 to 8.21)	0.67% (-0.47 to 1.96)	3.95% (-0.52 to 9.21)
Vanuatu	48.39% (40.16 to 55.93)	45.94% (38.22 to 53.97)	42.33% (33.99 to 50.29)	0.21% (0.09 to 0.33)	0.11% (0.09 to 0.14)	4.28% (-0.55 to 9.71)
Venezuela (Bolivarian Republic of)	19.62% (9.86 to 29.47)	10.61% (6.06 to 16.17)	0.16% (0.01 to 0.93)	0.85% (0.62 to 1.1)	0.45% (0.37 to 0.54)	8.9% (-1.18 to 19.89)

Viet Nam	34.45% (24.73 to 44.07)	28.29% (19.45 to 37.17)	16.35% (5.89 to 28.17)	1.24% (0.78 to 1.79)	1.7% (0.16 to 3.68)	5.85% (-0.79 to 13.26)
Yemen	45.81% (33.01 to 57.4)	34.24% (26.1 to 44.28)	17.41% (11.59 to 23.75)	2.8% (2.3 to 3.54)	1.5% (0.69 to 2.63)	14.03% (-1.97 to 30.15)
Zambia	50.32% (41.22 to 58.61)	45.3% (36.85 to 53.54)	37.84% (27.66 to 46.53)	2.28% (1.87 to 2.77)	0.52% (-0.37 to 1.89)	6.63% (-0.88 to 14.87)
Zimbabwe	49.16% (40.41 to 56.98)	42.63% (35 to 50.54)	38.26% (31.16 to 45.71)	3.1% (2.36 to 3.97)	0.44% (-0.37 to 1.66)	8.21% (-1.11 to 18.31)

Note: Percentage data are presented to one decimal place. 95% uncertainty intervals are presented to one decimal places, and if necessary, to two decimal places. DALY=disability-adjusted life-year. Environmental risks cluster includes ambient particulate matter pollution, household air pollution from solid fuels, low temperature, high temperature and lead exposure.

eTable 11b. Percent of DALYs (with 95% uncertainty intervals) due to IS for dietary risks by country and territory for both sexes combined in 2021, aged ≥20 years

	**Dietary risks**	Diet high in sodium	Diet high in red meat	Diet low in fruits	Diet high in processed meat	Diet low in vegetables	Diet low in whole grains	Alcohol use	Diet high in sugar-sweetened beverages	Diet low in fiber	Diet low in polyunsaturated fatty acids
Afghanistan	26.73% (2.9 to 43.79)	1.54% (0 to 7.7)	-1.64% (-4.56 to 0.79)	5.05% (2.91 to 7.22)	0.21% (0.05 to 0.36)	13.95% (8.93 to 18.66)	11.97% (-14.96 to 29.67)	0.08% (-0.01 to 0.24)	0.06% (0.03 to 0.09)	4.79% (-0.26 to 9.13)	0.04% (0.01 to 0.07)
Albania	24.76% (7.44 to 40.94)	16.3% (4.78 to 30.37)	-0.25% (-1.22 to 0.89)	0.6% (0.08 to 1.15)	3.97% (0.89 to 7.02)	0.09% (-0.04 to 0.28)	5.48% (-5.74 to 16.71)	5.79% (-0.6 to 14.33)	0.7% (0.32 to 1.15)	0.49% (-0.04 to 1.25)	0.02% (0.01 to 0.04)
Algeria	11.27% (-5.32 to 25.14)	1.45% (0 to 7.61)	-0.93% (-2.57 to 0.48)	1.55% (0.8 to 2.42)	0.26% (0.07 to 0.45)	1.23% (0.58 to 1.98)	7.88% (-9.35 to 21.02)	0.48% (-0.05 to 1.38)	0.24% (0.11 to 0.42)	0.46% (-0.02 to 0.99)	0.02% (0.01 to 0.04)
American Samoa	16.4% (2.55 to 29.43)	4.54% (0.07 to 14.77)	-1.15% (-3.58 to 0.78)	2.24% (1.25 to 3.33)	0.37% (0.1 to 0.66)	5.18% (3.08 to 7.63)	5.94% (-6.45 to 15.83)	0.54% (-0.05 to 1.87)	0.47% (0.25 to 0.79)	0.04% (0 to 0.12)	0.03% (0.01 to 0.05)
Andorra	10.96% (-0.72 to 23.07)	2.76% (0.01 to 11.07)	1.29% (-0.67 to 3.83)	0.9% (0.12 to 1.69)	2.11% (0.5 to 3.82)	0.65% (-0.01 to 1.28)	3.59% (-3.66 to 11.53)	10.58% (-1.28 to 25.5)	0.63% (0.29 to 1.03)	0.67% (-0.05 to 1.74)	0.02% (0 to 0.03)
Angola	13.29% (1.63 to 24.59)	3.02% (0 to 12.11)	-1.14% (-3.1 to 0.61)	2.6% (1.51 to 3.75)	0.19% (0.05 to 0.34)	4.14% (2.54 to 5.88)	5.22% (-5.22 to 13.85)	5.4% (-0.92 to 13.63)	0.05% (0.02 to 0.09)	0.49% (-0.02 to 1)	0.03% (0.01 to 0.05)
Antigua and Barbuda	13.25% (-0.67 to 26.72)	4.4% (0.02 to 15.14)	-0.78% (-2.16 to 0.44)	1.22% (0.54 to 2.01)	0.15% (0.04 to 0.27)	2.76% (1.1 to 4.41)	6.15% (-6.45 to 17.56)	4.91% (-0.66 to 12.58)	0.06% (0.03 to 0.1)	3.39% (-0.17 to 7.33)	0.01% (0 to 0.02)
Argentina	17.38% (-0.53 to 33.16)	6.69% (0.12 to 18.74)	3.59% (-2.31 to 8.81)	0.95% (0.38 to 1.61)	0.94% (0.24 to 1.66)	1.44% (0.44 to 2.48)	4.99% (-5.11 to 14.82)	8.54% (-1.26 to 20.8)	0.88% (0.45 to 1.4)	1.91% (-0.13 to 4.23)	0.02% (0.01 to 0.04)
Armenia	14.3% (-2.49 to 30.13)	6.79% (0.33 to 18.88)	-1.01% (-3.01 to 0.56)	1.12% (0.51 to 1.83)	0.4% (0.1 to 0.7)	0.01% (-0.02 to 0.06)	7.4% (-8.18 to 20.74)	4.14% (-0.49 to 11.55)	0.07% (0.03 to 0.11)	1% (-0.05 to 2.21)	0.03% (0.01 to 0.05)
Australia	11.11% (-0.31 to 23.03)	1.97% (0 to 9.17)	2.57% (-1.6 to 6.89)	1.21% (0.11 to 2.33)	2.32% (0.6 to 4.07)	0.94% (-0.05 to 1.93)	2.46% (-2.42 to 8.18)	10.92% (-1.3 to 27.21)	0.83% (0.41 to 1.3)	1.17% (-0.07 to 2.74)	0.02% (0.01 to 0.04)
Austria	13.35% (1.75 to 26.29)	7.06% (0.28 to 19.66)	1.12% (-0.67 to 3.41)	0.78% (0.07 to 1.51)	1.63% (0.36 to 2.85)	0.83% (-0.02 to 1.67)	2.32% (-2.22 to 7.63)	10.9% (-1.5 to 25.54)	0.51% (0.25 to 0.81)	0.54% (-0.06 to 1.37)	0.02% (0.01 to 0.04)
Azerbaijan	16.14% (-3.64 to 32.4)	6.14% (0.27 to 17.78)	-1.46% (-3.98 to 0.78)	1.72% (0.97 to 2.61)	0.76% (0.17 to 1.38)	0.14% (0.01 to 0.32)	9.25% (-10.98 to 23.98)	4.48% (-0.64 to 11.95)	0.2% (0.09 to 0.32)	0.75% (-0.04 to 1.54)	0.03% (0.01 to 0.05)
Bahamas	12.96% (-1.95 to 26.2)	4.13% (0.02 to 14.17)	-1.1% (-3.37 to 0.69)	1.73% (0.9 to 2.66)	0.22% (0.05 to 0.38)	1.57% (0.74 to 2.46)	6.9% (-7.73 to 18.76)	4.17% (-0.61 to 10.95)	0.11% (0.05 to 0.2)	3.22% (-0.17 to 6.45)	0.02% (0 to 0.03)
Bahrain	12.99% (-7.58 to 28.27)	1.79% (0 to 8.67)	-1.64% (-4.58 to 0.77)	1.45% (0.87 to 2.09)	0.42% (0.11 to 0.73)	0.57% (0.24 to 1.03)	10.05% (-12.16 to 25.36)	0.72% (-0.09 to 2.03)	0.6% (0.28 to 0.97)	0.31% (-0.01 to 0.69)	0.02% (0.01 to 0.04)

Bangladesh	18.11% (6.94 to 30.37)	6.19% (0.07 to 18.35)	-0.15% (-0.41 to 0.08)	5.65% (2.62 to 9.24)	0.68% (0.16 to 1.22)	6% (2.71 to 9.5)	1.5% (-1.37 to 4.29)	0.27% (-0.03 to 0.94)	0.03% (0.01 to 0.05)	4.95% (-0.27 to 10.38)	0.03% (0.01 to 0.05)
Barbados	9.8% (0.67 to 20.63)	2.82% (0 to 11.19)	-0.79% (-2.36 to 0.36)	1.9% (0.64 to 3.29)	0.32% (0.08 to 0.57)	1.99% (0.47 to 3.42)	3.76% (-3.65 to 11.32)	5.04% (-0.69 to 12.77)	0.27% (0.13 to 0.45)	1.56% (-0.1 to 3.72)	0.02% (0 to 0.03)
Belarus	13.76% (-3.77 to 28.65)	3.05% (0 to 11.95)	1.97% (-0.99 to 5.62)	1.87% (0.93 to 2.94)	1.02% (0.24 to 1.86)	0.39% (0.06 to 0.82)	6.75% (-7.09 to 19.15)	8.85% (-0.82 to 23.58)	0.08% (0.04 to 0.13)	0.15% (-0.01 to 0.37)	0.02% (0.01 to 0.04)
Belgium	13.53% (1.27 to 27.37)	6.04% (0.1 to 17.47)	1.1% (-0.72 to 3.19)	1.01% (0.01 to 2.02)	2.24% (0.53 to 3.98)	0.49% (-0.1 to 1.07)	3.11% (-3.02 to 10.28)	11.15% (-1.52 to 26.23)	0.63% (0.3 to 1.03)	0.87% (-0.2 to 2.33)	0.01% (0 to 0.03)
Belize	12.73% (1.08 to 24.14)	4.32% (0.02 to 14.36)	-0.77% (-2.15 to 0.39)	0.61% (0.33 to 0.95)	0.17% (0.04 to 0.28)	4.28% (2.34 to 6.38)	4.67% (-4.8 to 12.99)	4.3% (-0.63 to 11.17)	0.13% (0.06 to 0.22)	0.87% (-0.04 to 1.84)	0.02% (0.01 to 0.03)
Benin	14.53% (3.21 to 26.71)	4.53% (0.01 to 15.64)	-0.44% (-1.2 to 0.23)	4.15% (2.32 to 6.23)	0.32% (0.07 to 0.56)	3.25% (1.81 to 4.74)	3.83% (-4.02 to 10.03)	3.67% (-0.63 to 9.35)	0.03% (0.02 to 0.06)	0.03% (0 to 0.06)	0.03% (0.01 to 0.06)
Bermuda	10.95% (-0.31 to 23.75)	4.6% (0.02 to 15.36)	0.17% (-0.72 to 1.71)	1.29% (0.39 to 2.29)	0.32% (0.07 to 0.58)	1.03% (0.07 to 1.91)	4.02% (-4.04 to 12.44)	7.14% (-1.01 to 18.45)	0.17% (0.08 to 0.29)	1.76% (-0.11 to 4.18)	0.02% (0 to 0.03)
Bhutan	14.26% (3.59 to 26.5)	6.15% (0.07 to 18.31)	-0.36% (-1.05 to 0.18)	2.87% (1.39 to 4.63)	0.49% (0.12 to 0.92)	3.24% (1.58 to 5.07)	2.81% (-2.57 to 8.02)	0.8% (-0.09 to 2.33)	0.03% (0.01 to 0.05)	2.27% (-0.09 to 4.9)	0.02% (0.01 to 0.05)
Bolivia (Plurinational State of)	15.57% (3.67 to 28.14)	6.48% (0.15 to 18.85)	-0.94% (-3.01 to 0.68)	1.76% (0.93 to 2.74)	0.11% (0.03 to 0.19)	4.77% (2.51 to 7.22)	4.31% (-4.46 to 11.98)	5.53% (-0.91 to 13.8)	0.21% (0.09 to 0.38)	1.78% (-0.08 to 3.78)	0.02% (0.01 to 0.03)
Bosnia and Herzegovina	20.5% (6.4 to 34.75)	16.39% (4.24 to 30.76)	-0.67% (-2.02 to 0.36)	1.27% (0.45 to 2.15)	0.79% (0.2 to 1.43)	0.43% (0.04 to 0.89)	2.86% (-2.55 to 8.54)	6.7% (-0.81 to 16.19)	0.31% (0.14 to 0.55)	0.03% (-0.01 to 0.1)	0.02% (0.01 to 0.04)
Botswana	16.95% (6.46 to 27.52)	2.48% (0 to 10.62)	-1.36% (-3.88 to 0.7)	7.75% (4.31 to 11.23)	0.14% (0.03 to 0.24)	5.63% (3.23 to 8.17)	3.94% (-4.07 to 10.56)	3.48% (-0.52 to 9.03)	0.05% (0.03 to 0.09)	1.15% (-0.06 to 2.39)	0.03% (0.01 to 0.05)
Brazil	16.12% (2.19 to 29.7)	7.11% (0.3 to 20.07)	2.5% (-1.49 to 6.13)	1.18% (0.57 to 1.89)	0.46% (0.11 to 0.81)	2.86% (1.33 to 4.44)	3.17% (-2.94 to 8.94)	5.56% (-0.86 to 13.59)	0.42% (0.19 to 0.67)	1.32% (-0.05 to 2.89)	0.01% (0 to 0.02)
Brunei Darussalam	21.48% (7.45 to 35.48)	12.15% (1.93 to 25.85)	-1.32% (-3.65 to 0.63)	3.56% (1.98 to 5.12)	0.68% (0.16 to 1.24)	3.51% (2.1 to 5.07)	4.56% (-4.58 to 11.96)	0.42% (-0.04 to 1.27)	0.17% (0.08 to 0.29)	3.55% (-0.19 to 6.98)	0.03% (0.01 to 0.06)
Bulgaria	23.62% (6.77 to 39.33)	16.69% (4.71 to 31.33)	0.99% (-0.56 to 3.27)	1.62% (0.58 to 2.73)	1.23% (0.31 to 2.29)	0.22% (-0.04 to 0.51)	4.4% (-4.34 to 13.14)	10.78% (-1.3 to 25.67)	0.48% (0.21 to 0.79)	1.5% (-0.1 to 3.49)	0.02% (0 to 0.03)
Burkina Faso	26.54% (11.38 to 40.68)	3.78% (0 to 13.77)	-1.15% (-3.19 to 0.59)	13.07% (7.68 to 18.62)	0.29% (0.07 to 0.51)	9.35% (5.75 to 13.14)	5.61% (-6.01 to 15.09)	7.83% (-1.28 to 18.92)	0.03% (0.01 to 0.05)	0.1% (0 to 0.22)	0.04% (0.01 to 0.07)
Burundi	22.74% (5.02 to 37.85)	7.41% (0.28 to 19.81)	-0.18% (-0.48 to 0.09)	1.58% (0.91 to 2.33)	0.15% (0.04 to 0.28)	9.18% (5.63 to 12.63)	7.17% (-7.83 to 18.89)	5.09% (-0.84 to 13.06)	0.03% (0.01 to 0.06)	0.2% (-0.01 to 0.43)	0.04% (0.01 to 0.07)
Cabo Verde	10.68% (1.53 to 21.99)	3.85% (0 to 13.95)	-0.86% (-2.37 to 0.45)	2.89% (1.5 to 4.43)	0.49% (0.12 to 0.85)	1.4% (0.49 to 2.37)	3.38% (-3.44 to 9.14)	7.72% (-1.55 to 18.22)	0.05% (0.02 to 0.08)	0.73% (-0.03 to 1.53)	0.02% (0.01 to 0.04)
Cambodia	22.96% (10.63 to 35.8)	11.52% (1.72 to 24.92)	-0.81% (-2.29 to 0.45)	5.56% (3 to 8.54)	0.05% (0.01 to 0.09)	7.4% (4.14 to 10.79)	1.5% (-1.41 to 4.06)	5.35% (-0.95 to 13.15)	0.03% (0.01 to 0.06)	5.6% (-0.34 to 11.17)	0.03% (0.01 to 0.06)
Cameroon	11.54% (1.1 to 22.83)	3.83% (0 to 13.58)	-0.97% (-2.62 to 0.51)	1.99% (1.13 to 2.85)	0.61% (0.15 to 1.06)	2.29% (1.34 to 3.34)	4.24% (-4.24 to 10.97)	9.38% (-1.58 to 22.12)	0.07% (0.03 to 0.11)	0.06% (0 to 0.14)	0.04% (0.01 to 0.08)

Canada	10.83% (0.73 to 22.19)	4.87% (0.07 to 15.17)	0.49% (-0.33 to 2.23)	1.22% (0.35 to 2.16)	0.88% (0.21 to 1.54)	0.93% (0.19 to 1.66)	2.9% (-2.84 to 9.01)	8.09% (-1.13 to 20.54)	0.3% (0.14 to 0.48)	0.65% (-0.05 to 1.54)	0.02% (0.01 to 0.04)
Central African Republic	23.45% (9.09 to 35.82)	2.9% (0 to 12.05)	-1.34% (-4.05 to 0.93)	4.32% (2.47 to 6.18)	0.37% (0.1 to 0.65)	13.99% (9.12 to 18.53)	5.97% (-6.39 to 15.24)	3.21% (-0.44 to 9.13)	0.08% (0.03 to 0.13)	1.14% (-0.05 to 2.28)	0.04% (0.01 to 0.07)
Chad	30.33% (9.48 to 47.47)	3.8% (0 to 13.98)	-1.21% (-3.33 to 0.61)	11.23% (6.55 to 15.98)	0.4% (0.1 to 0.73)	12.76% (8.08 to 17.26)	9.68% (-11.43 to 24.6)	4.95% (-0.8 to 14.05)	0.03% (0.01 to 0.05)	0.42% (-0.02 to 0.88)	0.04% (0.01 to 0.07)
Chile	15.52% (0.99 to 29.94)	7.17% (0.23 to 19.54)	0.76% (-0.36 to 2.94)	1.42% (0.48 to 2.4)	1.67% (0.4 to 2.96)	0.82% (0.13 to 1.51)	4.51% (-4.6 to 13.5)	9.8% (-1.55 to 23.75)	0.52% (0.25 to 0.85)	1.27% (-0.08 to 2.94)	0.02% (0.01 to 0.04)
China	22.91% (7.89 to 36.94)	17.22% (5.98 to 31.55)	0.87% (-0.42 to 3.13)	1.61% (0.81 to 2.51)	0.3% (0.08 to 0.52)	0.1% (0.01 to 0.23)	4.52% (-4.57 to 12.57)	6.59% (-1.11 to 15.73)	0.14% (0.07 to 0.22)	0.73% (-0.02 to 1.57)	0.03% (0.01 to 0.05)
Colombi a	18.14% (5.52 to 31.3)	12.34% (2.53 to 25.71)	-1.1% (-3.17 to 0.6)	1.32% (0.6 to 2.2)	0.23% (0.05 to 0.41)	2.69% (1.14 to 4.29)	3.39% (-3.34 to 9.78)	2.88% (-0.43 to 7.6)	0.27% (0.13 to 0.46)	1.57% (-0.08 to 3.38)	0.02% (0.01 to 0.04)
Comoro s	22.17% (8.09 to 35.58)	7.57% (0.26 to 20.34)	-0.42% (-1.17 to 0.23)	1.81% (0.98 to 2.71)	0.13% (0.03 to 0.23)	10.8% (6.28 to 15.39)	4.57% (-4.63 to 12.41)	0.56% (-0.07 to 1.62)	0.05% (0.02 to 0.08)	1.12% (-0.06 to 2.32)	0.03% (0.01 to 0.06)
Congo	19.38% (7.6 to 30.74)	3.01% (0 to 11.86)	-1.11% (-3.06 to 0.59)	2.96% (1.67 to 4.35)	0.21% (0.06 to 0.36)	11.43% (7.11 to 15.6)	4.6% (-4.71 to 12.27)	7.19% (-1.13 to 18.55)	0.06% (0.03 to 0.09)	2.93% (-0.15 to 5.71)	0.03% (0.01 to 0.06)
Cook Islands	18.46% (4.71 to 32.44)	9.9% (0.97 to 22.83)	-0.8% (-2.66 to 0.71)	1.53% (0.74 to 2.42)	0.4% (0.1 to 0.69)	3.46% (1.57 to 5.47)	4.64% (-4.69 to 12.95)	7.11% (-1.08 to 18.4)	0.66% (0.31 to 1.07)	0% (0 to 0.02)	0.02% (0.01 to 0.04)
Costa Rica	15.03% (2.77 to 28.18)	7.93% (0.47 to 20.44)	-0.98% (-2.88 to 0.52)	1.1% (0.48 to 1.8)	0.26% (0.06 to 0.46)	3.24% (0.93 to 5.38)	4.08% (-4.18 to 12.1)	4.2% (-0.59 to 11.31)	0.34% (0.17 to 0.57)	2.18% (-0.11 to 4.7)	0.02% (0 to 0.03)
Coted'Iv oire	17.28% (2.01 to 30.16)	4.23% (0.01 to 14.83)	-1.3% (-3.5 to 0.65)	2.62% (1.5 to 3.67)	0.46% (0.12 to 0.81)	5.91% (3.7 to 8)	6.72% (-7.36 to 16.99)	9.5% (-1.82 to 23)	0.07% (0.03 to 0.11)	0.01% (0 to 0.03)	0.04% (0.01 to 0.07)
Croatia	20.54% (6.41 to 34.94)	16.3% (4.93 to 30.31)	-0.65% (-2.07 to 0.37)	0.95% (0.1 to 1.82)	0.33% (0.08 to 0.61)	0.92% (0.05 to 1.78)	3.07% (-2.91 to 9.75)	9.28% (-1.04 to 22.32)	0.56% (0.26 to 0.94)	1.4% (-0.11 to 3.41)	0.02% (0 to 0.03)
Cuba	9.16% (0.09 to 19.84)	4.41% (0.02 to 14.65)	-0.95% (-2.74 to 0.49)	1.05% (0.4 to 1.73)	0.29% (0.08 to 0.52)	0.87% (0.21 to 1.52)	3.33% (-3.28 to 10.03)	4.89% (-0.66 to 12.84)	0.4% (0.21 to 0.63)	0.08% (-0.04 to 0.27)	0.02% (0.01 to 0.04)
Cyprus	9.42% (-0.64 to 21.33)	3.82% (0.02 to 13.82)	-0.44% (-1.65 to 0.44)	0.87% (0.01 to 1.72)	0.64% (0.15 to 1.15)	1.06% (-0.21 to 2.16)	3.76% (-3.8 to 12.71)	8.2% (-1.08 to 19.57)	0.17% (0.08 to 0.28)	1.67% (-0.21 to 4.13)	0.01% (0 to 0.03)
Czechia	21.62% (7.86 to 35.36)	16.39% (4.5 to 31.04)	-0.04% (-0.99 to 1.2)	1.47% (0.43 to 2.51)	1.06% (0.24 to 1.88)	1.34% (0.33 to 2.38)	2.23% (-2.06 to 6.9)	12.16% (-1.38 to 28.03)	0.48% (0.23 to 0.79)	1.32% (-0.08 to 2.93)	0.02% (0.01 to 0.04)
Democr atic People's Republic of Korea	21.7% (8.16 to 36.07)	15.65% (4.44 to 30.69)	-0.71% (-1.97 to 0.36)	2.38% (1.33 to 3.55)	0.23% (0.05 to 0.41)	1.48% (0.84 to 2.24)	3.97% (-3.85 to 10.66)	4.78% (-0.81 to 11.71)	0.04% (0.02 to 0.06)	2.3% (-0.11 to 4.7)	0.02% (0.01 to 0.04)
Democr atic Republic of the Congo	20.15% (8.21 to 31.45)	1.56% (0 to 8.22)	-0.21% (-0.57 to 0.11)	4.14% (2.38 to 6.16)	0.42% (0.1 to 0.75)	12.29% (7.66 to 16.59)	4.21% (-4.33 to 11.14)	3.59% (-0.45 to 9.93)	0.09% (0.04 to 0.15)	1.86% (-0.09 to 3.84)	0.04% (0.01 to 0.07)
Denmar k	11.36% (0.08 to 24.02)	4.16% (0.02 to 14.35)	0.8% (-0.41 to 2.6)	0.81% (-0.03 to 1.66)	1.92% (0.49 to 3.42)	0.7% (-0.09 to 1.5)	3.39% (-3.33 to 10.98)	12.11% (-1.55 to 28.13)	0.42% (0.2 to 0.72)	0.7% (-0.13 to 1.88)	0.02% (0 to 0.03)

Djibouti	25.79% (7.52 to 41.27)	7.16% (0.25 to 19.9)	-1.53% (-4.21 to 0.77)	10.76% (6.17 to 15.15)	0.18% (0.04 to 0.3)	4.59% (2.82 to 6.48)	7.96% (-8.89 to 20.57)	0.22% (-0.21 to 0.84)	0.05% (0.02 to 0.08)	3.55% (-0.21 to 6.82)	0.03% (0.01 to 0.06)
Dominica	11.75% (-2.5 to 25.41)	4.21% (0.01 to 14.48)	-0.87% (-2.57 to 0.46)	0.21% (0.07 to 0.38)	0.26% (0.07 to 0.46)	1.63% (0.62 to 2.73)	6.46% (-6.93 to 18.64)	4.84% (-0.74 to 12.38)	0.25% (0.12 to 0.42)	0.12% (-0.01 to 0.32)	0.02% (0.01 to 0.04)
Dominican Republic	10.76% (0.53 to 22.07)	4.28% (0.02 to 14.22)	-1.12% (-3.17 to 0.56)	0.6% (0.31 to 0.93)	0.29% (0.07 to 0.5)	2.74% (1.5 to 4.16)	4.13% (-4.13 to 11.23)	4.83% (-0.69 to 12.38)	0.23% (0.11 to 0.39)	2.02% (-0.1 to 4.13)	0.01% (0 to 0.02)
Ecuador	15.45% (2.99 to 28.32)	6.38% (0.18 to 18.05)	-0.84% (-2.59 to 0.64)	0.85% (0.41 to 1.36)	0.28% (0.07 to 0.49)	5.05% (1.64 to 8.39)	4.29% (-4.34 to 12.33)	3.03% (-0.45 to 8.03)	0.52% (0.25 to 0.82)	3.52% (-0.2 to 7.26)	0.02% (0.01 to 0.03)
Egypt	7.98% (-3.41 to 18.01)	1.75% (0 to 8.57)	-1.34% (-3.71 to 0.67)	1.27% (0.71 to 1.86)	0.27% (0.07 to 0.46)	0.02% (0 to 0.06)	5.85% (-6.25 to 15.18)	0.22% (-0.02 to 0.64)	0.18% (0.09 to 0.3)	0.04% (0 to 0.1)	0.03% (0.01 to 0.05)
El Salvador	14.43% (3.37 to 26.93)	7.84% (0.5 to 20.15)	-0.5% (-1.38 to 0.27)	1.77% (0.85 to 2.8)	0.23% (0.06 to 0.41)	2.63% (0.95 to 4.29)	3.13% (-3.06 to 9.01)	2.84% (-0.43 to 7.81)	0.22% (0.1 to 0.36)	0.26% (-0.01 to 0.6)	0.02% (0.01 to 0.04)
Equatorial Guinea	14.53% (4.83 to 24.71)	3.1% (0 to 12.12)	-1.31% (-3.77 to 0.52)	1.83% (1.02 to 2.68)	0.67% (0.17 to 1.18)	7.14% (4.35 to 10)	3.65% (-3.69 to 9.56)	6.63% (-1.03 to 16.97)	0.43% (0.2 to 0.71)	0.24% (-0.01 to 0.56)	0.03% (0.01 to 0.06)
Eritrea	23.68% (9.28 to 37.11)	7.21% (0.27 to 19.62)	-0.79% (-2.19 to 0.42)	4.5% (2.61 to 6.46)	0.13% (0.03 to 0.23)	10.51% (6.69 to 14.34)	4.95% (-5.17 to 12.83)	1.33% (-0.26 to 3.98)	0.03% (0.02 to 0.06)	0.69% (-0.02 to 1.38)	0.04% (0.01 to 0.07)
Estonia	10.68% (0.74 to 21.23)	1.98% (0 to 9.06)	-0.71% (-2.22 to 0.48)	1.62% (0.6 to 2.72)	3.61% (0.84 to 6.66)	0.88% (0.11 to 1.63)	3.64% (-3.59 to 11.18)	8.79% (-0.71 to 24.07)	0.26% (0.12 to 0.42)	0.32% (-0.02 to 0.79)	0.02% (0.01 to 0.04)
Eswatini	14.76% (6.97 to 23.58)	2.49% (0 to 10.72)	-1.39% (-3.85 to 0.74)	3.03% (1.73 to 4.46)	0.16% (0.04 to 0.28)	8.9% (5.41 to 12.61)	2.46% (-2.39 to 6.59)	4.18% (-0.61 to 11.33)	0.07% (0.03 to 0.12)	1.07% (-0.05 to 2.24)	0.03% (0.01 to 0.06)
Ethiopia	27.4% (11.4 to 42.77)	7.18% (0.34 to 19.48)	-0.61% (-1.67 to 0.33)	11.2% (6.33 to 16.27)	0.21% (0.05 to 0.38)	8.19% (4.75 to 11.86)	6.04% (-6.46 to 16.26)	4.26% (-0.73 to 11.36)	0.05% (0.02 to 0.08)	0.18% (0 to 0.4)	0.03% (0.01 to 0.06)
Fiji	24.05% (7.2 to 38.72)	9.7% (1.07 to 22.75)	-1.47% (-4.15 to 0.83)	6.22% (3.67 to 9.08)	0.37% (0.1 to 0.64)	4.75% (2.91 to 6.7)	6.79% (-7.51 to 17.55)	2.43% (-0.34 to 6.43)	0.4% (0.18 to 0.66)	0.42% (-0.01 to 0.87)	0.02% (0.01 to 0.03)
Finland	10.34% (0.79 to 22.13)	3.96% (0.03 to 13.32)	0.27% (-0.34 to 1.44)	1.2% (0.06 to 2.34)	1.33% (0.32 to 2.55)	1.11% (-0.13 to 2.3)	2.81% (-2.71 to 9.53)	9.02% (-1.25 to 21.63)	0.35% (0.17 to 0.59)	0.74% (-0.26 to 2.09)	0.02% (0.01 to 0.04)
France	12.65% (-0.44 to 26.55)	3.62% (0.01 to 12.91)	1.59% (-0.94 to 4.51)	1.15% (0.04 to 2.27)	2.16% (0.53 to 3.91)	1.11% (-0.09 to 2.3)	3.95% (-3.98 to 13.36)	11.72% (-1.58 to 27.8)	0.41% (0.2 to 0.7)	1.12% (-0.18 to 2.96)	0.02% (0 to 0.03)
Gabon	12.99% (3.74 to 23.62)	3.13% (0 to 12.75)	-0.13% (-1.41 to 1.76)	1.14% (0.62 to 1.75)	0.52% (0.11 to 0.91)	6.06% (3.6 to 8.79)	2.92% (-2.78 to 7.79)	7.79% (-1.26 to 19.38)	0.24% (0.11 to 0.4)	0.56% (-0.02 to 1.18)	0.03% (0.01 to 0.06)
Gambia	26.27% (12.18 to 39.16)	3.82% (0 to 14.01)	-0.46% (-1.26 to 0.23)	13.67% (7.79 to 19.53)	0.41% (0.1 to 0.75)	8.75% (5.39 to 12.24)	4.43% (-4.59 to 11.65)	5.15% (-0.88 to 13.17)	0.04% (0.02 to 0.07)	1.03% (-0.05 to 2.11)	0.03% (0.01 to 0.06)
Georgia	14.99% (-0.31 to 30.22)	7.13% (0.36 to 19.3)	-0.76% (-2.22 to 0.39)	1.73% (0.67 to 2.88)	0.39% (0.09 to 0.7)	1.02% (0.3 to 1.79)	6.15% (-6.63 to 18.11)	5.65% (-0.63 to 16.12)	0.09% (0.04 to 0.14)	0.81% (-0.06 to 1.89)	0.02% (0.01 to 0.05)
Germany	11% (0.95 to 22.77)	4.25% (0.05 to 14.31)	1.01% (-0.63 to 3.05)	1.16% (0.18 to 2.15)	1.49% (0.35 to 2.66)	1.04% (0.08 to 2.02)	2.63% (-2.5 to 8.54)	12.96% (-1.95 to 30.35)	0.3% (0.14 to 0.48)	0.92% (-0.06 to 2.26)	0.02% (0 to 0.03)
Ghana	17.1% (1.54 to 30.99)	5.73% (0.05 to 17.44)	-0.74% (-2.02 to 0.36)	1.34% (0.74 to 1.95)	0.83% (0.19 to 1.49)	4.77% (2.94 to 6.6)	6.36% (-6.77 to 16.43)	7.23% (-1.3 to 18.04)	0.14% (0.07 to 0.24)	0% (0 to 0)	0.04% (0.01 to 0.06)
Greece	9.26% (-2.07 to 22.3)	3.6% (0.01 to 13.03)	0.71% (-0.35 to 2.11)	0.38% (-0.15 to 0.92)	1.19% (0.28 to 2.12)	0.12% (-0.13 to 0.41)	3.41% (-3.62 to 12.69)	7.74% (-0.96 to 18.91)	0.42% (0.21 to 0.75)	0.42% (-0.28 to 1.32)	0.02% (0.01 to 0.04)

Greenland	14.03% (-0.1 to 27.86)	5.13% (0.06 to 16.54)	2.33% (-1.37 to 6.21)	1.51% (0.76 to 2.34)	1.06% (0.25 to 1.94)	0.82% (0.34 to 1.47)	4.24% (-4.15 to 11.63)	8.51% (-1.29 to 21.52)	0.28% (0.14 to 0.47)	0.95% (-0.03 to 2.11)	0.02% (0 to 0.03)
Grenada	15.37% (0.67 to 29.83)	4.24% (0.01 to 14.55)	-0.58% (-1.62 to 0.32)	1.23% (0.61 to 1.92)	0.18% (0.04 to 0.31)	5.04% (2.4 to 7.7)	6.36% (-6.68 to 18.2)	5.12% (-0.77 to 13.18)	0.09% (0.04 to 0.15)	1.24% (-0.05 to 2.67)	0.01% (0 to 0.03)
Guam	19.76% (4.37 to 34.59)	9.57% (1.12 to 22.56)	-0.69% (-2.84 to 1.34)	1.76% (0.99 to 2.62)	0.33% (0.08 to 0.57)	3.54% (2.13 to 5.12)	6.05% (-6.54 to 15.59)	3.8% (-0.57 to 11.28)	0.76% (0.38 to 1.23)	0% (0 to 0.01)	0.02% (0.01 to 0.04)
Guatemala	15% (3 to 27.82)	7.82% (0.45 to 20.51)	-0.52% (-1.47 to 0.29)	1.95% (1.01 to 2.99)	0.18% (0.04 to 0.31)	2.9% (1.31 to 4.63)	3.53% (-3.6 to 9.69)	2.41% (-0.36 to 6.46)	0.15% (0.08 to 0.25)	0.32% (-0.01 to 0.68)	0.02% (0.01 to 0.04)
Guinea	13.98% (3.53 to 25.48)	3.81% (0 to 13.78)	-0.62% (-1.67 to 0.32)	1.98% (1.11 to 2.89)	0.42% (0.1 to 0.77)	5.52% (3.28 to 7.86)	3.8% (-3.8 to 10.22)	2.85% (-0.45 to 7.65)	0.04% (0.02 to 0.07)	0.89% (-0.04 to 1.79)	0.03% (0.01 to 0.06)
Guinea-Bissau	22.37% (10.31 to 33.2)	3.83% (0 to 14.13)	-1.46% (-3.99 to 0.76)	3.93% (2.24 to 5.56)	0.33% (0.08 to 0.58)	13.77% (8.86 to 18.26)	4.2% (-4.29 to 10.74)	5.51% (-0.96 to 13.96)	0.03% (0.01 to 0.04)	2.49% (-0.14 to 4.88)	0.03% (0.01 to 0.06)
Guyana	13.48% (2.2 to 25.48)	4.25% (0.01 to 14.64)	-0.35% (-0.97 to 0.18)	2.48% (1.36 to 3.75)	0.4% (0.09 to 0.71)	3.19% (1.79 to 4.68)	4.1% (-4.08 to 10.96)	5.21% (-0.82 to 13.27)	0.35% (0.17 to 0.57)	1.95% (-0.1 to 3.99)	0.03% (0.01 to 0.05)
Haiti	20.39% (6 to 33.05)	4.02% (0.01 to 13.86)	-0.68% (-1.88 to 0.36)	2.43% (1.37 to 3.58)	0.12% (0.03 to 0.21)	10.79% (6.67 to 15.02)	5.85% (-6.2 to 15.49)	4.83% (-0.62 to 12.41)	0.04% (0.02 to 0.07)	2.04% (-0.1 to 4.07)	0.02% (0.01 to 0.04)
Honduras	15.38% (3.73 to 27.93)	8.35% (0.48 to 21.48)	-0.67% (-1.87 to 0.33)	1.82% (0.94 to 2.83)	0.16% (0.04 to 0.28)	3.33% (1.62 to 5.02)	3.17% (-3.13 to 8.58)	2.78% (-0.4 to 7.23)	0.15% (0.07 to 0.23)	0.59% (-0.02 to 1.29)	0.03% (0.01 to 0.05)
Hungary	23.85% (9.14 to 38.02)	18.2% (6.39 to 32.4)	-0.6% (-2.08 to 0.6)	1.63% (0.69 to 2.68)	0.81% (0.18 to 1.4)	0.59% (0.13 to 1.13)	4.53% (-4.49 to 13.44)	9.93% (-1.24 to 23.8)	0.32% (0.15 to 0.51)	1.44% (-0.06 to 3.18)	0.02% (0.01 to 0.03)
Iceland	13.27% (0.92 to 26.56)	4.62% (0.06 to 14.57)	1.72% (-1.01 to 4.43)	1.15% (0.06 to 2.26)	2.01% (0.47 to 3.55)	1.36% (-0.18 to 2.76)	3.32% (-3.3 to 11.16)	9.59% (-1.16 to 23.33)	0.45% (0.21 to 0.75)	1.67% (-0.15 to 4.22)	0.02% (0 to 0.03)
India	21.87% (8.71 to 34.45)	5.92% (0.28 to 17.47)	-0.16% (-0.44 to 0.08)	11.45% (6.48 to 16.73)	0.12% (0.03 to 0.2)	3.04% (1.76 to 4.43)	3.99% (-3.94 to 10.73)	3.34% (-0.46 to 9.12)	0.18% (0.09 to 0.28)	1.45% (-0.04 to 2.89)	0.03% (0.01 to 0.06)
Indonesia	20.3% (7.76 to 33.38)	12.57% (2.4 to 26.36)	-0.41% (-1.13 to 0.2)	2.8% (1.56 to 4.08)	0.08% (0.02 to 0.14)	4.17% (2.48 to 5.93)	2.56% (-2.38 to 6.81)	0.42% (-0.06 to 1.15)	0.03% (0.02 to 0.05)	3.41% (-0.17 to 6.71)	0.03% (0.01 to 0.06)
Iran (Islamic Republic of)	11.41% (-7.95 to 26.5)	1.62% (0 to 7.86)	-0.97% (-2.69 to 0.53)	0.98% (0.58 to 1.44)	0.2% (0.05 to 0.35)	0.38% (0.13 to 0.73)	9.2% (-11.2 to 23.82)	0.56% (-0.07 to 1.64)	0.18% (0.09 to 0.31)	0.57% (-0.01 to 1.17)	0.02% (0.01 to 0.03)
Iraq	12.87% (-6.18 to 27.74)	1.8% (0 to 8.45)	-0.45% (-1.21 to 0.23)	2.44% (1.4 to 3.55)	0.21% (0.05 to 0.35)	0.64% (0.28 to 1.09)	8.77% (-10.14 to 22.43)	0.19% (-0.02 to 0.57)	0.1% (0.05 to 0.16)	1.64% (-0.08 to 3.28)	0.02% (0.01 to 0.04)
Ireland	10.09% (0.24 to 20.98)	2.77% (0 to 11.13)	2.36% (-1.54 to 6.49)	1.05% (0.03 to 2.1)	1.61% (0.38 to 2.87)	0.69% (-0.11 to 1.47)	1.73% (-1.62 to 6.05)	9.54% (-1.22 to 23.09)	0.7% (0.33 to 1.15)	0.53% (-0.08 to 1.4)	0.02% (0.01 to 0.04)
Israel	9.82% (-1.88 to 22.53)	4.03% (0.02 to 14.04)	-0.63% (-2.13 to 0.45)	0.59% (0.13 to 1.09)	0.73% (0.17 to 1.36)	0.07% (-0.05 to 0.23)	4.88% (-5.27 to 15.14)	3.93% (-0.43 to 9.97)	0.5% (0.23 to 0.84)	0.21% (-0.04 to 0.56)	0.01% (0 to 0.02)
Italy	11.26% (-0.75 to 24.91)	4.53% (0.11 to 14.46)	0.37% (-0.18 to 1.6)	0.5% (-0.18 to 1.13)	2.04% (0.48 to 3.58)	0.49% (-0.19 to 1.17)	3.67% (-3.94 to 13.25)	10.02% (-1.19 to 23.59)	0.41% (0.19 to 0.71)	0.62% (-0.23 to 1.77)	0.01% (0 to 0.03)
Jamaica	10.8% (-0.19 to 22.66)	4.31% (0.02 to 14.38)	-0.49% (-1.45 to 0.26)	1.21% (0.44 to 2.06)	0.11% (0.03 to 0.19)	1.74% (0.36 to 3.13)	4.32% (-4.44 to 13.05)	3.29% (-0.41 to 8.68)	0.09% (0.04 to 0.15)	0.62% (-0.07 to 1.51)	0.02% (0.01 to 0.03)
Japan	14.1% (2.83 to 26.95)	9.27% (0.83 to 22.03)	-0.48% (-1.56 to 0.23)	1.48% (0.23 to 2.75)	1.62% (0.41 to 2.8)	0.29% (-0.06 to 0.68)	2.17% (-2.08 to 7.31)	8.38% (-1.29 to 19.52)	0.44% (0.22 to 0.73)	0.82% (-0.1 to 2.14)	0.02% (0 to 0.03)

Jordan	14.17% (-7.37 to 30.11)	1.64% (0 to 8)	-1.21% (-3.3 to 0.64)	2.44% (1.37 to 3.53)	0.29% (0.07 to 0.51)	1.41% (0.78 to 2.13)	10% (-12.28 to 25.48)	0.28% (-0.03 to 0.83)	0.27% (0.13 to 0.46)	1.91% (-0.1 to 3.77)	0.02% (0.01 to 0.03)
Kazakhstan	18.72% (-4.49 to 36.56)	6.46% (0.29 to 18.03)	2.71% (-1.64 to 6.79)	2.26% (1.2 to 3.5)	0.57% (0.13 to 1)	0.22% (0.03 to 0.51)	8.51% (-9.78 to 22.56)	5.97% (-0.76 to 16.27)	0.21% (0.1 to 0.33)	1.19% (-0.06 to 2.56)	0.02% (0.01 to 0.04)
Kenya	14.55% (3.9 to 24.62)	4.97% (0.31 to 14.04)	-0.96% (-2.76 to 0.51)	3.27% (1.86 to 4.84)	0.25% (0.06 to 0.47)	3.82% (2.28 to 5.61)	4.09% (-4.12 to 10.95)	5.22% (-0.94 to 13.32)	0.09% (0.04 to 0.15)	0.4% (-0.01 to 0.83)	0.03% (0.01 to 0.06)
Kiribati	25.07% (5.36 to 41.1)	8.89% (1.13 to 21)	-1.34% (-3.69 to 0.64)	3.33% (1.92 to 4.65)	0.29% (0.07 to 0.51)	7.59% (4.94 to 10.18)	8.82% (-10.08 to 22.16)	1.05% (-0.15 to 3.88)	0.22% (0.11 to 0.38)	0.86% (-0.04 to 1.69)	0.04% (0.01 to 0.07)
Kuwait	15.14% (-4.38 to 29.98)	3.86% (0.03 to 13.33)	-1.45% (-4.56 to 1.57)	2.84% (1.7 to 3.93)	0.33% (0.08 to 0.59)	0.39% (0.14 to 0.77)	9.43% (-11.38 to 23.5)	0.1% (-0.01 to 0.38)	0.54% (0.26 to 0.89)	1.87% (-0.1 to 3.68)	0.02% (0.01 to 0.03)
Kyrgyzstan	20.04% (-3.73 to 38.6)	6.03% (0.26 to 17.8)	-0.89% (-3.09 to 1.6)	3.85% (2.19 to 5.54)	0.71% (0.16 to 1.27)	0.54% (0.22 to 0.97)	11.29% (-13.47 to 28.56)	5.05% (-0.75 to 13.52)	0.17% (0.08 to 0.28)	1.32% (-0.07 to 2.76)	0.03% (0.01 to 0.06)
Lao People's Democratic Republic	17.62% (6.27 to 30.65)	12.23% (2.04 to 26.64)	-1.3% (-3.56 to 0.67)	2.22% (1.24 to 3.21)	0.07% (0.02 to 0.12)	3.22% (1.87 to 4.72)	1.93% (-1.83 to 5.14)	8% (-1.53 to 19.64)	0.05% (0.02 to 0.08)	5.44% (-0.33 to 10.47)	0.03% (0.01 to 0.06)
Latvia	13.21% (1.35 to 26.21)	4.05% (0.06 to 13.33)	-0.66% (-2.15 to 0.38)	1.86% (0.56 to 3.31)	3.94% (0.79 to 7.49)	0.75% (0.08 to 1.45)	4.04% (-3.96 to 12.69)	8.78% (-0.74 to 24.61)	0.16% (0.07 to 0.28)	0.82% (-0.06 to 2.06)	0.02% (0 to 0.03)
Lebanon	9% (-6.09 to 21.78)	1.45% (0 to 7.29)	-1.2% (-3.54 to 0.64)	0.87% (0.44 to 1.38)	0.24% (0.06 to 0.41)	0.07% (-0.01 to 0.21)	7.38% (-8.66 to 20.84)	1.5% (-0.15 to 4.4)	0.19% (0.09 to 0.32)	0.28% (-0.01 to 0.65)	0.01% (0 to 0.03)
Lesotho	17.69% (9.41 to 27.34)	2.41% (0 to 10.92)	-1.07% (-2.99 to 0.59)	8.11% (4.52 to 11.98)	0.14% (0.04 to 0.24)	7.79% (4.59 to 10.97)	1.78% (-1.71 to 4.77)	4.34% (-0.69 to 11.32)	0.11% (0.05 to 0.19)	0.01% (0 to 0.02)	0.03% (0.01 to 0.06)
Liberia	21.14% (7.31 to 33.71)	3.8% (0 to 14.04)	-0.49% (-1.37 to 0.27)	4.39% (2.59 to 6.31)	0.48% (0.11 to 0.86)	10.33% (6.56 to 14.06)	5.29% (-5.61 to 13.6)	5.64% (-0.98 to 13.81)	0.04% (0.02 to 0.07)	4.36% (-0.25 to 8.3)	0.04% (0.01 to 0.07)
Libya	14.48% (-7.99 to 29.91)	1.63% (0 to 8.31)	-1.47% (-4 to 0.78)	2.33% (1.34 to 3.29)	0.17% (0.04 to 0.3)	1.5% (0.87 to 2.23)	10.73% (-13.25 to 27.12)	0.3% (-0.03 to 0.89)	0.14% (0.06 to 0.23)	1.6% (-0.08 to 3.14)	0.02% (0.01 to 0.03)
Lithuania	14.35% (1.01 to 27.8)	4.33% (0.01 to 15.16)	0.08% (-0.8 to 1.46)	1.59% (0.59 to 2.68)	4.53% (1 to 8.36)	0.78% (0.13 to 1.46)	3.92% (-3.82 to 11.79)	8.95% (-0.76 to 24.46)	0.27% (0.12 to 0.46)	0.31% (-0.02 to 0.79)	0.02% (0.01 to 0.04)
Luxembourg	11.1% (0.28 to 23.39)	3.97% (0.02 to 13.69)	1.66% (-0.92 to 4.31)	1.03% (0.1 to 1.97)	1.74% (0.4 to 3.19)	0.81% (-0.17 to 1.7)	2.63% (-2.51 to 8.66)	11.96% (-1.59 to 28.16)	0.3% (0.14 to 0.5)	0.91% (-0.15 to 2.4)	0.02% (0.01 to 0.04)
Madagascar	24.09% (11.57 to 36.25)	7.16% (0.25 to 19.8)	-1.08% (-2.9 to 0.58)	4.41% (2.59 to 6.33)	0.18% (0.04 to 0.31)	12.54% (8.02 to 16.69)	3.43% (-3.51 to 8.91)	2.32% (-0.33 to 5.99)	0.05% (0.02 to 0.08)	2.51% (-0.13 to 4.78)	0.04% (0.01 to 0.07)
Malawi	19.26% (7.96 to 31.7)	7.43% (0.28 to 19.75)	-0.49% (-1.34 to 0.27)	2.74% (1.51 to 3.97)	0.2% (0.05 to 0.35)	8.4% (5.2 to 11.66)	2.49% (-2.34 to 6.62)	3.39% (-0.64 to 8.71)	0.07% (0.03 to 0.12)	0.02% (0 to 0.05)	0.03% (0.01 to 0.06)
Malaysia	20.42% (6.01 to 34.49)	12.41% (2.08 to 26.38)	-0.84% (-2.32 to 0.43)	1.99% (1.09 to 2.95)	0.42% (0.1 to 0.74)	3.07% (1.78 to 4.54)	4.65% (-4.66 to 12.4)	1.37% (-0.21 to 3.8)	0.26% (0.12 to 0.41)	3.49% (-0.19 to 6.99)	0.02% (0.01 to 0.04)
Maldives	20.61% (3.71 to 35.72)	11.28% (1.7 to 24.52)	-0.35% (-0.97 to 0.19)	2.64% (1.51 to 3.83)	0.08% (0.02 to 0.15)	2.12% (1.08 to 3.24)	6.58% (-7.59 to 17.35)	1.05% (-0.11 to 3.09)	0.05% (0.02 to 0.09)	3.73% (-0.22 to 7.23)	0.03% (0.01 to 0.05)
Mali	18.1% (6.06 to 29.82)	3.66% (0 to 13.44)	-1.62% (-4.49 to 0.85)	4.14% (2.35 to 5.98)	0.9% (0.22 to 1.61)	7.66% (4.82 to 10.56)	4.98% (-5.06 to 13.12)	1.78% (-0.27 to 4.86)	0.12% (0.06 to 0.19)	0.17% (-0.01 to 0.35)	0.04% (0.01 to 0.07)
Malta	11.49% (-0.64 to 24.56)	4.67% (0.09 to 15.16)	-0.24% (-1.27 to 0.82)	1% (0.06 to 1.97)	1.76% (0.41 to 3.18)	0.31% (-0.15 to 0.75)	4.3% (-4.49 to 14.32)	7.5% (-0.98 to 18.78)	0.43% (0.21 to 0.68)	0.52% (-0.1 to 1.54)	0.01% (0 to 0.03)

Marshall Islands	27.01% (8.42 to 42.61)	8.76% (1.03 to 20.67)	-1.76% (-4.91 to 0.89)	4.35% (2.44 to 6.04)	0.22% (0.05 to 0.37)	10.11% (6.62 to 13.39)	8.47% (-9.73 to 21.31)	2.19% (-0.32 to 6.37)	0.16% (0.08 to 0.26)	0.31% (-0.01 to 0.71)	0.03% (0.01 to 0.06)
Mauritania	24.28% (7.35 to 38.89)	3.79% (0 to 13.8)	-1.4% (-3.87 to 0.75)	10.04% (5.81 to 14.82)	0.58% (0.12 to 1.05)	7.93% (4.59 to 11.4)	7.11% (-7.85 to 18.7)	0% (0 to 0)	0.09% (0.04 to 0.15)	0.56% (-0.02 to 1.2)	0.02% (0.01 to 0.04)
Mauritius	21.29% (6.68 to 35.36)	12.27% (1.99 to 26.78)	-0.89% (-2.51 to 0.48)	4.09% (2.27 to 6.11)	0.17% (0.05 to 0.3)	2.25% (1.26 to 3.38)	4.97% (-5.19 to 13.47)	5.1% (-0.94 to 12.52)	0.3% (0.14 to 0.5)	2.12% (-0.11 to 4.28)	0.01% (0 to 0.02)
Mexico	11.97% (2.59 to 22.99)	5.46% (0.13 to 16.37)	-0.22% (-1.24 to 1.43)	1.5% (0.8 to 2.26)	0.4% (0.1 to 0.71)	2.5% (1.25 to 3.85)	2.46% (-2.37 to 6.84)	4.53% (-0.74 to 11.5)	0.59% (0.28 to 0.96)	0.23% (0 to 0.49)	0.02% (0.01 to 0.04)
Micronesia (Federated States of)	26.08% (7.63 to 41.1)	8.93% (0.96 to 21.47)	-1.56% (-4.36 to 0.76)	4.05% (2.23 to 5.83)	0.19% (0.05 to 0.34)	9.63% (6.29 to 13.08)	7.85% (-8.81 to 20.25)	2.47% (-0.37 to 7.01)	0.13% (0.06 to 0.24)	0.3% (-0.01 to 0.69)	0.03% (0.01 to 0.06)
Monaco	12.72% (0.13 to 26.18)	3.7% (0.02 to 13.42)	1.73% (-0.96 to 5.28)	0.35% (-0.03 to 0.79)	3.37% (0.84 to 6.03)	0.1% (-0.04 to 0.32)	2.91% (-2.81 to 10.1)	7.5% (-0.89 to 22.97)	1.64% (0.76 to 2.67)	0.1% (-0.04 to 0.31)	0.01% (0 to 0.02)
Mongolia	36.19% (1.56 to 57.88)	6.58% (0.33 to 18.39)	8.56% (-5.65 to 19.69)	14.28% (8.54 to 19.65)	0.98% (0.24 to 1.73)	5.07% (3.21 to 6.92)	12.02% (-15.46 to 30.06)	7.59% (-1.2 to 19.34)	0.2% (0.1 to 0.32)	6.3% (-0.37 to 12.01)	0.03% (0.01 to 0.05)
Montenegro	21.75% (6.25 to 36.05)	16.23% (4.55 to 31.08)	0.02% (-0.88 to 1.43)	0.54% (0.12 to 1.05)	1.48% (0.35 to 2.72)	0.08% (-0.05 to 0.28)	4.43% (-4.47 to 13.63)	8.32% (-0.86 to 19.68)	0.27% (0.12 to 0.45)	0.34% (-0.05 to 0.86)	0.02% (0.01 to 0.04)
Morocco	11.64% (-6.95 to 26.73)	1.58% (0 to 8.23)	-1.12% (-3.17 to 0.56)	1.11% (0.59 to 1.69)	0.22% (0.05 to 0.39)	0.73% (0.34 to 1.27)	9.19% (-10.63 to 24.63)	0.11% (-0.01 to 0.32)	0.15% (0.07 to 0.23)	0.01% (0 to 0.03)	0.02% (0.01 to 0.04)
Mozambique	26.26% (9.85 to 40.54)	7.46% (0.21 to 20.21)	-0.48% (-1.31 to 0.24)	7.04% (4.1 to 9.98)	0.13% (0.03 to 0.23)	10.13% (6.29 to 13.83)	5.78% (-5.95 to 15.12)	3.27% (-0.59 to 8.61)	0.04% (0.02 to 0.06)	0.6% (-0.03 to 1.23)	0.03% (0.01 to 0.05)
Myanmar	19.67% (7.78 to 33.06)	12.5% (2.18 to 26.67)	-0.91% (-2.59 to 0.49)	3.87% (2.12 to 5.66)	0.09% (0.02 to 0.15)	2.62% (1.48 to 3.84)	2.75% (-2.66 to 7.57)	3.64% (-0.58 to 9.13)	0.06% (0.03 to 0.11)	4.39% (-0.25 to 8.71)	0.03% (0.01 to 0.06)
Namibia	14.8% (4.96 to 25.19)	2.38% (0 to 10.58)	-1.21% (-3.37 to 0.58)	5.75% (3.01 to 8.89)	0.17% (0.05 to 0.31)	5.2% (2.57 to 7.92)	3.71% (-3.77 to 10.22)	8.36% (-1.42 to 20.87)	0.05% (0.02 to 0.09)	1.84% (-0.09 to 3.75)	0.03% (0.01 to 0.06)
Nauru	24.55% (5.74 to 39.77)	8.72% (0.88 to 21.34)	-1.76% (-5.11 to 0.99)	3.34% (2 to 4.71)	0.24% (0.06 to 0.43)	7.9% (5.01 to 10.67)	8.34% (-9.58 to 20.92)	4.58% (-0.77 to 11.78)	0.27% (0.12 to 0.44)	0.09% (0 to 0.23)	0.03% (0.01 to 0.06)
Nepal	13.81% (3.55 to 25.95)	6.09% (0.08 to 18.08)	-0.81% (-2.26 to 0.43)	2.68% (1.37 to 4.12)	0.71% (0.16 to 1.35)	3.08% (1.67 to 4.66)	2.83% (-2.77 to 7.69)	3.06% (-0.47 to 8.86)	0.04% (0.02 to 0.07)	1.02% (-0.05 to 2.13)	0.02% (0.01 to 0.04)
Netherlands	10.65% (0.27 to 22.54)	3.56% (0.01 to 13.3)	1.02% (-0.63 to 3.06)	0.97% (0.07 to 1.88)	1.3% (0.31 to 2.26)	1.17% (-0.05 to 2.32)	3% (-2.9 to 10.01)	11.2% (-1.46 to 26.58)	0.49% (0.24 to 0.82)	0.93% (-0.16 to 2.51)	0.01% (0 to 0.03)
New Zealand	11.76% (-0.13 to 24.07)	3.37% (0.01 to 12.48)	2.21% (-1.37 to 6.03)	0.99% (0.1 to 1.91)	2.16% (0.53 to 3.79)	0.88% (-0.08 to 1.82)	2.76% (-2.73 to 9.2)	10.93% (-1.35 to 26.54)	0.56% (0.28 to 0.9)	0.98% (-0.14 to 2.61)	0.02% (0.01 to 0.04)
Nicaragua	23% (10.06 to 35.91)	8.13% (0.51 to 21)	-0.52% (-1.47 to 0.28)	5.73% (3.19 to 8.59)	0.16% (0.04 to 0.28)	9.35% (5.44 to 13.45)	3.21% (-3.23 to 8.68)	3.57% (-0.59 to 9.5)	0.13% (0.06 to 0.21)	1.12% (-0.05 to 2.42)	0.02% (0.01 to 0.04)
Niger	17.64% (4.52 to 29.79)	3.79% (0 to 13.32)	-1.44% (-4.03 to 0.8)	7.84% (4.52 to 11.55)	0.52% (0.12 to 0.94)	3.05% (1.79 to 4.48)	5.43% (-5.84 to 14.52)	0.56% (-0.05 to 2.01)	0.06% (0.03 to 0.1)	0.31% (-0.01 to 0.69)	0.04% (0.01 to 0.07)
Nigeria	13.12% (0.7 to 24.64)	3.29% (0.01 to 12.62)	-0.68% (-1.9 to 0.37)	2.94% (1.65 to 4.27)	0.39% (0.1 to 0.72)	2.77% (1.55 to 4)	5.26% (-5.69 to 13.5)	6.87% (-1.23 to 16.46)	0.05% (0.02 to 0.09)	0.1% (0 to 0.23)	0.04% (0.01 to 0.07)
Niue	19.88% (5.15 to 34.4)	10.08% (1.14 to 23.58)	-0.96% (-2.87 to 0.56)	1.9% (0.97 to 3.03)	0.35% (0.1 to 0.61)	4.75% (2.35 to 7.26)	5% (-5.16 to 14.06)	3.85% (-0.59 to 10.54)	0.38% (0.19 to 0.66)	0.03% (0 to 0.08)	0.03% (0.01 to 0.05)

North Macedonia	22% (5.97 to 37.01)	16.48% (4.53 to 31.31)	-0.72% (-2.13 to 0.37)	1% (0.34 to 1.73)	0.81% (0.2 to 1.51)	0.13% (-0.02 to 0.35)	5.13% (-5.24 to 15.49)	8.02% (-0.97 to 18.7)	0.31% (0.14 to 0.54)	0.51% (-0.04 to 1.27)	0.02% (0.01 to 0.04)
Northern Mariana Islands	20.79% (5.39 to 35.24)	9.95% (1.15 to 23.25)	-1.05% (-3.37 to 0.93)	1.95% (1.09 to 2.91)	0.27% (0.07 to 0.49)	4.53% (2.76 to 6.43)	6.35% (-6.48 to 16.53)	3.38% (-0.4 to 10.33)	0.47% (0.23 to 0.79)	0.01% (0 to 0.03)	0.03% (0.01 to 0.05)
Norway	14.41% (1.5 to 27.59)	3.38% (0.02 to 11.97)	0.56% (-0.33 to 2.01)	0.9% (0.01 to 1.79)	5.31% (1.28 to 9.37)	1.4% (-0.14 to 2.87)	3.65% (-3.66 to 12.05)	8.84% (-1.1 to 21.64)	0.59% (0.29 to 0.94)	0.86% (-0.28 to 2.39)	0.01% (0 to 0.02)
Oman	11.89% (-5.46 to 24.93)	1.88% (0 to 8.85)	-2.19% (-6.15 to 0.96)	1.58% (0.93 to 2.24)	0.28% (0.07 to 0.49)	1.45% (0.83 to 2.15)	8.85% (-10.6 to 21.38)	0.43% (-0.05 to 1.28)	0.22% (0.1 to 0.38)	0.9% (-0.04 to 1.78)	0.03% (0.01 to 0.06)
Pakistan	23.52% (2.75 to 40.5)	6.24% (0.13 to 18.33)	-1.07% (-2.96 to 0.56)	4.54% (2.56 to 6.69)	0.87% (0.21 to 1.59)	6.65% (4.04 to 9.41)	9.43% (-11.17 to 24.31)	0.82% (-0.13 to 2.24)	0.04% (0.02 to 0.07)	3.53% (-0.19 to 6.94)	0.03% (0.01 to 0.06)
Palau	22.67% (5.45 to 37.47)	9.8% (1.1 to 23.47)	-1.37% (-4.13 to 0.9)	2.5% (1.38 to 3.65)	0.34% (0.08 to 0.58)	6.13% (3.89 to 8.57)	6.94% (-7.37 to 18.08)	4.44% (-0.6 to 12.27)	0.42% (0.2 to 0.7)	0.03% (0 to 0.08)	0.03% (0.01 to 0.05)
Palestine	12.4% (-5.89 to 26.85)	1.55% (0 to 7.88)	-0.62% (-1.73 to 0.32)	2.17% (1.2 to 3.17)	0.13% (0.03 to 0.24)	1.3% (0.55 to 2.13)	8.45% (-9.97 to 22.07)	0.67% (-0.08 to 2.03)	0.04% (0.02 to 0.06)	3.39% (-0.18 to 6.7)	0.03% (0.01 to 0.05)
Panama	13.79% (3.67 to 25.71)	8.12% (0.47 to 20.73)	-0.83% (-2.53 to 0.49)	1.49% (0.59 to 2.49)	0.2% (0.05 to 0.36)	3.55% (1.04 to 5.97)	1.83% (-1.68 to 5.5)	4.28% (-0.62 to 11.43)	0.16% (0.08 to 0.27)	2.68% (-0.19 to 5.99)	0.01% (0 to 0.02)
Papua New Guinea	22.74% (4.15 to 38.46)	8.82% (0.87 to 20.94)	-1.34% (-3.75 to 0.7)	3.67% (2.06 to 5.33)	0.11% (0.03 to 0.19)	5.44% (3.44 to 7.45)	8.17% (-9.41 to 21.22)	1.08% (-0.15 to 3)	0.05% (0.02 to 0.08)	0.07% (0 to 0.16)	0.03% (0.01 to 0.06)
Paraguay	15.4% (2.5 to 28.87)	7.04% (0.2 to 19.06)	1.71% (-1.04 to 4.79)	1.22% (0.59 to 2)	0.27% (0.06 to 0.48)	3.45% (1.47 to 5.48)	2.99% (-2.82 to 8.76)	8.28% (-1.18 to 20.27)	0.15% (0.07 to 0.26)	0.25% (-0.01 to 0.59)	0.01% (0 to 0.02)
Peru	13.68% (2.57 to 25.87)	6.56% (0.18 to 18.64)	-0.78% (-2.18 to 0.43)	1.72% (0.86 to 2.73)	0.12% (0.03 to 0.21)	3.08% (1.33 to 4.88)	3.54% (-3.58 to 9.82)	6.16% (-1.04 to 15.43)	0.22% (0.11 to 0.36)	0.68% (-0.03 to 1.48)	0.02% (0.01 to 0.04)
Philippines	18.74% (6.86 to 31.84)	11.87% (2.28 to 25.63)	-1.27% (-3.61 to 0.65)	2.08% (1.19 to 2.97)	0.17% (0.04 to 0.29)	3.68% (2.28 to 5.17)	3.18% (-3.12 to 8.29)	7.71% (-1.48 to 18.34)	0.13% (0.06 to 0.21)	5.52% (-0.32 to 10.48)	0.03% (0.01 to 0.06)
Poland	19.03% (3.68 to 34.08)	11.72% (1.96 to 25.43)	1.22% (-0.75 to 3.61)	1.67% (0.59 to 2.82)	0.97% (0.24 to 1.68)	0.34% (0.02 to 0.73)	4.01% (-3.96 to 12.13)	9.32% (-1.18 to 22.02)	0.63% (0.3 to 1.02)	0.38% (-0.02 to 0.91)	0.02% (0.01 to 0.04)
Portugal	8.62% (-1.19 to 20.24)	3.06% (0.03 to 11.76)	1.25% (-0.82 to 3.54)	0.73% (-0.16 to 1.67)	0.86% (0.22 to 1.58)	0.28% (-0.18 to 0.73)	2.68% (-2.66 to 9.66)	9.56% (-1.24 to 22.96)	0.39% (0.19 to 0.67)	0.54% (-0.21 to 1.64)	0.02% (0 to 0.03)
Puerto Rico	12.41% (1.49 to 25.1)	4.48% (0.02 to 14.9)	-0.65% (-2.01 to 0.31)	1.02% (0.24 to 1.84)	0.36% (0.1 to 0.64)	4.06% (0.39 to 7.65)	3.64% (-3.66 to 11.17)	3.7% (-0.5 to 9.97)	0.24% (0.12 to 0.42)	0.87% (-0.08 to 2.19)	0.01% (0 to 0.02)
Qatar	16.5% (-12.52 to 35.24)	1.97% (0 to 8.69)	-2.4% (-7.16 to 1.59)	1.35% (0.72 to 2.04)	0.49% (0.12 to 0.87)	0.12% (0.01 to 0.32)	13.85% (-17.96 to 32.8)	0.8% (-0.1 to 2.33)	1.27% (0.58 to 2.07)	0.09% (0 to 0.26)	0.02% (0.01 to 0.04)
Republic of Korea	18.08% (5.45 to 31.22)	14.12% (3.1 to 28.21)	-0.09% (-1.17 to 1.19)	1.32% (0.41 to 2.27)	0.87% (0.23 to 1.47)	0.47% (-0.1 to 1.05)	1.89% (-1.79 to 5.9)	8.01% (-1.38 to 19.46)	0.31% (0.15 to 0.49)	2.66% (-0.19 to 6.01)	0.01% (0 to 0.02)
Republic of Moldova	11.3% (0.66 to 22.46)	3.12% (0 to 12.28)	-0.94% (-2.62 to 0.49)	2.15% (1.13 to 3.37)	1.44% (0.32 to 2.54)	1.45% (0.74 to 2.26)	4.58% (-4.46 to 12.9)	10.07% (-0.83 to 26.48)	0.08% (0.04 to 0.13)	0.63% (-0.03 to 1.35)	0.02% (0.01 to 0.04)
Romania	22.52% (7.69 to 36.46)	16.33% (4.55 to 31.15)	0.84% (-0.43 to 2.92)	1.14% (0.22 to 2.04)	1.95% (0.5 to 3.38)	0.03% (-0.03 to 0.11)	2.99% (-2.85 to 9.55)	9.74% (-1.11 to 23.52)	0.91% (0.45 to 1.43)	0.24% (-0.03 to 0.65)	0.02% (0.01 to 0.04)

Russian Federation	15.48% (1.22 to 29.31)	6.54% (0.4 to 18.1)	-0.26% (-1.35 to 1.17)	2% (0.89 to 3.22)	1.91% (0.44 to 3.4)	1.29% (0.59 to 2.04)	5.11% (-5.13 to 14.72)	6.64% (-0.64 to 17.75)	0.2% (0.1 to 0.32)	1.08% (-0.03 to 2.5)	0.02% (0.01 to 0.04)
Rwanda	17.85% (2.75 to 31.68)	7.56% (0.24 to 20.25)	-0.47% (-1.33 to 0.25)	0.11% (0.05 to 0.19)	0.12% (0.03 to 0.2)	6.25% (3.48 to 9.08)	5.52% (-5.97 to 14.81)	5.78% (-0.94 to 14.74)	0.03% (0.02 to 0.05)	0% (0 to 0.01)	0.04% (0.01 to 0.06)
Saint Kitts and Nevis	15.18% (1.63 to 28.52)	4.39% (0.01 to 14.75)	-1.02% (-2.8 to 0.51)	3.39% (1.72 to 5.32)	0.2% (0.05 to 0.34)	3.31% (1.66 to 4.93)	5.95% (-6.33 to 16.54)	3.14% (-0.32 to 10.99)	0.11% (0.05 to 0.19)	4.55% (-0.27 to 9.27)	0.02% (0.01 to 0.03)
Saint Lucia	13.37% (1 to 27.15)	4.4% (0.02 to 14.98)	-0.92% (-2.75 to 0.5)	1.28% (0.51 to 2.11)	0.19% (0.05 to 0.32)	4.02% (1.24 to 6.69)	5.14% (-5.35 to 15.36)	5.91% (-0.9 to 15.25)	0.11% (0.05 to 0.19)	2.07% (-0.1 to 4.66)	0.02% (0.01 to 0.04)
Saint Vincent and the Grenadines	12.99% (0.93 to 25.51)	4.35% (0.02 to 14.2)	-0.72% (-2.09 to 0.37)	1.15% (0.49 to 1.93)	0.19% (0.05 to 0.34)	3.6% (1.46 to 5.82)	5.01% (-5.12 to 14.29)	6.9% (-0.86 to 17.21)	0.17% (0.08 to 0.27)	1.43% (-0.07 to 3.25)	0.02% (0.01 to 0.04)
Samoa	21.54% (4.85 to 35.68)	3.51% (0.03 to 12.19)	-1.21% (-3.68 to 0.71)	2.8% (1.6 to 4.16)	0.29% (0.08 to 0.51)	10.56% (6.45 to 14.71)	7.69% (-8.96 to 20.41)	1.82% (-0.28 to 4.88)	0.34% (0.16 to 0.58)	0.01% (0 to 0.02)	0.03% (0.01 to 0.05)
San Marino	11.33% (0.13 to 23.73)	3.83% (0.02 to 13.27)	0.83% (-0.4 to 2.82)	0.82% (-0.04 to 1.64)	2.2% (0.49 to 3.97)	0.64% (-0.13 to 1.37)	3.28% (-3.2 to 11.44)	9.98% (-1.28 to 27.26)	0.61% (0.28 to 0.99)	0.64% (-0.16 to 1.81)	0.02% (0 to 0.03)
Sao Tome and Principe	18.79% (0.84 to 32.75)	3.85% (0 to 14.13)	-0.25% (-0.66 to 0.13)	1.49% (0.79 to 2.15)	0.51% (0.13 to 0.92)	7.47% (4.39 to 10.37)	7.68% (-8.86 to 19.41)	7.05% (-1.25 to 17.37)	0.06% (0.03 to 0.1)	0.02% (0 to 0.05)	0.03% (0.01 to 0.06)
Saudi Arabia	17.41% (-5.29 to 33)	1.82% (0 to 8.37)	-1.75% (-4.84 to 0.84)	3.33% (1.91 to 4.5)	0.36% (0.09 to 0.63)	2.43% (1.51 to 3.46)	11.12% (-12.78 to 27.04)	0.19% (-0.02 to 0.63)	1.27% (0.58 to 2.05)	0.89% (-0.04 to 1.76)	0.04% (0.01 to 0.06)
Senegal	16.77% (4.79 to 28.81)	3.86% (0 to 13.67)	-0.81% (-2.21 to 0.43)	5.98% (3.45 to 8.67)	0.51% (0.12 to 0.9)	4.1% (2.31 to 6.05)	4.72% (-4.9 to 12.3)	0.92% (-0.11 to 2.67)	0.03% (0.01 to 0.05)	0.14% (-0.01 to 0.31)	0.02% (0.01 to 0.04)
Serbia	20.98% (6.09 to 35.59)	16.35% (4.34 to 30.41)	-0.73% (-2.23 to 0.34)	0.83% (0.18 to 1.5)	0.55% (0.13 to 1.02)	0.79% (0.11 to 1.5)	3.94% (-3.88 to 12.2)	7.17% (-0.85 to 17.42)	0.24% (0.12 to 0.41)	0.77% (-0.06 to 1.89)	0.02% (0.01 to 0.04)
Seychelles	19.74% (7.42 to 32.71)	10.9% (1.47 to 24.39)	-0.64% (-1.83 to 0.31)	2.21% (1.16 to 3.31)	0.15% (0.03 to 0.26)	5.31% (3.03 to 7.68)	3.2% (-3.08 to 8.62)	4.35% (-0.74 to 10.88)	0.08% (0.04 to 0.14)	1.72% (-0.09 to 3.5)	0.03% (0.01 to 0.05)
Sierra Leone	17.26% (5.13 to 28.57)	3.86% (0 to 13.79)	-0.37% (-1 to 0.19)	4.81% (2.79 to 6.9)	0.34% (0.08 to 0.6)	6.02% (3.61 to 8.42)	4.38% (-4.63 to 11.56)	4.51% (-0.73 to 11.18)	0.02% (0.01 to 0.04)	4.63% (-0.26 to 9.01)	0.04% (0.01 to 0.07)
Singapore	16.79% (4.29 to 30.4)	11.36% (1.65 to 24.79)	0.04% (-1.15 to 2.09)	1.33% (0.71 to 2.06)	0.81% (0.19 to 1.37)	0.71% (0.29 to 1.23)	3.06% (-3.03 to 8.35)	2.44% (-0.32 to 6.46)	0.45% (0.22 to 0.71)	1.99% (-0.09 to 3.97)	0.02% (0.01 to 0.04)
Slovakia	23.87% (8.1 to 38.44)	16.55% (4.9 to 31.21)	-0.29% (-1.48 to 1.2)	1.79% (0.74 to 2.94)	1.76% (0.4 to 3.05)	0.94% (0.28 to 1.66)	4.53% (-4.49 to 13.11)	10.97% (-1.28 to 25.97)	0.5% (0.22 to 0.78)	2% (-0.09 to 4.37)	0.02% (0.01 to 0.04)
Slovenia	22.73% (8.13 to 36.66)	16.17% (4.71 to 30.51)	0.11% (-0.65 to 1.25)	0.8% (0.02 to 1.57)	3.26% (0.78 to 5.82)	0.98% (-0.02 to 1.95)	2.42% (-2.3 to 8.16)	7.33% (-0.65 to 19.8)	0.68% (0.3 to 1.07)	0.63% (-0.11 to 1.63)	0.02% (0 to 0.03)
Solomon Islands	27.96% (11.74 to 42.49)	8.65% (0.96 to 20.61)	-0.81% (-2.22 to 0.41)	3.7% (2.16 to 5.3)	0.09% (0.02 to 0.15)	13.78% (8.83 to 18.51)	6.32% (-6.95 to 16.12)	0.99% (-0.14 to 2.87)	0.04% (0.02 to 0.06)	0.05% (0 to 0.11)	0.04% (0.01 to 0.06)
Somalia	31.96% (13.69 to 46.97)	7.3% (0.23 to 19.58)	-1.64% (-4.5 to 0.87)	10.01% (5.91 to 13.98)	0.14% (0.04 to 0.25)	14.45% (9.47 to 18.91)	7.59% (-8.64 to 19.06)	0% (0 to 0)	0.02% (0.01 to 0.04)	5.98% (-0.37 to 11.3)	0.04% (0.01 to 0.07)

South Africa	12.05% (4.45 to 20.44)	2.2% (0 to 9.97)	-0.66% (-2.17 to 0.81)	5.08% (2.66 to 7.79)	0.19% (0.05 to 0.34)	3.68% (2.01 to 5.55)	2.27% (-2.15 to 6.12)	5.69% (-0.95 to 14.56)	0.17% (0.09 to 0.27)	0.7% (-0.02 to 1.46)	0.02% (0.01 to 0.04)
South Sudan	19.51% (6.09 to 32.32)	7.22% (0.24 to 19.77)	-0.94% (-2.58 to 0.44)	3.45% (1.98 to 5.13)	0.23% (0.06 to 0.42)	6.89% (4.2 to 9.71)	4.29% (-4.45 to 11.16)	0.2% (-0.16 to 0.95)	0.11% (0.05 to 0.19)	0.12% (0 to 0.26)	0.04% (0.01 to 0.06)
Spain	7.61% (-0.81 to 16.68)	1.83% (0.01 to 8.33)	0.63% (-0.27 to 2.21)	0.61% (-0.02 to 1.24)	1.37% (0.32 to 2.68)	0.53% (-0.13 to 1.15)	2.78% (-2.73 to 9.61)	8.76% (-1.12 to 21.21)	0.4% (0.19 to 0.67)	0.95% (-0.11 to 2.48)	0.02% (0 to 0.03)
Sri Lanka	18.77% (5.88 to 32.59)	11.77% (1.7 to 25.58)	-0.1% (-0.28 to 0.06)	2.88% (1.31 to 4.6)	0.08% (0.02 to 0.14)	2.9% (1.08 to 4.75)	2.59% (-2.42 to 7.46)	2.87% (-0.45 to 7.25)	0.09% (0.04 to 0.15)	0.73% (-0.04 to 1.68)	0.03% (0.01 to 0.05)
Sudan	15.6% (-5.78 to 31.51)	1.71% (0 to 8.18)	-1.14% (-3.24 to 0.55)	2.75% (1.51 to 3.93)	0.15% (0.04 to 0.27)	2.76% (1.65 to 3.98)	10.3% (-12.48 to 26.29)	0% (-0.01 to 0)	0.07% (0.03 to 0.12)	2.55% (-0.12 to 5.13)	0.03% (0.01 to 0.06)
Suriname	12.44% (2.34 to 23.81)	4.18% (0.02 to 14.1)	-0.52% (-1.44 to 0.27)	1.82% (0.95 to 2.82)	0.23% (0.05 to 0.41)	3.62% (1.87 to 5.48)	3.67% (-3.6 to 10.39)	4.45% (-0.63 to 11.38)	0.21% (0.1 to 0.36)	4.24% (-0.24 to 8.78)	0.01% (0 to 0.02)
Sweden	11.96% (0.72 to 24.38)	4.06% (0.03 to 13.81)	0.58% (-0.33 to 2)	0.96% (-0.08 to 1.99)	2.63% (0.59 to 5.1)	1.1% (-0.2 to 2.33)	3.28% (-3.3 to 11.28)	9.98% (-1.23 to 23.87)	0.37% (0.17 to 0.63)	0.81% (-0.19 to 2.21)	0.02% (0.01 to 0.04)
Switzerland	10.7% (-0.88 to 24.71)	3.75% (0.02 to 13.23)	1.25% (-0.7 to 3.36)	0.93% (-0.01 to 1.9)	0.99% (0.25 to 1.76)	0.98% (-0.21 to 2.06)	3.51% (-3.53 to 11.92)	11.34% (-1.39 to 26.83)	0.24% (0.11 to 0.41)	1.45% (-0.23 to 3.78)	0.02% (0.01 to 0.04)
Syrian Arab Republic	13.27% (-5.47 to 28.42)	1.7% (0 to 8.42)	-1.1% (-3.08 to 0.58)	1.8% (1.02 to 2.65)	0.23% (0.05 to 0.42)	1.93% (1.11 to 2.88)	9.14% (-10.61 to 23.36)	0.56% (-0.06 to 1.71)	0.16% (0.08 to 0.28)	1.42% (-0.07 to 2.87)	0.03% (0.01 to 0.05)
Taiwan (Province of China)	16.61% (1.63 to 31.09)	9.41% (0.87 to 22.56)	1.79% (-0.96 to 4.98)	1.11% (0.52 to 1.78)	0.46% (0.1 to 0.81)	0.59% (0.12 to 1.14)	4.26% (-4.33 to 12.08)	4.4% (-0.63 to 10.81)	0.21% (0.1 to 0.34)	1.8% (-0.09 to 3.92)	0.01% (0 to 0.01)
Tajikistan	18.94% (-3.33 to 36.37)	6.11% (0.28 to 17.65)	-1% (-2.79 to 0.53)	3.78% (2.18 to 5.39)	0.71% (0.18 to 1.27)	0.51% (0.18 to 0.95)	10.12% (-12.31 to 25.97)	1.37% (-0.17 to 3.67)	0.06% (0.02 to 0.1)	3.51% (-0.2 to 6.93)	0.03% (0.01 to 0.06)
Thailand	17.28% (5.85 to 30.36)	11.63% (1.84 to 25.21)	-0.94% (-2.65 to 0.5)	1.33% (0.73 to 2.02)	0.09% (0.02 to 0.16)	3.61% (1.97 to 5.4)	2.23% (-2.17 to 6.14)	4.81% (-0.8 to 11.99)	0.23% (0.11 to 0.36)	4.27% (-0.24 to 8.43)	0.02% (0.01 to 0.05)
Timor-Leste	26.51% (12.59 to 39.76)	12.6% (2.23 to 26.98)	-0.7% (-1.91 to 0.36)	7.74% (4.19 to 11.43)	0.05% (0.01 to 0.09)	7.66% (4.29 to 11.16)	2.62% (-2.43 to 7.33)	3.59% (-0.54 to 9.31)	0.02% (0.01 to 0.03)	1.89% (-0.09 to 4)	0.03% (0.01 to 0.06)
Togo	28.81% (13.32 to 42.1)	3.92% (0 to 14.01)	-0.51% (-1.38 to 0.26)	13.65% (7.95 to 18.84)	0.4% (0.1 to 0.72)	11.25% (7.08 to 15.11)	5.2% (-5.34 to 13.43)	4.23% (-0.74 to 10.98)	0.04% (0.02 to 0.06)	0.05% (0 to 0.11)	0.04% (0.01 to 0.07)
Tokelau	20.13% (5.93 to 34.24)	9.63% (0.99 to 22.76)	-0.99% (-2.9 to 0.55)	2.26% (1.16 to 3.55)	0.27% (0.07 to 0.47)	5.38% (2.6 to 8.28)	5.07% (-5.29 to 14.31)	2.65% (-0.34 to 7.27)	0.25% (0.12 to 0.42)	0.07% (0 to 0.18)	0.03% (0.01 to 0.05)
Tonga	20.76% (6.21 to 35.07)	9.61% (0.94 to 22.91)	-1.04% (-2.97 to 0.46)	2.37% (1.29 to 3.68)	0.19% (0.05 to 0.33)	6.1% (3.01 to 9.24)	5.29% (-5.6 to 14.51)	0.84% (-0.09 to 2.57)	0.15% (0.07 to 0.25)	0.12% (0 to 0.29)	0.03% (0.01 to 0.05)
Trinidad and Tobago	14.68% (1.07 to 28.81)	4.49% (0.02 to 15.05)	-0.7% (-2.03 to 0.35)	2.08% (1.02 to 3.29)	0.22% (0.05 to 0.39)	3.84% (1.76 to 6.03)	5.67% (-5.93 to 16.16)	5.73% (-0.79 to 14.7)	0.26% (0.12 to 0.44)	1.39% (-0.06 to 3.06)	0.01% (0 to 0.02)
Tunisia	9.92% (-5.4 to 23.27)	1.57% (0 to 8.05)	-0.79% (-2.22 to 0.43)	1.31% (0.7 to 2.05)	0.17% (0.05 to 0.3)	0.37% (0.1 to 0.77)	7.29% (-8.25 to 20.08)	0.95% (-0.11 to 2.74)	0.23% (0.11 to 0.39)	0.16% (-0.01 to 0.37)	0.02% (0.01 to 0.04)
Turkey	8.6% (-6.07 to 21.29)	1.05% (0 to 6.51)	-0.77% (-2.28 to 0.4)	0.5% (0.22 to 0.82)	0.28% (0.07 to 0.51)	0.01% (-0.01 to 0.05)	7.13% (-8.06 to 19.95)	1.34% (-0.15 to 3.85)	0.45% (0.2 to 0.74)	0.06% (-0.01 to 0.15)	0.02% (0.01 to 0.04)

Turkmenistan	22.33% (-7.62 to 42.92)	6.01% (0.25 to 17.22)	5.02% (-3 to 11.93)	2.22% (1.29 to 3.18)	1.11% (0.27 to 1.95)	0.11% (0.02 to 0.28)	10.8% (-12.89 to 27.44)	5.55% (-0.72 to 15.24)	0.44% (0.21 to 0.72)	2.32% (-0.11 to 4.57)	0.03% (0.01 to 0.06)
Tuvalu	23.73% (7 to 38.74)	9.71% (1.12 to 22.66)	-1.26% (-3.57 to 0.69)	3.2% (1.8 to 4.82)	0.1% (0.02 to 0.17)	7.89% (4.68 to 11.1)	6.54% (-7.14 to 17.2)	2.09% (-0.34 to 5.62)	0.07% (0.03 to 0.11)	0.22% (0 to 0.49)	0.03% (0.01 to 0.05)
Uganda	18.46% (6.31 to 31.15)	7.3% (0.24 to 19.4)	-0.82% (-2.37 to 0.42)	1.03% (0.59 to 1.53)	0.15% (0.04 to 0.27)	8.46% (5.07 to 12.09)	3.65% (-3.53 to 9.69)	5.81% (-1.05 to 15.21)	0.04% (0.02 to 0.07)	0.12% (0 to 0.27)	0.03% (0.01 to 0.06)
Ukraine	11.21% (-1.86 to 23.6)	2.95% (0 to 11.67)	-1.07% (-3 to 0.55)	2.64% (1.21 to 4.21)	0.63% (0.16 to 1.16)	0.43% (0.1 to 0.86)	6.05% (-6.36 to 17.34)	6.96% (-0.61 to 19.38)	0.07% (0.04 to 0.12)	0.78% (-0.03 to 1.65)	0.02% (0.01 to 0.04)
United Arab Emirates	17.89% (-7.81 to 35)	2.03% (0 to 9.18)	-2.94% (-7.87 to 1.3)	2.7% (1.62 to 3.74)	0.6% (0.15 to 1.04)	2.06% (1.27 to 2.97)	13.15% (-16.51 to 31.53)	2.7% (-0.4 to 7.52)	0.91% (0.45 to 1.43)	1.15% (-0.06 to 2.44)	0.03% (0.01 to 0.05)
United Kingdom	10.21% (0.38 to 21.6)	3.45% (0.03 to 12.14)	0% (-0.5 to 0.96)	1.46% (0.1 to 2.85)	1.35% (0.32 to 2.44)	1.08% (-0.07 to 2.19)	3.31% (-3.28 to 10.99)	10.36% (-1.41 to 24.49)	0.21% (0.1 to 0.34)	1.01% (-0.15 to 2.62)	0.02% (0 to 0.03)
United Republic of Tanzania	18.22% (6.33 to 31.41)	10.04% (0.84 to 23.8)	-0.62% (-1.7 to 0.33)	2.08% (1.16 to 3.17)	0.21% (0.05 to 0.37)	5.23% (2.92 to 7.89)	2.4% (-2.35 to 6.56)	5.89% (-1.03 to 14.73)	0.07% (0.03 to 0.11)	0.18% (-0.01 to 0.4)	0.03% (0.01 to 0.05)
United States of America	14.29% (1.64 to 26.69)	4.49% (0.09 to 14.52)	1.27% (-0.78 to 3.55)	1.41% (0.53 to 2.38)	3.25% (0.82 to 5.52)	1.27% (0.31 to 2.22)	3.43% (-3.33 to 10.24)	7.59% (-0.98 to 19.39)	0.78% (0.38 to 1.21)	1.1% (-0.06 to 2.59)	0.01% (0 to 0.01)
United States Virgin Islands	10.73% (0.27 to 22.5)	4.53% (0.02 to 15.31)	-0.75% (-2.37 to 0.4)	0.8% (0.31 to 1.35)	0.32% (0.08 to 0.58)	1.83% (0.57 to 3.15)	4.07% (-4.06 to 12.19)	6.68% (-0.73 to 19.52)	0.36% (0.17 to 0.63)	0.78% (-0.04 to 1.86)	0.01% (0 to 0.02)
Uruguay	16.47% (0.67 to 33.04)	6.97% (0.22 to 19.06)	2.04% (-1.11 to 5.77)	1.13% (0.26 to 2.07)	0.89% (0.23 to 1.59)	2.1% (0.36 to 3.75)	4.75% (-4.91 to 14.79)	7.99% (-1.15 to 19.4)	0.52% (0.25 to 0.84)	0.95% (-0.12 to 2.31)	0.01% (0 to 0.03)
Uzbekistan	18.22% (-5.28 to 35.92)	6.26% (0.28 to 17.71)	-0.7% (-2.7 to 1.91)	2.39% (1.39 to 3.44)	0.55% (0.14 to 0.99)	0.02% (-0.01 to 0.06)	10.77% (-12.99 to 27.32)	4.33% (-0.58 to 11.95)	0.12% (0.06 to 0.2)	1.45% (-0.07 to 2.89)	0.03% (0.01 to 0.06)
Vanuatu	24.08% (6.95 to 38.71)	9.46% (1.11 to 21.73)	-1.59% (-4.79 to 0.94)	2.75% (1.56 to 3.91)	0.19% (0.05 to 0.34)	7.84% (5.02 to 10.6)	7.72% (-8.77 to 19.72)	2.34% (-0.35 to 6.42)	0.13% (0.06 to 0.24)	0.09% (0 to 0.21)	0.03% (0.01 to 0.06)
Venezuela (Bolivarian Republic of)	13.55% (3.16 to 26.15)	8.27% (0.54 to 20.84)	-1.06% (-3.01 to 0.57)	1.45% (0.65 to 2.34)	0.26% (0.06 to 0.46)	2.67% (1.05 to 4.27)	2.39% (-2.19 to 6.93)	3.53% (-0.55 to 9.32)	0.19% (0.09 to 0.32)	2.03% (-0.1 to 4.45)	0.02% (0 to 0.03)
Viet Nam	16.4% (4.97 to 29.92)	12.81% (2.28 to 26.75)	-1.12% (-3.3 to 0.58)	2.02% (1.05 to 3.08)	0.05% (0.01 to 0.08)	1.25% (0.56 to 2.07)	1.91% (-1.71 to 5.39)	9.48% (-1.54 to 21.21)	0.03% (0.02 to 0.05)	4.73% (-0.27 to 9.57)	0.03% (0.01 to 0.06)
Yemen	20.21% (-2.24 to 37.34)	1.63% (0 to 8.25)	-0.82% (-2.19 to 0.44)	4.61% (2.67 to 6.52)	0.14% (0.03 to 0.26)	6.53% (4.08 to 9.12)	10.55% (-12.94 to 26.88)	0.31% (-0.03 to 0.91)	0.03% (0.02 to 0.06)	3.46% (-0.19 to 6.67)	0.03% (0.01 to 0.06)
Zambia	26.27% (13.22 to 39.39)	7.3% (0.24 to 19.46)	-0.82% (-2.3 to 0.46)	11.92% (6.91 to 17.2)	0.23% (0.05 to 0.43)	8.63% (5.24 to 12.27)	2.59% (-2.48 to 6.82)	5.27% (-0.92 to 13.37)	0.05% (0.03 to 0.09)	0.29% (-0.01 to 0.61)	0.03% (0.01 to 0.06)

Zimbabwe	26.2% (13.78 to 38.14)	3.99% (0.01 to 14.69)	-0.86% (-2.35 to 0.45)	11.2% (6.6 to 15.85)	0.12% (0.03 to 0.21)	12.45% (7.74 to 16.78)	3.28% (-3.29 to 8.69)	4.57% (-0.62 to 11.88)	0.04% (0.02 to 0.07)	0.48% (-0.02 to 0.99)	0.02% (0.01 to 0.04)

Note: Percentage data are presented to one decimal place. 95% uncertainty intervals are presented to one decimal places, and if necessary, to two decimal places. DALY=disability-adjusted life-year. Dietary risks cluster includes diet high in sodium, diet high in red meat, diet low in fruits, diet high in processed meat, diet low in vegetables, diet low in whole grains, alcohol use, diet high in sugar-sweetened beverages, diet low in fiber and diet low in polyunsaturated fatty acids.

eTable 11c. Percent of DALYs (with 95% uncertainty intervals) due to IS for metabolic risks by country and territory for both sexes combined in 2021, aged ≥20 years

	**Metabolic risks**	High body-mass index	High fasting plasma glucose	High systolic blood pressure	High LDL cholesterol	Kidney dysfunction
Afghanistan	78.9% (67.76 to 87.3)	9.55% (1.49 to 17.8)	21.53% (17.14 to 26.2)	52.4% (36.72 to 65.44)	36.51% (15.01 to 55.69)	10.65% (7.91 to 13.46)
Albania	81.37% (70.69 to 89.1)	8.01% (1.2 to 16.44)	17.52% (13.65 to 21.5)	64.54% (49.75 to 76.43)	30.38% (10.02 to 50.69)	7.89% (5.12 to 10.74)
Algeria	80.02% (69.57 to 88.32)	10.66% (1.72 to 20.27)	24.05% (19.4 to 29.31)	56.16% (40.97 to 68.83)	30.98% (10.93 to 49.54)	10.19% (7.08 to 13.41)
American Samoa	83.78% (74.84 to 90.69)	15.86% (2.76 to 27.75)	31.4% (25.81 to 37.56)	60.41% (44.48 to 72.48)	31.87% (11.87 to 50.35)	11.85% (8.94 to 14.98)
Andorra	78.77% (66.68 to 87.64)	6.1% (0.86 to 12.07)	17.9% (14 to 22.03)	57.96% (43.04 to 70.19)	32.04% (10.65 to 52.53)	8.92% (5.78 to 12.19)
Angola	78.83% (68.43 to 87.28)	4.86% (0.69 to 9.09)	14.53% (11.26 to 17.92)	60.7% (45.93 to 73.11)	27.69% (9.87 to 45.65)	12.75% (9.6 to 16.02)
Antigua and Barbuda	77.46% (66.1 to 85.71)	8.36% (1.31 to 16.34)	20.62% (16.22 to 25.24)	55.98% (40.15 to 68.11)	28.35% (9.24 to 46.98)	9.04% (6.45 to 11.9)
Argentina	79.31% (67.9 to 87.88)	9.51% (1.45 to 18.87)	20.36% (16.2 to 24.55)	58.55% (43.49 to 70.1)	31.02% (10.5 to 50.26)	7.25% (4.81 to 9.88)
Armenia	79.62% (69.12 to 88)	9.32% (1.43 to 17.85)	15.52% (12.09 to 19.06)	62.41% (46.27 to 73.8)	26.16% (8.6 to 43.69)	12.5% (8.92 to 16.14)
Australia	75.8% (63.78 to 85.56)	8.15% (1.24 to 16.41)	19.25% (15.09 to 23.52)	51.98% (37.85 to 64.24)	30.96% (9.99 to 51.56)	8.7% (5.3 to 12.45)
Austria	77.78% (66.12 to 87.22)	6.15% (0.88 to 12.53)	13.22% (10.29 to 16.51)	58.22% (42.98 to 70.47)	32.37% (10.7 to 53.57)	8.93% (5.77 to 12.28)
Azerbaijan	82.21% (71.58 to 89.77)	11.27% (1.77 to 21.33)	16.29% (12.69 to 20.14)	62.49% (46.66 to 73.96)	32.99% (12.13 to 51.68)	13.23% (9.99 to 16.47)
Bahamas	78.65% (67.94 to 87.22)	10.98% (1.71 to 20.27)	20.66% (16.39 to 25.42)	56.27% (41.35 to 69.35)	31.37% (11 to 50.35)	8.77% (6.31 to 11.47)
Bahrain	84.4% (74.08 to 91.46)	16.96% (2.91 to 30.33)	27.3% (22.28 to 32.37)	58.05% (44.1 to 70.02)	40.47% (16.91 to 60.06)	10.97% (8.11 to 13.91)
Bangladesh	76.34% (65.46 to 84.7)	2.09% (0.27 to 3.96)	18.9% (14.93 to 23.18)	58.81% (44.22 to 71)	21.39% (6.85 to 37.53)	10.21% (7.08 to 13.49)
Barbados	80.79% (70.67 to 88.91)	8.78% (1.36 to 17.08)	25.41% (20.19 to 30.62)	60.43% (44.97 to 72.29)	27.96% (9.15 to 46.8)	8.58% (5.87 to 11.4)
Belarus	81.8% (70.95 to 89.56)	10.36% (1.6 to 19.9)	11.13% (8.47 to 13.93)	66.32% (50.75 to 78.17)	30.92% (10.37 to 50.17)	11.5% (8.17 to 14.98)
Belgium	79.19% (68.17 to 87.71)	5.93% (0.85 to 11.81)	17.29% (13.43 to 21.11)	60.81% (45.17 to 72.94)	29.29% (9.39 to 49.43)	8.87% (5.74 to 12.11)
Belize	76.73% (65.45 to 85.92)	11.56% (1.88 to 21.22)	17.5% (13.81 to 21.59)	54.31% (38.91 to 67.25)	30.16% (10.55 to 48.5)	9.04% (6.52 to 11.57)
Benin	78.17% (66.66 to 86.77)	6.11% (0.87 to 11.55)	13.52% (10.5 to 16.77)	61.64% (45.6 to 73.51)	26.27% (8.96 to 43.53)	10.9% (8.05 to 13.97)
Bermuda	76.77% (64.95 to 85.55)	9.5% (1.48 to 18.67)	21.39% (17.03 to 26.07)	54.84% (39.91 to 67.64)	27.65% (8.99 to 46.34)	8.06% (5.34 to 10.9)
Bhutan	77.88% (67.08 to 86.5)	5.5% (0.8 to 10.85)	17.36% (13.6 to 21.25)	55.28% (39.55 to 67.42)	32.38% (11.33 to 51.83)	11.24% (7.92 to 14.5)

Bolivia (Plurinational State of)	72.91% (61.15 to 83.05)	8.12% (1.24 to 15.55)	17.93% (13.96 to 22.02)	46.98% (33.31 to 60.28)	30.6% (10.75 to 49.5)	8.06% (5.69 to 10.5)
Bosnia and Herzegovina	82.8% (72.73 to 90.43)	8.2% (1.23 to 16.03)	22.6% (17.83 to 27.94)	64.3% (48.31 to 77.03)	32.06% (10.96 to 52.06)	8.19% (5.5 to 10.92)
Botswana	79.52% (69.02 to 87.39)	8.68% (1.29 to 16.26)	15.55% (12.17 to 19.33)	63.21% (47.45 to 75.29)	23.69% (7.75 to 40.2)	13% (9.79 to 16.35)
Brazil	80.01% (68.87 to 88.11)	8.89% (1.32 to 16.94)	19.64% (15.46 to 24.16)	57.73% (43.07 to 69.32)	33.78% (11.88 to 53.36)	10.17% (7.41 to 13)
Brunei Darussalam	81.21% (70.54 to 88.82)	7.33% (1.11 to 13.26)	23.61% (18.84 to 29.14)	56.01% (40.67 to 68.71)	36.2% (13.86 to 55.8)	11.75% (8.34 to 15.26)
Bulgaria	81.89% (72.07 to 89.94)	8.72% (1.33 to 17.16)	21.17% (16.76 to 25.8)	63.46% (48.55 to 75.74)	31.25% (10.39 to 51.24)	8.16% (5.48 to 10.92)
Burkina Faso	72.92% (60.53 to 82.59)	2.6% (0.33 to 4.94)	12.91% (9.89 to 16.15)	56.84% (40.77 to 69.52)	19.15% (6.06 to 33.69)	10.57% (7.88 to 13.4)
Burundi	74.78% (62.71 to 84.24)	2.24% (0.3 to 4.25)	10.44% (7.86 to 13.07)	59.21% (43.5 to 71.12)	24.77% (8.48 to 41.36)	8.58% (6.01 to 11.18)
Cabo Verde	81.28% (71.48 to 88.96)	6.3% (0.96 to 12.08)	18.98% (15.1 to 23.24)	64.76% (49.26 to 76.34)	27.42% (9.65 to 44.95)	10.69% (7.85 to 13.74)
Cambodia	72.94% (61.26 to 83.49)	2.1% (0.29 to 4.04)	13.33% (10.21 to 16.93)	48.98% (34.66 to 62.21)	29.24% (10.12 to 47.45)	12.16% (8.94 to 15.55)
Cameroon	80.14% (69.76 to 87.87)	10.47% (1.61 to 20.31)	16.39% (12.53 to 20.08)	65.31% (50.31 to 76.38)	19.52% (6.21 to 34.21)	13.85% (10.82 to 16.97)
Canada	74.27% (62.42 to 83.99)	8.24% (1.25 to 16.41)	16.49% (13.08 to 20.36)	50.85% (36.88 to 62.94)	29.54% (9.75 to 49.16)	8.72% (5.57 to 11.94)
Central African Republic	78.92% (67.73 to 87.88)	4.6% (0.66 to 8.69)	17.47% (13.55 to 21.6)	59.17% (43.18 to 73.17)	27.98% (10.01 to 46)	13.24% (10.29 to 16.46)
Chad	75.8% (64.63 to 84.5)	3.74% (0.51 to 7.09)	15.25% (11.7 to 19.13)	56.36% (41.21 to 68.91)	26.92% (9.68 to 44.17)	10.74% (8.15 to 13.61)
Chile	82.18% (72.1 to 89.7)	9.78% (1.48 to 18.84)	20.02% (15.62 to 24.46)	64.19% (49.37 to 75.5)	29.94% (10.14 to 49.44)	7.75% (4.94 to 10.54)
China	77.22% (65.42 to 86)	5.08% (0.74 to 9.75)	16.79% (13.18 to 20.67)	57.71% (42.91 to 69.88)	29.3% (9.93 to 47.71)	8.02% (5.59 to 10.54)
Colombia	81.62% (71.67 to 89.63)	8.61% (1.29 to 16.74)	17.65% (13.88 to 21.74)	62.11% (47.47 to 73.46)	31.69% (11.12 to 51.05)	11.36% (8.02 to 14.63)
Comoros	77.26% (66.36 to 85.94)	5.54% (0.78 to 10.29)	11.36% (8.82 to 14.2)	60.41% (44.68 to 72.7)	28.91% (10.06 to 47.09)	8.39% (5.74 to 11.27)
Congo	80.5% (69.51 to 88.23)	7.48% (1.12 to 14.23)	15.44% (12.1 to 19.21)	62.4% (46.73 to 73.99)	28.64% (10.49 to 46.7)	13.06% (9.9 to 16.42)
Cook Islands	82.72% (73.3 to 89.82)	13.13% (2.24 to 23.41)	32.94% (27.26 to 38.88)	57.99% (43.71 to 70.66)	30.65% (10.8 to 49.61)	10.23% (7.42 to 13.28)
Costa Rica	81.8% (71.95 to 89.46)	8.49% (1.3 to 16.48)	20.16% (15.96 to 24.88)	61.37% (45.92 to 73.21)	31.14% (10.6 to 51.01)	13.56% (9.41 to 17.73)
Coted'Ivoire	80.64% (70.34 to 88.72)	7.44% (1.12 to 14.02)	14.17% (10.95 to 17.64)	64.12% (50.27 to 75.64)	29.47% (11.05 to 47.7)	11.15% (8.4 to 14.09)
Croatia	81.77% (71.56 to 89.69)	8.16% (1.2 to 16.24)	22.43% (17.64 to 27.2)	63.48% (48.76 to 75.57)	29.58% (9.51 to 49.72)	8.02% (5.07 to 10.96)
Cuba	74.45% (62 to 83.98)	7.2% (1.07 to 13.71)	21.26% (16.87 to 25.88)	50.35% (35.32 to 62.66)	28.12% (9.25 to 46.69)	8.27% (5.73 to 10.87)
Cyprus	78.69% (67.57 to 87.94)	5.76% (0.84 to 11.17)	19.31% (15.11 to 23.56)	58.22% (42.48 to 71.19)	30.23% (9.72 to 50.91)	8.97% (5.56 to 12.47)
Czechia	81.88% (71.5 to 89.88)	9.06% (1.39 to 18.14)	24.21% (19.29 to 29.35)	61.07% (45.11 to 73.49)	32.54% (10.81 to 53.24)	7.96% (5.15 to 10.72)

Democratic People's Republic of Korea	77.77% (65.5 to 87.35)	3.17% (0.42 to 6.61)	16.2% (12.69 to 19.8)	54.74% (39.75 to 67.4)	35.11% (13.28 to 54.88)	8.63% (6.52 to 10.9)
Democratic Republic of the Congo	77.83% (66.95 to 86.35)	5.55% (0.75 to 10.61)	18.45% (14.56 to 22.8)	57.34% (42.5 to 69.79)	26.58% (9.48 to 43.6)	13.05% (9.85 to 16.4)
Denmark	79.1% (68.28 to 88.01)	5.46% (0.78 to 10.86)	14.47% (11.23 to 17.96)	60.72% (46.27 to 72.98)	31.38% (10.03 to 52.46)	8.91% (5.77 to 12.13)
Djibouti	76.73% (65.23 to 85.52)	2.62% (0.34 to 4.92)	11.27% (8.74 to 14.19)	60.84% (45.51 to 73.25)	26.73% (9.57 to 44.08)	8.48% (6.01 to 10.99)
Dominica	77.63% (67.58 to 86.58)	11% (1.77 to 20.87)	23.64% (18.95 to 29.04)	55.88% (41.05 to 68.46)	25.85% (8.48 to 43.1)	9.23% (6.44 to 12.17)
Dominican Republic	76.51% (65.33 to 85.83)	8.01% (1.25 to 15.08)	15.78% (12.41 to 19.58)	54.72% (38.96 to 67.57)	31.71% (11.63 to 50.53)	8.8% (6.31 to 11.36)
Ecuador	73.55% (61.21 to 83.55)	10.17% (1.63 to 18.83)	21.25% (17.09 to 25.89)	46.37% (32.02 to 59.05)	30.35% (10.55 to 49.07)	7.96% (5.61 to 10.47)
Egypt	82.34% (71.72 to 90.14)	17.62% (3.07 to 31.81)	19.22% (15.06 to 23.41)	60.92% (44.67 to 72.29)	35.14% (13.69 to 54.45)	11.45% (8.56 to 14.6)
El Salvador	79.13% (68.42 to 87.82)	10.06% (1.58 to 19.4)	18.53% (14.39 to 22.92)	56.92% (41.79 to 69.59)	32.14% (11.33 to 51.76)	10.87% (7.84 to 13.84)
Equatorial Guinea	81.88% (72.91 to 89.26)	8.76% (1.29 to 16.69)	22.45% (17.99 to 26.97)	62.54% (46.76 to 74.19)	28.58% (10.65 to 46.51)	12.78% (9.49 to 16.16)
Eritrea	72.62% (58.78 to 83.42)	2.47% (0.33 to 4.66)	9.66% (7.32 to 12.14)	54.59% (39.2 to 67.92)	26.54% (9.16 to 43.72)	8.39% (5.98 to 10.94)
Estonia	82.9% (72.6 to 90.64)	8.97% (1.33 to 17.7)	16.02% (12.57 to 19.77)	64.38% (48.18 to 76.2)	35.09% (12.06 to 56.11)	11.47% (7.86 to 15.22)
Eswatini	80.8% (70.86 to 88.72)	13.41% (2.12 to 24.88)	17.72% (13.83 to 21.99)	64.09% (48.52 to 76.26)	23.45% (7.86 to 39.98)	13.4% (10.24 to 16.67)
Ethiopia	66.21% (53.36 to 76.44)	2.58% (0.36 to 4.98)	11.02% (8.46 to 13.96)	47.76% (34.38 to 59.66)	21.48% (6.97 to 37.15)	8.02% (5.49 to 10.54)
Fiji	85.46% (77.04 to 91.61)	14.63% (2.45 to 26.2)	30.59% (24.92 to 36.08)	63.08% (47.24 to 75.15)	35.79% (13.92 to 54.7)	11.53% (8.72 to 14.45)
Finland	79.41% (68.28 to 88.29)	6.52% (0.94 to 13.14)	20.55% (16.38 to 25)	59.65% (44.82 to 71.84)	29.79% (9.4 to 50.29)	8.34% (5.32 to 11.48)
France	76.95% (65.04 to 86.56)	5.78% (0.83 to 11.56)	14.23% (10.98 to 17.78)	57.23% (42.72 to 69.34)	31.26% (9.9 to 52.07)	8.08% (4.95 to 11.33)
Gabon	81.16% (71.39 to 89.12)	9.91% (1.52 to 18.54)	21.7% (17.43 to 26.46)	61.58% (46.59 to 74.18)	27.23% (9.45 to 45.06)	12.89% (9.59 to 16.21)
Gambia	80.39% (69.48 to 88.17)	6.24% (0.9 to 11.79)	14.69% (11.41 to 18.34)	64.18% (48.52 to 75.46)	28.5% (10.22 to 46.5)	10.85% (8.09 to 13.78)
Georgia	80.92% (70.66 to 89.29)	7.42% (1.08 to 14.41)	15.08% (11.62 to 18.88)	65.1% (49.52 to 77.13)	25.92% (8.32 to 44.15)	12.54% (8.73 to 16.44)
Germany	81.52% (71.22 to 89.76)	6.91% (1 to 13.86)	18.67% (14.53 to 23.19)	62.58% (47.56 to 74.7)	32.49% (10.54 to 53.4)	8.67% (5.42 to 12)
Ghana	81.56% (70.99 to 89.62)	7.07% (1.03 to 13.27)	17.31% (13.66 to 21.04)	62.13% (47.81 to 73.37)	34.75% (13.69 to 53.61)	8.77% (6.33 to 11.49)
Greece	73.37% (61.36 to 83.35)	5.92% (0.86 to 12.54)	18.18% (14.35 to 22.59)	50.79% (36.27 to 63.5)	26.93% (8.04 to 46.53)	8.9% (5.23 to 12.75)
Greenland	74.28% (60.31 to 85.18)	9.84% (1.49 to 18.93)	12.99% (10.22 to 16.39)	50.08% (35.82 to 62.9)	32.55% (11.62 to 51.59)	9.6% (6.72 to 12.52)
Grenada	78.15% (66.24 to 86.6)	7.9% (1.21 to 15.2)	20.9% (16.37 to 25.62)	56.66% (41.55 to 69.42)	29.11% (9.94 to 47.6)	9.54% (6.81 to 12.54)
Guam	81.59% (71.36 to 89.22)	12.25% (2.03 to 23.01)	24.63% (19.95 to 29.82)	57.65% (41.22 to 69.53)	34.38% (12.99 to 53.05)	10.87% (8.18 to 13.64)

Guatemala	76.14% (65.52 to 84.72)	9.39% (1.45 to 17.78)	18.68% (14.75 to 23.25)	53.75% (38.96 to 65.6)	28.29% (9.82 to 46.09)	12.26% (8.79 to 15.87)
Guinea	76.3% (64.7 to 85.07)	4.49% (0.64 to 8.34)	13.83% (10.74 to 16.85)	57.62% (43.26 to 69.96)	27.77% (9.7 to 45.44)	10.96% (8.25 to 13.86)
Guinea-Bissau	79.04% (68.18 to 87.17)	5.79% (0.87 to 10.57)	13.79% (10.62 to 17.24)	60.99% (45.06 to 73.15)	29.72% (11.27 to 47.35)	11.34% (8.74 to 14.18)
Guyana	80.3% (70.83 to 88.1)	8.49% (1.3 to 16.39)	27.45% (21.95 to 32.94)	56.32% (40.32 to 68.89)	30.97% (11.05 to 49.39)	9.53% (7.05 to 12.19)
Haiti	79.86% (69.88 to 87.89)	4.31% (0.62 to 8.27)	17.45% (13.68 to 21.49)	61.83% (47.39 to 74.04)	30.66% (11.01 to 48.98)	9.46% (6.97 to 12.19)
Honduras	79.59% (68.32 to 87.99)	8.53% (1.33 to 16.72)	18.21% (14.03 to 22.83)	58.06% (41.85 to 70.8)	30.9% (10.59 to 49.49)	12.69% (9.35 to 16.14)
Hungary	85.78% (76.39 to 92.38)	10.23% (1.54 to 19.25)	20.19% (15.78 to 24.91)	69.74% (54.63 to 80.29)	34.81% (12.02 to 55.27)	8.05% (5.43 to 10.79)
Iceland	77.39% (66.33 to 87.04)	6.92% (0.99 to 14.02)	18.09% (14.32 to 22.11)	55.93% (41.48 to 68.57)	31.85% (10.41 to 52.78)	7.15% (4.55 to 9.94)
India	77.64% (67.12 to 86.15)	3.12% (0.41 to 5.88)	19.44% (15.5 to 24.14)	56.91% (42.04 to 68.42)	27.38% (9.56 to 45.29)	11.67% (8.47 to 15.04)
Indonesia	83.63% (73.88 to 90.55)	3.55% (0.48 to 6.81)	15.03% (11.76 to 18.63)	69.34% (54.08 to 79.55)	27.14% (9.66 to 44.08)	13.46% (10.02 to 17.01)
Iran (Islamic Republic of)	79.4% (68.74 to 87.7)	11.79% (1.88 to 22.01)	18.9% (14.96 to 23.17)	55.13% (41.21 to 66.28)	35.23% (13.44 to 54.14)	11.06% (7.97 to 14.22)
Iraq	85.97% (77.01 to 92.04)	13.44% (2.14 to 25.45)	26.97% (21.44 to 32.51)	66.1% (50.46 to 77.12)	35.16% (13.51 to 54.12)	11.54% (8.32 to 14.91)
Ireland	78.65% (67.28 to 87.2)	6.61% (0.95 to 13.4)	17.47% (13.65 to 21.52)	59% (44.09 to 70.67)	27.78% (8.87 to 47.53)	11.42% (7.65 to 15.21)
Israel	80.08% (69.53 to 88.38)	6.94% (1.01 to 13.84)	20.91% (16.69 to 25.65)	60.53% (45.35 to 71.98)	28.95% (9.52 to 48.03)	9.25% (6.08 to 12.62)
Italy	72.73% (60.53 to 82.72)	5.28% (0.74 to 10.99)	19.1% (15.18 to 23.59)	48.78% (33.98 to 60.6)	28.21% (8.58 to 48.33)	7.87% (4.77 to 11.09)
Jamaica	73.98% (62.2 to 83.88)	7.96% (1.24 to 15.09)	14.83% (11.48 to 18.54)	54.47% (39.88 to 66.81)	26.15% (8.53 to 44.33)	8.67% (5.85 to 11.45)
Japan	76.31% (66.53 to 85.87)	2.4% (0.32 to 4.76)	21.26% (17.22 to 25.87)	53.96% (39.33 to 66.68)	27.42% (8.59 to 46.62)	10.58% (6.14 to 14.99)
Jordan	83.19% (72.85 to 90.78)	17.2% (2.97 to 30.53)	26.43% (21.19 to 31.54)	56.94% (40.88 to 69.04)	38.62% (15.45 to 58.23)	10.74% (7.76 to 13.75)
Kazakhstan	84.67% (75.43 to 91.48)	10.18% (1.56 to 19.26)	16.29% (12.84 to 20.12)	68.55% (53.03 to 79.38)	31.5% (11.36 to 50.21)	13.05% (9.63 to 16.41)
Kenya	74.81% (63.89 to 83.85)	5.53% (0.79 to 10.47)	11.52% (8.92 to 14.57)	59.14% (44.79 to 70.61)	23.42% (7.92 to 39.63)	8.55% (5.93 to 11.1)
Kiribati	79.52% (68.61 to 87.99)	15.47% (2.63 to 27.92)	27.4% (22.39 to 32.7)	48.89% (33.53 to 60.57)	35.1% (14.09 to 54.68)	11.31% (8.86 to 13.92)
Kuwait	83.13% (72.29 to 91)	20.76% (3.71 to 35.74)	23.12% (18.41 to 27.81)	54.14% (39.37 to 66.2)	42.66% (18.77 to 61.96)	10.06% (7.31 to 12.83)
Kyrgyzstan	79.35% (66.98 to 88.43)	12.58% (1.98 to 23.87)	12.08% (9.21 to 15.05)	56.01% (39.4 to 69.59)	35.61% (14 to 55.04)	12.7% (10.01 to 15.59)
Lao People's Democratic Republic	78.66% (67.35 to 87.4)	3.39% (0.44 to 6.51)	15.52% (12.06 to 19.42)	57.36% (41.9 to 69.63)	31.73% (11.63 to 50.22)	13.3% (9.98 to 16.73)
Latvia	82.18% (70.89 to 90.41)	8.77% (1.32 to 17.68)	15.75% (12.18 to 19.56)	64.86% (49.32 to 76.93)	31.89% (10.49 to 52.7)	11.42% (7.59 to 15.39)
Lebanon	80.21% (69.67 to 88.12)	11.58% (1.83 to 22.08)	24.31% (19.56 to 29.47)	55.75% (40.84 to 67.37)	34.44% (12.4 to 54.17)	10.11% (6.86 to 13.53)

Lesotho	75.56% (63.67 to 84.83)	9.17% (1.46 to 17.48)	14.45% (11.19 to 18.21)	58.45% (43.21 to 71.72)	19.88% (6.3 to 35.27)	13.26% (9.92 to 16.59)
Liberia	80.45% (70.67 to 88.21)	10.01% (1.55 to 18.57)	16.17% (12.65 to 20.11)	63% (47.67 to 74.67)	28.93% (10.38 to 46.65)	10.94% (8.23 to 13.83)
Libya	83.44% (73.28 to 90.75)	17.6% (3.01 to 31.04)	18.28% (14.42 to 22.37)	63.45% (47.76 to 74.96)	36.69% (15.03 to 55.63)	10.94% (8 to 13.88)
Lithuania	82.79% (72.05 to 90.77)	9.23% (1.39 to 17.93)	14.18% (11.08 to 17.7)	65.44% (49.87 to 76.85)	34.41% (11.6 to 55.45)	11.45% (7.83 to 15.01)
Luxembourg	79.26% (68.65 to 87.52)	6.61% (0.95 to 13.34)	20.27% (16 to 24.69)	58.78% (43.27 to 71.64)	30.42% (9.9 to 51.11)	8.98% (5.81 to 12.4)
Madagascar	74.29% (61.19 to 83.38)	3.87% (0.51 to 7.05)	9.79% (7.54 to 12.53)	57.49% (42.23 to 69.34)	26.88% (9.75 to 44.18)	8.34% (5.87 to 10.78)
Malawi	78.32% (66.82 to 86.73)	4.04% (0.57 to 7.67)	10.02% (7.58 to 12.85)	63.26% (46.99 to 75.02)	27.97% (9.9 to 45.78)	8.82% (6.18 to 11.61)
Malaysia	87.22% (78.44 to 93.11)	6.97% (1.02 to 12.89)	22.47% (17.98 to 27.38)	68.75% (52.9 to 79.45)	38.76% (15.05 to 58.71)	14.11% (10.57 to 17.76)
Maldives	81.03% (70.73 to 89.08)	5.7% (0.87 to 10.62)	16.36% (12.93 to 20.04)	59.62% (44.38 to 71.59)	35.68% (13.87 to 54.87)	12.1% (8.79 to 15.57)
Mali	76.45% (64.98 to 84.96)	3.89% (0.51 to 7.3)	17.15% (13.59 to 21.12)	56.14% (41.36 to 68.67)	26.98% (9.62 to 44.39)	10.75% (8.1 to 13.52)
Malta	78.46% (67.13 to 87.97)	5.97% (0.86 to 12.14)	19.26% (15.11 to 23.83)	57.53% (42.16 to 70.41)	30.83% (9.87 to 51.7)	8.86% (5.63 to 12.13)
Marshall Islands	81.96% (71.61 to 89.69)	16.52% (2.87 to 29.49)	33.09% (26.97 to 39.11)	48.3% (33.48 to 61.12)	37.83% (15.49 to 57.45)	11.54% (9.04 to 14.25)
Mauritania	79.3% (68.69 to 88.01)	9.18% (1.37 to 17.59)	13.13% (10.14 to 16.21)	62.21% (46.07 to 74.05)	28.14% (9.99 to 45.73)	10.61% (7.78 to 13.53)
Mauritius	83.87% (74.62 to 90.64)	7.24% (1.08 to 13.69)	23.76% (18.75 to 29.41)	62.07% (46.62 to 74.24)	34.74% (12.95 to 54.04)	14.39% (10.85 to 18.03)
Mexico	79.98% (69.05 to 88.12)	11.24% (1.8 to 20.92)	22.54% (18.08 to 27.52)	55.28% (40.21 to 66.92)	33.5% (12.25 to 52.55)	11.82% (8.62 to 15.09)
Micronesia (Federated States of)	77.4% (66.26 to 86.2)	16.38% (2.73 to 28.93)	22.93% (18.36 to 27.89)	46.4% (32.14 to 59.21)	36.21% (14.42 to 55.73)	11.67% (9.16 to 14.34)
Monaco	78.12% (67.02 to 86.91)	6.76% (0.98 to 13.52)	18.58% (14.7 to 22.96)	57.21% (43.07 to 69.36)	30.5% (9.56 to 51.19)	8.93% (5.64 to 12.5)
Mongolia	83.28% (72.31 to 90.58)	9.87% (1.49 to 18.86)	10.78% (8.3 to 13.62)	64.15% (49.31 to 76.38)	39.92% (16.71 to 59.13)	13.01% (10.14 to 15.89)
Montenegro	82.81% (71.89 to 90.47)	9.94% (1.54 to 19.24)	23.55% (18.72 to 28.3)	63.89% (48.52 to 75.56)	31.47% (10.67 to 51.78)	8.26% (5.41 to 11.15)
Morocco	84.76% (75.67 to 91.57)	10.16% (1.6 to 19.1)	26.12% (20.82 to 31.41)	64.77% (48.75 to 76.24)	34.46% (12.76 to 53.72)	10.01% (7.03 to 13.11)
Mozambique	79.32% (68.45 to 87.58)	4.3% (0.56 to 8.23)	10.32% (7.81 to 12.89)	64.38% (48.82 to 75.96)	29.85% (10.77 to 47.88)	8.99% (6.36 to 11.84)
Myanmar	80.14% (70.88 to 87.8)	2.79% (0.39 to 5.28)	18.25% (14.3 to 22.41)	60.19% (45.55 to 72.27)	30.3% (10.58 to 49.17)	12.76% (9.46 to 16.25)
Namibia	77.43% (67 to 86.12)	8.19% (1.21 to 15.64)	16.42% (12.98 to 20.47)	58.94% (42.81 to 72.24)	24.09% (7.7 to 41.04)	12.52% (9.26 to 15.93)
Nauru	84.86% (74.78 to 91.42)	19.83% (3.71 to 33.57)	23.19% (18.51 to 28.2)	62.74% (47.08 to 74.04)	38.22% (15.4 to 58.03)	11.87% (9.15 to 14.58)
Nepal	70.93% (59.68 to 80.61)	2.63% (0.39 to 4.88)	19.28% (15.24 to 23.92)	43.89% (29.17 to 56.89)	25.85% (8.67 to 43.47)	13.95% (10.25 to 17.86)
Netherlands	78.04% (66.87 to 87.92)	5.73% (0.82 to 11.35)	15.77% (12.4 to 19.58)	57.94% (42.44 to 69.75)	31.41% (9.95 to 52.4)	9.11% (6.04 to 12.54)
New Zealand	76.76% (64.34 to 85.79)	7.49% (1.11 to 14.98)	20.4% (16.05 to 25.27)	53.44% (38.96 to 65.24)	30.21% (9.67 to 50.22)	9.03% (5.56 to 12.59)

Nicaragua	81% (70.02 to 88.86)	11.08% (1.76 to 20.98)	17.4% (13.7 to 21.44)	59.78% (44.06 to 71.83)	32.18% (11.77 to 51.11)	14.85% (11.03 to 18.51)
Niger	76.83% (66 to 85.34)	3.41% (0.44 to 6.44)	14.61% (11.41 to 18.17)	59.49% (43.92 to 70.98)	25.77% (8.94 to 43.16)	10.27% (7.66 to 13.11)
Nigeria	80.96% (71.33 to 88.93)	6.88% (1.01 to 12.99)	12.87% (9.98 to 16.07)	64.4% (48.61 to 75.67)	29.14% (10.6 to 47.12)	13.36% (10.11 to 16.67)
Niue	83.28% (74.84 to 90.26)	11.81% (1.96 to 21.79)	30.95% (25.47 to 36.92)	61.66% (46.07 to 73.66)	27.91% (9.38 to 45.74)	11.16% (8.08 to 14.33)
North Macedonia	82.97% (72.31 to 90.21)	8.81% (1.32 to 17.06)	23.16% (18.2 to 28.11)	63.91% (48.12 to 76.5)	31.35% (10.49 to 51.49)	9.88% (6.7 to 13.29)
Northern Mariana Islands	82.87% (73.17 to 90.21)	15.8% (2.74 to 28.3)	25.93% (20.93 to 31.4)	58.91% (43.26 to 71.65)	34.49% (13.17 to 53.61)	11.85% (9.08 to 14.7)
Norway	78.52% (68.1 to 87.22)	5.13% (0.74 to 10.05)	19.25% (14.99 to 23.54)	57.68% (42.54 to 68.92)	31.33% (10.12 to 52.1)	8.32% (5.29 to 11.49)
Oman	84.32% (73.84 to 91.48)	17.42% (3 to 31.58)	20.88% (16.57 to 25.27)	60.66% (44.69 to 72.72)	42.04% (18.33 to 61.6)	10.62% (7.83 to 13.59)
Pakistan	80.67% (70.11 to 88.24)	5.92% (0.88 to 11.16)	21.25% (16.99 to 25.86)	60.64% (44.6 to 72.75)	30.68% (11.4 to 49.31)	11.02% (8.11 to 14.02)
Palau	84.12% (75.03 to 90.68)	15.76% (2.6 to 28.66)	30.23% (24.36 to 36.24)	58.89% (42.49 to 70.92)	35.2% (13.84 to 54.4)	11.79% (9.03 to 14.67)
Palestine	78.38% (66.3 to 87.07)	14.51% (2.46 to 26.43)	20.35% (16.02 to 24.96)	52.22% (36.65 to 65.2)	34.64% (13.25 to 54.31)	10.69% (7.64 to 14.01)
Panama	80.17% (69.99 to 88.07)	10.1% (1.55 to 19.48)	19.62% (15.71 to 24.08)	59.78% (44.07 to 71.05)	29.29% (9.83 to 48.62)	11.95% (8.33 to 15.49)
Papua New Guinea	70.83% (57.58 to 80.76)	6.27% (0.92 to 11.91)	18.32% (14.43 to 22.7)	41.51% (27.73 to 53.95)	32.28% (12.38 to 51.16)	10.11% (7.63 to 12.74)
Paraguay	79.95% (68.27 to 88.18)	8.62% (1.31 to 16.88)	19.56% (15.51 to 23.93)	58.6% (42.57 to 70.86)	32.36% (10.98 to 52.1)	10.13% (7.3 to 12.98)
Peru	75.28% (62.95 to 84.73)	8.33% (1.31 to 16.26)	14.41% (11.23 to 17.97)	53.52% (39 to 65.46)	30.8% (10.8 to 49.73)	7.26% (5.03 to 9.66)
Philippines	76.53% (64.5 to 85.74)	5.08% (0.69 to 9.53)	13.42% (10.41 to 16.9)	51.35% (37.1 to 62.73)	35.18% (13.79 to 54.29)	13.44% (10.21 to 16.77)
Poland	78.4% (68.11 to 86.82)	8.42% (1.25 to 16.75)	22.87% (18.32 to 27.69)	55.51% (40.65 to 67.27)	30.63% (10.19 to 50.33)	8.69% (5.77 to 11.71)
Portugal	75.82% (63.65 to 85.04)	5.69% (0.8 to 11.68)	22.59% (17.85 to 27.29)	52.17% (38.28 to 63.99)	29.54% (9.02 to 50.02)	7.23% (4.6 to 10.06)
Puerto Rico	79.25% (68.57 to 87.74)	9.22% (1.44 to 17.6)	27.05% (21.55 to 32.53)	55.99% (40.49 to 68.64)	28.84% (9.49 to 48.35)	8.76% (5.89 to 11.59)
Qatar	83.58% (71.85 to 91.25)	22.99% (4.23 to 39.55)	24.49% (19.83 to 29.28)	54.01% (38.87 to 65.73)	42.86% (19.08 to 62.22)	10.36% (7.82 to 12.88)
Republic of Korea	71.76% (59.68 to 81.58)	3.3% (0.44 to 6.37)	20.21% (15.96 to 24.7)	45.55% (30.91 to 57.62)	28.53% (9.44 to 47.02)	8.8% (5.44 to 12.22)
Republic of Moldova	84.66% (74.4 to 91.58)	11.83% (1.86 to 22.21)	15.86% (12.39 to 19.47)	69.67% (53.55 to 81.14)	29.72% (10.07 to 48.59)	13.72% (10.16 to 17.56)
Romania	83.35% (73.19 to 90.82)	8.08% (1.18 to 15.67)	20.28% (15.91 to 24.86)	66.53% (51.31 to 77.84)	31.55% (10.26 to 51.97)	8.19% (5.23 to 11.3)
Russian Federation	81.91% (70.72 to 89.89)	10.11% (1.53 to 19.38)	15.15% (11.86 to 18.96)	62.65% (47.94 to 73.5)	35.31% (12.46 to 55.51)	10.58% (7.36 to 13.95)
Rwanda	70.36% (58.17 to 81.44)	3.2% (0.44 to 6.17)	9.25% (7.06 to 11.58)	55.66% (39.78 to 68.61)	19.37% (6.05 to 34.31)	8.28% (5.73 to 10.96)
Saint Kitts and Nevis	79.42% (69.23 to 87.89)	9.92% (1.55 to 18.71)	21.27% (16.95 to 26.09)	58.4% (44.11 to 71.09)	30.67% (10.53 to 49.23)	9.29% (6.75 to 12.04)
Saint Lucia	76.52% (65.3 to 85.37)	6.76% (1.04 to 13.34)	21.81% (17.03 to 26.75)	56.25% (40.63 to 68.7)	24.52% (7.86 to 41.92)	8.87% (6.08 to 11.65)

Saint Vincent and the Grenadines	75.47% (64.54 to 84.14)	6.22% (0.91 to 12.23)	21.17% (16.79 to 25.88)	54.15% (39.21 to 66.64)	25.47% (8.28 to 43.42)	8.9% (6.25 to 11.62)
Samoa	79.42% (69.32 to 87.16)	15% (2.62 to 26.56)	26.63% (21.18 to 31.99)	54.8% (39.21 to 67.48)	28.6% (10.26 to 46.2)	10.96% (8.19 to 13.81)
San Marino	78.19% (66.44 to 86.92)	6.56% (0.94 to 13.27)	18.55% (14.56 to 22.6)	57.41% (43.18 to 69.63)	30.62% (9.61 to 51.61)	8.81% (5.62 to 12.42)
Sao Tome and Principe	81.72% (72.12 to 88.86)	8.32% (1.28 to 16.2)	14.07% (11.07 to 17.34)	66.3% (51.33 to 77.93)	29.12% (10.64 to 46.85)	11.7% (8.82 to 14.72)
Saudi Arabia	82.32% (71.09 to 90.66)	21.77% (3.89 to 37.47)	21.08% (16.81 to 25.74)	53.2% (38.08 to 65.99)	42.06% (17.99 to 61.42)	11.37% (8.79 to 14.06)
Senegal	80.89% (70.76 to 88.84)	5.51% (0.79 to 10.4)	18.95% (15.02 to 23.12)	64.46% (49.51 to 76.02)	26.38% (9.08 to 43.37)	9.65% (7 to 12.43)
Serbia	83.82% (73.73 to 91.36)	8.83% (1.37 to 17.1)	21.82% (17.3 to 26.71)	66.19% (50.77 to 77.05)	33.72% (11.18 to 54.71)	7.02% (4.61 to 9.61)
Seychelles	84% (74.54 to 91.07)	9.71% (1.5 to 18.18)	23.49% (18.43 to 28.45)	61.85% (46.24 to 73.38)	36.44% (14.16 to 56.71)	13.57% (10.18 to 17.24)
Sierra Leone	82.01% (72.11 to 89.48)	4.96% (0.67 to 9.35)	13.58% (10.59 to 16.83)	67.78% (53.06 to 78.88)	27.9% (9.88 to 44.86)	10.97% (8.18 to 14)
Singapore	74.7% (62 to 84.45)	5.94% (0.88 to 11.3)	21.73% (17.51 to 26.29)	42.86% (29.93 to 55.22)	35.04% (13.02 to 54.54)	11.5% (8.01 to 15.09)
Slovakia	82.73% (72.18 to 90.54)	10.15% (1.55 to 19.61)	20.77% (16.18 to 25.06)	64.25% (48.01 to 75.84)	32.82% (11.28 to 52.79)	7.99% (5.4 to 10.71)
Slovenia	81.07% (69.96 to 89.07)	8.15% (1.19 to 16.24)	21.98% (17.75 to 26.47)	62.15% (46.11 to 72.95)	29.93% (9.45 to 50.31)	7.96% (5.02 to 11.02)
Solomon Islands	73.17% (60.95 to 83.35)	9.42% (1.46 to 17.66)	15.77% (12 to 19.47)	45.49% (30.8 to 58.27)	32.81% (12.46 to 51.08)	10.63% (8.09 to 13.29)
Somalia	72.65% (60.38 to 82.59)	4.21% (0.57 to 7.72)	10.28% (7.9 to 13.01)	54.92% (38.98 to 68.13)	25.79% (9.18 to 42.66)	8.35% (6 to 10.84)
South Africa	82.01% (71.4 to 89.5)	11.42% (1.81 to 21.5)	16.92% (13.29 to 20.84)	64.74% (48.98 to 75.99)	27.99% (9.52 to 45.83)	13.19% (9.77 to 16.57)
South Sudan	73.51% (61.6 to 82.96)	1.95% (0.25 to 3.66)	11.02% (8.3 to 13.85)	56.85% (40.26 to 69.08)	24.98% (8.79 to 41.3)	8.31% (5.88 to 10.87)
Spain	76.47% (65.03 to 85.55)	7.42% (1.11 to 15.01)	20.23% (16.11 to 24.88)	54.63% (39.95 to 66.76)	28.74% (9.1 to 48.62)	8.21% (4.96 to 11.56)
Sri Lanka	81.25% (70.82 to 89.11)	3.87% (0.53 to 7.5)	18.84% (14.86 to 23.23)	61.63% (45.61 to 73.08)	31.83% (10.89 to 51.75)	12.72% (9.1 to 16.44)
Sudan	83.01% (73.03 to 90.18)	12.72% (2.03 to 23.57)	18.43% (14.12 to 22.81)	65.12% (49.53 to 76.48)	32.13% (12.61 to 50.61)	10.97% (7.97 to 14.1)
Suriname	76.01% (64.27 to 85.48)	6.73% (0.97 to 13.04)	21.84% (17.23 to 26.56)	49.42% (34.63 to 62.9)	33.11% (11.85 to 52.61)	9.16% (6.6 to 11.69)
Sweden	78.17% (66.44 to 87.35)	5.45% (0.78 to 10.93)	17.6% (13.61 to 21.96)	57.34% (42.15 to 68.93)	30.47% (9.65 to 51)	9.58% (6.09 to 13.19)
Switzerland	76.11% (64.18 to 85.84)	5.08% (0.71 to 10.3)	19.03% (14.8 to 23.67)	51.82% (35.86 to 64.84)	31.41% (10.07 to 52.53)	9.88% (6.23 to 13.87)
Syrian Arab Republic	81.5% (71.31 to 89.8)	16.5% (2.78 to 29.44)	21.05% (16.86 to 25.76)	58.24% (43.85 to 70.2)	35.1% (13.87 to 54.48)	10.83% (7.84 to 14.05)
Taiwan (Province of China)	76.53% (64.4 to 86.17)	5.81% (0.84 to 11.07)	19.59% (15.5 to 24.03)	50.63% (35.22 to 63.77)	34.7% (12.5 to 54.79)	8.7% (6.08 to 11.26)
Tajikistan	80.75% (70.01 to 88.84)	10.31% (1.6 to 19.52)	15.63% (12.21 to 19.3)	60.73% (44.85 to 73.09)	32.36% (12.02 to 50.78)	12.42% (9.45 to 15.47)

Thailand	77.91% (66.76 to 86.91)	5.41% (0.81 to 10.24)	17.03% (13.22 to 20.95)	51.06% (36.05 to 63.71)	35.63% (13.09 to 55.33)	14.3% (10.8 to 17.84)
Timor-Leste	78.52% (67.45 to 86.87)	1.3% (0.16 to 2.47)	13.46% (10.49 to 16.81)	61.82% (47.48 to 74.04)	26.27% (9.04 to 43.6)	13.14% (9.81 to 16.65)
Togo	80.97% (70.54 to 89.19)	6.07% (0.88 to 11.46)	11.82% (9.07 to 14.84)	64.33% (48.09 to 76.02)	31.06% (11.31 to 49.33)	10.9% (8.26 to 13.69)
Tokelau	82.07% (71.89 to 89.43)	11.36% (1.87 to 20.83)	33.19% (27.01 to 39.44)	56.57% (39.67 to 68.72)	29.68% (10.23 to 48.23)	10.62% (7.7 to 13.7)
Tonga	80.84% (69.96 to 89.29)	14.41% (2.49 to 25.44)	23.73% (18.82 to 28.58)	58.38% (42.53 to 71.51)	32.16% (11.54 to 50.9)	10.74% (7.82 to 13.78)
Trinidad and Tobago	80.72% (70.97 to 88.52)	8.85% (1.36 to 16.7)	21.84% (17.32 to 26.94)	60.87% (46.81 to 72.42)	31.14% (10.77 to 50.29)	9.27% (6.65 to 12.1)
Tunisia	77.86% (66.97 to 86.6)	10.16% (1.6 to 19.05)	20.62% (16.32 to 25.5)	53.13% (38.98 to 65.68)	32.98% (11.6 to 52.05)	10.37% (7.23 to 13.62)
Turkey	79.19% (69.08 to 87.28)	11.53% (1.79 to 21.79)	21.59% (17.17 to 26.51)	56.57% (41.77 to 67.89)	30.12% (10.49 to 49.22)	11.11% (7.54 to 14.9)
Turkmenistan	83.04% (73.02 to 90.59)	10.71% (1.66 to 20.36)	14.89% (11.6 to 18.47)	63.64% (48.97 to 75.86)	35.56% (14.09 to 54.9)	13.72% (10.78 to 16.75)
Tuvalu	78.82% (66.68 to 87.45)	13.33% (2.26 to 24.12)	17.43% (13.57 to 21.58)	56.35% (40.6 to 68.93)	31.99% (11.49 to 50.37)	11.25% (8.52 to 14.21)
Uganda	74.36% (63.25 to 83.79)	3.85% (0.59 to 7.1)	11.75% (9.1 to 14.86)	59.09% (44.74 to 70.79)	22.49% (7.44 to 38.51)	8.63% (5.94 to 11.35)
Ukraine	82.41% (71.91 to 90.36)	10.11% (1.56 to 19.1)	13.58% (10.71 to 17.2)	64.49% (47.76 to 75.9)	34.58% (12.14 to 54.6)	11.63% (8.24 to 15.11)
United Arab Emirates	85.85% (75 to 93.2)	23.53% (4.27 to 40.13)	23.72% (18.92 to 28.59)	55.46% (39.83 to 68.48)	49.02% (22.33 to 68.47)	11.76% (9.18 to 14.4)
United Kingdom	74.33% (62.61 to 84.13)	7.46% (1.09 to 14.83)	15.63% (12.22 to 19.49)	51.54% (38.08 to 62.22)	29.79% (9.52 to 49.97)	9.45% (5.9 to 13.03)
United Republic of Tanzania	75.27% (62.45 to 84.62)	5.91% (0.84 to 11.33)	11.32% (8.76 to 14.17)	56.95% (41.82 to 69.69)	30.04% (10.92 to 48.24)	8.27% (5.66 to 11.07)
United States of America	77.44% (66.99 to 86.32)	10.48% (1.63 to 19.51)	24.93% (19.95 to 29.95)	51.8% (37.08 to 64.7)	27.71% (9.24 to 46.22)	11.08% (7.41 to 14.8)
United States Virgin Islands	78.5% (68.09 to 87.06)	9.73% (1.53 to 18.61)	25.54% (20.49 to 31.49)	55.47% (39.81 to 68.07)	28.2% (9.33 to 47.24)	8.97% (6.22 to 11.86)
Uruguay	77.87% (66.63 to 86.71)	7.65% (1.14 to 15.37)	17.19% (13.45 to 21.39)	57.47% (40.53 to 69.28)	30.89% (10.08 to 51.19)	8.07% (5.04 to 11.07)
Uzbekistan	81.62% (71.17 to 89.54)	11.55% (1.8 to 22)	15.01% (11.72 to 18.47)	60.14% (44.86 to 72.78)	37.03% (14.61 to 56.72)	13.66% (10.65 to 16.76)
Vanuatu	84.19% (74.9 to 90.72)	9.51% (1.46 to 18.15)	21.09% (16.68 to 25.76)	64.9% (49.07 to 76.17)	35.81% (13.99 to 55.2)	11.33% (8.66 to 14.1)
Venezuela (Bolivarian Republic of)	80.31% (69.97 to 88.6)	9.96% (1.54 to 18.92)	18.6% (14.71 to 22.66)	61.37% (45.55 to 73.14)	26.75% (8.92 to 44.75)	12.08% (8.57 to 15.53)
Viet Nam	80.62% (70 to 88.62)	1.58% (0.2 to 3.01)	13.79% (10.62 to 17.16)	64.4% (48.84 to 76.1)	29.3% (10.09 to 47.99)	10.56% (7.44 to 13.84)
Yemen	77.9% (65.79 to 86.98)	8.34% (1.22 to 15.46)	15.97% (12.45 to 19.87)	53.87% (38.06 to 66.9)	35.07% (13.65 to 54.53)	10.48% (7.65 to 13.42)
Zambia	71.92% (59.53 to 82.01)	5.95% (0.86 to 11.21)	15.46% (11.99 to 19.25)	53.06% (38.11 to 66.07)	22.74% (7.62 to 38.49)	8.35% (5.86 to 11.17)

Zimbabwe	81.84% (71.26 to 89.35)	8.12% (1.18 to 15.36)	14.38% (11.12 to 17.84)	66.08% (50.42 to 77.29)	27.2% (9.54 to 44.82)	13.18% (10.11 to 16.4)

Note: Percentage data are presented to one decimal place. 95% uncertainty intervals are presented to one decimal places, and if necessary, to two decimal places. DALY=disability-adjusted life-year. Metabolic risks cluster includes high body-mass index, high fasting plasma glucose, high systolic blood pressure, high LDL cholesterol and kidney dysfunction.

eTable 11d. Percent of DALYs (with 95% uncertainty intervals) due to IS for tobacco and behavioral risks by country and territory for both sexes combined in 2021, aged ≥20 years

	**Tobacco**	Smoking	Secondhand smoke	**Low physical activity**
Afghanistan	11.26% (8.94 to 13.94)	7.21% (5.73 to 9.12)	4.41% (2.9 to 6)	8.56% (4.08 to 12.93)
Albania	19.5% (15.56 to 23.57)	15.82% (12.75 to 19.33)	4.25% (2.77 to 5.8)	3.35% (-0.64 to 7.75)
Algeria	15.14% (11.98 to 18.8)	10% (7.94 to 12.65)	5.65% (3.85 to 7.55)	7.08% (2.52 to 12.43)
American Samoa	15.86% (12.49 to 19.28)	11.08% (8.93 to 13.29)	5.47% (3.67 to 7.4)	9.86% (4.73 to 15.53)
Andorra	10.37% (8.07 to 13.11)	8.86% (6.83 to 11.25)	1.78% (1.19 to 2.46)	4% (-1.42 to 9.66)
Angola	8.84% (6.96 to 10.86)	7.12% (5.61 to 8.75)	1.89% (1.26 to 2.56)	4.11% (1.57 to 7.09)
Antigua and Barbuda	7.15% (5.52 to 9.14)	5.37% (4.18 to 6.9)	1.9% (1.26 to 2.59)	4.65% (0.85 to 9.26)
Argentina	11.2% (9.01 to 13.53)	8.6% (7.07 to 10.25)	2.98% (2 to 3.99)	2.76% (0.04 to 6.02)
Armenia	19.68% (16.21 to 23.09)	15.34% (13.08 to 17.67)	4.93% (3.34 to 6.56)	3.35% (0.2 to 6.84)
Australia	6.57% (5.11 to 8.3)	5.36% (4.19 to 6.82)	1.34% (0.89 to 1.83)	4.71% (-1.01 to 11.09)
Austria	10.97% (8.64 to 13.44)	9.42% (7.5 to 11.44)	1.86% (1.23 to 2.52)	3.71% (-1.56 to 9.31)
Azerbaijan	18.95% (15.11 to 23.04)	13.79% (10.91 to 17.06)	5.76% (3.92 to 7.64)	3.74% (1.05 to 7.25)
Bahamas	7.54% (5.92 to 9.47)	5.69% (4.41 to 7.24)	1.99% (1.31 to 2.68)	5.2% (1.41 to 9.63)
Bahrain	16.53% (13.6 to 19.93)	12.56% (10.49 to 15.03)	4.6% (3.08 to 6.22)	8.42% (3.9 to 13.38)
Bangladesh	15.81% (12.13 to 19.62)	11.34% (8.73 to 14.28)	4.94% (3.4 to 6.6)	3.41% (0.88 to 6.41)
Barbados	4.61% (3.46 to 5.92)	3.65% (2.75 to 4.71)	1.01% (0.66 to 1.39)	6.01% (0.98 to 11.87)
Belarus	16.78% (14.03 to 19.76)	14.12% (12.15 to 16.41)	3.03% (2.05 to 4.11)	3.8% (0.77 to 7.55)
Belgium	9.96% (7.85 to 12.32)	8.77% (6.94 to 10.75)	1.38% (0.93 to 1.92)	3.93% (-2.06 to 9.81)
Belize	9.01% (7.09 to 11.25)	7% (5.61 to 8.72)	2.16% (1.4 to 2.99)	4.74% (1.23 to 8.8)
Benin	4.75% (3.63 to 6.18)	3.38% (2.57 to 4.45)	1.44% (0.95 to 1.96)	1.92% (0.52 to 3.61)

Bermuda	8.43% (6.46 to 10.87)	6.48% (5.02 to 8.36)	2.11% (1.38 to 2.89)	3.87% (-0.39 to 8.81)
Bhutan	8.24% (6.01 to 10.57)	5.53% (4.12 to 7.37)	2.9% (1.92 to 3.91)	9.61% (3.48 to 16.09)
Bolivia (Plurinational State of)	7% (5.39 to 8.79)	5.22% (4.17 to 6.62)	1.91% (1.14 to 2.7)	3.89% (0.91 to 7.4)
Bosnia and Herzegovina	17.24% (13.94 to 20.6)	13.88% (11.36 to 16.39)	3.98% (2.63 to 5.5)	3.77% (0.27 to 7.9)
Botswana	13.63% (10.85 to 16.71)	10.27% (8.22 to 12.48)	3.81% (2.46 to 5.05)	7.04% (2.54 to 12.15)
Brazil	12.05% (9.64 to 14.74)	9.66% (7.9 to 11.78)	2.71% (1.83 to 3.71)	6.07% (1.56 to 11.37)
Brunei Darussalam	13.53% (10.84 to 16.62)	11.27% (9.09 to 13.76)	2.63% (1.78 to 3.5)	6.24% (2.57 to 10.8)
Bulgaria	12.36% (9.83 to 14.7)	9.6% (8.05 to 11.3)	3.16% (2.04 to 4.32)	3.28% (-0.04 to 7.22)
Burkina Faso	6.58% (4.94 to 8.52)	4.02% (3 to 5.2)	2.7% (1.78 to 3.74)	3.03% (1.12 to 5.26)
Burundi	4.9% (3.71 to 6.42)	3.64% (2.74 to 4.83)	1.32% (0.83 to 1.89)	2.75% (0.98 to 4.68)
Cabo Verde	5.92% (4.47 to 7.44)	3.84% (3.11 to 4.84)	2.2% (1.45 to 3.01)	2.29% (0.4 to 4.63)
Cambodia	18.66% (14.8 to 22.78)	14.39% (11.62 to 17.78)	4.74% (3.2 to 6.32)	1.85% (0.39 to 3.8)
Cameroon	6.86% (5.14 to 8.62)	5.13% (3.91 to 6.46)	1.84% (1.19 to 2.52)	4.13% (1.62 to 6.91)
Canada	10.77% (8.54 to 13.28)	9.32% (7.46 to 11.53)	1.71% (1.13 to 2.29)	2.42% (-0.94 to 6.45)
Central African Republic	6.69% (4.87 to 8.75)	4.77% (3.49 to 6.44)	2.04% (1.32 to 2.75)	3.77% (1.52 to 6.26)
Chad	7.24% (5.31 to 9.46)	5.34% (3.85 to 7.02)	2.04% (1.36 to 2.79)	3.57% (1.48 to 5.78)
Chile	9.86% (7.71 to 12.33)	5.97% (4.92 to 7.28)	4.21% (2.74 to 5.79)	2.94% (0.03 to 6.15)
China	21.02% (17.17 to 24.98)	16.74% (14.04 to 19.75)	4.98% (3.35 to 6.77)	4.64% (1.03 to 8.92)
Colombia	6.8% (5.28 to 8.5)	4.63% (3.73 to 5.71)	2.31% (1.48 to 3.2)	5.77% (2.14 to 10.19)
Comoros	8.4% (5.98 to 11)	5.1% (3.73 to 6.76)	3.48% (2.26 to 4.81)	2.7% (0.89 to 4.78)
Congo	8.01% (6.1 to 10.24)	5.67% (4.27 to 7.36)	2.49% (1.66 to 3.39)	3.74% (1.44 to 6.1)
Cook Islands	12.96% (10.14 to 16.25)	8.85% (7.12 to 10.96)	4.6% (3.1 to 6.25)	4.91% (1.7 to 8.52)
Costa Rica	8.21% (6.48 to 10.01)	6.14% (4.93 to 7.35)	2.24% (1.49 to 3.03)	3.25% (0.08 to 6.78)
Coted'Ivoire	10.05% (7.71 to 12.66)	6.91% (5.36 to 8.64)	3.42% (2.28 to 4.56)	4.14% (1.69 to 6.82)
Croatia	15.13% (11.81 to 18.77)	11.42% (9.13 to 14.17)	4.25% (2.81 to 5.88)	3.44% (-1.46 to 8.6)
Cuba	13.4% (10.78 to 16.37)	10.48% (8.59 to 12.64)	3.28% (2.15 to 4.45)	4.16% (0.5 to 8.37)

Cyprus	11.59% (9.01 to 14.58)	9.48% (7.39 to 12.02)	2.41% (1.59 to 3.27)	3.74% (-2.23 to 9.82)
Czechia	13.23% (10.74 to 16.04)	10.71% (8.85 to 12.8)	2.9% (1.93 to 3.91)	4.25% (-0.45 to 9.35)
Democratic People's Republic of Korea	18.01% (14.54 to 21.47)	13.3% (10.8 to 15.87)	5.34% (3.62 to 7.08)	4.85% (1.63 to 8.3)
Democratic Republic of the Congo	4.87% (3.58 to 6.55)	3.08% (2.24 to 4.21)	1.86% (1.13 to 2.64)	4.74% (1.62 to 8.17)
Denmark	13.41% (10.73 to 16.47)	12.33% (9.89 to 15.17)	1.3% (0.85 to 1.79)	3.66% (-1.33 to 9.01)
Djibouti	14.69% (11.11 to 18.69)	10.46% (8.08 to 13.28)	4.63% (2.93 to 6.45)	2.89% (1.1 to 4.95)
Dominica	6.06% (4.64 to 7.78)	4.25% (3.3 to 5.52)	1.91% (1.25 to 2.66)	3.34% (-0.29 to 7.32)
Dominican Republic	12.91% (10.05 to 15.96)	10.92% (8.57 to 13.68)	2.25% (1.53 to 3.07)	5.12% (1.58 to 9.28)
Ecuador	6.57% (5.2 to 8.22)	5.01% (4.06 to 6.13)	1.65% (1.04 to 2.33)	3.04% (0.72 to 5.61)
Egypt	18.19% (14.94 to 21.92)	13.92% (11.46 to 16.52)	4.9% (3.33 to 6.5)	7.08% (3.13 to 11.36)
El Salvador	6.14% (4.69 to 7.63)	4.31% (3.32 to 5.34)	1.96% (1.3 to 2.61)	3.34% (0.59 to 6.73)
Equatorial Guinea	8.13% (5.76 to 10.69)	4.51% (3.24 to 6.14)	3.8% (2.41 to 5.32)	3.89% (1.37 to 6.7)
Eritrea	5.54% (4.1 to 7.27)	3.29% (2.32 to 4.54)	2.36% (1.59 to 3.2)	1.67% (0.63 to 2.93)
Estonia	12.24% (10.11 to 14.69)	10.32% (8.68 to 12.31)	2.25% (1.44 to 3.04)	3.23% (-0.6 to 7.68)
Eswatini	6.57% (4.92 to 8.55)	4.48% (3.38 to 5.98)	2.21% (1.41 to 3.17)	5.23% (2.01 to 8.91)
Ethiopia	3.58% (2.72 to 4.67)	2.17% (1.66 to 2.85)	1.46% (0.97 to 2.04)	3.08% (1.14 to 5.38)
Fiji	14.03% (11.23 to 17.01)	9.98% (8.32 to 11.92)	4.51% (2.89 to 6.21)	6.38% (2.76 to 10.28)
Finland	6.88% (5.34 to 8.53)	6.04% (4.69 to 7.49)	0.95% (0.63 to 1.3)	3.71% (-1.59 to 9.27)
France	8.68% (6.78 to 10.83)	7.39% (5.85 to 9.18)	1.48% (0.94 to 2.03)	3.89% (-2.29 to 9.89)
Gabon	6.87% (5.21 to 8.82)	4.46% (3.34 to 5.85)	2.55% (1.63 to 3.51)	2.05% (0.52 to 3.77)
Gambia	9.69% (7.36 to 12.21)	6.35% (4.98 to 8.03)	3.58% (2.37 to 4.82)	2.9% (1.03 to 5.12)
Georgia	16.54% (13.41 to 19.9)	12.25% (10.33 to 14.57)	4.85% (3.25 to 6.6)	3.41% (-0.6 to 7.88)
Germany	10.18% (8.3 to 12.6)	8.98% (7.33 to 11.12)	1.41% (0.93 to 1.92)	3.33% (-1.37 to 8.57)
Ghana	6.79% (5.17 to 8.61)	5.38% (4.13 to 6.81)	1.52% (0.98 to 2.11)	4.37% (1.89 to 7.18)
Greece	11.36% (8.65 to 14.3)	9.21% (7.03 to 11.57)	2.44% (1.59 to 3.36)	2.85% (-3.09 to 8.65)
Greenland	19.58% (16.1 to 23.66)	16.98% (14.08 to 20.43)	3.36% (2.19 to 4.57)	3.7% (0.84 to 6.9)

Grenada	6.42% (5.09 to 8.08)	4.61% (3.69 to 5.79)	1.91% (1.27 to 2.67)	4.44% (1.12 to 8.5)
Guam	14.61% (11.83 to 17.83)	10.67% (8.73 to 12.96)	4.51% (3.09 to 6.07)	4.01% (1.66 to 6.85)
Guatemala	6.67% (4.99 to 8.44)	4.64% (3.63 to 5.86)	2.14% (1.31 to 2.99)	2.18% (0 to 4.64)
Guinea	8.59% (6.64 to 11.04)	5.99% (4.55 to 7.82)	2.8% (1.86 to 3.77)	2.05% (0.6 to 3.78)
Guinea-Bissau	6.92% (5.2 to 8.82)	4.84% (3.61 to 6.26)	2.21% (1.48 to 2.95)	4.43% (1.81 to 7.14)
Guyana	8.41% (6.61 to 10.54)	6.24% (4.99 to 7.76)	2.34% (1.57 to 3.18)	5.14% (1.67 to 9.04)
Haiti	4.92% (3.73 to 6.23)	3.42% (2.64 to 4.5)	1.56% (1 to 2.18)	5.7% (2.19 to 9.76)
Honduras	10.71% (8.22 to 13.61)	7.65% (5.85 to 9.75)	3.33% (2.2 to 4.55)	3.63% (1.08 to 6.75)
Hungary	12.36% (10.2 to 14.9)	10.13% (8.51 to 11.97)	2.61% (1.74 to 3.52)	3.89% (0.13 to 8.45)
Iceland	8.96% (6.96 to 11.38)	7.91% (6.19 to 10.04)	1.2% (0.78 to 1.68)	3.8% (-1.3 to 9.57)
India	12.08% (9.58 to 15)	8.66% (6.88 to 10.78)	3.76% (2.55 to 5.07)	5% (1.56 to 8.81)
Indonesia	18.29% (14.56 to 22.18)	13.66% (10.55 to 16.97)	5.13% (3.5 to 6.89)	9.59% (3.81 to 16.26)
Iran (Islamic Republic of)	12.39% (10.03 to 14.72)	9.71% (8.07 to 11.44)	3.02% (2.05 to 4.07)	6.57% (2.57 to 10.9)
Iraq	19.95% (16.23 to 23.68)	14.92% (12.5 to 17.43)	5.85% (3.99 to 7.72)	8.92% (3.9 to 14.3)
Ireland	10.26% (8.01 to 12.67)	8.7% (6.88 to 10.85)	1.77% (1.15 to 2.44)	4.38% (-1.94 to 11.05)
Israel	9.53% (7.57 to 11.72)	7.92% (6.38 to 9.73)	1.84% (1.22 to 2.46)	3.89% (-0.83 to 8.73)
Italy	7.6% (5.83 to 9.65)	6.07% (4.68 to 7.62)	1.7% (1.11 to 2.4)	3.65% (-3.28 to 10.31)
Jamaica	7.68% (5.97 to 9.8)	5.69% (4.5 to 7.26)	2.13% (1.42 to 2.89)	5.56% (0.62 to 10.92)
Japan	9.61% (7.48 to 11.8)	7.93% (6.27 to 9.78)	1.87% (1.23 to 2.57)	3.84% (-1 to 8.91)
Jordan	20.99% (17.49 to 24.54)	16.34% (13.99 to 18.77)	5.57% (3.86 to 7.36)	6.9% (2.84 to 11.59)
Kazakhstan	14.02% (11.46 to 16.57)	10.53% (8.92 to 12.18)	3.92% (2.65 to 5.23)	4.07% (1.25 to 7.5)
Kenya	6.41% (4.9 to 7.95)	4.54% (3.4 to 5.76)	1.96% (1.32 to 2.64)	4.24% (1.7 to 7.09)
Kiribati	31.03% (26.05 to 35.91)	24.67% (21.28 to 28.4)	8.52% (5.94 to 11.15)	9.49% (4.53 to 14.35)
Kuwait	20.82% (17.01 to 24.51)	16.12% (13.52 to 18.54)	5.58% (3.78 to 7.43)	12.84% (6.8 to 18.78)
Kyrgyzstan	24.78% (20.91 to 28.58)	20.32% (17.62 to 23.27)	5.37% (3.68 to 7.2)	3.73% (1.33 to 6.38)
Lao People's Democratic Republic	19.83% (16.12 to 23.77)	14.94% (12.4 to 17.81)	5.52% (3.71 to 7.42)	2.56% (0.84 to 4.68)

Latvia	10.59% (8.29 to 12.88)	7.85% (6.47 to 9.38)	3.01% (1.94 to 4.21)	3.27% (-0.19 to 7.1)
Lebanon	21.14% (17.09 to 25.46)	17.16% (14.05 to 20.44)	4.96% (3.32 to 6.62)	5.87% (1.33 to 11.2)
Lesotho	14.21% (10.47 to 17.96)	10.13% (7.4 to 13.15)	4.51% (3.11 to 5.97)	2.36% (0.43 to 4.78)
Liberia	6.49% (4.94 to 8.22)	4.66% (3.61 to 5.95)	1.95% (1.25 to 2.7)	5.84% (2.62 to 9.36)
Libya	17.26% (13.27 to 21.01)	10.91% (8.41 to 13.78)	7.08% (4.92 to 9.34)	8.75% (4.09 to 13.74)
Lithuania	11.13% (8.98 to 13.59)	9.04% (7.44 to 10.9)	2.37% (1.56 to 3.28)	4.35% (-0.01 to 9.35)
Luxembourg	9.92% (7.73 to 12.88)	8.53% (6.64 to 11.11)	1.6% (1.05 to 2.18)	3.53% (-1.72 to 9.4)
Madagascar	5.96% (4.48 to 7.61)	3.67% (2.86 to 4.7)	2.39% (1.46 to 3.39)	2.89% (1.11 to 4.83)
Malawi	10.61% (7.97 to 13.79)	8.33% (6.06 to 10.97)	2.5% (1.65 to 3.41)	2.54% (0.96 to 4.44)
Malaysia	14.3% (11.3 to 17.13)	9.75% (8.19 to 11.47)	5.01% (3.42 to 6.72)	6.53% (2.67 to 10.72)
Maldives	19.45% (15.83 to 23.67)	14.41% (12.11 to 17.09)	5.82% (3.96 to 7.76)	9.85% (3.89 to 16.52)
Mali	9.23% (6.94 to 11.67)	5.81% (4.35 to 7.46)	3.64% (2.39 to 4.93)	4.69% (1.9 to 7.81)
Malta	7.55% (5.88 to 9.49)	6.31% (4.96 to 7.86)	1.39% (0.92 to 1.92)	4.6% (-1.65 to 10.85)
Marshall Islands	15.24% (11.9 to 18.82)	10.54% (8.38 to 13.28)	5.28% (3.56 to 7.03)	14.29% (7.29 to 21)
Mauritania	8.17% (6.26 to 10.4)	4.3% (3.25 to 5.5)	4.1% (2.73 to 5.67)	7.23% (3.05 to 11.98)
Mauritius	16.93% (13.76 to 20.35)	12.22% (10.35 to 14.42)	5.28% (3.62 to 7.09)	4.11% (1.4 to 7.47)
Mexico	7.43% (5.88 to 9.08)	5.49% (4.46 to 6.56)	2.09% (1.43 to 2.79)	3.71% (1.06 to 6.97)
Micronesia (Federated States of)	22.08% (17.58 to 26.53)	16.31% (13.3 to 19.45)	6.99% (4.53 to 9.49)	13.53% (6.37 to 20.14)
Monaco	8.84% (6.59 to 11.53)	7.65% (5.67 to 10.04)	1.35% (0.88 to 1.85)	3.73% (-2.08 to 9.49)
Mongolia	22.59% (18.67 to 26.73)	18.21% (15.33 to 21.51)	5.16% (3.55 to 6.8)	2.68% (1.05 to 4.68)
Montenegro	16.3% (12.79 to 20.02)	12.49% (10.02 to 15.37)	4.43% (2.99 to 5.96)	3.58% (-0.34 to 8.09)
Morocco	9.7% (7.62 to 12.04)	5.49% (4.32 to 7.12)	4.46% (3.05 to 5.99)	7.74% (3.18 to 12.79)
Mozambique	8.84% (6.72 to 11.12)	6.88% (5.3 to 8.75)	2.13% (1.33 to 2.98)	2.72% (1 to 4.81)
Myanmar	14.58% (11.62 to 18.25)	10.97% (8.9 to 13.55)	4.07% (2.57 to 5.66)	1.92% (0.64 to 3.48)
Namibia	10.76% (8.46 to 13.28)	7.93% (6.3 to 9.71)	3.09% (1.96 to 4.41)	5.2% (1.71 to 9.66)
Nauru	20.84% (16.6 to 25.06)	15.19% (12.54 to 18.04)	6.87% (4.47 to 9.2)	7.43% (3.47 to 11.11)
Nepal	16.17% (12.98 to 19.78)	12.94% (10.42 to 15.63)	3.74% (2.41 to 5.13)	3.38% (1.04 to 6.24)

Netherlands	10.67% (8.37 to 13.42)	9.6% (7.56 to 12.04)	1.25% (0.83 to 1.73)	2.88% (-1.73 to 7.32)
New Zealand	8.61% (6.76 to 11)	7.41% (5.81 to 9.37)	1.36% (0.91 to 1.87)	4.36% (-2.12 to 10.79)
Nicaragua	10.28% (7.82 to 12.92)	6.73% (5.37 to 8.35)	3.82% (2.51 to 5.11)	3.63% (0.99 to 6.76)
Niger	4.55% (3.31 to 6)	2.65% (1.94 to 3.67)	1.96% (1.27 to 2.71)	4.01% (1.63 to 6.85)
Nigeria	4.22% (3.19 to 5.33)	2.94% (2.21 to 3.74)	1.33% (0.89 to 1.83)	4.39% (1.56 to 7.46)
Niue	11.94% (9.18 to 14.66)	8.26% (6.39 to 10.27)	4.1% (2.76 to 5.55)	4.95% (1.79 to 8.88)
North Macedonia	17.43% (13.94 to 21.14)	12.43% (10.21 to 14.96)	5.74% (3.91 to 7.75)	3.71% (0.02 to 7.95)
Northern Mariana Islands	16.39% (13.07 to 19.96)	11.96% (9.66 to 14.66)	5.1% (3.4 to 6.77)	6.01% (2.59 to 9.57)
Norway	7.63% (5.94 to 9.52)	6.54% (5.1 to 8.1)	1.23% (0.8 to 1.7)	3.71% (-1.43 to 9.1)
Oman	12.09% (9.54 to 14.48)	8.43% (6.96 to 9.96)	4.06% (2.74 to 5.44)	8.51% (3.85 to 13.24)
Pakistan	14.18% (10.83 to 17.45)	9.77% (7.59 to 12.18)	4.92% (3.39 to 6.54)	5.34% (2.25 to 8.74)
Palau	14.36% (11.41 to 17.56)	10.65% (8.73 to 13.12)	4.21% (2.84 to 5.7)	6.13% (2.81 to 9.72)
Palestine	17.07% (13.89 to 20.53)	12.83% (10.66 to 15.4)	4.82% (3.26 to 6.4)	7.46% (3.06 to 12.63)
Panama	6.3% (4.85 to 7.99)	4.75% (3.72 to 6.14)	1.64% (1.07 to 2.22)	3.04% (-0.2 to 6.52)
Papua New Guinea	15.57% (11.7 to 19.6)	9.83% (7.57 to 12.65)	6.43% (4.27 to 8.66)	6.48% (3.02 to 10.45)
Paraguay	14.75% (11.35 to 18.38)	12.01% (9.28 to 15.1)	3.14% (2.03 to 4.29)	3.17% (0.42 to 6.37)
Peru	6.55% (5.12 to 8.28)	5.34% (4.22 to 6.78)	1.3% (0.81 to 1.85)	3.7% (0.62 to 7.33)
Philippines	17.24% (14.14 to 20.53)	13.02% (10.89 to 15.32)	4.84% (3.33 to 6.45)	3.43% (1.16 to 5.86)
Poland	12.33% (10.03 to 14.84)	10.06% (8.42 to 12.07)	2.63% (1.75 to 3.59)	4.28% (0 to 9.09)
Portugal	5.82% (4.4 to 7.34)	3.98% (3.14 to 5.01)	1.96% (1.28 to 2.71)	3.71% (-2.87 to 10.29)
Puerto Rico	6.87% (5.25 to 8.98)	5.74% (4.4 to 7.52)	1.21% (0.79 to 1.67)	3.8% (-0.51 to 8.39)
Qatar	19.16% (15.36 to 23.15)	14.46% (12.03 to 17.21)	5.53% (3.73 to 7.32)	10.63% (5.35 to 15.61)
Republic of Korea	13.9% (11.1 to 17.01)	11.36% (9.22 to 13.93)	2.89% (1.93 to 3.97)	4.5% (0.29 to 9.68)
Republic of Moldova	14.7% (12.18 to 17.59)	12.4% (10.46 to 14.74)	2.63% (1.8 to 3.57)	3.94% (1.03 to 7.33)
Romania	9.55% (7.51 to 11.82)	7% (5.68 to 8.54)	2.83% (1.89 to 3.84)	3.64% (-0.19 to 7.86)
Russian Federation	14.03% (11.83 to 16.57)	11.74% (10.14 to 13.68)	2.66% (1.78 to 3.62)	3.7% (0.1 to 7.94)
Rwanda	13.43% (10.15 to 17.05)	11.52% (8.65 to 14.85)	2.16% (1.42 to 2.95)	2.69% (0.83 to 5.02)
Saint Kitts and Nevis	5.94% (4.6 to 7.55)	4.21% (3.25 to 5.43)	1.83% (1.19 to 2.5)	5.03% (1.45 to 9.36)

Saint Lucia	5.85% (4.58 to 7.34)	4.5% (3.51 to 5.71)	1.43% (0.94 to 1.97)	4.17% (0.02 to 8.96)
Saint Vincent and the Grenadines	6.8% (5.31 to 8.5)	4.9% (3.9 to 6.17)	2.02% (1.32 to 2.71)	4.47% (0.69 to 9)
Samoa	17.59% (14 to 21.34)	12.83% (10.34 to 15.38)	5.52% (3.83 to 7.35)	8.14% (3.74 to 13.06)
San Marino	8.57% (6.37 to 11.05)	7.44% (5.5 to 9.63)	1.29% (0.83 to 1.79)	3.72% (-1.88 to 9.73)
Sao Tome and Principe	4.78% (3.56 to 6.28)	3.71% (2.74 to 4.88)	1.14% (0.73 to 1.59)	2.66% (0.96 to 4.6)
Saudi Arabia	16.25% (13.04 to 19.66)	11.7% (9.49 to 14.11)	5.19% (3.5 to 6.88)	10.62% (5.43 to 15.94)
Senegal	8.28% (6.24 to 10.5)	4.91% (3.73 to 6.35)	3.57% (2.45 to 4.74)	4.78% (1.91 to 8.43)
Serbia	13.15% (10.28 to 16.02)	9.47% (7.72 to 11.58)	4.15% (2.77 to 5.52)	3.54% (-0.48 to 8.05)
Seychelles	16.96% (13.88 to 20.53)	13.25% (10.99 to 15.96)	4.26% (2.88 to 5.67)	4.23% (0.93 to 8.12)
Sierra Leone	9.1% (6.71 to 11.81)	6.05% (4.5 to 7.82)	3.27% (2.07 to 4.58)	4.1% (1.61 to 7.12)
Singapore	9.05% (7.35 to 10.95)	7.1% (5.84 to 8.39)	2.18% (1.49 to 2.97)	5.07% (1.82 to 9.05)
Slovakia	13.24% (10.48 to 15.97)	10.19% (8.39 to 12.36)	3.49% (2.32 to 4.68)	3.33% (0.22 to 6.93)
Slovenia	9.62% (7.48 to 11.96)	6.98% (5.56 to 8.66)	2.92% (1.89 to 3.99)	2.95% (-1.73 to 7.66)
Solomon Islands	19.96% (16.17 to 24.18)	14.16% (11.72 to 16.93)	6.78% (4.44 to 9.04)	7.75% (3.79 to 11.86)
Somalia	7.85% (5.23 to 10.73)	5.14% (3.33 to 7.53)	2.87% (1.75 to 4.06)	3.05% (1.28 to 5.16)
South Africa	9.74% (7.7 to 11.76)	6.26% (5.22 to 7.38)	3.78% (2.53 to 5.06)	7.32% (2.54 to 12.89)
South Sudan	8.04% (5.95 to 10.73)	5.62% (4.02 to 7.75)	2.57% (1.64 to 3.52)	2.68% (0.96 to 4.73)
Spain	9.44% (7.48 to 11.9)	7.28% (5.83 to 9.05)	2.42% (1.62 to 3.27)	3.82% (-1.96 to 9.63)
Sri Lanka	8.1% (6.09 to 10.31)	5.19% (4.18 to 6.38)	3.06% (1.93 to 4.47)	2.26% (0.44 to 4.51)
Sudan	11.76% (9.14 to 14.78)	8.13% (6.21 to 10.59)	3.97% (2.68 to 5.29)	15.2% (7.63 to 22.4)
Suriname	11.47% (9.07 to 14.2)	8.64% (6.81 to 10.78)	3.14% (2.09 to 4.2)	5.98% (2.23 to 10.75)
Sweden	9.79% (7.57 to 12.13)	8.88% (6.86 to 10.96)	1.04% (0.66 to 1.44)	3.11% (-1.59 to 8.03)
Switzerland	10.25% (7.97 to 12.92)	9.03% (7 to 11.35)	1.41% (0.92 to 1.95)	3.49% (-1.95 to 8.94)
Syrian Arab Republic	16.71% (13.51 to 19.69)	12.65% (10.45 to 14.77)	4.69% (3.18 to 6.19)	7.68% (3.23 to 12.7)
Taiwan (Province of China)	14.21% (11.26 to 17.2)	10.37% (8.44 to 12.28)	4.27% (2.87 to 5.7)	5.43% (1.65 to 9.88)

Tajikistan	10.88% (8.37 to 13.81)	7.68% (5.95 to 9.69)	3.5% (2.38 to 4.58)	3.7% (1.18 to 6.9)
Thailand	13.89% (11.4 to 16.69)	10.45% (8.82 to 12.25)	3.79% (2.56 to 5.09)	4.08% (0.9 to 8.03)
Timor-Leste	16.47% (12.45 to 20.48)	11.83% (8.56 to 15.24)	5.11% (3.31 to 6.98)	2.23% (0.69 to 4.14)
Togo	11.14% (8.68 to 14.17)	8.86% (6.89 to 11.34)	2.58% (1.71 to 3.43)	2.62% (0.94 to 4.27)
Tokelau	12.32% (9.44 to 15.4)	7.9% (6.24 to 9.83)	4.86% (3.21 to 6.58)	5.03% (1.81 to 8.95)
Tonga	16.53% (13.03 to 20.17)	11.64% (9.25 to 14.34)	5.53% (3.78 to 7.4)	5.65% (2.04 to 9.9)
Trinidad and Tobago	9.89% (7.61 to 12.15)	7.23% (5.76 to 8.96)	2.89% (1.83 to 3.84)	6.36% (1.86 to 11.33)
Tunisia	17.82% (14.06 to 22.02)	13.47% (10.43 to 16.87)	4.91% (3.32 to 6.48)	4.59% (1.03 to 8.97)
Turkey	14.74% (11.78 to 17.94)	10.25% (8.51 to 12.07)	5.04% (3.39 to 6.78)	5.62% (1.23 to 10.98)
Turkmenistan	14.25% (11.16 to 17.15)	10.12% (7.99 to 12.19)	4.65% (3.2 to 6.21)	3.98% (1.58 to 6.88)
Tuvalu	17.07% (13.41 to 20.97)	11.58% (9.39 to 14.28)	6.25% (4.19 to 8.45)	5.92% (2.44 to 9.48)
Uganda	5.66% (4.3 to 7.37)	4.14% (3.15 to 5.37)	1.59% (1.03 to 2.22)	2.63% (0.79 to 4.94)
Ukraine	12.93% (9.78 to 16.14)	10.24% (7.78 to 12.96)	3.03% (2.07 to 4.11)	2.92% (0.49 to 6.02)
United Arab Emirates	18.66% (15.01 to 22.54)	13.59% (11.12 to 16.16)	5.96% (4.08 to 7.9)	10.77% (5.24 to 15.85)
United Kingdom	9.53% (7.46 to 11.96)	8.67% (6.81 to 10.99)	1.01% (0.68 to 1.36)	6.1% (-1.76 to 14.15)
United Republic of Tanzania	9.12% (7.04 to 11.69)	7.26% (5.59 to 9.4)	2.04% (1.26 to 2.8)	1.13% (0.3 to 2.13)
United States of America	10.99% (8.92 to 13.46)	9.86% (8.05 to 12.08)	1.33% (0.89 to 1.81)	4% (-0.38 to 9.06)
United States Virgin Islands	7.1% (5.2 to 9.23)	5.42% (3.91 to 7.21)	1.79% (1.16 to 2.5)	3.66% (-0.28 to 7.84)
Uruguay	9.91% (7.82 to 12.08)	7.79% (6.24 to 9.45)	2.38% (1.62 to 3.2)	3.27% (-0.08 to 7.18)
Uzbekistan	13.55% (10.9 to 16.09)	9.8% (8.21 to 11.43)	4.17% (2.85 to 5.53)	2.46% (0.97 to 4.22)
Vanuatu	11.45% (9.14 to 14.08)	8.3% (6.76 to 10.16)	3.45% (2.26 to 4.64)	2.1% (0.77 to 3.61)
Venezuela (Bolivarian Republic of)	8.53% (6.62 to 10.62)	5.78% (4.63 to 7.17)	2.96% (1.9 to 4.08)	3.32% (0.37 to 6.78)
Viet Nam	19.46% (15.54 to 23.62)	15.53% (12.52 to 18.69)	4.53% (3.06 to 6.02)	3.34% (1.06 to 6.37)
Yemen	18.7% (15.15 to 22.49)	14.11% (11.52 to 17.13)	5.39% (3.67 to 7.22)	8.14% (3.55 to 12.37)
Zambia	7.97% (5.96 to 10.23)	5.77% (4.39 to 7.34)	2.35% (1.45 to 3.39)	3.46% (1.19 to 5.91)

Zimbabwe	13% (9.81 to 16.19)	9.95% (7.72 to 12.51)	3.38% (2.3 to 4.51)	3.98% (1.5 to 6.71)

Note: Percentage data are presented to one decimal place. 95% uncertainty intervals are presented to one decimal places, and if necessary, to two decimal places. DALY=disability-adjusted life-year. Tobacco risks cluster includes smoking and secondhand smoke. Behavioral risk cluster includes low physical activity.

eTable 12. Risk factors for ischemic stroke related DALYs among people aged ≥20 years at global in 2021, by sex

Risk classification	Both		Female		Male	
	Absolute NO (00000s)	Percent	Absolute NO (00000s)	Percent	Absolute NO (00000s)	Percent
Ambient particulate matter pollution	120.0 (83.4 to 151.4)	17.2% (12.3 to 21.4)	53.1 (35.6 to 67.7)	16.0% (11.1 to 20.2)	66.9 (45.9 to 85.5)	18.2% (13.1 to 22.6)
Household air pollution from solid fuels	62.9 (34.7 to 116.6)	9.0% (5.0 to 16.8)	32 (18.1 to 56.8)	9.7% (5.4 to 17.4)	31 (16.4 to 60.2)	8.4% (4.5 to 16.3)
Low temperature	34.5 (29.7 to 40.2)	4.9% (4.3 to 5.7)	16.3 (13.7 to 19.4)	4.9% (4.2 to 5.7)	18.2 (15.4 to 21.6)	5.0% (4.3 to 5.8)
High temperature	6.6 (0.7 to 15.4)	0.9% (0.1 to 2.2)	3 (0.3 to 6.9)	0.9% (0.1 to 2.1)	3.6 (0.4 to 8.3)	1.0% (0.1 to 2.3)
Lead exposure	51.8 (-7.1 to 113.4)	7.4% (-1.0 to 16.5)	21 (-2.8 to 47.1)	6.3% (-0.8 to 14.1)	30.8 (-4.3 to 66)	8.4% (-1.1 to 18.6)
Diet high in sodium	72.5 (17.9 to 160.1)	10.4% (2.6 to 22.5)	26.5 (4.6 to 64.9)	8.0% (1.4 to 18.9)	46 (12.8 to 95.8)	12.5% (3.6 to 25.7)
Diet high in red meat	1.2 (-1.3 to 11.6)	0.2% (-0.2 to 1.7)	0.3 (-1 to 4.6)	0.1% (-0.3 to 1.4)	0.9 (-0.9 to 6.5)	0.2% (-0.2 to 1.8)
Diet low in fruits	20.6 (10.9 to 32.7)	3.0% (1.6 to 4.6)	9.3 (4.7 to 15.3)	2.8% (1.5 to 4.5)	11.4 (6.1 to 17.6)	3.1% (1.7 to 4.7)
Diet high in processed meat	4.4 (1.0 to 7.8)	0.6% (0.2 to 1.1)	2.4 (0.6 to 4.2)	0.7% (0.2 to 1.2)	2 (0.5 to 3.6)	0.5% (0.1 to 0.9)
Diet low in vegetables	11.9 (6.4 to 18.3)	1.7% (0.9 to 2.6)	5.5 (2.7 to 8.7)	1.7% (0.8 to 2.6)	6.5 (3.6 to 9.7)	1.8% (1.0 to 2.6)
Diet low in whole grains	31.3 (-31.7 to 87.8)	4.5% (-4.6 to 12.3)	13.1 (-12.9 to 38.9)	3.9% (-4.0 to 11.3)	18.1 (-18.5 to 48.9)	4.9% (-5.1 to 13.3)
High alcohol use	38.7 (-6.0 to 96.2)	5.6% (-0.9 to 13.6)	6.5 (-0.8 to 16.7)	2.0% (-0.2 to 5.2)	32.2 (-5.2 to 79)	8.8% (-1.4 to 21.3)
Diet high in sugar-sweetened beverages	1.4 (0.7 to 2.3)	0.2% (0.1 to 0.3)	0.8 (0.4 to 1.2)	0.2% (0.1 to 0.4)	0.7 (0.3 to 1.1)	0.2% (0.1 to 0.3)
Diet low in fiber	9.5 (-0.4 to 19.2)	1.4% (-0.1 to 2.8)	4.3 (-0.2 to 9.4)	1.3% (-0.1 to 2.8)	5.2 (-0.2 to 10.4)	1.4% (-0.1 to 2.8)
Diet low in polyunsaturated fatty acids	0.2 (0.1 to 0.3)	0.03% (0.01 to 0.05)	0.1 (0 to 0.2)	0.0% (0.0 to 0.0)	0.1 (0 to 0.2)	0.0% (0.0 to 0.0)

Low physical activity	33.6 (9.1 to 63.0)	4.8% (1.3 to 9.0)	18.5 (4.4 to 35.8)	5.6% (1.4 to 10.5)	15.1 (5 to 27.9)	4.1% (1.3 to 7.4)
Smoking	85.1 (70.4 to 102.8)	12.2% (10.3 to 14.3)	10.4 (8.4 to 13.1)	3.1% (2.6 to 3.8)	74.7 (61.6 to 90.3)	20.3% (17.5 to 23.3)
Secondhand smoke	27.4 (18.2 to 36.8)	3.9% (2.7 to 5.2)	15.3 (10.3 to 20.9)	4.6% (3.1 to 6.2)	12.1 (7.9 to 16.3)	3.3% (2.3 to 4.4)
High body-mass index	44.4 (6.5 to 86.5)	6.4% (1.0 to 12.0)	23.3 (3.4 to 45.1)	7.0% (1.1 to 13.3)	21.1 (3.1 to 41.3)	5.7% (0.9 to 11.0)
High fasting plasma glucose	123.7 (95.9 to 153.8)	17.7% (14.0 to 21.8)	58.7 (44.7 to 73.7)	17.7% (14.0 to 21.8)	65 (50.8 to 81)	17.7% (13.9 to 21.7)
High systolic blood pressure	410.6 (311.5 to 502.0)	58.8% (44.6 to 70.0)	195.7 (149.7 to 241.8)	59.2% (44.3 to 70.5)	214.9 (158.4 to 265.5)	58.5% (43.7 to 69.9)
High LDL cholesterol	209.8 (74.1 to 346.6)	30.0% (10.4 to 48.6)	99.4 (34.4 to 164.9)	30.0% (10.3 to 48.9)	110.4 (39.7 to 181.8)	30.1% (10.6 to 48.3)
Kidney dysfunction	69.6 (49.4 to 91.6)	10.0% (7.1 to 12.9)	34 (23.6 to 45.3)	10.3% (7.2 to 13.4)	35.6 (25.4 to 46)	9.7% (7.0 to 12.5)
Combined risk factors						
All factors	622.5 (557.3 to 686.7)	89.2% (82.8 to 93.6)	290.4 (252.6 to 327.0)	87.9% (80.7 to 93.0)	331.6 (296.4 to 371.5)	90.3% (84.4 to 94.4)

Note: Data in parentheses are 95% uncertainty intervals. Count data in 100000 are presented to one decimal places and percentage data are presented to one decimal place. Percentages and number of DALYs are not mutually exclusive: the sum of percentages and number of DALYs in the columns exceeds the totals for all risk factors combined because of overlap between various risk factors. The crude sum of population attributable fraction (PAF) of the risk factors might exceed 100% because the effects of many of these risk factors are mediated partly or wholly through another risk factor or risk factors. DALY=disability-adjusted life-year.

eTable 13. Sociodemographic-index-related inequalities in prevalence, deaths, disease-adjusted life years (DALYs) for ischemic stroke, aged ≥20 years, both sexes

Region	Year	Inequality metrics	Prevalence (95% CI)	Deaths (95% CI)	DALYs (95% CI)
Global	1990	slope index of inequality	-172.72 (-280.03 to -65.41)	-3.89 (-16.7 to 8.93)	-234.84 (-459.39 to -10.28)
Global	2021	slope index of inequality	-272.63 (-369.65 to -175.61)	-40.75 (-49.77 to -31.73)	-756.96 (-919.56 to -594.36)
Global	1990	concentration index	0.06 (0.04 to 0.08)	0.04 (0 to 0.08)	0.02 (-0.02 to 0.05)
Global	2021	concentration index	0.04 (0.02 to 0.06)	-0.06 (-0.1 to -0.02)	-0.06 (-0.1 to -0.02)
High SDI	1990	slope index of inequality	38.3 (-206.7 to 283.29)	-33.6 (-64.94 to -2.27)	-666.91 (-1133.84 to -199.97)
High SDI	2021	slope index of inequality	-33.5 (-207.22 to 140.22)	-12.13 (-21.47 to -2.79)	-185.99 (-333.62 to -38.37)
High SDI	1990	concentration index	0.02 (-0.03 to 0.06)	-0.11 (-0.2 to -0.02)	-0.11 (-0.2 to -0.03)
High SDI	2021	concentration index	0.03 (0 to 0.07)	-0.14 (-0.24 to -0.04)	-0.11 (-0.2 to -0.01)
High-middle SDI	1990	slope index of inequality	-59.39 (-458.63 to 339.86)	-13.82 (-90.16 to 62.53)	-519.96 (-1723.7 to 683.78)
High-middle SDI	2021	slope index of inequality	1.5 (-308.23 to 311.24)	0.01 (-39.39 to 39.41)	-11.27 (-698.22 to 675.69)
High-middle SDI	1990	concentration index	0.05 (0.03 to 0.07)	0.13 (0.09 to 0.18)	0.1 (0.06 to 0.15)
High-middle SDI	2021	concentration index	-0.04 (-0.06 to -0.02)	0.01 (-0.04 to 0.05)	0 (-0.04 to 0.04)
Middle SDI	1990	slope index of inequality	-171.08 (-348.49 to 6.32)	-40.68 (-63.5 to -17.87)	-730.77 (-1135.62 to -325.93)
Middle SDI	2021	slope index of inequality	-84.72 (-284.54 to 115.11)	-10.78 (-33.19 to 11.63)	-213.01 (-590.36 to 164.35)
Middle SDI	1990	concentration index	-0.03 (-0.06 to 0.01)	-0.12 (-0.17 to -0.07)	-0.13 (-0.17 to -0.08)
Middle SDI	2021	concentration index	0.01 (-0.04 to 0.05)	-0.05 (-0.15 to 0.06)	-0.04 (-0.14 to 0.06)
Low-middle SDI	1990	slope index of inequality	94.72 (-147.53 to 336.97)	-5.08 (-25.08 to 14.92)	-62.67 (-472.32 to 346.97)
Low-middle SDI	2021	slope index of inequality	-102.29 (-367.04 to 162.47)	-13.8 (-32.52 to 4.93)	-205.8 (-548.12 to 136.53)
Low-middle SDI	1990	concentration index	-0.02 (-0.07 to 0.04)	0 (-0.09 to 0.1)	0 (-0.08 to 0.09)
Low-middle SDI	2021	concentration index	-0.1 (-0.15 to -0.05)	-0.07 (-0.15 to 0.01)	-0.06 (-0.14 to 0.02)

Low SDI	1990	slope index of inequality	126.55 (-135.06 to 388.16)	22.05 (-1.86 to 45.97)	404.21 (-64.22 to 872.64)
Low SDI	2021	slope index of inequality	-53.39 (-292.03 to 185.26)	3.19 (-21.33 to 27.7)	12.08 (-426.54 to 450.69)
Low SDI	1990	concentration index	0.03 (-0.01 to 0.07)	0.07 (0 to 0.14)	0.06 (0 to 0.13)
Low SDI	2021	concentration index	0 (-0.05 to 0.05)	0.03 (-0.05 to 0.1)	0.02 (-0.06 to 0.1)
Eastern Sub-Saharan Africa	1990	slope index of inequality	266.82 (17.48 to 516.17)	7.34 (-33.64 to 48.33)	128.45 (-692.96 to 949.86)
Eastern Sub-Saharan Africa	2021	slope index of inequality	242.68 (118.9 to 366.47)	1.41 (-24.09 to 26.92)	14.06 (-387.68 to 415.8)
Eastern Sub-Saharan Africa	1990	concentration index	0.05 (0.01 to 0.09)	0.05 (-0.05 to 0.15)	0.04 (-0.06 to 0.13)
Eastern Sub-Saharan Africa	2021	concentration index	0.04 (-0.02 to 0.11)	0.01 (-0.11 to 0.12)	0 (-0.12 to 0.11)
Western Sub-Saharan Africa	1990	slope index of inequality	405.92 (159.77 to 652.07)	18.29 (-6.78 to 43.36)	363.02 (-133.68 to 859.72)
Western Sub-Saharan Africa	2021	slope index of inequality	432.46 (169.38 to 695.53)	5.39 (-19.74 to 30.52)	151.76 (-297.41 to 600.94)
Western Sub-Saharan Africa	1990	concentration index	0.03 (-0.01 to 0.06)	0.05 (0.02 to 0.08)	0.05 (0.01 to 0.08)
Western Sub-Saharan Africa	2021	concentration index	0.05 (0.01 to 0.09)	0.02 (-0.03 to 0.07)	0.02 (-0.04 to 0.08)
Central Sub-Saharan Africa	1990	slope index of inequality	236.56 (-32.66 to 505.78)	-0.6 (-19.69 to 18.49)	-64.22 (-447.3 to 318.86)
Central Sub-Saharan Africa	2021	slope index of inequality	251.22 (63.61 to 438.83)	-6.49 (-24.27 to 11.29)	-94.39 (-367.68 to 178.89)
Central Sub-Saharan Africa	1990	concentration index	-0.01 (-0.05 to 0.04)	-0.01 (-0.04 to 0.03)	-0.01 (-0.05 to 0.03)
Central Sub-Saharan Africa	2021	concentration index	0.05 (0.03 to 0.07)	0.01 (-0.02 to 0.05)	0.02 (-0.01 to 0.05)

North Africa and Middle East	1990	slope index of inequality	109.09 (-131.18 to 349.36)	-37.03 (-65.15 to -8.91)	-813.39 (-1255.16 to -371.62)
North Africa and Middle East	2021	slope index of inequality	6.71 (-177.41 to 190.83)	-62.97 (-86.26 to -39.69)	-1151.96 (-1561.15 to -742.76)
North Africa and Middle East	1990	concentration index	0.03 (-0.01 to 0.06)	-0.08 (-0.15 to -0.01)	-0.09 (-0.15 to -0.03)
North Africa and Middle East	2021	concentration index	-0.02 (-0.05 to 0.02)	-0.16 (-0.24 to -0.09)	-0.17 (-0.24 to -0.09)
Oceania	1990	slope index of inequality	40.12 (-322.46 to 402.71)	-2.91 (-27.79 to 21.98)	-49.97 (-529.75 to 429.81)
Oceania	2021	slope index of inequality	415.93 (-197.44 to 1029.31)	-17.54 (-65.41 to 30.32)	-267.08 (-1169.72 to 635.56)
Oceania	1990	concentration index	0.08 (0.03 to 0.14)	0 (-0.03 to 0.02)	0.01 (-0.02 to 0.04)
Oceania	2021	concentration index	0.11 (0.09 to 0.13)	0.01 (-0.02 to 0.03)	0.02 (0 to 0.05)
South Asia	1990	slope index of inequality	-118.27 (-542.44 to 305.9)	-26.29 (-54.68 to 2.1)	-426.53 (-843.75 to -9.3)
South Asia	2021	slope index of inequality	-117.6 (-862.79 to 627.59)	-19.65 (-65.84 to 26.54)	-343.01 (-1528.75 to 842.73)
South Asia	1990	concentration index	-0.05 (-0.09 to -0.01)	-0.1 (-0.16 to -0.03)	-0.08 (-0.13 to -0.03)
South Asia	2021	concentration index	-0.06 (-0.11 to -0.02)	-0.12 (-0.18 to -0.05)	-0.1 (-0.14 to -0.05)
Caribbean	1990	slope index of inequality	-54.18 (-289.8 to 181.44)	-35.04 (-83.3 to 13.21)	-706.57 (-1423.59 to 10.45)
Caribbean	2021	slope index of inequality	-73.2 (-224.45 to 78.06)	-32.26 (-69 to 4.48)	-638.91 (-1218.21 to -59.61)
Caribbean	1990	concentration index	-0.02 (-0.06 to 0.01)	-0.16 (-0.27 to -0.06)	-0.18 (-0.29 to -0.07)
Caribbean	2021	concentration index	-0.04 (-0.07 to -0.01)	-0.22 (-0.31 to -0.13)	-0.22 (-0.3 to -0.14)
Southeast Asia	1990	slope index of inequality	135.11 (-119.95 to 390.17)	-15.66 (-45.72 to 14.4)	-284.48 (-817.2 to 248.24)
Southeast Asia	2021	slope index of inequality	43.9 (-109.36 to 197.15)	-33.79 (-62.46 to -5.13)	-492.75 (-966.08 to -19.43)
Southeast Asia	1990	concentration index	-0.01 (-0.06 to 0.04)	-0.09 (-0.15 to -0.03)	-0.09 (-0.14 to -0.05)
Southeast Asia	2021	concentration index	0.02 (-0.03 to 0.07)	-0.05 (-0.17 to 0.06)	-0.05 (-0.15 to 0.05)
Southern Sub-Saharan Africa	1990	slope index of inequality	812.28 (169.54 to 1455.02)	-25.23 (-105.59 to 55.13)	-240.91 (-1631.54 to 1149.72)

Southern Sub-Saharan Africa	2021	slope index of inequality	123.29 (-73.25 to 319.84)	-37.72 (-128.69 to 53.26)	-686.76 (-1821.48 to 447.96)
Southern Sub-Saharan Africa	1990	concentration index	0.06 (0.04 to 0.07)	-0.06 (-0.12 to 0.01)	-0.03 (-0.09 to 0.03)
Southern Sub-Saharan Africa	2021	concentration index	0.01 (-0.01 to 0.02)	-0.04 (-0.07 to -0.02)	-0.05 (-0.07 to -0.03)
Central Latin America	1990	slope index of inequality	69.79 (-69.57 to 209.14)	0.37 (-10.38 to 11.11)	-22.25 (-197.34 to 152.84)
Central Latin America	2021	slope index of inequality	59.78 (-15.53 to 135.1)	-3.27 (-15.51 to 8.97)	-48.35 (-215.42 to 118.71)
Central Latin America	1990	concentration index	0.01 (-0.03 to 0.05)	0.01 (-0.04 to 0.05)	-0.01 (-0.05 to 0.03)
Central Latin America	2021	concentration index	0.04 (0.02 to 0.06)	-0.08 (-0.23 to 0.06)	-0.06 (-0.19 to 0.06)
Central Asia	1990	slope index of inequality	43.98 (-413.22 to 501.19)	9.17 (-88.02 to 106.36)	-114.5 (-1333.52 to 1104.53)
Central Asia	2021	slope index of inequality	210.99 (-163.71 to 585.69)	38.02 (-12.26 to 88.3)	498.02 (-584.94 to 1580.97)
Central Asia	1990	concentration index	0.01 (-0.09 to 0.11)	0.05 (-0.09 to 0.19)	0.02 (-0.11 to 0.15)
Central Asia	2021	concentration index	0.04 (-0.02 to 0.1)	0.09 (-0.02 to 0.2)	0.06 (-0.04 to 0.16)
East Asia	1990	slope index of inequality	735.35 (521.5 to 949.21)	-24.6 (-62.21 to 13)	-232.76 (-1129.57 to 664.05)
East Asia	2021	slope index of inequality	-211.87 (-427.72 to 3.99)	-50.74 (-176.6 to 75.13)	-992.8 (-2537.19 to 551.58)
East Asia	1990	concentration index	0.02 (0.01 to 0.02)	-0.01 (-0.01 to 0)	0 (-0.01 to 0)
East Asia	2021	concentration index	0 (-0.01 to 0)	-0.01 (-0.04 to 0.01)	-0.01 (-0.03 to 0)
Andean Latin America	1990	slope index of inequality	153.67 (-65.58 to 372.91)	-16.27 (-66.64 to 34.1)	-264.03 (-1162 to 633.94)
Andean Latin America	2021	slope index of inequality	0.05 (-359.86 to 359.96)	-23.24 (-42.97 to -3.51)	-338.7 (-687.09 to 9.69)
Andean Latin America	1990	concentration index	0.04 (-0.02 to 0.1)	-0.04 (-0.3 to 0.22)	-0.03 (-0.29 to 0.23)
Andean Latin America	2021	concentration index	-0.01 (-0.09 to 0.07)	-0.15 (-0.27 to -0.02)	-0.12 (-0.24 to 0)
Central Europe	1990	slope index of inequality	-51.32 (-498.22 to 395.58)	-25.62 (-121.65 to 70.42)	-450.08 (-1868.98 to 968.82)
Central Europe	2021	slope index of inequality	-411.23 (-710.02 to -112.44)	-80.27 (-128.18 to -32.35)	-1277.57 (-1957.19 to -597.94)
Central Europe	1990	concentration index	0.03 (-0.05 to 0.11)	0.01 (-0.08 to 0.09)	0.01 (-0.06 to 0.09)

Central Europe	2021	concentration index	-0.08 (-0.13 to -0.03)	-0.24 (-0.38 to -0.09)	-0.22 (-0.34 to -0.1)
Eastern Europe	1990	slope index of inequality	-59.75 (-396.72 to 277.22)	49.68 (-50.62 to 149.97)	803.16 (-250.71 to 1857.03)
Eastern Europe	2021	slope index of inequality	-86.94 (-376.45 to 202.58)	-10.24 (-59.97 to 39.49)	-297.77 (-1071.08 to 475.53)
Eastern Europe	1990	concentration index	-0.03 (-0.06 to 0)	0.06 (0.02 to 0.1)	0.05 (0.01 to 0.09)
Eastern Europe	2021	concentration index	-0.02 (-0.03 to 0)	0.04 (-0.01 to 0.08)	0.02 (-0.02 to 0.07)
Southern Latin America	1990	slope index of inequality	-364.03 (-1299.27 to 571.21)	-25.63 (-65.69 to 14.43)	-427.05 (-1060.56 to 206.46)
Southern Latin America	2021	slope index of inequality	-89.74 (-155.2 to -24.28)	-11.84 (-36.87 to 13.19)	-192.28 (-518.08 to 133.52)
Southern Latin America	1990	concentration index	-0.01 (-0.1 to 0.08)	-0.03 (-0.09 to 0.03)	-0.03 (-0.08 to 0.02)
Southern Latin America	2021	concentration index	-0.01 (-0.02 to 0)	0 (-0.15 to 0.14)	-0.01 (-0.11 to 0.09)
Western Europe	1990	slope index of inequality	203.83 (-36.29 to 443.95)	-31.8 (-61.22 to -2.38)	-379.69 (-812.59 to 53.22)
Western Europe	2021	slope index of inequality	182.59 (-24.83 to 390.02)	-3.54 (-10.18 to 3.1)	-35.52 (-139.13 to 68.08)
Western Europe	1990	concentration index	0.1 (0.05 to 0.14)	-0.06 (-0.13 to 0.01)	-0.03 (-0.09 to 0.04)
Western Europe	2021	concentration index	0.1 (0.05 to 0.14)	-0.02 (-0.07 to 0.04)	0.03 (-0.01 to 0.07)
High-income North America	1990	slope index of inequality	-747.15 (-1531.28 to 36.98)	-70.15 (-166.38 to 26.09)	-1138.85 (-2593.44 to 315.74)
High-income North America	2021	slope index of inequality	-138.95 (-249.53 to -28.38)	-24.57 (-42.06 to -7.09)	-356.7 (-565.55 to -147.84)
High-income North America	1990	concentration index	0 (-0.01 to 0)	0.01 (-0.01 to 0.03)	0 (-0.01 to 0.02)
High-income North America	2021	concentration index	-0.01 (-0.01 to -0.01)	-0.03 (-0.03 to -0.02)	-0.02 (-0.03 to -0.02)
High-income Asia Pacific	1990	slope index of inequality	-1038.75 (-1944.18 to -133.32)	-23.93 (-168.63 to 120.76)	-724.84 (-2779.69 to 1330.01)
High-income Asia Pacific	2021	slope index of inequality	46.74 (-382.75 to 476.24)	1.65 (-45.72 to 49.02)	6.65 (-710.94 to 724.23)
High-income Asia Pacific	1990	concentration index	-0.13 (-0.17 to -0.1)	-0.15 (-0.23 to -0.06)	-0.16 (-0.24 to -0.09)

High-income Asia Pacific	2021	concentration index	0.03 (0.02 to 0.04)	0.14 (0.09 to 0.2)	0.1 (0.06 to 0.14)
Sub-Saharan Africa	1990	slope index of inequality	244.42 (92.19 to 396.64)	7.59 (-9.43 to 24.61)	83.84 (-237.07 to 404.75)
Sub-Saharan Africa	2021	slope index of inequality	196.3 (73.81 to 318.79)	3.09 (-12.75 to 18.92)	31.84 (-248.35 to 312.04)
Sub-Saharan Africa	1990	concentration index	0.04 (0.02 to 0.06)	0.03 (-0.02 to 0.08)	0.03 (-0.02 to 0.07)
Sub-Saharan Africa	2021	concentration index	0.05 (0.02 to 0.07)	0.03 (-0.02 to 0.08)	0.03 (-0.02 to 0.08)
Southeast Asia, east Asia, and Oceania	1990	slope index of inequality	75.8 (-66.54 to 218.13)	-15.27 (-28.01 to -2.53)	-252.19 (-474.69 to -29.69)
Southeast Asia, east Asia, and Oceania	2021	slope index of inequality	27.7 (-123.52 to 178.93)	-25.12 (-40.06 to -10.18)	-406.77 (-665.57 to -147.96)
Southeast Asia, east Asia, and Oceania	1990	concentration index	-0.02 (-0.04 to 0)	-0.03 (-0.05 to -0.02)	-0.03 (-0.05 to -0.02)
Southeast Asia, east Asia, and Oceania	2021	concentration index	0.02 (0 to 0.03)	-0.05 (-0.09 to -0.02)	-0.05 (-0.08 to -0.02)
Latin America and Caribbean	1990	slope index of inequality	20.68 (-89.58 to 130.94)	-3.46 (-24.77 to 17.86)	-53.1 (-380.7 to 274.51)
Latin America and Caribbean	2021	slope index of inequality	44.73 (-22.49 to 111.94)	-3.84 (-18.14 to 10.45)	-73.07 (-285.02 to 138.88)
Latin America and Caribbean	1990	concentration index	0 (-0.03 to 0.02)	-0.07 (-0.13 to 0)	-0.07 (-0.14 to -0.01)
Latin America and Caribbean	2021	concentration index	0.01 (-0.01 to 0.03)	-0.13 (-0.2 to -0.06)	-0.11 (-0.18 to -0.05)
Central Europe, eastern Europe, and central Asia	1990	slope index of inequality	-93.06 (-336.92 to 150.8)	43.21 (-18.44 to 104.85)	525.72 (-431.62 to 1483.06)
Central Europe, eastern Europe, and central Asia	2021	slope index of inequality	-195.26 (-398.45 to 7.92)	-23.96 (-58.52 to 10.6)	-538.35 (-1108.14 to 31.43)
Central Europe, eastern Europe, and central Asia	1990	concentration index	-0.01 (-0.05 to 0.03)	0.1 (0.06 to 0.15)	0.09 (0.05 to 0.13)

Central Europe, eastern Europe, and central Asia	2021	concentration index	-0.05 (-0.08 to -0.03)	-0.02 (-0.09 to 0.06)	-0.03 (-0.1 to 0.03)
High income	1990	slope index of inequality	87.4 (-134.5 to 309.29)	-25.14 (-53.12 to 2.85)	-388.95 (-779.63 to 1.73)
High income	2021	slope index of inequality	81.08 (-118.79 to 280.95)	-2.25 (-7.59 to 3.1)	-27.55 (-113.56 to 58.45)
High income	1990	concentration index	0.03 (-0.02 to 0.08)	-0.09 (-0.17 to 0)	-0.07 (-0.15 to 0.01)
High income	2021	concentration index	0.07 (0.03 to 0.11)	0.01 (-0.03 to 0.05)	0.04 (0.01 to 0.08)

Note: CI=confidence interval; SDI=sociodemographic index; IS= ischemic stroke

eTable 14. Case number and ASR prediction of prevalence for ischemic stroke aged ≥20 years from 2022 to 2050 by BAPC models in global, by both sexes

Year	Sex	Number (00000s)	ASR (per 100,000)	Crude rate (per 100,000)
2021	Both	68.36 (68.24 to 68.48)	1266.05 (1263.8 to 1268.31)	1300.65 (1298.38 to 1302.93)
2022	Both	71.14 (69.69 to 72.58)	1271.31 (1245.51 to 1297.11)	1315.92 (1289.25 to 1342.59)
2023	Both	73.27 (71.44 to 75.11)	1277.18 (1245.19 to 1309.18)	1337.86 (1304.39 to 1371.33)
2024	Both	75.51 (73.07 to 77.96)	1283.19 (1241.59 to 1324.8)	1361.16 (1317.09 to 1405.23)
2025	Both	77.83 (74.58 to 81.08)	1289.34 (1235.37 to 1343.32)	1385.25 (1327.35 to 1443.16)
2026	Both	80.2 (75.96 to 84.44)	1295.64 (1227.02 to 1364.26)	1409.64 (1335.1 to 1484.19)
2027	Both	82.58 (77.18 to 87.98)	1302 (1216.76 to 1387.24)	1433.78 (1340.05 to 1527.51)
2028	Both	85.02 (78.3 to 91.74)	1308.43 (1204.84 to 1412.02)	1458.29 (1343.02 to 1573.56)
2029	Both	87.55 (79.34 to 95.76)	1314.99 (1191.49 to 1438.5)	1483.79 (1344.67 to 1622.91)
2030	Both	90.15 (80.29 to 100.02)	1321.75 (1176.85 to 1466.64)	1509.9 (1344.68 to 1675.13)
2031	Both	92.8 (81.11 to 104.49)	1328.75 (1161.01 to 1496.49)	1536.3 (1342.72 to 1729.88)
2032	Both	95.46 (81.76 to 109.16)	1335.96 (1143.9 to 1528.02)	1562.54 (1338.34 to 1786.73)
2033	Both	98.16 (82.28 to 114.04)	1343.23 (1125.45 to 1561.01)	1588.96 (1331.87 to 1846.04)
2034	Both	100.93 (82.67 to 119.19)	1350.61 (1105.75 to 1595.46)	1616.13 (1323.77 to 1908.49)
2035	Both	103.76 (82.93 to 124.6)	1358.21 (1084.91 to 1631.52)	1643.86 (1313.83 to 1973.9)
2036	Both	106.64 (83.03 to 130.25)	1366.16 (1062.98 to 1669.34)	1672.01 (1301.84 to 2042.18)
2037	Both	109.53 (82.93 to 136.12)	1374.43 (1039.88 to 1708.97)	1700.07 (1287.29 to 2112.85)
2038	Both	112.44 (82.65 to 142.23)	1382.77 (1015.44 to 1750.11)	1728.93 (1270.84 to 2187.01)
2039	Both	115.42 (82.2 to 148.64)	1391.24 (989.68 to 1792.79)	1758.61 (1252.42 to 2264.79)
2040	Both	118.46 (81.57 to 155.35)	1399.96 (962.7 to 1837.23)	1788.93 (1231.81 to 2346.05)

2041	Both	121.53 (80.73 to 162.34)	1409.11 (934.54 to 1883.68)	1819.8 (1208.79 to 2430.8)
2042	Both	124.63 (79.66 to 169.6)	1418.65 (905.08 to 1932.23)	1850.87 (1182.99 to 2518.75)
2043	Both	127.75 (78.35 to 177.15)	1428.37 (874.15 to 1982.58)	1882.29 (1154.44 to 2610.14)
2044	Both	130.94 (76.83 to 185.05)	1438.27 (841.75 to 2034.79)	1914.64 (1123.39 to 2705.89)
2045	Both	134.17 (75.06 to 193.29)	1448.52 (807.9 to 2089.13)	1947.8 (1089.62 to 2805.98)
2046	Both	137.45 (73.03 to 201.87)	1459.25 (772.6 to 2145.91)	1981.7 (1052.91 to 2910.49)
2047	Both	140.75 (70.71 to 210.79)	1470.47 (735.7 to 2205.24)	2016.02 (1012.85 to 3019.18)
2048	Both	144.08 (68.1 to 220.07)	1481.99 (697.06 to 2266.92)	2050.84 (969.37 to 3132.32)
2049	Both	147.48 (65.2 to 229.76)	1493.85 (656.61 to 2331.08)	2086.72 (922.56 to 3250.89)
2050	Both	150.94 (61.99 to 239.89)	1506.17 (614.32 to 2398.01)	2123.58 (872.18 to 3374.99)
2021	Male	34.58 (34.52 to 34.64)	1371.72 (1371.26 to 1372.18)	1329.48 (1327.2 to 1331.75)
2022	Male	35.94 (35.3 to 36.59)	1375.12 (1361.83 to 1388.41)	1344.61 (1320.55 to 1368.67)
2023	Male	36.88 (36.06 to 37.7)	1375.46 (1353.05 to 1397.87)	1361.82 (1331.5 to 1392.13)
2024	Male	37.86 (36.76 to 38.96)	1375.86 (1341.93 to 1409.8)	1380.19 (1340.12 to 1420.26)
2025	Male	38.87 (37.41 to 40.34)	1376.32 (1329.05 to 1423.6)	1399.13 (1346.4 to 1451.86)
2026	Male	39.9 (37.99 to 41.81)	1376.83 (1314.66 to 1439.01)	1418.11 (1350.26 to 1485.95)
2027	Male	40.92 (38.49 to 43.34)	1377.4 (1298.86 to 1455.94)	1436.62 (1351.45 to 1521.78)
2028	Male	41.96 (38.94 to 44.97)	1377.93 (1281.75 to 1474.11)	1455.25 (1350.78 to 1559.72)
2029	Male	43.03 (39.36 to 46.7)	1378.47 (1263.49 to 1493.44)	1474.67 (1348.98 to 1600.35)
2030	Male	44.13 (39.73 to 48.52)	1379.05 (1244.19 to 1513.91)	1494.41 (1345.69 to 1643.14)
2031	Male	45.23 (40.05 to 50.42)	1379.73 (1223.92 to 1535.53)	1514.13 (1340.57 to 1687.68)
2032	Male	46.33 (40.28 to 52.38)	1380.46 (1202.64 to 1558.27)	1533.38 (1333.22 to 1733.54)
2033	Male	47.43 (40.45 to 54.41)	1381.02 (1180.24 to 1581.79)	1552.45 (1323.95 to 1780.95)

2034	Male	48.55 (40.56 to 56.54)	1381.46 (1156.83 to 1606.08)	1571.95 (1313.31 to 1830.59)
2035	Male	49.69 (40.62 to 58.76)	1381.89 (1132.55 to 1631.23)	1591.71 (1301.15 to 1882.27)
2036	Male	50.83 (40.6 to 61.06)	1382.43 (1107.5 to 1657.36)	1611.57 (1287.29 to 1935.86)
2037	Male	51.97 (40.51 to 63.44)	1383.03 (1081.62 to 1684.44)	1631.06 (1271.29 to 1990.84)
2038	Male	53.11 (40.33 to 65.89)	1383.38 (1054.73 to 1712.03)	1651.06 (1253.85 to 2048.27)
2039	Male	54.26 (40.09 to 68.44)	1383.53 (1026.91 to 1740.14)	1671.56 (1234.95 to 2108.17)
2040	Male	55.43 (39.78 to 71.09)	1383.63 (998.33 to 1768.93)	1692.41 (1214.42 to 2170.4)
2041	Male	56.6 (39.38 to 73.83)	1383.79 (969.05 to 1798.52)	1713.5 (1192.12 to 2234.88)
2042	Male	57.77 (38.9 to 76.65)	1383.98 (939.04 to 1828.91)	1734.51 (1167.73 to 2301.29)
2043	Male	58.94 (38.32 to 79.56)	1383.92 (908.16 to 1859.68)	1755.55 (1141.32 to 2369.78)
2044	Male	60.12 (37.66 to 82.59)	1383.66 (876.47 to 1890.84)	1777.18 (1113.22 to 2441.15)
2045	Male	61.32 (36.92 to 85.72)	1383.33 (844.1 to 1922.55)	1799.26 (1083.27 to 2515.25)
2046	Male	62.51 (36.07 to 88.94)	1383.02 (811.11 to 1954.93)	1821.61 (1051.26 to 2591.96)
2047	Male	63.69 (35.13 to 92.26)	1382.68 (777.45 to 1987.92)	1843.89 (1016.88 to 2670.89)
2048	Male	64.87 (34.07 to 95.67)	1382.17 (743.06 to 2021.27)	1866.19 (980.15 to 2752.22)
2049	Male	66.06 (32.92 to 99.21)	1381.51 (708.01 to 2055.01)	1889.05 (941.32 to 2836.79)
2050	Male	67.26 (31.66 to 102.87)	1380.8 (672.38 to 2089.23)	1912.39 (900.21 to 2924.56)
2021	Female	33.78 (33.72 to 33.84)	1179.67 (1179.27 to 1180.07)	1272.41 (1270.14 to 1274.69)
2022	Female	35.19 (34.4 to 35.99)	1187.44 (1173.48 to 1201.4)	1287.86 (1258.64 to 1317.09)
2023	Female	36.39 (35.38 to 37.41)	1198.03 (1174.45 to 1221.61)	1314.44 (1277.88 to 1350.99)
2024	Female	37.65 (36.31 to 39)	1208.75 (1172.87 to 1244.63)	1342.54 (1294.56 to 1390.52)
2025	Female	38.96 (37.17 to 40.75)	1219.6 (1169.33 to 1269.87)	1371.67 (1308.7 to 1434.64)
2026	Female	40.3 (37.97 to 42.63)	1230.58 (1164.08 to 1297.09)	1401.36 (1320.27 to 1482.46)

2027	Female	41.66 (38.69 to 44.63)	1241.56 (1157.05 to 1326.06)	1431.01 (1328.9 to 1533.11)
2028	Female	43.06 (39.35 to 46.77)	1252.56 (1148.42 to 1356.71)	1461.26 (1335.42 to 1587.09)
2029	Female	44.52 (39.98 to 49.06)	1263.63 (1138.28 to 1388.98)	1492.72 (1340.46 to 1644.99)
2030	Female	46.03 (40.55 to 51.5)	1274.8 (1126.72 to 1422.87)	1525.06 (1343.69 to 1706.43)
2031	Female	47.57 (41.06 to 54.08)	1286.13 (1113.81 to 1458.45)	1558 (1344.82 to 1771.17)
2032	Female	49.13 (41.48 to 56.78)	1297.53 (1099.42 to 1495.63)	1591.07 (1343.36 to 1838.78)
2033	Female	50.73 (41.83 to 59.63)	1308.9 (1083.54 to 1534.26)	1624.68 (1339.63 to 1909.74)
2034	Female	52.38 (42.11 to 62.65)	1320.25 (1066.19 to 1574.3)	1659.36 (1334.01 to 1984.71)
2035	Female	54.07 (42.31 to 65.84)	1331.66 (1047.47 to 1615.84)	1694.89 (1326.24 to 2063.55)
2036	Female	55.8 (42.42 to 69.18)	1343.24 (1027.45 to 1659.03)	1731.15 (1316.08 to 2146.22)
2037	Female	57.55 (42.43 to 72.68)	1354.94 (1006.04 to 1703.83)	1767.6 (1302.96 to 2232.24)
2038	Female	59.33 (42.32 to 76.35)	1366.55 (983.13 to 1749.98)	1805.13 (1287.48 to 2322.79)
2039	Female	61.16 (42.11 to 80.21)	1378.07 (958.72 to 1797.42)	1843.8 (1269.53 to 2418.07)
2040	Female	63.03 (41.79 to 84.26)	1389.62 (932.92 to 1846.31)	1883.4 (1248.82 to 2517.98)
2041	Female	64.93 (41.35 to 88.51)	1401.33 (905.8 to 1896.86)	1923.84 (1225.1 to 2622.57)
2042	Female	66.86 (40.76 to 92.95)	1413.19 (877.31 to 1949.07)	1964.77 (1197.93 to 2731.61)
2043	Female	68.81 (40.03 to 97.59)	1424.94 (847.29 to 2002.58)	2006.36 (1167.29 to 2845.44)
2044	Female	70.81 (39.16 to 102.46)	1436.56 (815.76 to 2057.36)	2049.21 (1133.35 to 2965.07)
2045	Female	72.86 (38.14 to 107.57)	1448.17 (782.8 to 2113.55)	2093.24 (1095.85 to 3090.63)
2046	Female	74.94 (36.96 to 112.93)	1459.95 (748.47 to 2171.42)	2138.45 (1054.52 to 3222.39)
2047	Female	77.06 (35.59 to 118.53)	1471.85 (712.73 to 2230.98)	2184.58 (1008.9 to 3360.26)
2048	Female	79.21 (34.03 to 124.39)	1483.64 (675.44 to 2291.84)	2231.7 (958.8 to 3504.59)
2049	Female	81.42 (32.28 to 130.55)	1495.29 (636.63 to 2353.94)	2280.34 (904.19 to 3656.49)

2050	Female	83.68 (30.33 to 137.02)	1506.92 (596.35 to 2417.48)	2330.48 (844.72 to 3816.24)

Data in parentheses are 95% confidence intervals. Count data in 100000 are presented to two decimal places. ASR, age-standardized rate; BAPC, Bayesian age-period-cohort.

eTable 15. Case number and ASR prediction of deaths for ischemic stroke aged ≥20 years from 2022 to 2050 by BAPC models in global, by both sexes

Year	Sex	Number (00000s)	ASR (per 100,000)	Crude rate (per 100,000)
2021	Both	3.59 (3.56 to 3.61)	70.03 (69.57 to 70.48)	68.27 (67.82 to 68.72)
2022	Both	3.66 (3.48 to 3.85)	68.26 (64.86 to 71.67)	67.76 (64.38 to 71.14)
2023	Both	3.73 (3.49 to 3.97)	67.35 (63.05 to 71.65)	68.08 (63.74 to 72.42)
2024	Both	3.81 (3.48 to 4.13)	66.49 (60.84 to 72.15)	68.64 (62.81 to 74.48)
2025	Both	3.89 (3.46 to 4.33)	65.67 (58.32 to 73.01)	69.3 (61.55 to 77.05)
2026	Both	3.98 (3.41 to 4.55)	64.84 (55.56 to 74.11)	69.92 (59.91 to 79.92)
2027	Both	4.06 (3.33 to 4.78)	64.03 (52.62 to 75.44)	70.46 (57.9 to 83.02)
2028	Both	4.15 (3.25 to 5.05)	63.32 (49.6 to 77.04)	71.13 (55.72 to 86.54)
2029	Both	4.25 (3.15 to 5.35)	62.7 (46.52 to 78.88)	72.04 (53.45 to 90.63)
2030	Both	4.36 (3.04 to 5.68)	62.16 (43.36 to 80.95)	73.09 (50.99 to 95.19)
2031	Both	4.48 (2.91 to 6.05)	61.65 (40.11 to 83.2)	74.17 (48.25 to 100.1)
2032	Both	4.6 (2.76 to 6.44)	61.21 (36.76 to 85.66)	75.27 (45.2 to 105.34)
2033	Both	4.73 (2.59 to 6.87)	60.88 (33.34 to 88.42)	76.54 (41.92 to 111.16)
2034	Both	4.88 (2.4 to 7.35)	60.66 (29.84 to 91.48)	78.06 (38.4 to 117.72)
2035	Both	5.03 (2.18 to 7.89)	60.52 (26.21 to 94.83)	79.75 (34.55 to 124.95)
2036	Both	5.2 (1.93 to 8.47)	60.44 (22.43 to 98.45)	81.5 (30.26 to 132.74)
2037	Both	5.36 (1.64 to 9.09)	60.44 (18.48 to 102.39)	83.26 (25.47 to 141.05)
2038	Both	5.54 (1.31 to 9.77)	60.54 (14.33 to 106.75)	85.21 (20.18 to 150.23)
2039	Both	5.74 (0.94 to 10.53)	60.74 (9.95 to 111.53)	87.39 (14.33 to 160.46)
2040	Both	5.94 (0.52 to 11.37)	61.04 (5.29 to 116.79)	89.77 (7.79 to 171.75)

2041	Both	6.16 (0.03 to 12.3)	61.43 (0.31 to 122.57)	92.29 (0.46 to 184.13)
2042	Both	6.39 (0 to 13.31)	61.95 (0 to 129.01)	94.93 (0 to 197.68)
2043	Both	6.64 (0 to 14.44)	62.61 (0 to 136.2)	97.83 (0 to 212.79)
2044	Both	6.91 (0 to 15.72)	63.4 (0 to 144.21)	101.06 (0 to 229.84)
2045	Both	7.2 (0 to 17.15)	64.32 (0 to 153.16)	104.58 (0 to 248.99)
2046	Both	7.52 (0 to 18.76)	65.38 (0 to 163.2)	108.37 (0 to 270.46)
2047	Both	7.85 (0 to 20.57)	66.63 (0 to 174.58)	112.45 (0 to 294.57)
2048	Both	8.21 (0 to 22.62)	68.06 (0 to 187.48)	116.92 (0 to 322)
2049	Both	8.61 (0 to 24.98)	69.67 (0 to 202.13)	121.88 (0 to 353.49)
2050	Both	9.05 (0 to 27.69)	71.49 (0 to 218.85)	127.33 (0 to 389.64)
2021	Male	1.78 (1.76 to 1.79)	81.08 (80.96 to 81.2)	68.31 (67.85 to 68.76)
2022	Male	1.82 (1.72 to 1.91)	78.52 (76.13 to 80.9)	68.02 (64.49 to 71.54)
2023	Male	1.86 (1.74 to 1.98)	77.63 (73.74 to 81.52)	68.58 (64.09 to 73.07)
2024	Male	1.9 (1.74 to 2.07)	76.8 (71.02 to 82.59)	69.39 (63.4 to 75.39)
2025	Male	1.95 (1.73 to 2.17)	75.98 (68.03 to 83.94)	70.3 (62.36 to 78.25)
2026	Male	2 (1.71 to 2.29)	75.12 (64.8 to 85.44)	71.16 (60.9 to 81.42)
2027	Male	2.05 (1.68 to 2.42)	74.25 (61.38 to 87.13)	71.94 (59.05 to 84.83)
2028	Male	2.1 (1.64 to 2.56)	73.47 (57.89 to 89.06)	72.85 (57 to 88.69)
2029	Male	2.16 (1.6 to 2.72)	72.76 (54.33 to 91.2)	73.99 (54.84 to 93.15)
2030	Male	2.22 (1.55 to 2.9)	72.09 (50.68 to 93.5)	75.27 (52.45 to 98.08)
2031	Male	2.29 (1.49 to 3.09)	71.41 (46.92 to 95.9)	76.57 (49.75 to 103.39)
2032	Male	2.35 (1.41 to 3.29)	70.75 (43.08 to 98.42)	77.88 (46.7 to 109.05)
2033	Male	2.42 (1.33 to 3.52)	70.17 (39.2 to 101.15)	79.36 (43.4 to 115.32)

2034	Male	2.5 (1.23 to 3.78)	69.66 (35.28 to 104.05)	81.1 (39.83 to 122.38)
2035	Male	2.59 (1.12 to 4.06)	69.19 (31.29 to 107.08)	83.01 (35.88 to 130.14)
2036	Male	2.68 (0.99 to 4.37)	68.71 (27.23 to 110.18)	84.99 (31.46 to 138.52)
2037	Male	2.77 (0.84 to 4.7)	68.24 (23.1 to 113.38)	86.99 (26.5 to 147.47)
2038	Male	2.87 (0.68 to 5.06)	67.81 (18.92 to 116.7)	89.18 (21.01 to 157.36)
2039	Male	2.97 (0.48 to 5.47)	67.4 (14.68 to 120.12)	91.63 (14.89 to 168.37)
2040	Male	3.09 (0.26 to 5.91)	67 (10.38 to 123.62)	94.28 (8.03 to 180.53)
2041	Male	3.21 (0.01 to 6.4)	66.6 (6.03 to 127.18)	97.08 (0.3 to 193.87)
2042	Male	3.33 (0 to 6.95)	66.23 (1.62 to 130.83)	100.02 (0 to 208.51)
2043	Male	3.47 (0 to 7.55)	65.88 (-2.83 to 134.59)	103.23 (0 to 224.82)
2044	Male	3.61 (0 to 8.23)	65.54 (-7.34 to 138.42)	106.78 (0 to 243.2)
2045	Male	3.77 (0 to 8.99)	65.2 (-11.9 to 142.31)	110.66 (0 to 263.88)
2046	Male	3.94 (0 to 9.85)	64.88 (-16.51 to 146.27)	114.85 (0 to 287.15)
2047	Male	4.12 (0 to 10.83)	64.59 (-21.17 to 150.35)	119.4 (0 to 313.4)
2048	Male	4.32 (0 to 11.94)	64.32 (-25.88 to 154.52)	124.38 (0 to 343.33)
2049	Male	4.54 (0 to 13.21)	64.05 (-30.65 to 158.74)	129.91 (0 to 377.75)
2050	Male	4.78 (0 to 14.68)	63.78 (-35.45 to 163.02)	136 (0 to 417.36)
2021	Female	1.81 (1.8 to 1.82)	61.1 (61.01 to 61.19)	68.23 (67.8 to 68.67)
2022	Female	1.84 (1.76 to 1.93)	59.76 (58.01 to 61.51)	67.51 (64.27 to 70.75)
2023	Female	1.87 (1.75 to 1.99)	58.81 (55.92 to 61.69)	67.59 (63.39 to 71.79)
2024	Female	1.9 (1.75 to 2.06)	57.88 (53.58 to 62.18)	67.91 (62.23 to 73.59)
2025	Female	1.94 (1.73 to 2.16)	56.96 (51.06 to 62.87)	68.32 (60.76 to 75.88)
2026	Female	1.98 (1.69 to 2.26)	56.03 (48.38 to 63.68)	68.7 (58.94 to 78.46)

2027	Female	2.01 (1.65 to 2.37)	55.11 (45.6 to 64.61)	69.01 (56.78 to 81.24)
2028	Female	2.05 (1.61 to 2.49)	54.23 (42.78 to 65.69)	69.46 (54.47 to 84.44)
2029	Female	2.09 (1.55 to 2.63)	53.41 (39.93 to 66.9)	70.14 (52.1 to 88.17)
2030	Female	2.14 (1.5 to 2.79)	52.62 (37.04 to 68.21)	70.96 (49.57 to 92.35)
2031	Female	2.19 (1.43 to 2.96)	51.84 (34.11 to 69.58)	71.83 (46.79 to 96.87)
2032	Female	2.25 (1.35 to 3.14)	51.09 (31.15 to 71.03)	72.72 (43.74 to 101.71)
2033	Female	2.3 (1.26 to 3.34)	50.39 (28.19 to 72.59)	73.79 (40.48 to 107.09)
2034	Female	2.37 (1.17 to 3.57)	49.73 (25.22 to 74.24)	75.09 (37.01 to 113.17)
2035	Female	2.44 (1.06 to 3.82)	49.09 (22.24 to 75.95)	76.56 (33.24 to 119.87)
2036	Female	2.52 (0.94 to 4.1)	48.46 (19.24 to 77.69)	78.08 (29.07 to 127.08)
2037	Female	2.59 (0.8 to 4.39)	47.84 (16.22 to 79.47)	79.61 (24.45 to 134.78)
2038	Female	2.67 (0.64 to 4.71)	47.26 (13.21 to 81.31)	81.32 (19.37 to 143.26)
2039	Female	2.76 (0.46 to 5.07)	46.69 (10.19 to 83.19)	83.25 (13.78 to 152.72)
2040	Female	2.86 (0.25 to 5.46)	46.14 (7.16 to 85.12)	85.36 (7.57 to 163.16)
2041	Female	2.96 (0.02 to 5.89)	45.6 (4.14 to 87.07)	87.6 (0.63 to 174.59)
2042	Female	3.06 (0 to 6.37)	45.1 (1.11 to 89.08)	89.95 (0 to 187.08)
2043	Female	3.17 (0 to 6.89)	44.62 (-1.92 to 91.16)	92.54 (0 to 201.02)
2044	Female	3.3 (0 to 7.49)	44.16 (-4.95 to 93.28)	95.45 (0 to 216.77)
2045	Female	3.43 (0 to 8.16)	43.72 (-7.98 to 95.41)	98.63 (0 to 234.42)
2046	Female	3.58 (0 to 8.91)	43.27 (-11.02 to 97.56)	102.02 (0 to 254.11)
2047	Female	3.73 (0 to 9.74)	42.84 (-14.06 to 99.75)	105.64 (0 to 276.14)
2048	Female	3.89 (0 to 10.69)	42.43 (-17.1 to 101.97)	109.61 (0 to 301.11)
2049	Female	4.07 (0 to 11.77)	42.03 (-20.13 to 104.19)	114.01 (0 to 329.73)

2050	Female	4.27 (0 to 13.01)	41.62 (-23.16 to 106.41)	118.83 (0 to 362.48)

Data in parentheses are 95% confidence intervals. Count data in 100000 are presented to two decimal places. ASR, age-standardized rate; BAPC, Bayesian age-period-cohort.

eTable 16. Case number and ASR prediction of DALYs for ischemic stroke aged ≥20 years from 2022 to 2050 by BAPC models in global, by both sexes

Year	Sex	Number (00000s)	ASR (per 100,000)	Crude rate (per 100,000)
2021	Both	69.83 (69.72 to 69.95)	1316.91 (1314.74 to 1319.09)	1328.75 (1326.58 to 1330.93)
2022	Both	71.32 (68.27 to 74.37)	1291.38 (1236.16 to 1346.6)	1319.3 (1262.86 to 1375.73)
2023	Both	72.75 (68.87 to 76.62)	1279.57 (1211.39 to 1347.75)	1328.25 (1257.46 to 1399.04)
2024	Both	74.35 (69.17 to 79.53)	1268.73 (1180.32 to 1357.15)	1340.21 (1246.81 to 1433.62)
2025	Both	76.05 (69.15 to 82.95)	1258.55 (1144.37 to 1372.73)	1353.59 (1230.79 to 1476.4)
2026	Both	77.76 (68.79 to 86.74)	1248.58 (1104.47 to 1392.69)	1366.83 (1209.08 to 1524.57)
2027	Both	79.45 (68.07 to 90.82)	1239.22 (1061.74 to 1416.69)	1379.4 (1181.87 to 1576.94)
2028	Both	81.23 (67.12 to 95.33)	1231.07 (1017.27 to 1444.86)	1393.27 (1151.34 to 1635.19)
2029	Both	83.17 (66 to 100.34)	1224.07 (971.29 to 1476.85)	1409.54 (1118.52 to 1700.56)
2030	Both	85.22 (64.64 to 105.81)	1218.1 (923.82 to 1512.37)	1427.37 (1082.64 to 1772.1)
2031	Both	87.33 (62.99 to 111.67)	1212.89 (874.69 to 1551.09)	1445.81 (1042.81 to 1848.8)
2032	Both	89.47 (61.01 to 117.94)	1208.76 (824.07 to 1593.46)	1464.58 (998.69 to 1930.48)
2033	Both	91.74 (58.75 to 124.72)	1205.9 (772.07 to 1639.73)	1485.02 (951.07 to 2018.98)
2034	Both	94.18 (56.22 to 132.14)	1204.18 (718.5 to 1689.87)	1507.96 (900.14 to 2115.78)
2035	Both	96.74 (53.34 to 140.15)	1203.57 (663.15 to 1743.98)	1532.67 (844.99 to 2220.35)
2036	Both	99.39 (50.05 to 148.73)	1203.98 (605.75 to 1802.21)	1558.35 (784.69 to 2332.02)
2037	Both	102.08 (46.29 to 157.87)	1205.7 (546.14 to 1865.25)	1584.51 (718.54 to 2450.48)
2038	Both	104.87 (42.06 to 167.68)	1208.58 (484.01 to 1933.15)	1612.55 (646.8 to 2578.3)
2039	Both	107.83 (37.34 to 178.32)	1212.56 (418.99 to 2006.14)	1642.91 (568.94 to 2716.88)
2040	Both	110.94 (32.05 to 189.83)	1217.81 (350.73 to 2084.89)	1675.39 (484.05 to 2866.72)

2041	Both	114.19 (26.12 to 202.26)	1224.57 (278.8 to 2170.35)	1709.8 (391.14 to 3028.46)
2042	Both	117.58 (19.48 to 215.69)	1233.22 (202.81 to 2263.8)	1746.16 (289.33 to 3203.12)
2043	Both	121.14 (12.07 to 230.26)	1243.43 (122.3 to 2365.28)	1784.9 (177.81 to 3392.56)
2044	Both	124.94 (4.22 to 246.18)	1255.09 (41.11 to 2475.34)	1826.89 (61.69 to 3599.8)
2045	Both	128.95 (0.27 to 263.57)	1268.38 (2.56 to 2595.19)	1872.01 (3.94 to 3826.33)
2046	Both	133.2 (0 to 282.59)	1283.7 (0 to 2726.67)	1920.36 (0 to 4074.28)
2047	Both	137.68 (0 to 303.43)	1301.44 (0 to 2871.85)	1972.01 (0 to 4346)
2048	Both	142.42 (0 to 326.28)	1321.17 (0 to 3031.16)	2027.12 (0 to 4644.2)
2049	Both	147.47 (0 to 351.53)	1342.84 (0 to 3206.04)	2086.57 (0 to 4973.8)
2050	Both	152.85 (0 to 379.46)	1366.74 (0 to 3398.96)	2150.5 (0 to 5338.69)
2021	Male	36.74 (36.68 to 36.8)	1535.92 (1535.42 to 1536.43)	1412.69 (1410.46 to 1414.93)
2022	Male	37.64 (35.95 to 39.33)	1504.67 (1467.37 to 1541.98)	1408.07 (1344.82 to 1471.31)
2023	Male	38.45 (36.31 to 40.6)	1492.51 (1431.17 to 1553.84)	1419.97 (1340.91 to 1499.04)
2024	Male	39.36 (36.51 to 42.22)	1481.32 (1389.59 to 1573.05)	1434.96 (1330.9 to 1539.02)
2025	Male	40.32 (36.53 to 44.12)	1470.52 (1343.87 to 1597.17)	1451.31 (1314.67 to 1587.95)
2026	Male	41.28 (36.34 to 46.22)	1459.41 (1294.24 to 1624.58)	1467.28 (1291.82 to 1642.75)
2027	Male	42.22 (35.96 to 48.48)	1448.57 (1241.69 to 1655.45)	1482.47 (1262.72 to 1702.23)
2028	Male	43.22 (35.45 to 50.98)	1438.8 (1187.36 to 1690.23)	1498.94 (1229.68 to 1768.2)
2029	Male	44.29 (34.83 to 53.75)	1429.87 (1131.32 to 1728.42)	1517.82 (1193.76 to 1841.89)
2030	Male	45.42 (34.08 to 56.76)	1421.48 (1073.51 to 1769.46)	1538.2 (1154.12 to 1922.28)
2031	Male	46.57 (33.15 to 59.99)	1413.17 (1013.69 to 1812.65)	1558.98 (1109.78 to 2008.18)
2032	Male	47.74 (32.04 to 63.44)	1405.38 (952.27 to 1858.49)	1580.05 (1060.48 to 2099.61)
2033	Male	48.97 (30.77 to 67.17)	1398.4 (889.58 to 1907.23)	1602.88 (1007.11 to 2198.65)

2034	Male	50.29 (29.34 to 71.25)	1391.99 (825.54 to 1958.43)	1628.33 (949.8 to 2306.86)
2035	Male	51.69 (27.71 to 75.66)	1385.96 (760.12 to 2011.81)	1655.67 (887.56 to 2423.77)
2036	Male	53.12 (25.85 to 80.39)	1380.08 (693.2 to 2066.96)	1684.03 (819.39 to 2548.67)
2037	Male	54.58 (23.72 to 85.44)	1374.68 (624.95 to 2124.41)	1713.03 (744.57 to 2681.49)
2038	Male	56.11 (21.34 to 90.88)	1369.6 (555.38 to 2183.81)	1744.27 (663.31 to 2825.24)
2039	Male	57.72 (18.66 to 96.78)	1364.58 (484.45 to 2244.7)	1778.07 (574.9 to 2981.25)
2040	Male	59.42 (15.66 to 103.18)	1359.7 (412.26 to 2307.14)	1814.22 (478.25 to 3150.2)
2041	Male	61.2 (12.3 to 110.1)	1355.07 (338.86 to 2371.28)	1852.55 (372.21 to 3332.88)
2042	Male	63.06 (8.52 to 117.61)	1351.09 (264.28 to 2437.9)	1893.22 (255.86 to 3530.84)
2043	Male	65.02 (4.3 to 125.78)	1347.35 (188.47 to 2506.23)	1936.7 (128.02 to 3746.21)
2044	Male	67.11 (0 to 134.73)	1343.59 (111.44 to 2575.73)	1983.83 (0 to 3982.32)
2045	Male	69.34 (0 to 144.53)	1339.89 (33.25 to 2646.53)	2034.61 (0 to 4241.26)
2046	Male	71.69 (0 to 155.31)	1336.53 (-46.09 to 2719.15)	2089.25 (0 to 4525.84)
2047	Male	74.2 (0 to 167.17)	1333.85 (-126.68 to 2794.38)	2148.09 (0 to 4839.52)
2048	Male	76.87 (0 to 180.27)	1331.26 (-208.49 to 2871.01)	2211.31 (0 to 5185.69)
2049	Male	79.73 (0 to 194.79)	1328.48 (-291.39 to 2948.35)	2279.69 (0 to 5569.95)
2050	Male	82.78 (0 to 210.94)	1325.6 (-375.36 to 3026.56)	2353.4 (0 to 5997.17)
2021	Female	33.09 (33.03 to 33.15)	1129.81 (1129.42 to 1130.19)	1246.51 (1244.4 to 1248.63)
2022	Female	33.68 (32.32 to 35.04)	1107.03 (1082.08 to 1131.98)	1232.47 (1182.7 to 1282.25)
2023	Female	34.29 (32.56 to 36.03)	1095.25 (1053.83 to 1136.66)	1238.54 (1175.85 to 1301.22)
2024	Female	34.99 (32.66 to 37.32)	1084.09 (1021.99 to 1146.19)	1247.54 (1164.55 to 1330.53)
2025	Female	35.73 (32.63 to 38.83)	1073.36 (987.63 to 1159.09)	1258 (1148.74 to 1367.27)
2026	Female	36.48 (32.44 to 40.52)	1062.78 (951.07 to 1174.49)	1268.55 (1128.13 to 1408.96)

2027	Female	37.22 (32.11 to 42.34)	1052.51 (912.77 to 1192.25)	1278.57 (1102.78 to 1454.36)
2028	Female	38.01 (31.67 to 44.35)	1042.91 (873.36 to 1212.47)	1289.88 (1074.69 to 1505.06)
2029	Female	38.88 (31.16 to 46.59)	1033.94 (833 to 1234.88)	1303.6 (1044.91 to 1562.28)
2030	Female	39.8 (30.56 to 49.05)	1025.5 (791.74 to 1259.26)	1318.94 (1012.7 to 1625.18)
2031	Female	40.76 (29.84 to 51.69)	1017.46 (749.56 to 1285.36)	1335.08 (977.3 to 1692.87)
2032	Female	41.74 (28.97 to 54.5)	1009.93 (706.56 to 1313.29)	1351.61 (938.23 to 1765)
2033	Female	42.77 (27.98 to 57.55)	1002.91 (662.85 to 1342.97)	1369.71 (896.23 to 1843.18)
2034	Female	43.88 (26.88 to 60.89)	996.27 (618.41 to 1374.14)	1390.18 (851.55 to 1928.81)
2035	Female	45.06 (25.63 to 64.49)	989.96 (573.25 to 1406.67)	1412.32 (803.32 to 2021.32)
2036	Female	46.27 (24.2 to 68.34)	983.92 (527.36 to 1440.48)	1435.38 (750.73 to 2120.03)
2037	Female	47.5 (22.57 to 72.43)	978.25 (480.79 to 1475.71)	1458.74 (693.07 to 2224.41)
2038	Female	48.76 (20.73 to 76.8)	972.75 (433.49 to 1512.02)	1483.64 (630.64 to 2336.64)
2039	Female	50.11 (18.68 to 81.54)	967.37 (385.49 to 1549.24)	1510.63 (563.11 to 2458.15)
2040	Female	51.52 (16.39 to 86.65)	962.2 (336.88 to 1587.51)	1539.5 (489.73 to 2589.27)
2041	Female	52.99 (13.83 to 92.15)	957.42 (287.71 to 1627.13)	1570.09 (409.67 to 2730.5)
2042	Female	54.52 (10.96 to 98.08)	953.17 (237.97 to 1668.37)	1602.2 (322.08 to 2882.32)
2043	Female	56.12 (7.77 to 104.48)	949.06 (187.56 to 1710.56)	1636.3 (226.56 to 3046.36)
2044	Female	57.82 (4.22 to 111.45)	944.99 (136.52 to 1753.46)	1673.25 (122.09 to 3225.3)
2045	Female	59.62 (0.27 to 119.04)	940.99 (84.87 to 1797.11)	1712.82 (7.79 to 3420.08)
2046	Female	61.5 (0 to 127.29)	937.27 (32.63 to 1841.91)	1754.99 (0 to 3632.12)
2047	Female	63.48 (0 to 136.25)	933.93 (-20.23 to 1888.09)	1799.58 (0 to 3862.71)
2048	Female	65.55 (0 to 146.02)	930.53 (-73.72 to 1934.77)	1846.74 (0 to 4113.86)
2049	Female	67.75 (0 to 156.74)	926.99 (-127.74 to 1981.73)	1897.41 (0 to 4389.86)

2050	Female	70.08 (0 to 168.52)	923.43 (-182.28 to 2029.13)	1951.73 (0 to 4693.63)

Data in parentheses are 95% confidence intervals. Count data in 100000 are presented to two decimal places. ASR, age-standardized rate; DALYs, Disability-Adjusted Life Years; BAPC, Bayesian age-period-cohort.

eFigure 1. **The changes in the proportion of prevalence cases among ischemic stroke patients (IS) aged over 20 years to the overall IS patients from 1990 to 2021.**

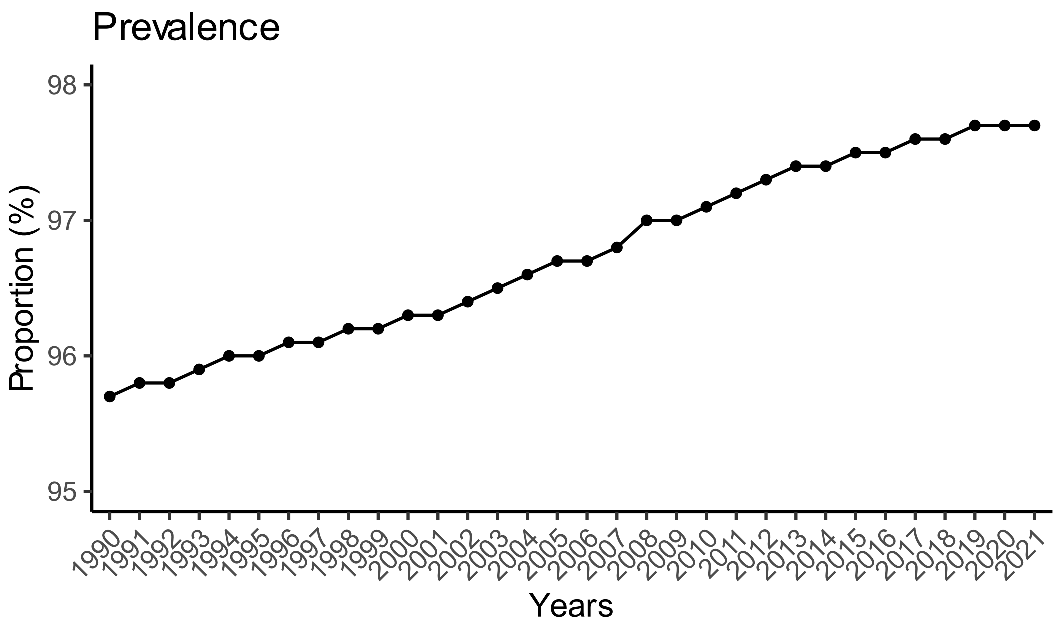

eFigure 2. Temporal trend of age-standardized prevalence (A) mortality (B) and disability-adjusted life years (C) for IS patients aged over 20 years and overall IS patients from 1990 to 2021.

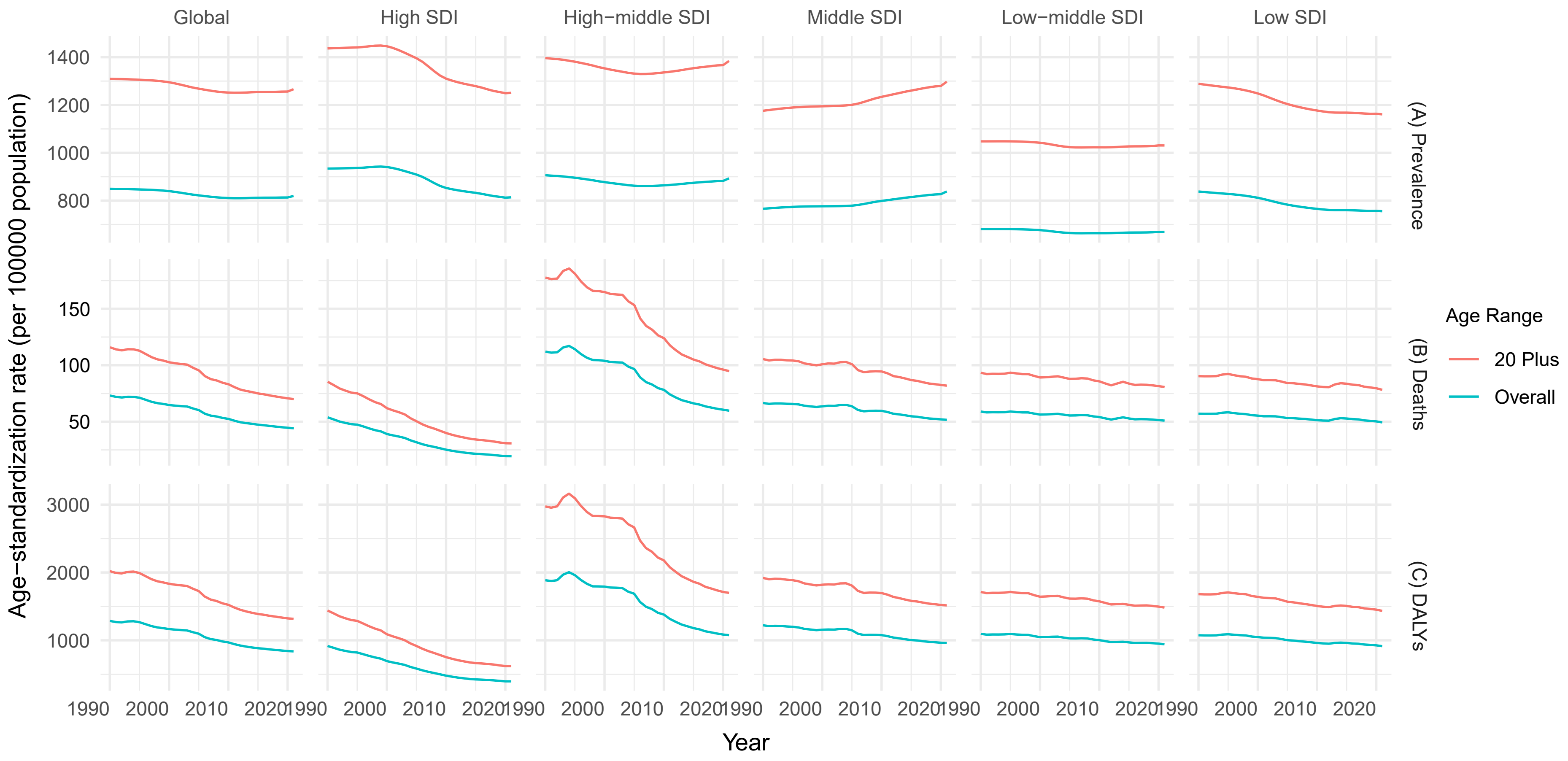

eFigure 3. The age-standardized mortality (A) and disability-adjusted life years (B) proportion of IS to all-cause in patients aged over 20 years from 1990 to 2021.

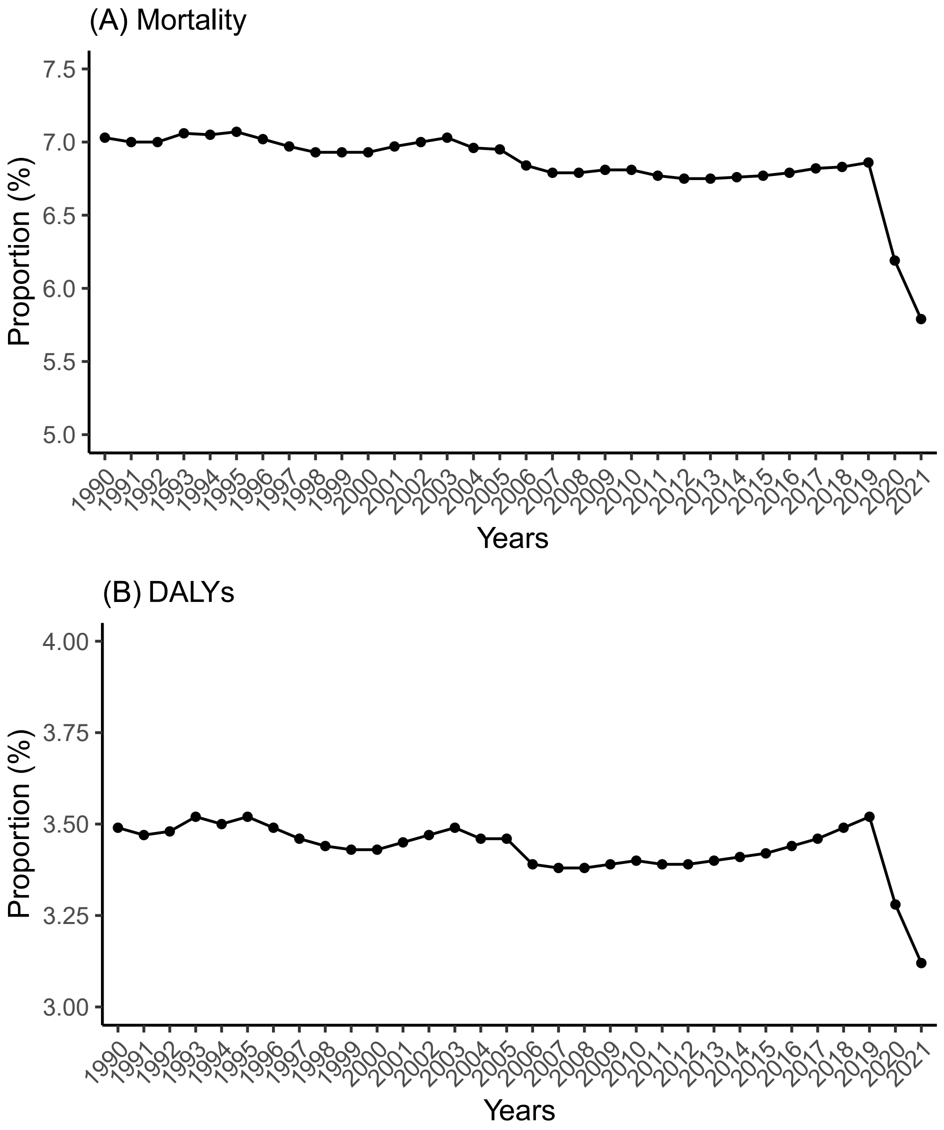

eFigure 4. Temporal trend of age-standardized mortality and disability-adjusted life years of IS in patients aged over 20 years from 1990 to 2021 at global and socio-demographic index levels by sex.

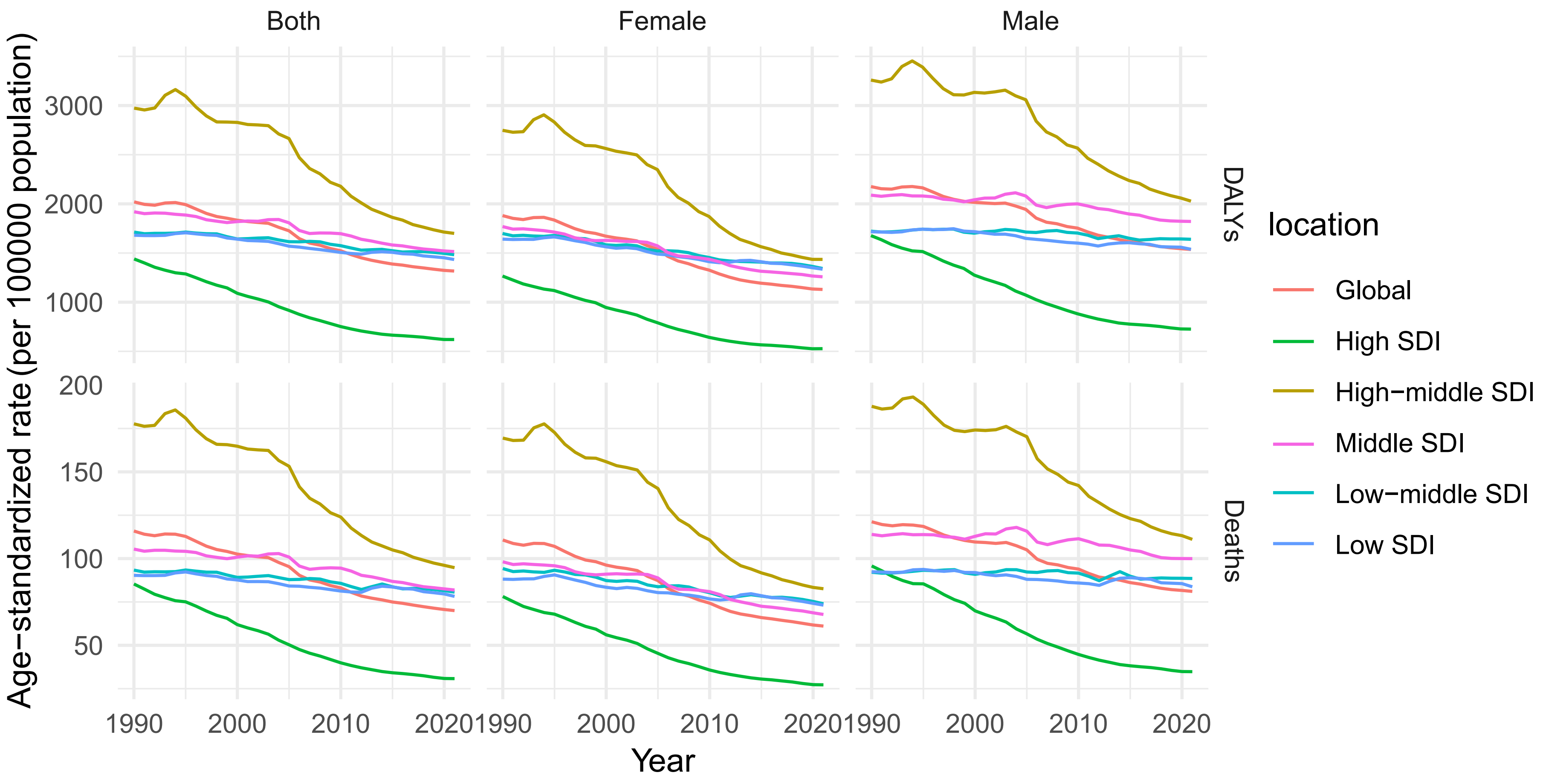

eFigure 5. Average annual percent changes of age-standardized mortality and disability-adjusted life years of IS in patients aged over 20 years from 1990 to 2021 at socio-demographic index levels by sex.

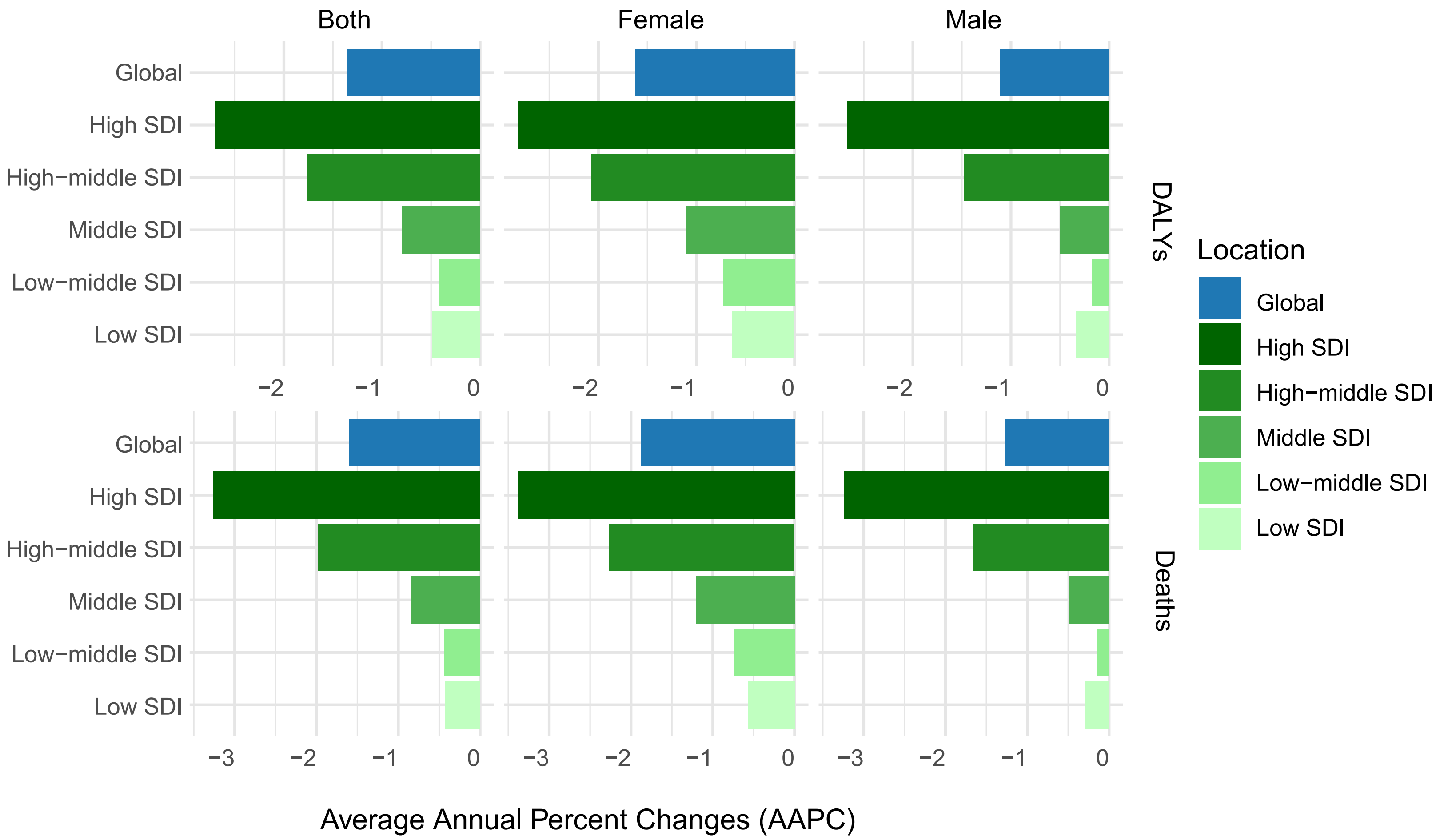

eFigure 6. Average annual percent changes of age-standardized prevalence, mortality and disability-adjusted life years of IS in patients aged over 20 years from 1990 to 2021 by sex and age.

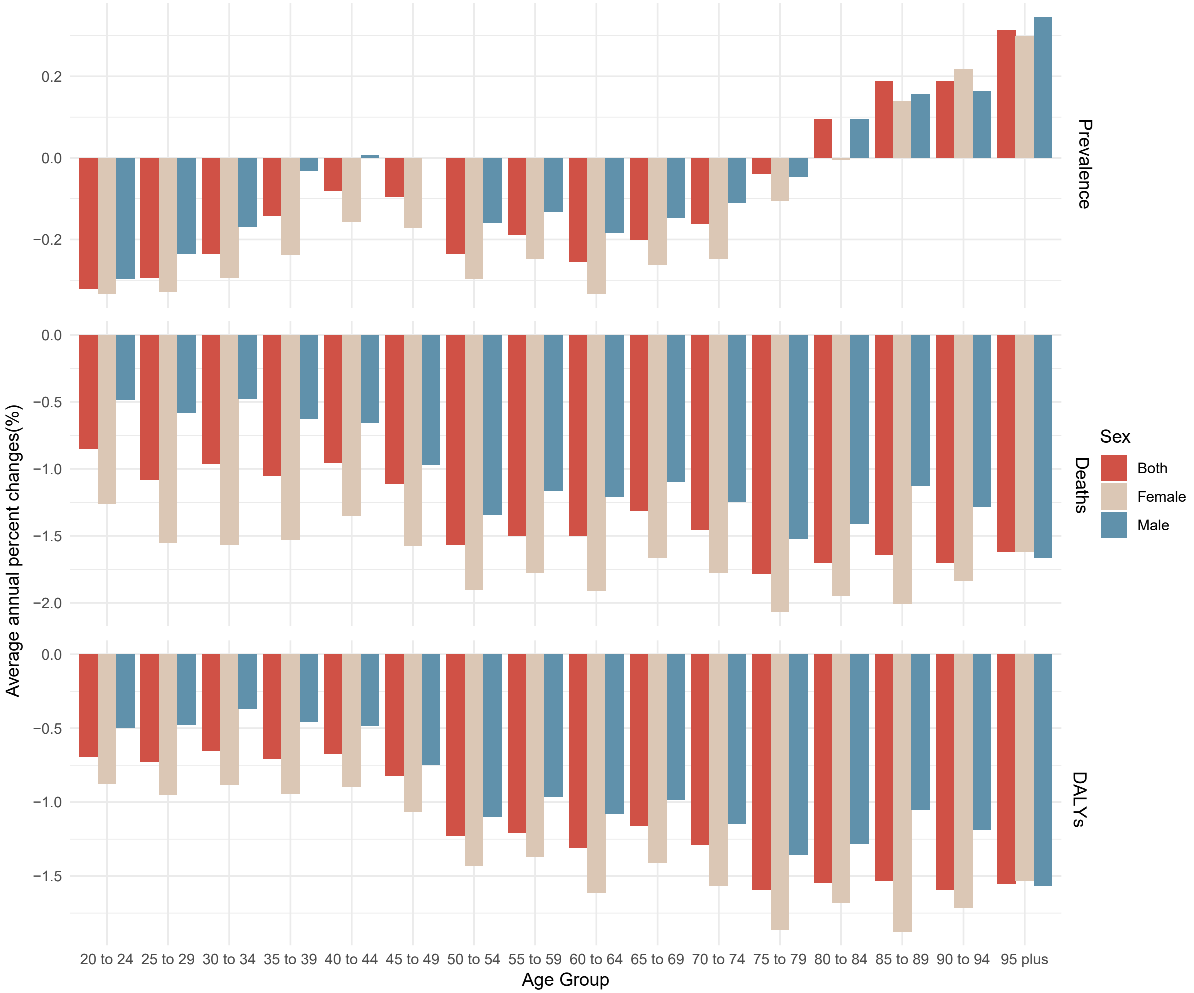

frontiers

eFigure 7. Temporal trend of age-standardized prevalence, mortality and disability-adjusted life years of IS aged over 20 years and overall IS patients from 1990 to 2021 at global and socio-demographic index levels.

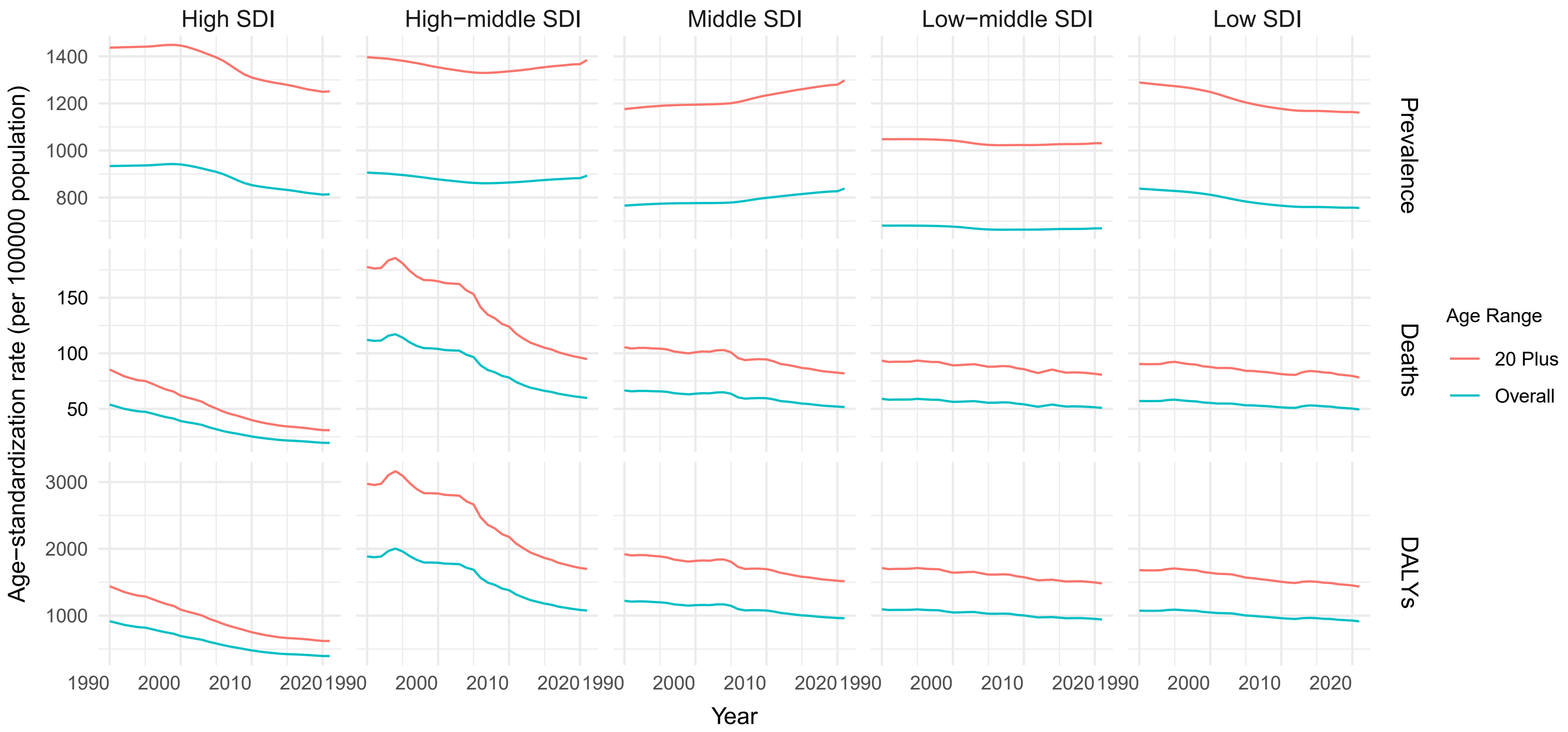

eFigure 8. Average annual percent changes of age-standardized prevalence (A), mortality (B) and disability-adjusted life years (C) of IS aged over 20 years and overall IS patients from 1990 to 2021 at global and socio-demographic index levels.

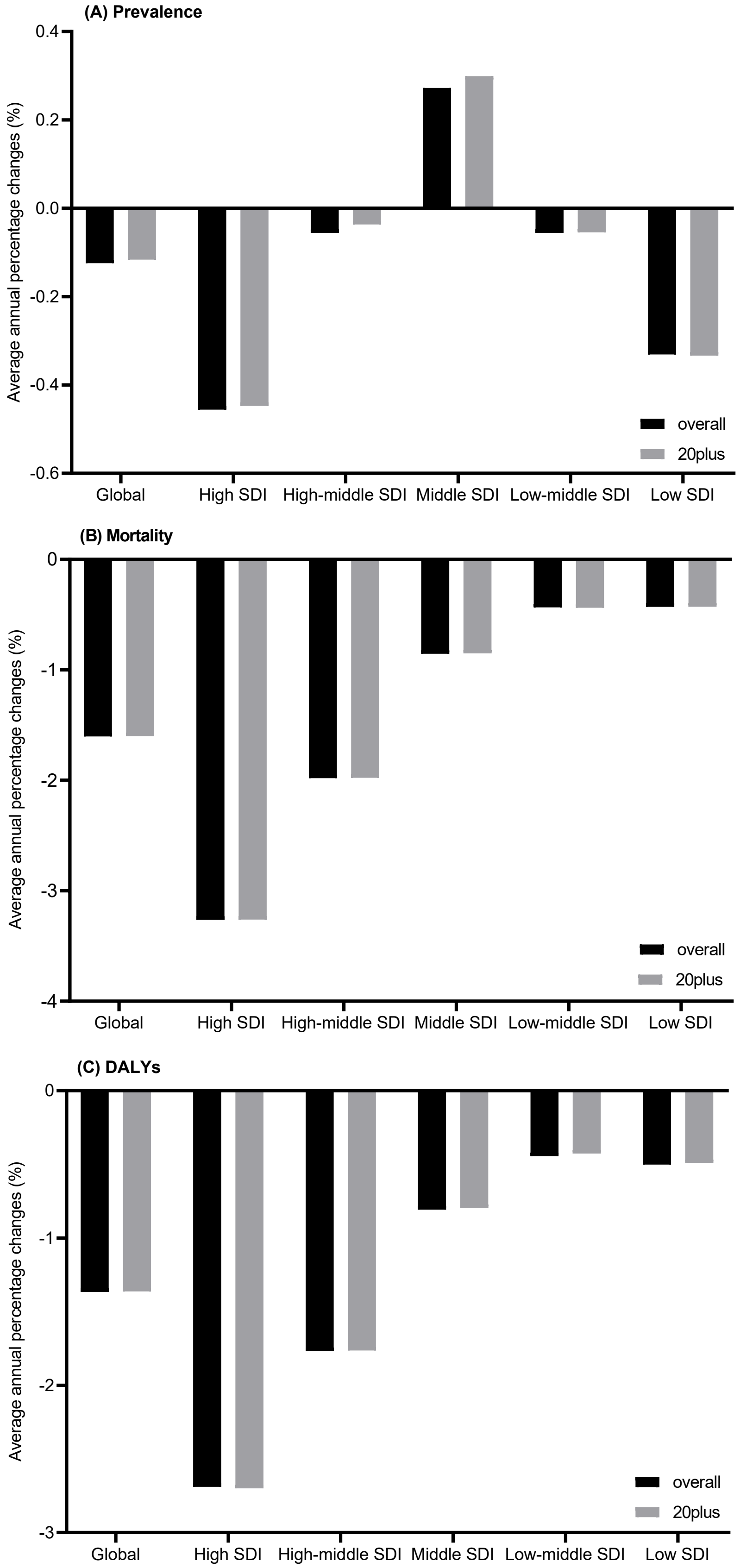
(A) Prevalence
Average annual percentage changes (%)
0.4
0.2
0.0
-0.2
-0.4
-0.6
overall
20plus
Global
High SDI
High-middle SDI
Middle SDI
Low-middle SDI
Low SDI
(B) Mortality
Average annual percentage changes (%)
0
-1
-2
-3
-4
overall
20plus
Global
High SDI
High-middle SDI
Middle SDI
Low-middle SDI
Low SDI
(C) DALYs
Average annual percentage changes (%)
0
-1
-2
-3
overall
20plus
Global
High SDI
High-middle SDI
Middle SDI
Low-middle SDI
Low SDI

eFigure 9. Prevalence (A), mortality (B) and disability-adjusted life years (C) rate of IS patients aged over 20 years from 204 countries according to the socio-demographic index in 2021.

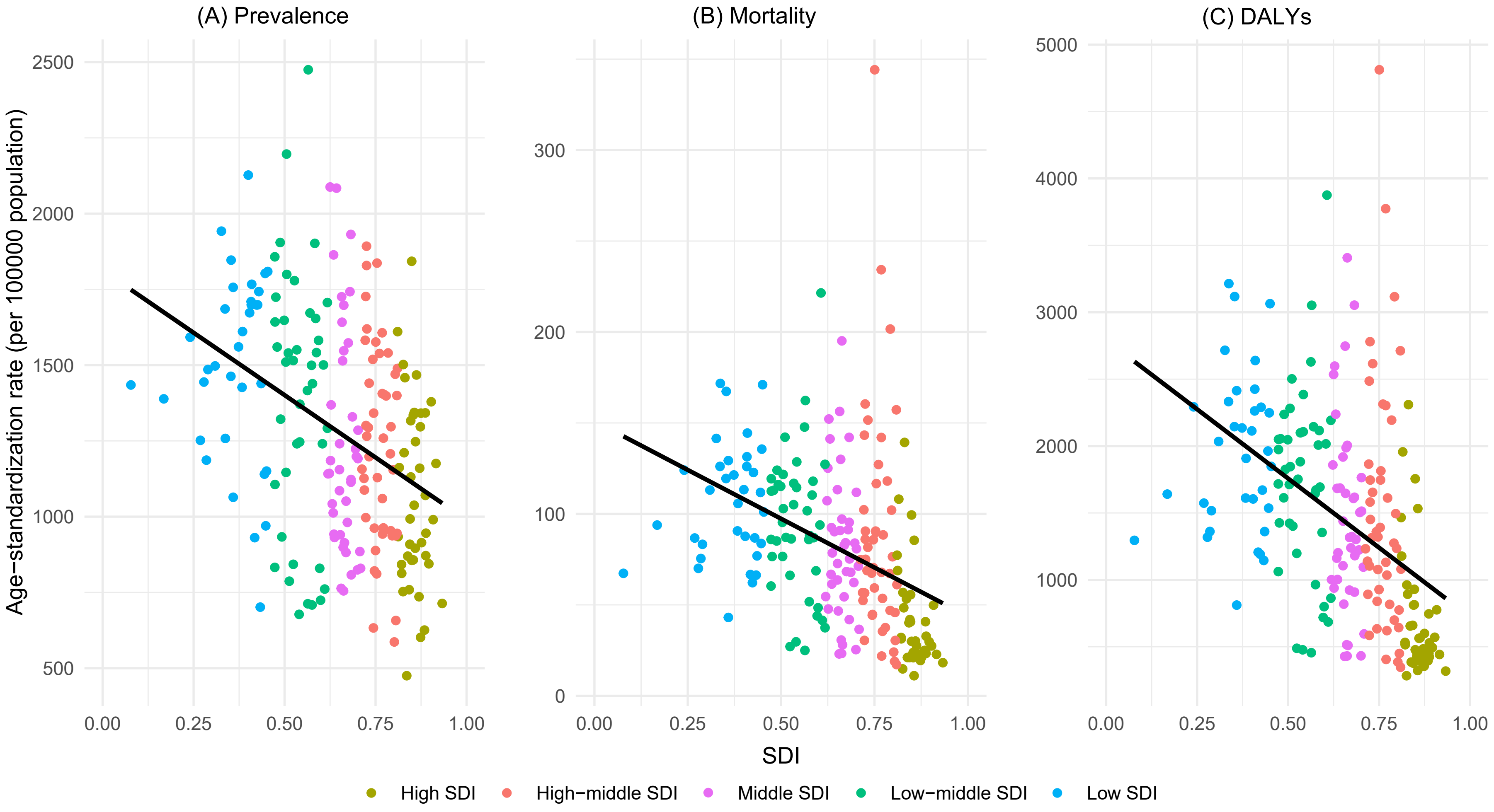

eFigure 10. Average annual percent changes of age-standardized prevalence, mortality and disability-adjusted life years of IS patients aged over 20 years from 1990 to 2021 at regions levels.

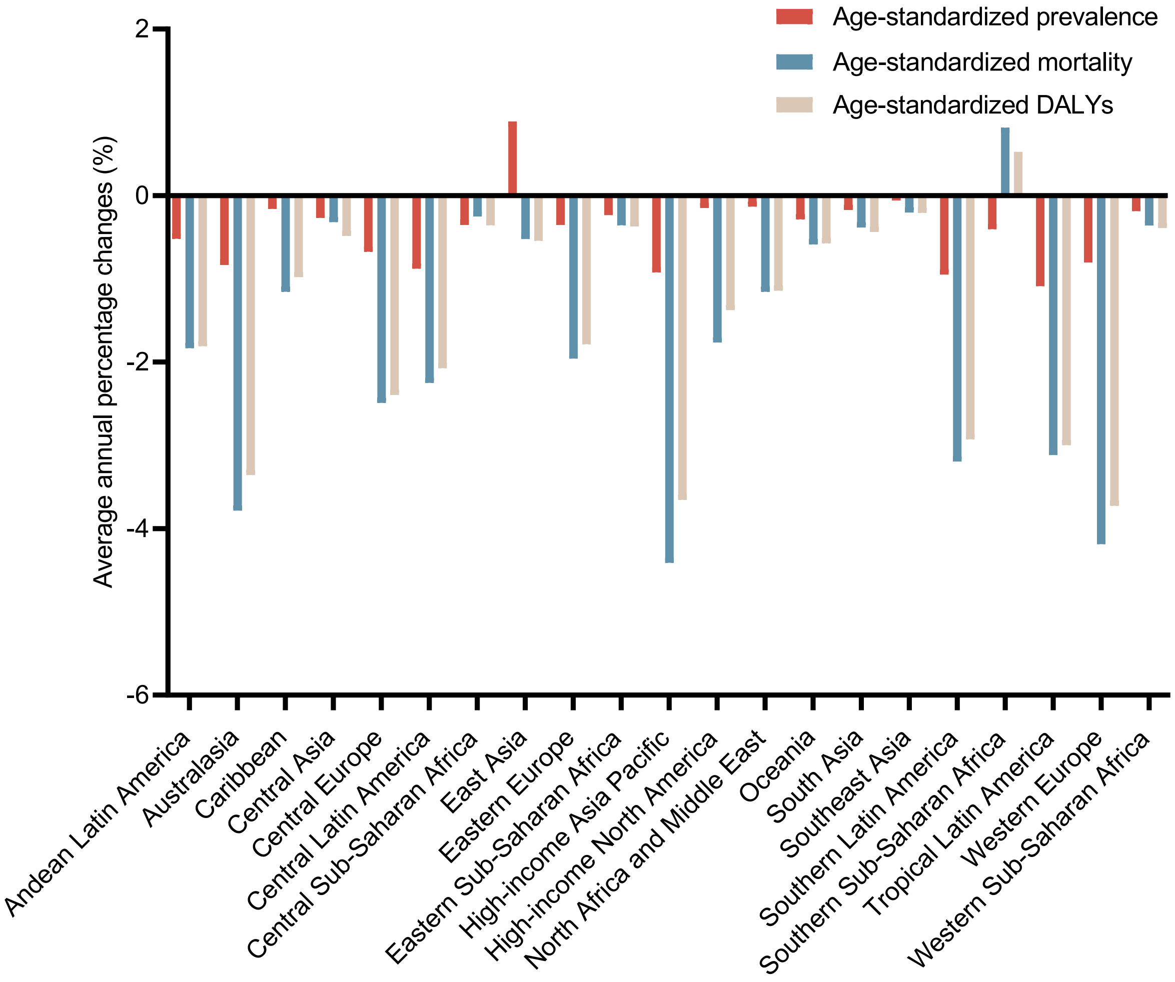

eFigure 11. Average annual percent changes of age-standardized mortality and disability-adjusted life years of IS patients aged over 20 years from 1990 to 2021 at regions levels by sex.

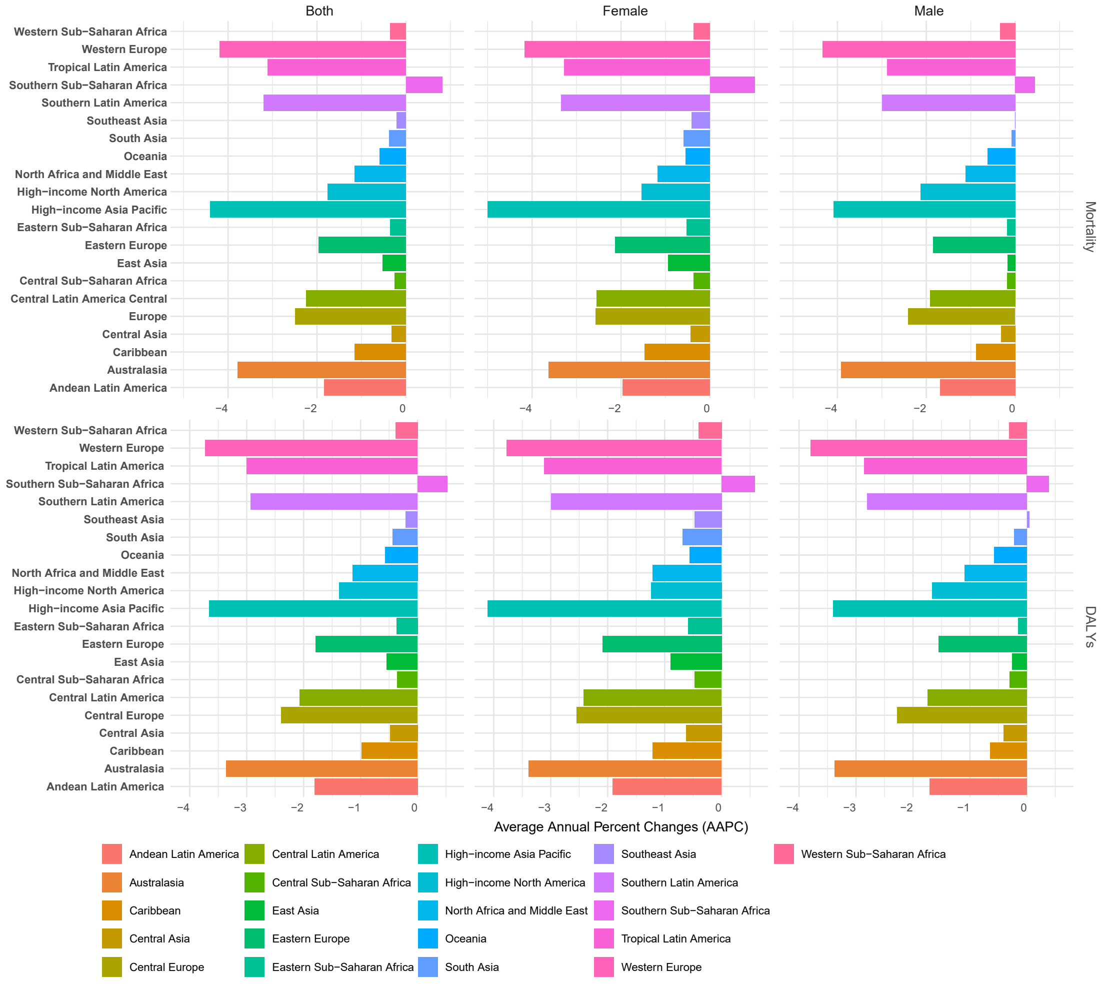

eFigure 12. Proportion of DALYs attributable to risk factors of IS patients aged over 20 years for both sexes, 2021

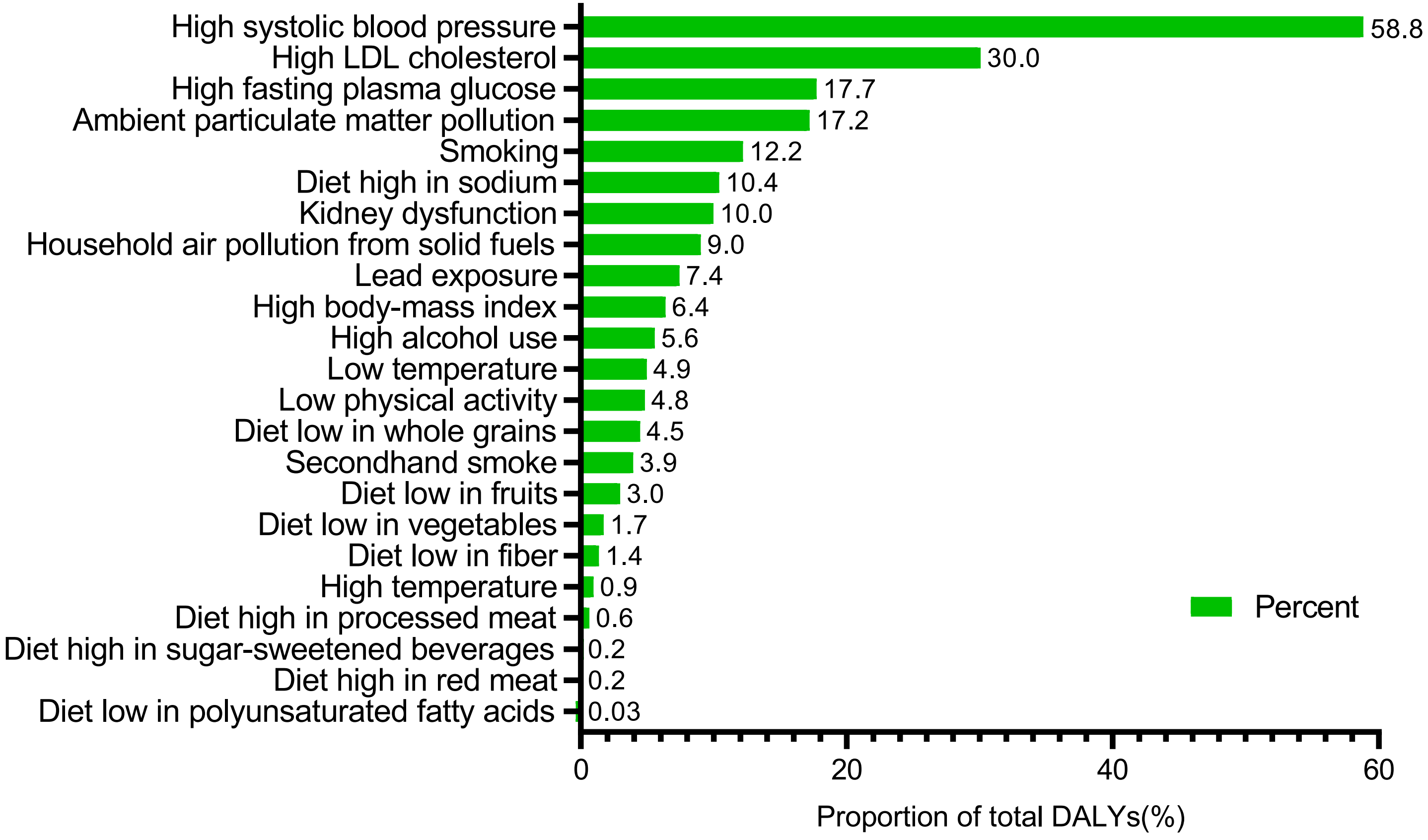

DALY= disability-adjusted life-year. LDL= low density lipoprotein

eFigure 13. Global ischemic stroke related DALYs attributable to risk factors in 1990 and 2021 (rank based on total number of IS patients aged over 20 years DALYs), by sex

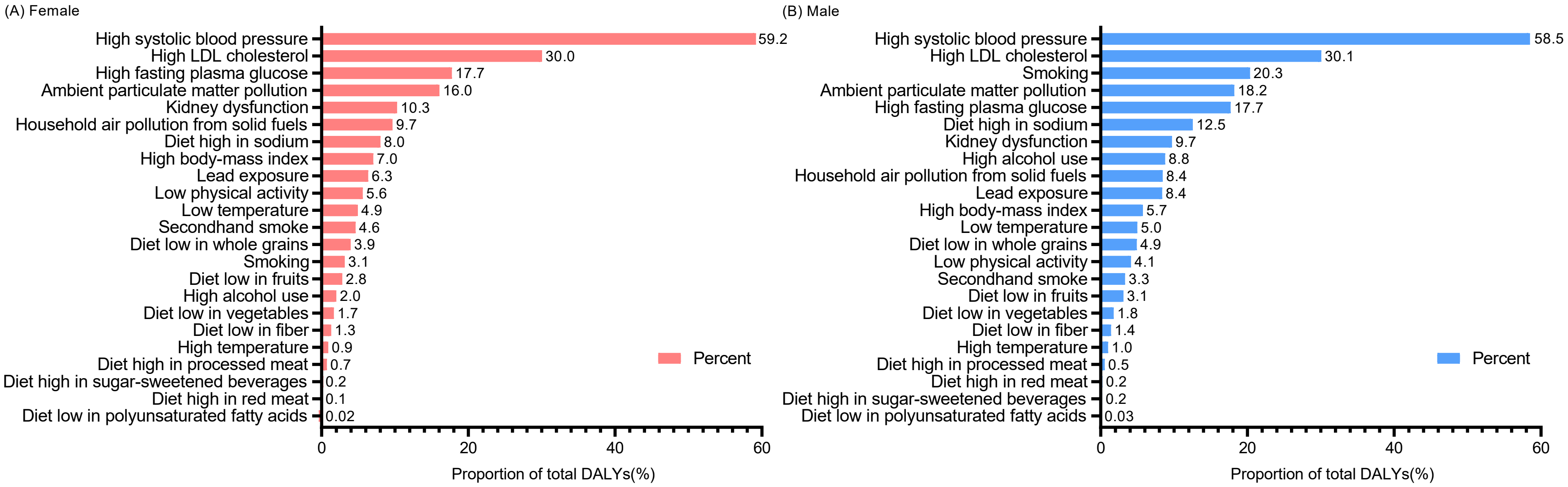

DALY= disability-adjusted life-year. LDL= low density lipoprotein.

eFigure 14. Global ischemic stroke related DALYs attributable to risk factors in 1990 and 2021 (rank based on total number of IS patients aged over 20 years DALYs)

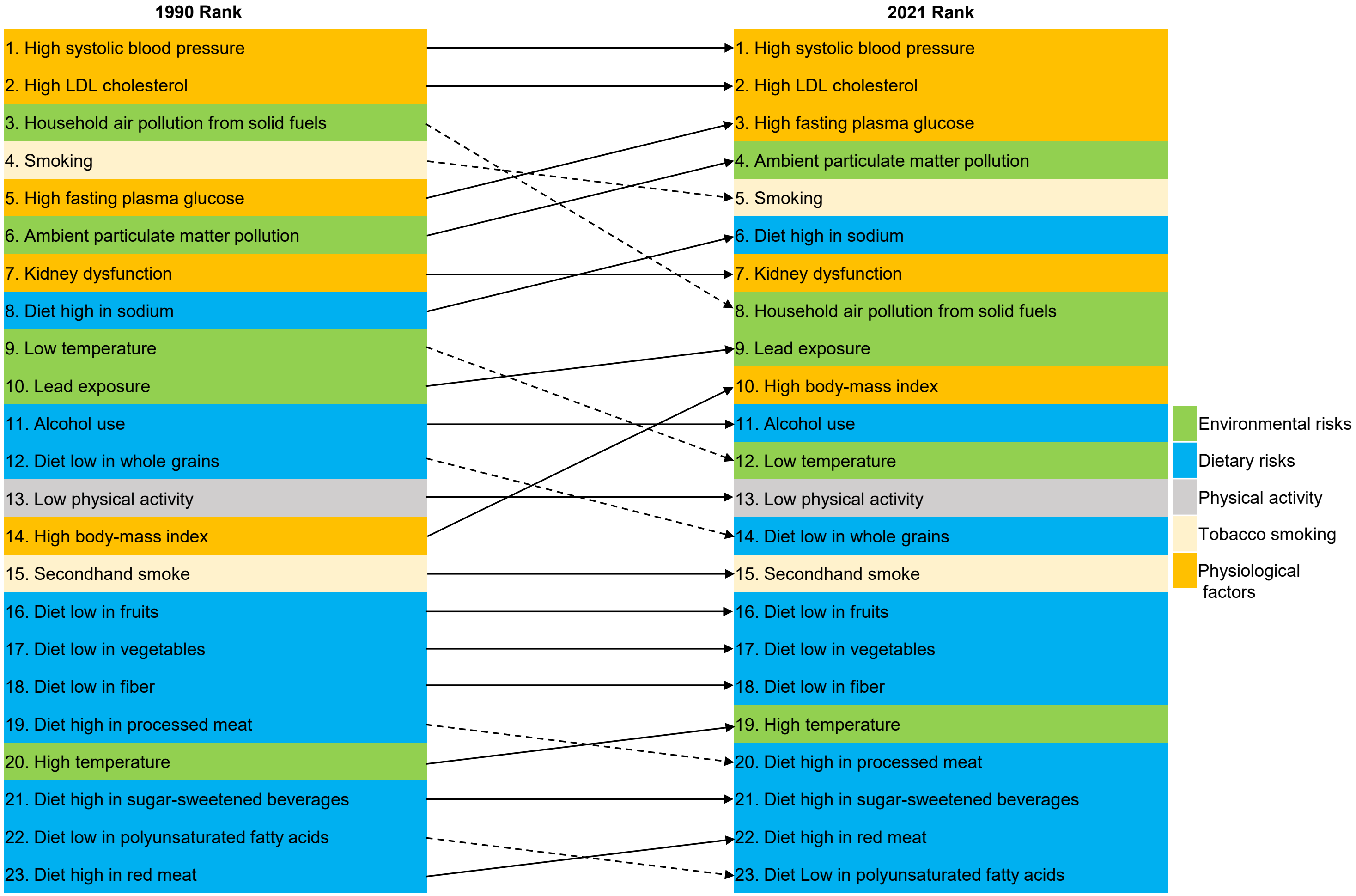

The solid line represents an increase in ranking, while the dashed line represents a decrease in ranking.

Environmental risks cluster includes ambient particulate matter pollution, household air pollution from solid fuels, low temperature, high temperature and lead exposure.
Dietary risks cluster includes diet high in sodium, diet high in red meat, diet low in fruits, diet high in processed meat, diet low in vegetables, diet low in whole grains, alcohol use, diet high in sugar-sweetened beverages, diet low in fiber and diet low in polyunsaturated fatty acids.
Metabolic risks cluster includes high body-mass index, high fasting plasma glucose, high systolic blood pressure, high LDL cholesterol and kidney dysfunction.
Behavioral risk cluster includes low physical activity.

eFigure 15. Health inequality regression curves and concentration curves for ischemic stroke aged over 20 years in terms of deaths

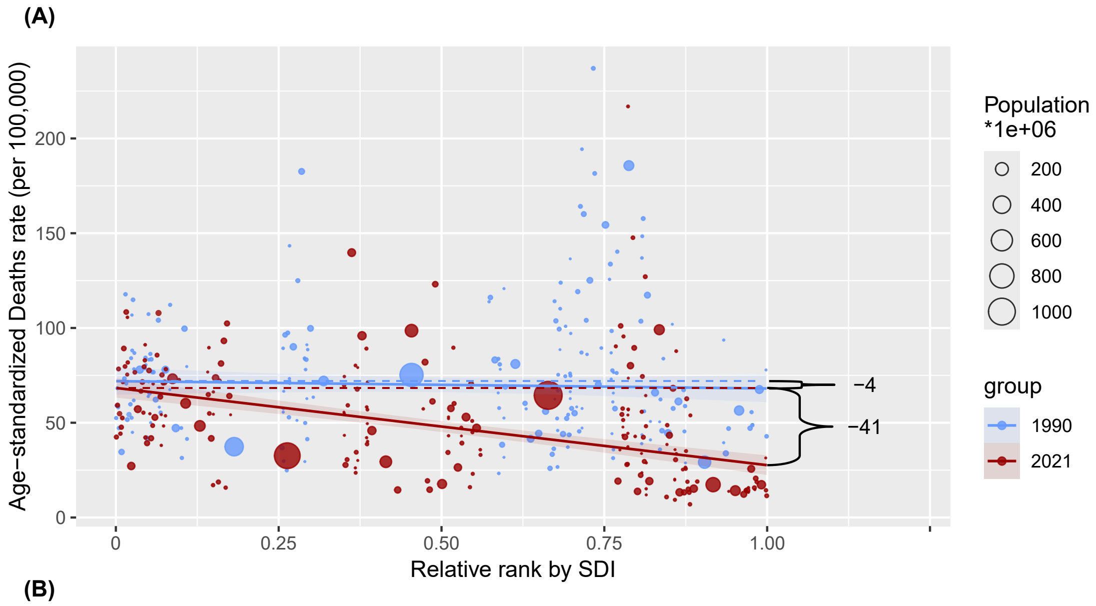

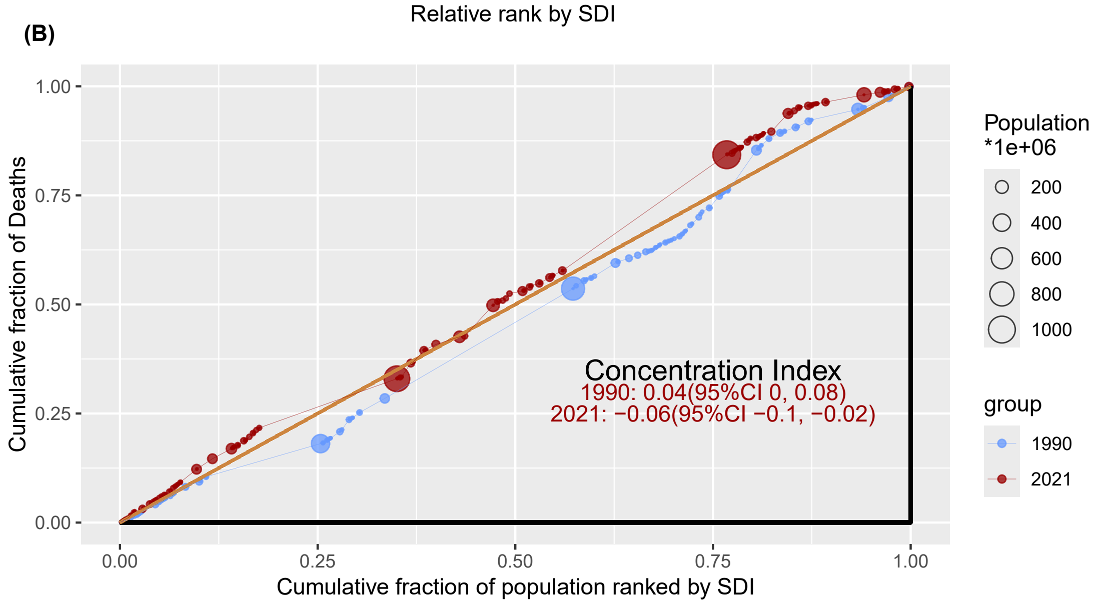

(A) Health inequality regression curves of death for ischemic stroke aged over 20 years

(B) Concentration curves of death for ischemic stroke aged over 20 years

eFigure 16. Health inequality regression curves and concentration curves for ischemic stroke aged over 20 years in terms of disease-adjusted life years (DALYs)

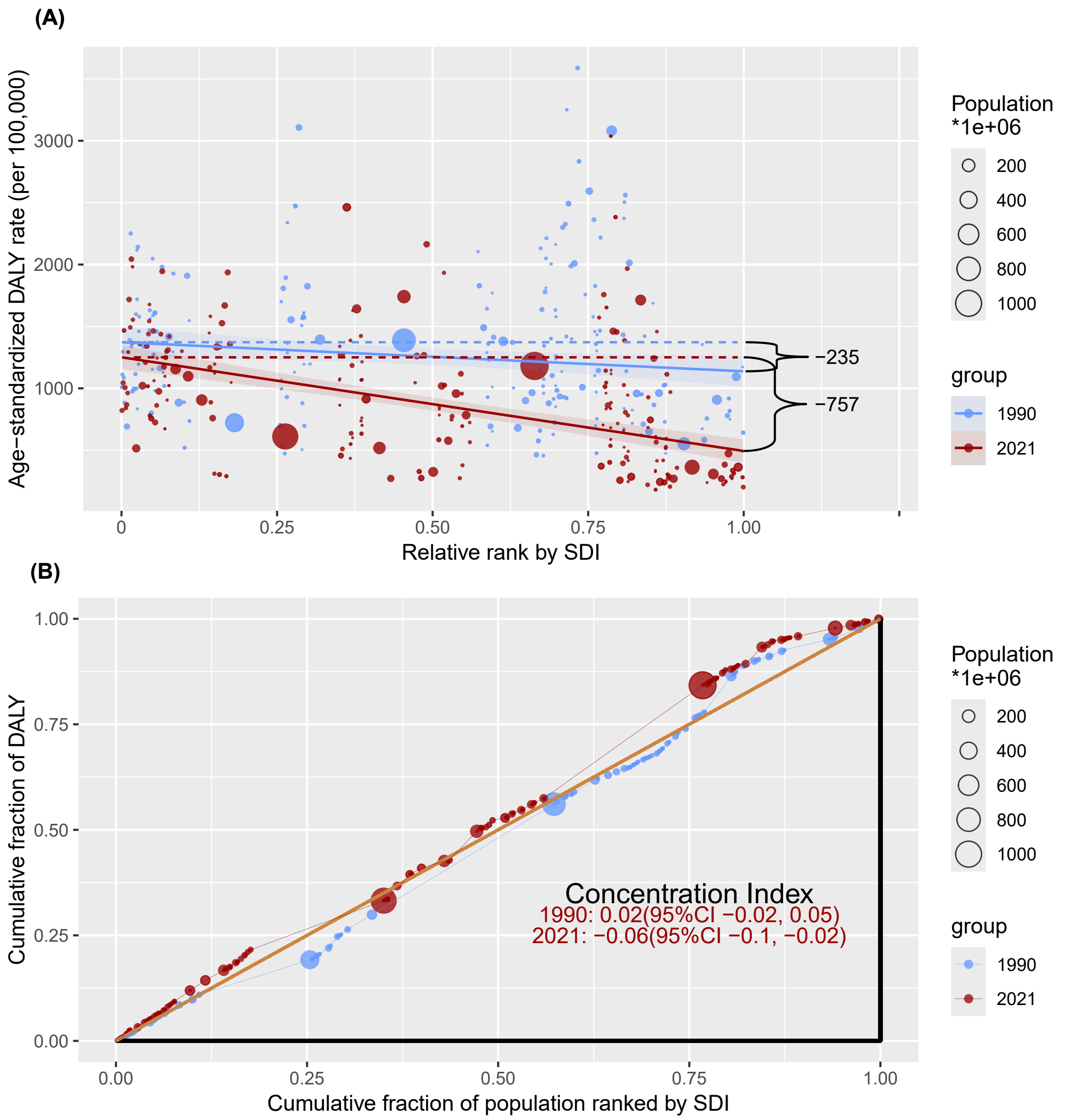

(A) Health inequality regression curves of disease-adjusted life years for ischemic stroke aged over 20 years

(B) Concentration curves of disease-adjusted life years for ischemic stroke aged over 20 years

STROBE Statement

STROBE Statement—checklist of items that should be included in reports of observational studies

	Item No.	Recommendation	Page No.
**Title and abstract**	1	(*a*) Indicate the study's design with a commonly used term in the title or the abstract	1
		(*b*) Provide in the abstract an informative and balanced summary of what was done and what was found	1-2
**Introduction**			
Background/rationale	2	Explain the scientific background and rationale for the investigation being reported	3
Objectives	3	State specific objectives, including any prespecified hypotheses	4
**Methods**			
Study design	4	Present key elements of study design early in the paper	4
Setting	5	Describe the setting, locations, and relevant dates, including periods of recruitment, exposure, follow-up, and data collection	4-6
Participants	6	*Cohort study*—Give the eligibility criteria, and the sources and methods of selection of participants. Describe methods of follow-up	6
		Cohort study—For matched studies, give matching criteria and number of exposed and unexposed	NA
Variables	7	Clearly define all outcomes, exposures, predictors, potential confounders, and effect modifiers. Give diagnostic criteria, if applicable	4-6
Data sources/ measurement	8*	For each variable of interest, give sources of data and details of methods of assessment (measurement). Describe comparability of assessment methods if there is more than one group	4-6
Bias	9	Describe any efforts to address potential sources of bias	17-18
Study size	10	Explain how the study size was arrived at	NA

Continued on next page

Quantitative variables	11	Explain how quantitative variables were handled in the analyses. If applicable, describe which groupings were chosen and why	7-8
Statistical methods	12	(*a*) Describe all statistical methods, including those used to control for confounding	7-8
		(*b*) Describe any methods used to examine subgroups and interactions	7-8
		(*c*) Explain how missing data were addressed	Supplementary Methods
		(*d*) *Cohort study*—If applicable, explain how loss to follow-up was addressed	4 Supplementary Methods
		(*e*) Describe any sensitivity analyses	NA
**Results**			
Participants	13*	(a) Report numbers of individuals at each stage of study—eg numbers potentially eligible, examined for eligibility, confirmed eligible, included in the study, completing follow-up, and analysed	9
		(b) Give reasons for non-participation at each stage	Supplementary Methods
		(c) Consider use of a flow diagram	Supplementary Methods
Descriptive data	14*	(a) Give characteristics of study participants (eg demographic, clinical, social) and information on exposures and potential confounders	9
		(b) Indicate number of participants with missing data for each variable of interest	Supplementary Methods
		(c) *Cohort study*—Summarise follow-up time (eg, average and total amount)	Supplementary Methods
Outcome data	15*	*Cohort study*—Report numbers of outcome events or summary measures over time	9
Main results	16	(*a*) Give unadjusted estimates and, if applicable, confounder-adjusted estimates and their precision (eg, 95% confidence interval). Make clear which confounders were adjusted for and why they were included	9-14
		(*b*) Report category boundaries when continuous variables were categorized	9-14
		(*c*) If relevant, consider translating estimates of relative risk into absolute risk for a meaningful time period	NA

Continued on next page

Other analyses	17	Report other analyses done—eg analyses of subgroups and interactions, and sensitivity analyses	Supplementary eTables 1-16 and Supplementary eFigures 1-16
**Discussion**			
Key results	18	Summarise key results with reference to study objectives	15
Limitations	19	Discuss limitations of the study, taking into account sources of potential bias or imprecision. Discuss both direction and magnitude of any potential bias	17-18
Interpretation	20	Give a cautious overall interpretation of results considering objectives, limitations, multiplicity of analyses, results from similar studies, and other relevant evidence	15-17
Generalisability	21	Discuss the generalisability (external validity) of the study results	17
**Other information**			
Funding	22	Give the source of funding and the role of the funders for the present study and, if applicable, for the original study on which the present article is based	8, 19

*Give information separately for cases and controls in case-control studies and, if applicable, for exposed and unexposed groups in cohort and cross-sectional studies.

Note: An Explanation and Elaboration article discusses each checklist item and gives methodological background and published examples of transparent reporting. The checklist is best used in conjunction with this article (freely available on the Web sites of PLoS Medicine at http://www.plosmedicine.org/, Annals of Internal Medicine at http://www.annals.org/, and Epidemiology at http://www.epidem.com/). Information on the STROBE Initiative is available at www.strobe-statement.org.
